# Automated Synthesis of Algal Fucoidan Oligosaccharides

**DOI:** 10.1021/jacs.4c02348

**Published:** 2024-06-25

**Authors:** Conor J. Crawford, Mikkel Schultz-Johansen, Phuong Luong, Silvia Vidal-Melgosa, Jan-Hendrik Hehemann, Peter H. Seeberger

**Affiliations:** † Max Planck Institute for Colloids and Interfaces, Am Mühlenberg 1, 14476 Potsdam, Germany; ‡ Max Planck Institute for Marine Microbiology, Celsiusstraße 1, 28359 Bremen, Germany; § MARUM, Center for Marine Environmental Sciences, University of Bremen, 28359 Bremen, Germany; ∥ Institute for Chemistry and Biochemistry, Freie Universität Berlin, Arnimallee 22, 14195 Berlin, Germany

## Abstract

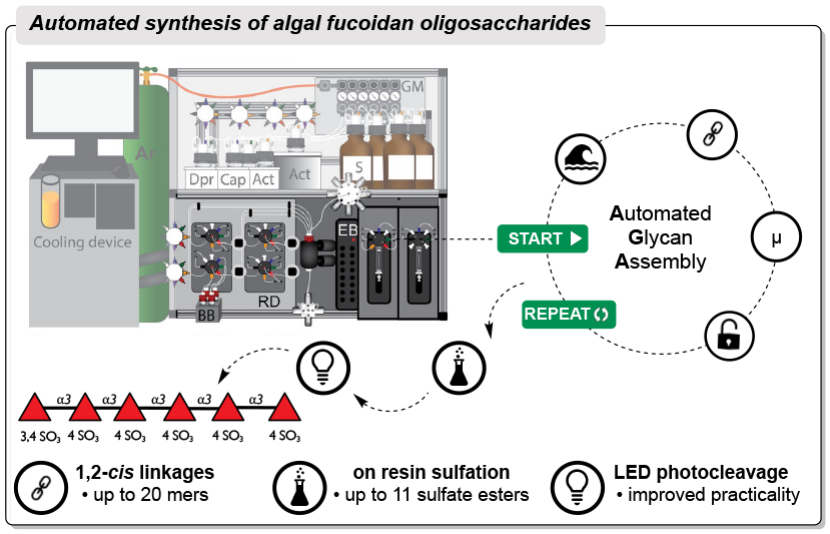

Fucoidan, a sulfated polysaccharide found in algae, plays a central role in marine carbon sequestration and exhibits a wide array of bioactivities. However, the molecular diversity and structural complexity of fucoidan hinder precise structure–function studies. To address this, we present an automated method for generating well-defined linear and branched α-fucan oligosaccharides. Our syntheses include oligosaccharides with up to 20 *cis*-glycosidic linkages, diverse branching patterns, and 11 sulfate monoesters. In this study, we demonstrate the utility of these oligosaccharides by (i) characterizing two *endo*-acting fucoidan glycoside hydrolases (GH107), (ii) utilizing them as standards for NMR studies to confirm suggested structures of algal fucoidans, and (iii) developing a fucoidan microarray. This microarray enabled the screening of the molecular specificity of four monoclonal antibodies (mAb) targeting fucoidan. It was found that mAb BAM4 has cross-reactivity to β-glucans, while mAb BAM2 has reactivity to fucoidans with 4-*O*-sulfate esters. Knowledge of the mAb BAM2 epitope specificity provided evidence that a globally abundant marine diatom, *Thalassiosira weissflogii*, synthesizes a fucoidan with structural homology to those found in brown algae. Automated glycan assembly provides access to fucoidan oligosaccharides. These oligosaccharides provide the basis for molecular level investigations into fucoidan’s roles in medicine and carbon sequestration.

## Introduction

Polysaccharides are the central metabolic fuel of the marine carbon cycle. Annually, algae sequester petagrams of carbon dioxide into a rich diversity of glycans.^1^ The unique structure of each glycan dictates its residence time and flow within marine ecosystems.^2^ Macroalgae and diatoms synthesize and secrete fucose-containing sulfated polysaccharides, termed fucoidan, into the environment.^3,4^ The structural diversity of fucoidan poses challenges to marine bacteria, necessitating evolution of complex enzymatic cascades for its degradation.^5^ Fucoidan that escapes microbial degradation can self-assemble into particles,^6^ sink to the deep ocean, and store carbon for centuries.^7,8^ Moreover, fucoidan also displays a range of biological activities that are under investigation in drug development and cosmetics.^9,10^ A current limitation is that poor knowledge exists regarding the exact molecular determinants of fucoidan that mediate a specific bioactivity or if there are precise structures in fucoidan that best mediate carbon sequestration.

To uncover the molecular mechanisms governing fucoidan carbon sequestration and its bioactivities, well-defined standards are imperative. Extraction from biological systems does not lead to homogeneous samples due to the nontemplate-encoded nature of glycans. Consequently, chemical synthesis stands out as a distinct method to obtain precisely defined organic matter.^11^ An automated process would significantly enhance the accessibility of defined fucoidan oligosaccharides. These defined standards would form the basis for a variety of investigations including: creating microarrays,^12−16^ delineating the activities of carbohydrate-active enzymes (CAZymes),^17−20^ and serving as standards for NMR experiments.^21−26^

The chemical synthesis of complex glycans is challenging,^11^ however, advances in automated approaches have enabled high-throughput assembly of both oligosaccharides and polysaccharides.^27,28^ The automated chemical synthesis of fucoidan oligosaccharides faces three primary challenges: (i) the stereocontrolled formation of 1,2 *cis*-glycosidic bonds,^29−33^ (ii) the high degree of sulfation,^34−37^ and (iii) the high reactivity of fucose glycosyl donors.^38−40^

Here, we present an automated glycan assembly (AGA) process for synthesizing well-defined fucoidan oligosaccharides, encompassing linear α-fucans up to 20-mers, branched fucoidan oligosaccharides, and glycans that contain up to 11 sulfate esters. These glycans served as standards for NMR spectroscopy, aided in delineation of CAZymes activities, and enabled the creation of a fucoidan microarray. Utilizing this microarray, we elucidated the specificity of fucoidan-directed antibodies, which, in turn, suggests brown algae-type fucoidan motifs can be found in marine diatoms.

## Results and Discussion

### Retrosynthetic Analyses and Building Block Design

**Figure 1:**
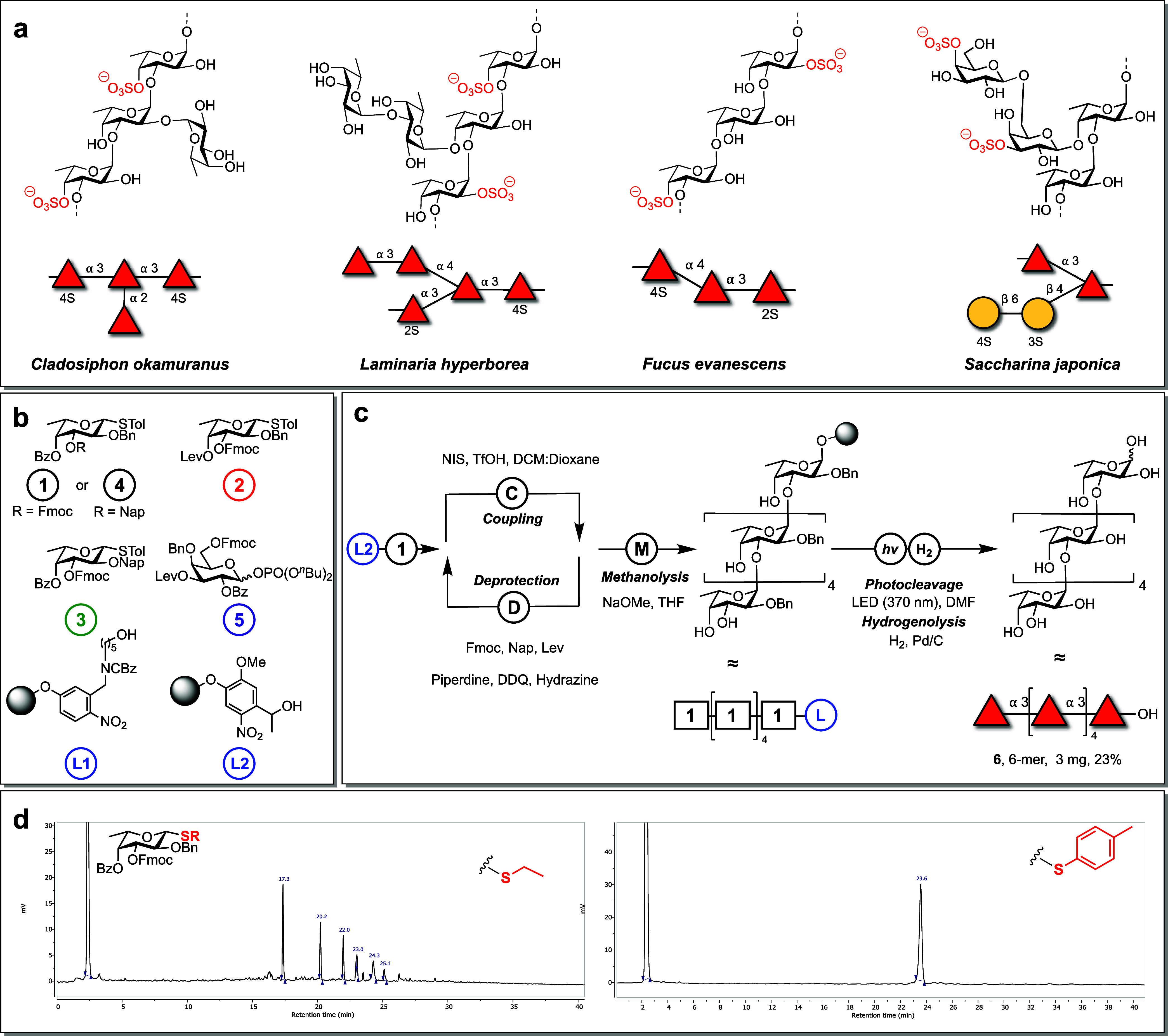
Structural diversity of fucoidan. (a) Four examples of fucoidan found in brown algae. From left: *Cladosiphon okamuranus* contains an α-1,3-backbone and is known to possess a degree of 4-*O*-sulfation.^41,42^*Laminaria hyperborea* is an α-1,3 linked fucan with a defined number of motifs including those with α-1,4 and α-1,2 branches with sulfate esters primarily on C-2 and C-4.^43^*Fucus evanescens* is an α-1,3/1,4 linked fucoidan.^44^*Saccharina japonica* is an α-1,3 linked fucoidan known to contain β-1,4 galactopyranosyl branches.^45^ (b) Building blocks used in this study. (c) Automated assembly of fucoidan hexasaccharide. (d) Representative example of HPLC traces from AGA of fucoidan hexasaccharide **6**. Left trace with a thioethyl thioglycoside, right trace with a 4-methylphenyl thioglycoside.

Homo- and heterofucans represent two classes of fucoidan. Homofucans consist of either α-(1 → 3)-linked structures or alternating α-(1→3)−α-(1→4)-linked l-fucose linkages. On the other hand, heterofucans do not have a defined glycan backbone and can consist of galactose,^46^ mannose, or glucuronic acid with fucose branches.^47^ The structural diversity of homofucans is increased by the presence of sulfate esters, acetylation, and saccharide modifications like galactose, glucuronic acid, or xylose.^41,48^ Despite the diverse structures within homofucans across brown algae, each species contains distinct motifs. For instance, fucoidan from *Laminaria hyperborea* primarily features α-(1→3)-linkages, accompanied by smaller quantities of α-(1→2) and α-(1→4)-linkages.^43^ Presently, defining precise sulfation patterns in fucoidan polysaccharides is technically challenging. However, *Cladosiphon okamuranus* possess a high degree of 4-*O*-sulfation (Figure 1a).^42^

Retrosynthetic analyses identified thioglycoside building blocks **1**–**5** as suitable candidates for assembling different fucoidan oligosaccharides (Figure 1b). A constraint on the l-fucose building block design is the requirement of nonparticipating protecting groups at the 2-hydroxyl, to allow for α-selectivity in glycosylations. Building block **1** is equipped with a nonparticipating benzyl ether on the 2-hydroxyl, a temporary fluorenylmethoxycarbonyl (Fmoc) group on the 3-*O*-position, and the 4-*O*-position was protected with a benzoate ester. The 4-*O*-benzoate ester serves as a long-range participating group (LRP) to support 1,2-*cis*-glycoside formation.^37,49−51^ Additionally, it can be cleaved on the resin to enable 4-*O*-sulfation. Building block **2** carries a 4-*O*-levulinate ester (Lev) for the formation of 1 → 4 linkages and could be utilized for precise 4-*O*-sulfation. Building block **3** bears a nonparticipating 2-naphthylmethyl ether (Nap) protecting group that can be selectively removed to install 1 → 2 linkages or 2-*O*-sulfate esters. Building block **4** used a Nap ether at the 3-OH, permitting Lev and Fmoc related protecting group manipulations elsewhere in the oligosaccharide. This Nap ether could later be removed to proceed with the backbone synthesis. Finally, building block **5** allowed for the synthesis of galactopyranoside branches.

### Altering the Thioglycoside Leaving Group Improves Automated Glycan Assembly of Fucoidan Oligosaccharides

Initially, thioethyl glycosides were used for AGA of α-(1→3)-homofucans, resulting in significant quantities of deletion sequences and reproducibility problems (SI Table 1, Figure 1c and d, SI Figure 1). Efforts to enhance the efficiency of the glycosylation by trialling different Lewis acids, varying temperatures, and employing double coupling cycles still produced inconsistent results (SI Table 1, entries 1–4). Considering that fucosyl donors are often highly reactive,^38,52^ and coupling temperatures below −40 °C are difficult to adopt at current automated synthesizers,^53^ dibutyl glycosyl phosphate donors were tested but did not improve yields (SI Table 1, entries 5 and 6).

Modifying thioglycoside reactivity by changing the protecting groups was impractical due to the structural complexity of the oligosaccharide targets that include branches and sulfate esters. Instead, the thioglycoside aglycon leaving group was modified to regulate glycosyl donor reactivity,^54^ which can adjust the activation temperature of thioglycosides by as much as +10 °C.^38^ Initially, 4-methylthiophenol thioglycosides were chosen for their availability and low cost, and this was found to enhance both the quality and reproducibility of the glycosylation modules during AGA. Under these conditions, no detectable deletion sequences by HPLC or MALDI-MS were identified (Figure 1d and SI Figure 1, SI Table 1, entries 7 and 8). The optimized glycosylation modules involved *N*-iodosuccinimide (NIS) and triflic acid (TfOH) with a reaction sequence of −20 °C for 15 min, followed by 0 °C for 35 min using five equivalents of the donor (CH_2_Cl_2_/dioxane, 2:1).

Subsequent investigations focused on assessing the stereoselectivity of glycosylations, on-resin methanolysis, photocleavage, sulfation, and hydrogenolysis.

### Automated Synthesis of Fucoidan Oligo- and Polysaccharides

**Figure 2:**
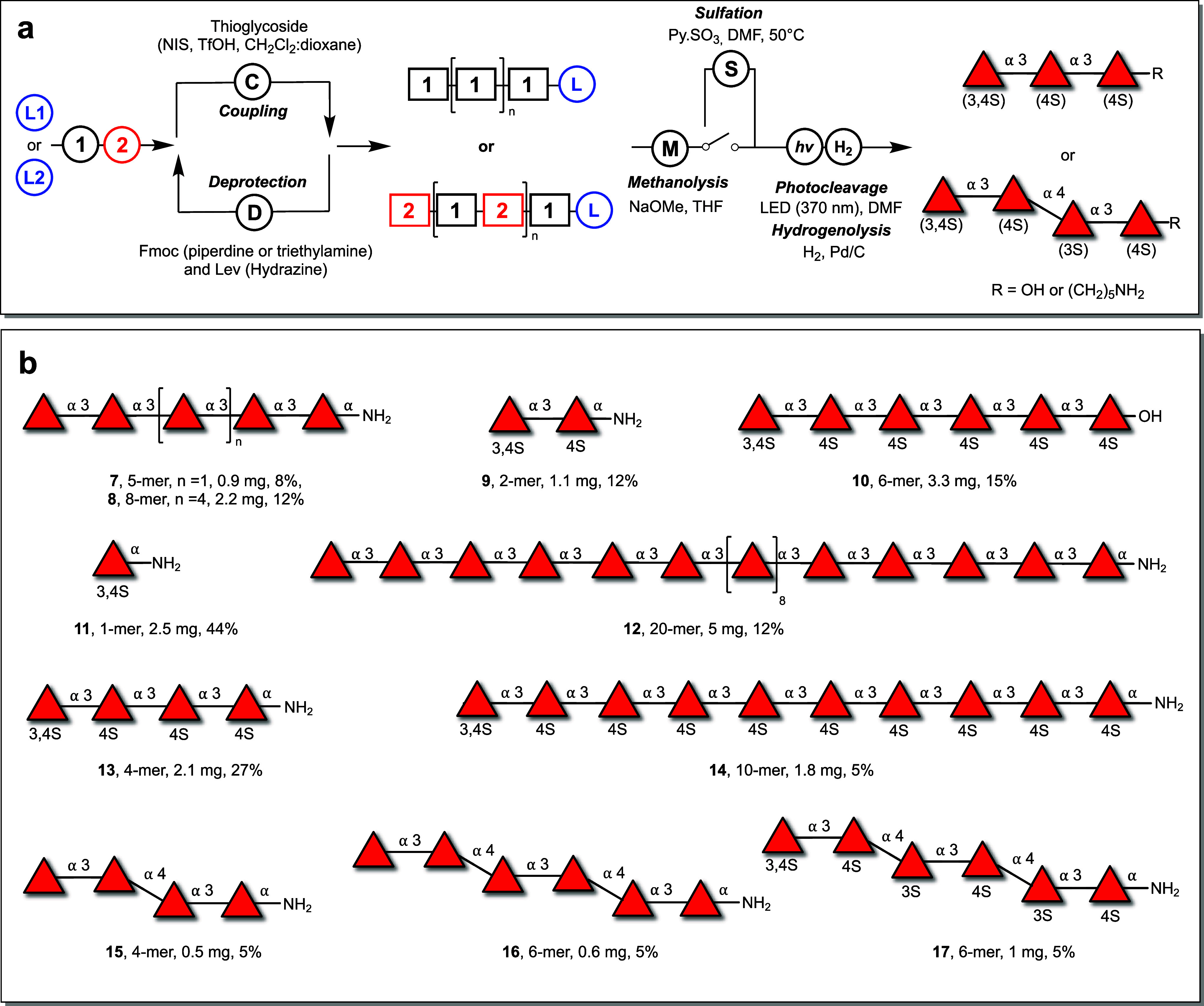
Automated glycan assembly of two major types of fucoidan backbone. (a) Automated assembly of two types of fucoidan backbone, α-1,3-linked structures and those with alternating α-1,3/α-1,4-linked l-fucose linkages. (b) Collection of fucoidan oligosaccharides synthesized.

The chemical synthesis of 1,2-*cis*-glycosides in a stereocontrolled fashion is not a solved problem.^11,29−33^ However, long-range remote assistance is useful for the synthesis of 1,2-*cis* glucosides and fucosides.^37,49,50^ Leveraging the optimized AGA conditions, a series of α-(1→3)-linked fucoidan oligosaccharides, pentamer **7**, hexamer **10**, octamer **8**, and a 20-mer **12**, were prepared (Figure 2).

Polystyrene resins were either equipped with a 5-aminopentanol to release glycans with a terminal amine for coupling to microarray surfaces or a “traceless” photocleavable linker,^55^ which permits the synthesis of free-reducing end glycosides for enzyme assays.^56^ Each coupling cycle consisted of an acidic wash with trimethylsilyl trifluoromethanesulfonate (TMSOTf), followed by NIS-TfOH promoted glycosylation. Subsequently, the resin underwent incubation with a solution of acetic anhydride (Ac_2_O) and methanesulfonic acid (MsOH) to “cap” any unreacted nucleophile (Figure 2a).^57^ For α-(1→3)-linked fucans, the temporary Fmoc protective group was removed using a piperidine solution (20% in dimethylformamide) to expose the nucleophile for the subsequent coupling cycle. The coupling cycles were reiterated five times for **7**, six times for **10**, and eight times for **8**.

For the synthesis of 20-mer fucan **12**, an initial assembly of a 10-mer was undertaken, monitoring the process by cleaving and analyzing a small resin sample via MALDI-MS and analytical HPLC (SI Figure 2a). Subsequently, the synthesis continued to reach the targeted 1,2-*cis*-linked 20-mer (SI Figure 2b and c).

The second major backbone of fucoidan comprises alternating α-fucopyranosyl-(1→3)−α-fucopyranosyl-(1→4) linkages. AGA of these oligosaccharides relied on the iterative use of building blocks **1** and **2**, leading to the production of tetramer **15** and hexamer **16**. During the synthesis of these mixed linkage oligosaccharides, the Fmoc protecting group removal module was completed by using a 20% triethylamine solution in dimethylformamide. This method was chosen as Lev esters can be sensitive to the treatment with piperidine.^28^ Smooth coupling between the axial 4-OH acceptor and thioglycoside **1** was observed without any reactivity issues (SI Figure 3).

The methanolysis module, employed for removing base-labile protecting groups, was not effective under “standard conditions”, even after extended incubation periods of 168 h (10% 0.5 M NaOMe in anhydrous THF, 5 mL, v/v).^58,59^ Utilizing a reduced volume (<5%) of sodium methoxide was necessary for efficiently removing the benzoate esters in under 16 h. Oligosaccharides larger than 10-mers necessitated prolonged incubation periods of 70 h.

Oligosaccharides **6** and **16** were released from the solid support using a flow-based photoreactor,^60^ followed by hydrogenolysis using 5% Pd/C in THF/*t*-BuOH/H_2_O (60:10:30, v/v/v).^61,62^ The two distinct fucoidan backbones were individually purified via reverse-phase HPLC (Hypercarb, gradient 0 to 80%, acetonitrile/water) to yield hexasaccharides **6** and **16**.

NMR analysis confirmed that both fucoidan backbones were prepared with complete α-selectivity, where the only observed β-linkage was associated with the free-reducing end at 4.57 ppm (d, *J* = 7.9 Hz) in **6**. Comparison of the synthetic fucoidan oligosaccharide NMR spectra to polysaccharides extracted from brown algae showed agreement.^43^ Fucoidan oligosaccharides with α-1,3-backbone shifts occurred at ∼5.06 ppm (^1^H NMR) and 95.5 ppm (^13^C NMR), while the α-1,4-backbone occurred at ∼4.96 ppm (^1^H NMR) and 100.2 ppm (^13^C NMR).^43^ The use of the nucleophilic 5-aminopentanol linker can lead to stereoselectivity issues.^63^ However, NMR analysis of compounds **7**, **8**, **9**, and **11**–**17** contain exclusively 1,2-*cis* linkages in NMR, with an α-linked anomeric proton at ∼4.88 ppm (^1^H NMR) and >98.2 ppm (^13^C NMR).

### Automated Assembly of Sulfated Fucoidan Oligosaccharides

The precise pattern and degree of fucoidan sulfation depend on a range of factors, including environmental conditions, growth stages, and extraction methods. Moreover, our understanding of how different sulfation patterns impact fucoidan’s biological functions remains limited. This is in contrast to glycosaminoglycans (GAGs), where well-defined oligosaccharides have played a pivotal role, allowing for a detailed molecular-level understanding of the roles that individual sulfate groups play in modulating bioactivity.^64−68^

**Figure 3:**
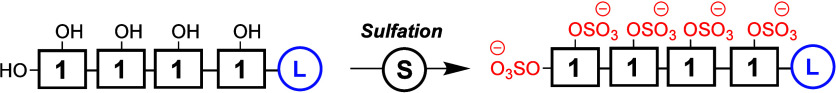
Optimization of on-resin sulfation using tetrasaccharide **13**.

Sulfated oligosaccharides prepared via AGA to date contain up to four sulfate esters.^58,69^ However, to decode glycan–protein interactions with high fidelity, sulfated tetra- to dodecasaccharides are ideal. Therefore, we initially targeted the synthesis of tetrasaccharide **13** with five sulfate esters. AGA, followed by methanolysis, provided the tetrasaccharide that was attached to the solid support. Two methods reported for on-resin sulfation did not yield the desired penta-*O*-sulfated compound (Figure 3, Table 1, entries 1–4, SI Figure 4).^53,58^ These results may be due to the low nucleophilicity of the axial C4 hydroxyl group vs primary hydroxyl groups,^53,70^ or the steric demands involved in placing several sulfate esters in close proximity on the solid support.^58^

**Table 1:** On Resin Sulfation Optimization

entry	solvent	reagent	temperature	time	comment	reference
1	DMF	NMe_3_·SO_3_	90 °C	30 min (2 cycles)	incomplete	(53)
2	DMF	NMe_3_·SO_3_	90 °C	90 min (6 cycles)	incomplete	(53)
3	DMF:Py (1:1)	Py·SO_3_	40 °C	12 h	incomplete	(58)
4	DMF	Py·SO_3_	50 °C	16 h	irreproducible	this work
5	DMF	NEt_3_·SO_3_	50 °C	16 h	irreproducible	this work
6	DMF:Py (80:20)	Py·SO_3_	50 °C	16 h	complete	this work
7	DMF:NEt_3_ (80:20)	NEt_3_·SO_3_	50 °C	16 h	complete	this work

Solution-phase syntheses of sulfated glycans, such as GAGs, can necessitate prolonged reaction times,^50,64^ that in turn renders sulfation in an automated fashion not always practical (Table 1, entries 1 and 2).^53^ While, a recently published on-resin approach, conducted in plastic syringes could not provide the precise atmospheric and temperature control necessary for extended reaction times (Table 1, entry 3).^58^ Sealable silanized microwave vials proved ideal for carrying out long sulfation reactions in an aluminum heating block (SI Figure 16, Table 1, entries 4–7).

In sealed microwave vials, both sulfur trioxide pyridine (Py·SO_3_) and triethylamine (NEt_3_·SO_3_) complexes yielded comparable results (Table 1, entries 4 and 5). Prebuffering the sulfation solution with an appropriate base,^71^ such as pyridine for sulfation reactions using Py·SO_3_, helped reduce the batch-to-batch variability in the quality of sulfur trioxide reagents (Table 1, entries 6 and 7).^72^ Even then sometimes, multiple cycles of the sulfation module were required to achieve full sulfation, for example, hexa- (**10**) and decasaccharides (**14**).

To monitor solid-phase sulfation reactions, microcleavage must be performed, which releases minute quantities of oligosaccharides for HPLC and MS analysis. Here, we employed dimethylformamide (DMF) as a photocleavage solvent, for its good swelling of polystyrene resins and its ability to solubilize the released sulfated glycans.^73,74^ Released glycans were analyzed using quadrupole time-of-flight mass spectrometry (Q-TOF MS, SI Figure 5) and reverse-phase HPLC (C5 Luna, 5% ACN to 100), with the HPLC analysis only effective for oligosaccharides with fewer than six sulfate esters (SI Figure 4).

Using these conditions (see SI, modules h1 and h2), solid-phase sulfation from a mono- to a decasaccharide was completed, with oligosaccharide **14** containing 11 sulfate esters (**9**–**11**, **13**, **14**, and **17**). ^1^H–^13^C HSQC NMR analysis confirmed the sulfation of the 4-OH, with the carbon atoms shifting characteristically downfield compared to nonsulfated fucoidan oligosaccharides from 68 to 79 ppm.

### Photocleavage Using a LED Lamp Allows for Parallel Cleavage of Multiple Resins

Reported approaches for cleaving sulfated oligosaccharides from the solid support involve a mercury lamp flow-reactor setup, utilizing a DCM–methanol mixture.^58,60^ This approach requires multiple passages through the flow cell, which are required to achieve good photocleavage results;^60^ this may be due to poor resin-swelling properties of methanol.^58,60^ Similar results were obtained when sulfated oligosaccharide **10** was cleaved from the solid support using photolysis. Therefore, we alternatively employed an LED lamp (370 nm) with DMF as a photocleavage solvent.^75,76^ DMF was chosen as it can solubilize the released amphiphilic sulfated glycans due to its good resin-swelling properties.^73,74^ The LED lamp can facilitate the parallel cleavage of multiple resins (SI Figure 6).

Following the release of the oligosaccharides from the solid support, the crude material was subjected to hydrogenolysis (5% Pd/C, THF/t-BuOH/H_2_O, 50:20:30, v/v/v), and the sulfated glycans were purified using RP-HPLC, size-exclusion chromatography, or a combination of both methods. The choice of purification method depended on the number of sulfate esters on the oligosaccharide. Glycans containing more than five sulfate esters were best purified using a combination of size-exclusion chromatography and HPLC. Using this approach, a series of sulfated fucans (**9**-11, **13**, **14**, and **17**) with different backbones, lengths, and sulfation patterns was prepared (Figure 2b).

### Automated Assembly of Branched Fucoidan Oligosaccharides

Brown algae synthesize a diverse range of fucoidans with distinct branching and sulfation patterns. The utility of the AGA platform to synthesize such branched fucoidans was demonstrated by four examples. These glycans contain α and β branching residues and cover the branching patterns found in fucoidan (2-OH, 3-OH, and 4-OH).

**Figure 4:**
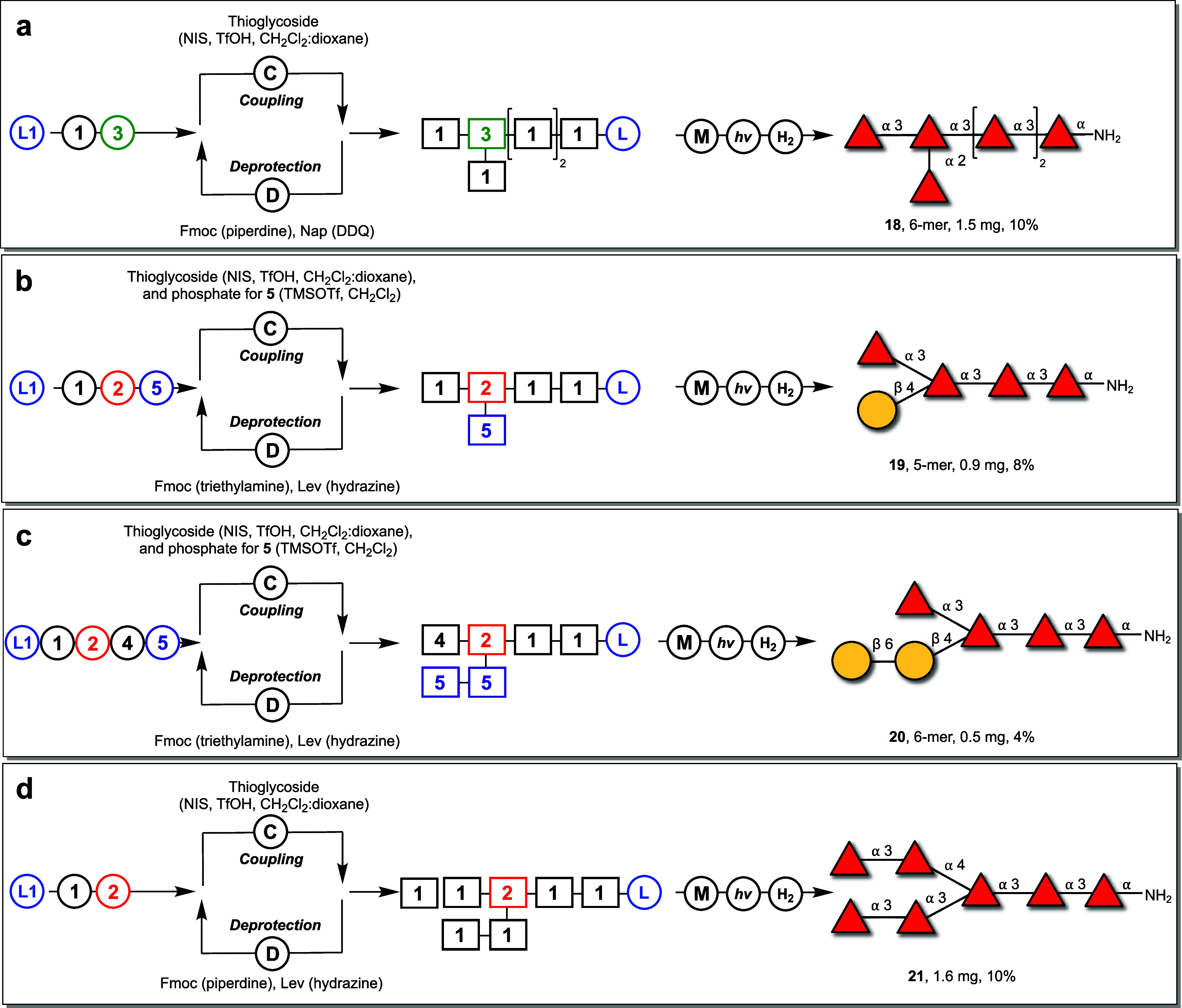
AGA of branched fucoidan oligosaccharides. (a) Synthesis of oligosaccharide **18** with a α-(1→2)-branch found in *Fucus vesiculosus*. (b) Synthesis of an oligosaccharide **19** with a β-(1→4)-galactopyranoside branch found in *Saccharina japonica*. (c) Synthesis of oligosaccharide **20** with a β-(1→6)–gal-β-(1→4)-galactopyranoside found in *Saccharina japonica*. (d) Synthesis of oligosaccharide **21** with a α-(1→4)-branch found in *Laminaria hyperborea*.

α-(1→2)-Fucopyranoside branches occur in various brown algae species, including *Cladosiphon okamuranus* and *Laminaria hyperborea* (Figure 1a). Therefore, we prepared hexasaccharide **18** that contains this motif (Figure 4a).^48^ Two cycles of building block **3** were required to fully convert the acceptor trisaccharide to the desired tetrasaccharide (SI Figure 11). Selective oxidative cleavage of the Nap group facilitated regioselective glycosylation of the 2-OH acceptor (SI Figure 12). Subsequently, the Fmoc removal and glycosylation produced the desired protected oligosaccharide intermediate, with HPLC displaying a major peak at 20 min (SI Figure 13). Following methanolysis, the presence of the semideprotected hexasaccharide was confirmed by MALDI-TOF (SI Figure 14). Photocleavage released the oligosaccharide from the resin; following hydrogenolysis and HPLC purification, 1.5 mg (10%) of α-fucan **18** was isolated. NMR analysis of **18** revealed a distinct upfield chemical shift at 5.33 ppm (d, *J* = 3.8 Hz, 1H), previously annotated for α-(1 → 2)-linkages in *Laminaria hyperborea*,^43^ therefore, the synthetic oligosaccharide supported the assigned structure.

Subsequently, two *Saccharina japonica* motifs were synthesized, one featuring a β-(1 → 4)-galactopyranoside branch, and the other presenting a more extended gal-β-(1→6)–gal-β-(1→4) branching pattern.^77^ AGA of pentasaccharide **19** relied on building blocks **1** and **2** to construct the tetrasaccharide backbone (Figure 4b). Following the removal of the 4-*O*-Lev ester, galactosylation was completed using the dibutyl phosphate donor **5** (TMSOTf, −35 °C for 5 min → −20 °C for 30 min, 5.5 equiv).^78^ HPLC analysis revealed a major peak at 32 min for the pentasaccharide (SI Figure 9). To assemble glycan **20** with the gal-β-(1 → 6)-gal-β-(1 → 4)-branch, AGA utilized building blocks **1** and **2**, alongside **4**, containing a 3-Nap ether, permitting future extension of the fucan backbone following the assembly of the galactose chain (Figure 4c). HPLC analysis of the hexasaccharide displayed a major peak at 30.52 min (SI Figure 10). Following methanolysis, photocleavage, hydrogenolysis, and HPLC purification yielded glycans **19** (0.9 mg, 8%) and **20** (0.5 mg, 4%). NMR analysis of **19** showed a β-(1 → 4)-linkage at 4.52 ppm (d, *J* = 7.3 Hz, 1H), while **20** contained an additional β-linkage at 4.32 ppm (SI Table 2).

Heptasaccharide **21** contains an α-(1 → 4)-fucopyranosyl branch found in *Laminaria hyperborea* (Figure 1a, Figure 4d).^43^ This oligosaccharide was assembled using building blocks **1** and **2**, with the Lev ester on **2** allowing the synthesis of the α-(1→4)-branch. Microcleavage analysis at the pentasaccharide stage revealed a single major peak at 22.2 min in the HPLC (SI Figure 7). Subsequently, the two Fmoc protecting groups were removed, and two coupling cycles of **1** produced the protected branched oligosaccharide (SI Figure 8). Methanolysis prior to photolytic release from the solid-phase was followed by hydrogenolysis. Reverse-phase HPLC (Hypercarb, 0 to 80 H_2_O) yielded 1.6 mg (10%) of the α-(1→4)-containing fucan **21**. NMR analysis of **21** revealed a 1,2-*cis*-linked oligosaccaride, with the anomeric carbon of the α-(1→4)-linkage occurring at 5.15 ppm (d, *J* = 4.0 Hz, 1H), downfield of the α-(1→3)-backbone linkages (SI Table 2).^43^

### Synthetic Glycan Defines the Activity of Two *endo*-Fucoidan Hydrolases

The microbial degradation of fucoidan involves hundreds of enzymes.^5,79^ Enhancing our understanding of fucoidan-active CAZymes and their substrate tolerances will advance our mechanistic understanding of how certain fucoidan structures resist degradation, leading to carbon sequestration. Furthermore, characterized enzymes can serve as biocatalytic assays to assist in the detection and quantification of fucoidan in the environment.^80,81^

CAZymes of the glycoside hydrolase family 107 (GH107) cleave in midchain glycosidic bonds of algal fucoidans.^82^ All current GH107 members are *endo*-fucoidanases targeting either α-1,3 or α-1,4 fucosyl linkages. Several α-1,4-*endo*-fucoidanases have been functionally validated, e.g., MfFcnA from *Mariniflexile fucanivorans* and Mef1 from *Allomuricauda eckloniae*, for which also protein structures were obtained.^83,84^ On the other hand, only one α-1,3-*endo*-fucoidanase has been characterized.^85^

**Figure 5:**
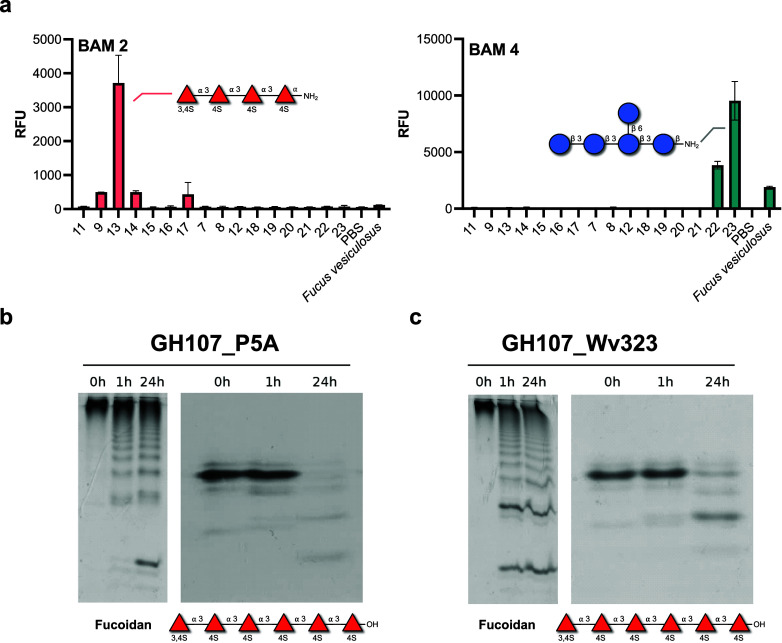
Synthetic fucoidans as tools for marine glycobiology. (a) Mapping the reactivity of BAM monoclonal antibodies with a fucoidan microarray. Shown is the 1:50 antibody dilution. (b) Left gel shows the activity of GH107_P5A on fucoidan from *L. hyperborea* by CPAGE. Right gel demonstrates the activity of GH107_P5A on synthetic α-1,3 fucan oligosaccharide **10**. (c) Left gel is the activity of GH107_P5A on fucoidan from *L. hyperborea* by CPAGE. Right gel shows the activity of GH107_Wv323 on synthetic α-1,3 fucan oligosaccharide **10**. Enzyme incubations were complete at 1 μM for 0, 1, and 24 h with each lane containing ∼4 μg of the initial substrate. The products resulting from enzymatic degradation are separated according to the size and degree of sulfation and visualized with Stains-All.

GH107_P5AFcnA from *Psychromonas* sp. SW5A displays activity against fucoidan from *Laminaria hyperborea*—a fucoidan consisting predominantly of sulfated α-1,3-linked fucan—while it is inactive on fucoidans with alternating α-1,3/α-1,4-linked fucan backbone.^43,83^ This suggests that GH107_P5AFcnA is an α-1,3-endofucoidanase but requires functional validation. Therefore, we used the synthetic α-(1→3) fucan oligosaccharide **10** to test the activity of GH107_P5AFcnA. Recombinant, purified GH107_P5AFcnA was obtained as previously described,^83^ and the enzyme was incubated with fucoidan and oligo **10**. Enzyme activity and product formation were assayed over time by CPAGE (Figure 5b). The results show that GH107_P5AFcnA degrades both fucoidan from *L. hyperborea* as well as oligo **10** and confirms that the enzyme cleaves α-1,3-linked sulfated fucan. Next, we used the protein sequence of GH107_P5AFcnA to search for homologue enzymes at National Center for Biotechnology Information (NCBI). A putative GH107 (WP_179351272) from the marine flavobacterium *Winogradskyella vidalii* showed 59% identity (>90% coverage) with GH107_P5AFcnA. Genomic studies have linked *Winogradskyella spp.* to fucoidan utilization, but so far this has not been biochemically verified.^86^ We found that pure recombinant GH107 from *W. vidalii* displayed similar activity as GH107_P5AFcnA against fucoidan and α-1,3-linked sulfated fucan oligosaccharide (Figure 5c). As such, both enzymes are α-1,3-*endo*-fucoidanases that are able to initiate the degradation of fucoidan derived from *L. hyperborea*.

According to the CPAGE results, both GH107s show activity against fucoidan after 1 h, whereas longer incubation time is needed to degrade the oligosaccharide. This suggests that the enzymes prefer substrates with longer glycan chains or different sulfation patterns than **10**—structures that seem to occur in the native fucoidan. For example, fucoidan from *L. hyperborea* can be C2 sulfated in addition to C4.^43^ Nevertheless, we demonstrate here the α-(1→3)-fucan specificity of two GH107 fucoidanases derived from marine bacteria. These results demonstrate the utility of synthetic oligosaccharides in the discovery and characterization of fucoidan-degrading enzymes.

### Glycan Microarrays Map Specificity of Fucoidan-Directed Antibodies

Understanding the relationship between the structure of fucoidans and their functional properties is challenging due to the heterogeneity of polysaccharides extracted from algae. Therefore, we constructed a glycan microarray to investigate the binding of protein to our synthetic fucoidan library. Amine-functionalized fucoidan oligosaccharides along with two control β-(1→3)-glucans, **22** and **23**, were covalently attached to *N*-hydroxylsuccinimide (NHS)-functionalized glass slides. Each glycan was printed in quadruplicate at a concentration of 100 μM using a robotic printer.

The binding specificity of four monoclonal antibodies (mAbs) targeting fucoidan, BAM1–BAM4, was investigated. These antibodies allow the visualization of algae and diatom cell walls.^3,4,12^ Furthermore, they aid in the environmental detection and quantification of fucoidan in seawater and sediments.^3,4,7^ A limitation of these antibodies is that their epitopes are not precisely defined.^12^

Microarray analysis of BAM1 and BAM3 revealed no binding to any fucoidan oligosaccharides on the array, suggesting interactions with structural epitopes not present in the current library (Figure 5a). mAb BAM4 demonstrated no binding to any fucoidan structures on the array but did display reproducible binding to a β-(1→3)-glucan tetrasaccharide **22** and **23** β-(1→3)-glucan tetrasaccharide with a central β-(1→6)-branch (Figure 5a; SI Figure 15).

In the case of mAb BAM2, binding was observed to sulfated fucoidan oligosaccharides **11**, **9**, **13**, **14**, and **17** (Figure 5a). Low level binding to glycan **11**, a monosaccharide with a di-3,4-*O*-sulfation pattern, was only observed at a higher concentration of mAb BAM2, suggesting that this di-*O*-sulfated monosaccharide poorly represents the BAM2 epitope. While binding to α-(1→3)-fucoidan oligosaccharides (**9**, **13**, and **14**) with 4-*O*-sulfate esters and a terminal di-3,4-*O*-sulfation pattern provided more robust binding across a range of concentrations (SI Figure 15). mAb BAM2 also bound weakly to sulfated fucoidan oligosaccharide **17**, with an α-(1→3)−α-(1→4)-linked backbone. Collectively, this suggests that these 4-*O*-sulfated oligosaccharides contain the BAM2 epitope.^14,86^

### Binding of BAM2 to *Thalassiosira weissflogii* Suggests the Diatom Synthesizes a Fucoidan Structurally Similar to Those in Brown Algae

The formation of sinking particles in the ocean promotes carbon sequestration, and microalgal polysaccharides are involved in this process. Recent findings, utilizing the fucoidan-specific monoclonal antibodies BAM1 and BAM2, have revealed that diatoms *Chaetoceros spp*. and *Thalassiosira weissflogii* produce fucose-containing sulfated polysaccharides (FCSP). These polysaccharides form particles that promote aggregation, sinking, and, consequently, carbon sequestration.^4,87^ FCSP is a broad term used to imply the presence of fucoidan-like structures but does not refer to a particular structure.

**Figure 6:**
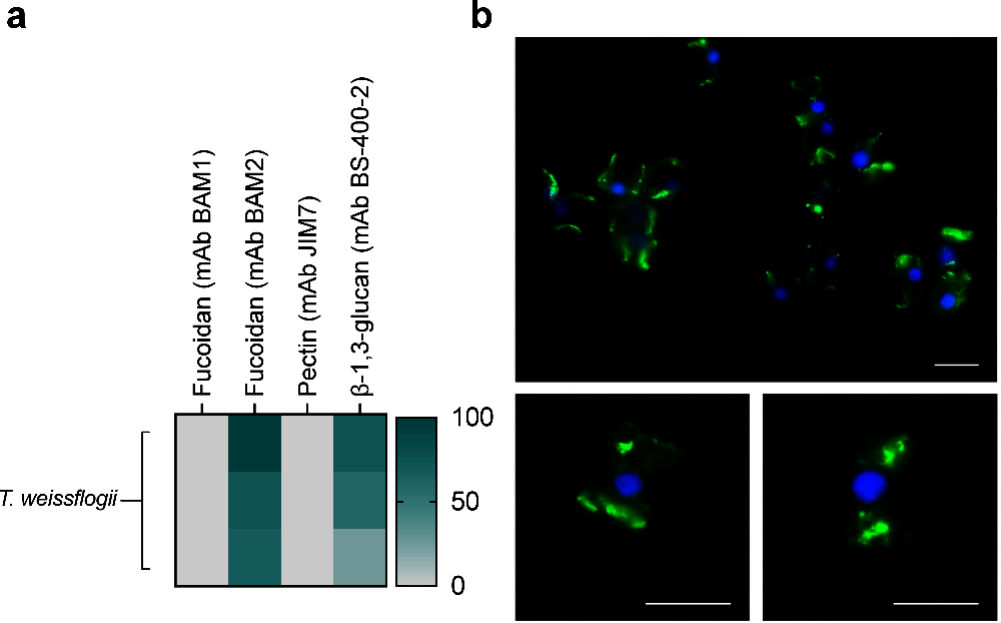
Binding of mAb BAM2 detects that *Thalassiosira weissflogii* synthesizes fucoidan. (a) *T. weissflogii* water extracts were printed onto microarrays, and those were probed with a panel of monoclonal antibodies (mAbs), each row represents a replicate. In addition to the antifucoidan BAM1 and BAM2, JIM7 (antipectin) and BS-400-2 (anti-β-1,3-glucan) were included as negative and positive controls, respectively. The heatmap shows normalized mAb binding intensity in the extraction triplicates. (b) Representative images showing that mAb BAM2 binds to *T. weissflogii* cells. Fluorescence microscopy images demonstrate that the BAM2 epitope is present on the diatom cell surfaces. α-1,3-Linked fucoidan (green), DAPI (blue). Scale bars: 10 μm. Experiments were performed three times with similar results.

Analysis of polysaccharide extracts from the diatom *Thalassiosira weissflogii* using microarrays suggested the presence of distinct fucoidan that was reactive to BAM2 but not BAM1 (Figure 6a),^87^ implying that diatom species synthesize diverse fucoidan structures. In this study, we mapped the specificity of the mAb BAM2, and hypothesized that this means *T. weissflogii* produces a fucoidan with either α-(1→3)-linked fucose or alternating α-(1→3)−α-(1→4)-linked fucose and 4-O-sulfation. Microscopy analysis of diatom cells post-roller tank experiments, which were employed to induce aggregation, revealed the presence of the BAM2 fucoidan epitope surrounding the diatom cell aggregates.^87^ Furthermore, here we demonstrate that individual diatom cells produce this fucoidan epitope and present it on their cell surfaces (Figure 6b).

The presence of structures known for their carbon sequestration capacity, within a globally distributed diatom suggests that the synthesis of molecules is more common than previously assumed. Detailed analysis of the glycans present in marine ecosystems will allow for a deeper understanding of the marine carbon cycle. In this process, structurally defined synthetic oligosaccharides served as a missing link in various existing tools, including enzymatic, immunological, and spectroscopic methods.

## Conclusions

Automated glycan assembly provides access to fucoidan oligosaccharides, reaching lengths of up to 20-mers, with diverse branching patterns, and up to 11 sulfate esters. Modulating the reactivity of building blocks, by altering the thioglycoside aglycon from an alkyl to a less reactive aryl group, enabled the synthesis of the oligosaccharides. NMR experiments confirmed that these synthetic fucoidan oligosaccharides contain structural features that are found in brown algae. A synthetic oligosaccharide also enabled the characterization of two GH107 *endo*-fucoidanases from marine bacteria, with both enzymes capable of degrading α-(1→3)-linked fucoidan sulfated oligosaccharides. A fucoidan microarray was used to map the specificity of four monoclonal antibodies (mAbs) directed toward fucoidan. The mAb BAM4 was discovered to have cross-reactivity toward β-(1→3)-glucans, both with and without β-(1→6)-glucose branches. The mAb BAM2 bound to fucoidan oligosaccharides with α-(1→3)- and α-(1→3)−α-(1→4)-linkages bearing 4-*O*-sulfate esters. This molecular understanding of mAb BAM2 specificity provides evidence that *Thalassiosira weissflogii*, a globally abundant diatom, synthesizes fucoidan structurally similar to that found in brown algae. Synthetic fucoidan oligosaccharides are tools to investigate fucoidan’s roles in carbon sequestration and medicine.

## Supporting Information Available

Supporting figures, tables, experimental procedures, and spectral data for all reactions and compounds (PDF)

## References

[ref1] Field C. B.; Behrenfeld M. J.; Randerson J. T.; Falkowski P. Primary Production of the Biosphere: Integrating Terrestrial and Oceanic Components. Science (1979) 1998, 281 (5374), 237–240. 10.1126/science.281.5374.237. 9657713

[ref2] Bligh M.; Nguyen N.; Buck-Wiese H.; Vidal-Melgosa S.; Hehemann J.-H. Structures and Functions of Algal Glycans Shape Their Capacity to Sequester Carbon in the Ocean. Curr. Opin Chem. Biol. 2022, 71, 102204 10.1016/j.cbpa.2022.102204. 36155346

[ref3] Buck-Wiese H.; Andskog M. A.; Nguyen N. P.; Bligh M.; Asmala E.; Vidal-Melgosa S.; Liebeke M.; Gustafsson C.; Hehemann J. H. Fucoid Brown Algae Inject Fucoidan Carbon into the Ocean. Proc. Natl. Acad. Sci. U. S. A. 2023, 120 (1), e2210561119 10.1073/pnas.2210561119. 36584294 PMC9910443

[ref4] Vidal-Melgosa S.; Sichert A.; Francis T. B.; Bartosik D.; Niggemann J.; Wichels A.; Willats W. G. T.; Fuchs B. M.; Teeling H.; Becher D.; Schweder T.; Amann R.; Hehemann J.-H. Diatom Fucan Polysaccharide Precipitates Carbon during Algal Blooms. Nat. Commun. 2021, 12 (1), 1150 10.1038/s41467-021-21009-6. 33608542 PMC7896085

[ref5] Sichert A.; Corzett C. H.; Schechter M. S.; Unfried F.; Markert S.; Becher D.; Fernandez-Guerra A.; Liebeke M.; Schweder T.; Polz M. F.; Hehemann J. H. Verrucomicrobia Use Hundreds of Enzymes to Digest the Algal Polysaccharide Fucoidan. Nature Microbiology 2020 5:8 2020, 5 (8), 1026–1039. 10.1038/s41564-020-0720-2. 32451471

[ref6] Reintjes G.; Heins A.; Wang C.; Amann R. Abundance and Composition of Particles and Their Attached Microbiomes along an Atlantic Meridional Transect. Front Mar Sci. 2023, 10, 1051510 10.3389/fmars.2023.1051510.

[ref7] Vidal-Melgosa S.; Lagator M.; Sichert A.; Priest T.; Pätzold J.; Hehemann J.-H. Not Digested: Algal Glycans Move Carbon Dioxide into the Deep-Sea. bioRxiv 2022, 2022.03.04.483023 10.1101/2022.03.04.483023.

[ref8] Salmeán A. A.; Willats W. G. T.; Ribeiro S.; Andersen T. J.; Ellegaard M. Over 100-Year Preservation and Temporal Fluctuations of Cell Wall Polysaccharides in Marine Sediments. Front Plant Sci. 2022, 13, 785902 10.3389/fpls.2022.785902. 35519816 PMC9062592

[ref9] Priyan Shanura Fernando I.; Kim K. N.; Kim D.; Jeon Y. J. Algal Polysaccharides: Potential Bioactive Substances for Cosmeceutical Applications. Crit Rev. Biotechnol 2019, 39 (1), 99–113. 10.1080/07388551.2018.1503995. 30198346

[ref10] Fitton J. H.; Stringer D. N.; Karpiniec S. S. Therapies from Fucoidan: An Update. Mar Drugs 2015, 13 (9), 5920–5946. 10.3390/md13095920. 26389927 PMC4584361

[ref11] Crawford C. J.; Seeberger P. H. Advances in Glycoside and Oligosaccharide Synthesis. Chem. Soc. Rev. 2023, 52 (22), 7773–7801. 10.1039/D3CS00321C. 37830906

[ref12] Torode T. A.; Marcus S. E.; Jam M.; Tonon T.; Blackburn R. S.; Hervé C.; Knox J. P. Monoclonal Antibodies Directed to Fucoidan Preparations from Brown Algae. PLoS One 2015, 10 (2), e0118366 10.1371/journal.pone.0118366. 25692870 PMC4333822

[ref13] Geissner A.; Reinhardt A.; Rademacher C.; Johannssen T.; Monteiro J.; Lepenies B.; Thépaut M.; Fieschi F.; Mrázková J.; Wimmerova M.; Schuhmacher F.; Götze S.; Grünstein D.; Guo X.; Hahm H. S.; Kandasamy J.; Leonori D.; Martin C. E.; Parameswarappa S. G.; Pasari S.; Schlegel M. K.; Tanaka H.; Xiao G.; Yang Y.; Pereira C. L.; Anish C.; Seeberger P. H. Microbe-Focused Glycan Array Screening Platform. Proc. Natl. Acad. Sci. U. S. A. 2019, 116 (6), 1958–1967. 10.1073/pnas.1800853116. 30670663 PMC6369816

[ref14] Crawford C. J.; Guazzelli L.; McConnell S. A.; McCabe O.; d’Errico C.; Greengo S. D.; Wear M. P.; Jedlicka A. E.; Casadevall A.; Oscarson S. Synthetic Glycans Reveal Determinants of Antibody Functional Efficacy against a Fungal Pathogen. ACS Infect Dis 2023, 10 (2), 475–488. 10.1021/acsinfecdis.3c00447. 37856427 PMC10862557

[ref15] Fukui S.; Feizi T.; Galustian C.; Lawson A. M.; Chai W. Oligosaccharide Microarrays for High-Throughput Detection and Specificity Assignments of Carbohydrate-Protein Interactions. Nature Biotechnology 2002 20:10 2002, 20 (10), 1011–1017. 10.1038/nbt735. 12219077

[ref16] Palma A. S.; Feizi T.; Zhang Y.; Stoll M. S.; Lawson A. M.; Díaz-Rodríguez E.; Campanero-Rhodes M. A.; Costa J.; Gordon S.; Brown G. D.; Chai W. Ligands for the β-Glucan Receptor, Dectin-1, Assigned Using “Designer” Microarrays of Oligosaccharide Probes (Neoglycolipids) Generated from Glucan Polysaccharides. J. Biol. Chem. 2006, 281 (9), 5771–5779. 10.1074/jbc.M511461200. 16371356

[ref17] Becker S.; Tebben J.; Coffinet S.; Wiltshire K.; Iversen M. H.; Harder T.; Hinrichs K. U.; Hehemann J. H. Laminarin Is a Major Molecule in the Marine Carbon Cycle. Proc. Natl. Acad. Sci. U. S. A. 2020, 117 (12), 6599–6607. 10.1073/pnas.1917001117. 32170018 PMC7104365

[ref18] Solanki V.; Krüger K.; Crawford C. J.; Pardo-Vargas A.; Danglad-Flores J.; Hoang K. L. M.; Klassen L.; Abbott D. W.; Seeberger P. H.; Amann R. I.; Teeling H.; Hehemann J.-H. Glycoside Hydrolase from the GH76 Family Indicates That Marine Salegentibacter Sp. Hel_I_6 Consumes Alpha-Mannan from Fungi. ISME J. 2022, 16 (7), 1818–1830. 10.1038/s41396-022-01223-w. 35414716 PMC9213526

[ref19] Kelly S. D.; Williams D. M.; Nothof J. T.; Kim T.; Lowary T. L.; Kimber M. S.; Whitfield C. The Biosynthetic Origin of Ribofuranose in Bacterial Polysaccharides. Nature Chemical Biology 2022 18:5 2022, 18 (5), 530–537. 10.1038/s41589-022-01006-6. 35393575

[ref20] Silchenko A. S.; Ustyuzhanina N. E.; Kusaykin M. I.; Krylov V. B.; Shashkov A. S.; Dmitrenok A. S.; Usoltseva R. V.; Zueva A. O.; Nifantiev N. E.; Zvyagintseva T. N. Expression and Biochemical Characterization and Substrate Specificity of the Fucoidanase from Formosa Algae. Glycobiology 2016, 27 (3), 254–263. 10.1093/glycob/cww138. 28031251

[ref21] Delbianco M.; Kononov A.; Poveda A.; Yu Y.; Diercks T.; Jiménez-Barbero J.; Seeberger P. H. Well-Defined Oligo- and Polysaccharides as Ideal Probes for Structural Studies. J. Am. Chem. Soc. 2018, 140 (16), 5421–5426. 10.1021/jacs.8b00254. 29624385

[ref22] Canales A.; Mallagaray A.; Pérez-Castells J.; Boos I.; Unverzagt C.; André S.; Gabius H. J.; Cañada F. J.; Jiménez-Barbero J. Breaking Pseudo-Symmetry in Multiantennary Complex N-Glycans Using Lanthanide-Binding Tags and NMR Pseudo-Contact Shifts. Angew. Chem., Int. Ed. 2013, 52 (51), 13789–13793. 10.1002/anie.201307845. 24346952

[ref23] Hargett A. A.; Azurmendi H. F.; Crawford C. J.; Wear M. P.; Oscarson S.; Casadevall A.; Freedberg D. I. The Structure of a C. Neoformans Polysaccharide Motif Recognized by Protective Antibodies: A Combined NMR and MD Study. Proc. Natl. Acad. Sci. U. S. A. 2024, 121 (7), e2315733121 10.1073/pnas.2315733121. 38330012 PMC10873606

[ref24] Nestor G.; Anderson T.; Oscarson S.; Gronenborn A. M. Exploiting Uniformly 13C-Labeled Carbohydrates for Probing Carbohydrate-Protein Interactions by NMR Spectroscopy. J. Am. Chem. Soc. 2017, 139 (17), 6210–6216. 10.1021/jacs.7b01929. 28406013 PMC5725960

[ref25] Hu Z.; Silipo A.; Li W.; Molinaro A.; Yu B. Synthesis of Forsythenethoside A, a Neuroprotective Macrocyclic Phenylethanoid Glycoside, and NMR Analysis of Conformers. J. Org. Chem. 2019, 84 (21), 13733–13743. 10.1021/acs.joc.9b01956. 31552736

[ref26] Schmidt R. R.; Toepfer A. Glycosylation with Highly Reactive Glycosyl Donors: Efficiency of the Inverse Procedure. Tetrahedron Lett. 1991, 32 (28), 3353–3356. 10.1016/S0040-4039(00)92704-7.

[ref27] Joseph A. A.; Pardo-Vargas A.; Seeberger P. H. Total Synthesis of Polysaccharides by Automated Glycan Assembly. J. Am. Chem. Soc. 2020, 142 (19), 8561–8564. 10.1021/jacs.0c00751. 32338884 PMC7304863

[ref28] Zhu Y.; Delbianco M.; Seeberger P. H. Automated Assembly of Starch and Glycogen Polysaccharides. J. Am. Chem. Soc. 2021, 143 (26), 9758–9768. 10.1021/jacs.1c02188. 34115468 PMC8267850

[ref29] Wang L.; Overkleeft H. S.; Van Der Marel G. A.; Codée J. D. C. Reagent Controlled Stereoselective Synthesis of α-Glucans. J. Am. Chem. Soc. 2018, 140 (13), 4632–4638. 10.1021/jacs.8b00669. 29553729 PMC5890317

[ref30] Lu S. R.; Lai Y. H.; Chen J. H.; Liu C. Y.; Mong K. K. T. Dimethylformamide: An Unusual Glycosylation Modulator. Angew. Chem., Int. Ed. 2011, 50 (32), 7315–7320. 10.1002/anie.201100076. 21688356

[ref31] Lemieux R. U.; Hendriks K. B.; Stick R. V.; James K. Halide Ion Catalyzed Glycosidation Reactions. Syntheses of α-Linked Disaccharides. J. Am. Chem. Soc. 1975, 97 (14), 4056–4062. 10.1021/ja00847a032.

[ref32] Oscarson S.; Sehgelmeble F. W. A Novel β-Directing Fructofuranosyl Donor Concept. Stereospecific Synthesis of Sucrose. J. Am. Chem. Soc. 2000, 122 (37), 8869–8872. 10.1021/ja001439u.

[ref33] Crich D.; Sun S. Formation of Beta-Mannopyranosides of Primary Alcohols Using the Sulfoxide Method. J. Org. Chem. 1996, 61 (14), 4506–4507. 10.1021/jo9606517. 11667369

[ref34] Miller G. J.; Hansen S. U.; Avizienyte E.; Rushton G.; Cole C.; Jayson G. C.; Gardiner J. M. Efficient Chemical Synthesis of Heparin-like Octa-, Deca- and Dodecasaccharides and Inhibition of FGF2- and VEGF165-Mediated Endothelial Cell Functions. Chem. Sci. 2013, 4 (8), 3218–3222. 10.1039/c3sc51217g.

[ref35] Wu Y.; Bosman G. P.; Chapla D.; Huang C.; Moremen K. W.; de Vries R. P.; Boons G.-J. A Biomimetic Synthetic Strategy Can. Provide Keratan Sulfate I and II Oligosaccharides with Diverse Fucosylation and Sulfation Patterns. J. Am. Chem. Soc. 2024, 146, 9230 10.1021/jacs.4c00363. 38494637 PMC10996015

[ref36] Wang L.; Sorum A. W.; Huang B. S.; Kern M. K.; Su G.; Pawar N.; Huang X.; Liu J.; Pohl N. L. B.; Hsieh-Wilson L. C. Efficient Platform for Synthesizing Comprehensive Heparan Sulfate Oligosaccharide Libraries for Decoding Glycosaminoglycan-Protein Interactions. Nature Chemistry 2023 15:8 2023, 15 (8), 1108–1117. 10.1038/s41557-023-01248-4. PMC10979459 37349377

[ref37] Vinnitskiy D. Z.; Krylov V. B.; Ustyuzhanina N. E.; Dmitrenok A. S.; Nifantiev N. E. The Synthesis of Heterosaccharides Related to the Fucoidan from Chordaria Flagelliformis Bearing an α-L-Fucofuranosyl Unit. Org. Biomol Chem. 2016, 14 (2), 598–611. 10.1039/C5OB02040A. 26536063

[ref38] Tuck O. T.; Sletten E. T.; Danglad-Flores J.; Seeberger P. H. Towards a Systematic Understanding of the Influence of Temperature on Glycosylation Reactions. Angew. Chem., Int. Ed. 2022, 61, e202115433 10.1002/anie.202115433. PMC9306470 35032966

[ref39] Zhang Z.; Ollmann I. R.; Ye X. S.; Wischnat R.; Baasov T.; Wong C. H. Programmable One-Pot Oligosaccharide Synthesis. J. Am. Chem. Soc. 1999, 121 (4), 734–753. 10.1021/ja982232s.

[ref40] Daly R.; McCabe T.; Scanlan E. M. Development of Fully and Partially Protected Fucosyl Donors for Oligosaccharide Synthesis. J. Org. Chem. 2013, 78 (3), 1080–1090. 10.1021/jo302487c. 23268556

[ref41] Lim S. J.; Wan Aida W. M.; Schiehser S.; Rosenau T.; Böhmdorfer S. Structural Elucidation of Fucoidan from Cladosiphon Okamuranus (Okinawa Mozuku). Food Chem. 2019, 272, 222–226. 10.1016/j.foodchem.2018.08.034. 30309536

[ref42] Nagaoka M.; Shibata H.; Kimura-Takagi I.; Hashimoto S.; Kimura K.; Makino T.; Aiyama R.; Ueyama S.; Yokokura T. Structural Study of Fucoidan from Cladosiphon Okamuranus. Glycoconj J. 1999, 16 (1), 19–26. 10.1023/A:1006945618657. 10580647

[ref43] Kopplin G.; Rokstad A. M.; Mélida H.; Bulone V.; Skjåk-Bræk G.; Aachmann F. L. Structural Characterization of Fucoidan from Laminaria Hyperborea: Assessment of Coagulation and Inflammatory Properties and Their Structure-Function Relationship. ACS Appl. Bio Mater. 2018, 1 (6), 1880–1892. 10.1021/acsabm.8b00436. 34996289

[ref44] Bilan M. I.; Grachev A. A.; Ustuzhanina N. E.; Shashkov A. S.; Nifantiev N. E.; Usov A. I. Structure of a Fucoidan from the Brown Seaweed Fucus Evanescens C.Ag. Carbohydr. Res. 2002, 337 (8), 719–730. 10.1016/S0008-6215(02)00053-8. 11950468

[ref45] Jin W.; Zhang W.; Mitra D.; McCandless M. G.; Sharma P.; Tandon R.; Zhang F.; Linhardt R. J. The Structure-Activity Relationship of the Interactions of SARS-CoV-2 Spike Glycoproteins with Glucuronomannan and Sulfated Galactofucan from Saccharina Japonica. Int. J. Biol. Macromol. 2020, 163, 1649–1658. 10.1016/j.ijbiomac.2020.09.184. 32979436 PMC7513770

[ref46] Koh H. S. A.; Lu J.; Zhou W. Structure Characterization and Antioxidant Activity of Fucoidan Isolated from Undaria Pinnatifida Grown in New Zealand. Carbohydr. Polym. 2019, 212, 178–185. 10.1016/j.carbpol.2019.02.040. 30832845

[ref47] Deniaud-Bouët E.; Hardouin K.; Potin P.; Kloareg B.; Hervé C. A Review about Brown Algal Cell Walls and Fucose-Containing Sulfated Polysaccharides: Cell Wall Context, Biomedical Properties and Key Research Challenges. Carbohydr. Polym. 2017, 175, 395–408. 10.1016/j.carbpol.2017.07.082. 28917882

[ref48] Patankar M. S.; Oehninger S.; Barnett T.; Williams R. L.; Clark G. F. A Revised Structure for Fucoidan May Explain Some of Its Biological Activities. J. Biol. Chem. 1993, 268 (29), 21770–21776. 10.1016/S0021-9258(20)80609-7. 8408031

[ref49] Behera A.; Rai D.; Kulkarni S. S. Total Syntheses of Conjugation-Ready Trisaccharide Repeating Units of Pseudomonas Aeruginosa O11 and Staphylococcus Aureus Type 5 Capsular Polysaccharide for Vaccine Development. J. Am. Chem. Soc. 2020, 142 (1), 456–467. 10.1021/jacs.9b11309. 31815459

[ref50] Kasai A.; Arafuka S.; Koshiba N.; Takahashi D.; Toshima K. Systematic Synthesis of Low-Molecular Weight Fucoidan Derivatives and Their Effect on Cancer Cells. Org. Biomol Chem. 2015, 13 (42), 10556–10568. 10.1039/C5OB01634G. 26340595

[ref51] Romero J. A. C.; Tabacco S. A.; Woerpel K. A. Stereochemical Reversal of Nucleophilic Substitution Reactions Depending upon Substituent: Reactions of Heteroatom-Substituted Six-Membered-Ring Oxocarbenium Ions through Pseudoaxial Conformers. J. Am. Chem. Soc. 2000, 122 (1), 168–169. 10.1021/ja993366o. 14664599

[ref52] Ye X. S.; Wong C. H. Anomeric Reactivity-Based One-Pot Oligosaccharide Synthesis: A Rapid Route to Oligosaccharide Libraries. J. Org. Chem. 2000, 65 (8), 2410–2431. 10.1021/jo991558w. 10789453

[ref53] Danglad-Flores J.; Leichnitz S.; Sletten E. T.; Abragam Joseph A.; Bienert K.; Le Mai Hoang K.; Seeberger P. H. Microwave-Assisted Automated Glycan Assembly. J. Am. Chem. Soc. 2021, 143 (23), 8893–8901. 10.1021/jacs.1c03851. 34060822 PMC8213053

[ref54] Lahmann M.; Oscarson S. Investigation of the Reactivity Difference between Thioglycoside Donors with Variant Aglycon Parts. Can. J. Chem. 2002, 80 (8), 889–893. 10.1139/v02-101.

[ref55] Le Mai Hoang K.; Pardo-Vargas A.; Zhu Y.; Yu Y.; Loria M.; Delbianco M.; Seeberger P. H. Traceless Photolabile Linker Expedites the Chemical Synthesis of Complex Oligosaccharides by Automated Glycan Assembly. J. Am. Chem. Soc. 2019, 141 (22), 9079–9086. 10.1021/jacs.9b03769. 31091089 PMC6750752

[ref56] Calabro A.; Midura R.; Wang A.; West L.; Plaas A.; Hascall V. C. Fluorophore-Assisted Carbohydrate Electrophoresis (FACE) of Glycosaminoglycans. Osteoarthritis Cartilage 2001, 9, S16–S22. 10.1053/joca.2001.0439. 11680680

[ref57] Yu Y.; Kononov A.; Delbianco M.; Seeberger P. H. A Capping Step During Automated Glycan Assembly Enables Access to Complex Glycans in High Yield. Chem.—Eur. J. 2018, 24 (23), 6075–6078. 10.1002/chem.201801023. 29498436

[ref58] Tyrikos-Ergas T.; Sletten E. T.; Huang J. Y.; Seeberger P. H.; Delbianco M. On Resin Synthesis of Sulfated Oligosaccharides. Chem. Sci. 2022, 13 (7), 2115–2120. 10.1039/D1SC06063E. 35308866 PMC8848854

[ref59] Tyrikos-Ergas T.; Bordoni V.; Fittolani G.; Chaube M. A.; Grafmüller A.; Seeberger P. H.; Delbianco M. Systematic Structural Characterization of Chitooligosaccharides Enabled by Automated Glycan Assembly. Chem.—Eur. J. 2021, 27 (7), 2321–2325. 10.1002/chem.202005228. 33290603 PMC7898498

[ref60] Eller S.; Collot M.; Yin J.; Hahm H. S.; Seeberger P. H. Automated Solid-Phase Synthesis of Chondroitin Sulfate Glycosaminoglycans. Angew. Chem., Int. Ed. 2013, 52 (22), 5858–5861. 10.1002/anie.201210132. 23589381

[ref61] Crawford C. J.; Qiao Y.; Liu Y.; Huang D.; Yan W.; Seeberger P. H.; Oscarson S.; Chen S. Defining the Qualities of High-Quality Palladium on Carbon Catalysts for Hydrogenolysis. Org. Process Res. Dev 2021, 25 (7), 1573–1578. 10.1021/acs.oprd.0c00536. 34305386 PMC8291771

[ref62] Crawford C.; Oscarson S. Optimized Conditions for the Palladium-Catalyzed Hydrogenolysis of Benzyl and Naphthylmethyl Ethers: Preventing Saturation of Aromatic Protecting Groups. Eur. J. Org. Chem. 2020, 2020, 3332–3337. 10.1002/ejoc.202000401.

[ref63] Schumann B.; Parameswarappa S. G.; Lisboa M. P.; Kottari N.; Guidetti F.; Pereira C. L.; Seeberger P. H. Nucleophile-Directed Stereocontrol Over Glycosylations Using Geminal-Difluorinated Nucleophiles. Angew. Chem., Int. Ed. 2016, 55 (46), 14431–14434. 10.1002/anie.201606774. 27735117

[ref64] Chopra P.; Joshi A.; Wu J.; Lu W.; Yadavalli T.; Wolfert M. A.; Shukla D.; Zaia J.; Boons G. J. The 3-O-Sulfation of Heparan Sulfate Modulates Protein Binding and Lyase Degradation. Proc. Natl. Acad. Sci. U. S. A. 2021, 118 (3), e2012935118 10.1073/pnas.2012935118. 33441484 PMC7826381

[ref65] Sankaranarayanan N. V.; Strebel T. R.; Boothello R. S.; Sheerin K.; Raghuraman A.; Sallas F.; Mosier P. D.; Watermeyer N. D.; Oscarson S.; Desai U. R. A Hexasaccharide Containing Rare 2-O-Sulfate-Glucuronic Acid Residues Selectively Activates Heparin Cofactor II. Angew. Chem., Int. Ed. 2017, 56 (9), 2312–2317. 10.1002/anie.201609541. PMC5347859 28124818

[ref66] de Paz J. L.; Moseman E. A.; Noti C.; Polito L.; von Andrian U. H.; Seeberger P. H. Profiling Heparin - Chemokine Interactions Using Synthetic Tools. ACS Chem. Biol. 2007, 2 (11), 735–744. 10.1021/cb700159m. 18030990 PMC2716178

[ref67] Jayson G. C.; Hansen S. U.; Miller G. J.; Cole C. L.; Rushton G.; Avizienyte E.; Gardiner J. M. Synthetic Heparan Sulfate Dodecasaccharides Reveal Single Sulfation Site Interconverts CXCL8 and CXCL12 Chemokine Biology. Chem. Commun. 2015, 51 (72), 13846–13849. 10.1039/C5CC05222J. PMC4608306 26234943

[ref68] Schwörer R.; Zubkova O. V.; Turnbull J. E.; Tyler P. C. Synthesis of a Targeted Library of Heparan Sulfate Hexa- to Dodecasaccharides as Inhibitors of β-Secretase: Potential Therapeutics for Alzheimer’s Disease. Chem.—Eur. J. 2013, 19 (21), 6817–6823. 10.1002/chem.201204519. 23553710

[ref69] Hahm H. S.; Broecker F.; Kawasaki F.; Mietzsch M.; Heilbronn R.; Fukuda M.; Seeberger P. H. Automated Glycan Assembly of Oligo-N-Acetyllactosamine and Keratan Sulfate Probes to Study Virus-Glycan Interactions. Chem. 2017, 2 (1), 114–124. 10.1016/j.chempr.2016.12.004.

[ref70] Pongener I.; Sletten E. T.; Danglad-Flores J.; Seeberger P. H.; Miller G. J. Synthesis of a Heparan Sulfate Tetrasaccharide Using Automated Glycan Assembly. Org. Biomol Chem. 2024, 22 (7), 1395–1399. 10.1039/D3OB01909H. 38291974 PMC10865181

[ref71] Chhabra M.; Wimmer N.; He Q. Q.; Ferro V. Development of Improved Synthetic Routes to Pixatimod (PG545), a Sulfated Oligosaccharide-Steroid Conjugate. Bioconjug Chem. 2021, 32 (11), 2420–2431. 10.1021/acs.bioconjchem.1c00453. 34652896

[ref72] Chen L.; Lee S.; Renner M.; Tian Q.; Nayyar N. A Simple Modification to Prevent Side Reactions in Swern-Type Oxidations Using Py·SO3. Org. Process Res. Dev 2006, 10 (1), 163–164. 10.1021/op0502203.

[ref73] Santini R.; Griffith M. C.; Qi M. A Measure of Solvent Effects on Swelling of Resins for Solid Phase Organic Synthesis. Tetrahedron Lett. 1998, 39 (49), 8951–8954. 10.1016/S0040-4039(98)02069-3.

[ref74] Sarin V. K.; Kent S. B. H.; Merrifield R. B. Properties of Swollen Polymer Networks. Solvation and Swelling of Peptide-Containing Resins in Solid-Phase Peptide Synthesis. J. Am. Chem. Soc. 1980, 102 (17), 5463–5470. 10.1021/ja00537a006.

[ref75] Bakhatan Y.; Alshanski I.; Grunhaus D.; Hurevich M. The Breaking Beads Approach for Photocleavage from Solid Support. Org. Biomol Chem. 2020, 18 (22), 4183–4188. 10.1039/D0OB00821D. 32441723

[ref76] Teschers C. S.; Gilmour R. Flow Photocleavage for Automated Glycan Assembly (AGA). Org. Process Res. Dev 2020, 24 (10), 2234–2239. 10.1021/acs.oprd.0c00286.

[ref77] Jin W.; Lu C.; Zhu Y.; Zhao J.; Zhang W.; Wang L.; Linhardt R. J.; Wang C.; Zhang F. Fucoidans Inhibited Tau Interaction and Cellular Uptake. Carbohydr. Polym. 2023, 299, 120176 10.1016/j.carbpol.2022.120176. 36876791 PMC10506861

[ref78] Bartetzko M. P.; Schuhmacher F.; Hahm H. S.; Seeberger P. H.; Pfrengle F. Automated Glycan Assembly of Oligosaccharides Related to Arabinogalactan Proteins. Org. Lett. 2015, 17 (17), 4344–4347. 10.1021/acs.orglett.5b02185. 26295743

[ref79] Orellana L. H.; Francis T. B.; Ferraro M.; Hehemann J. H.; Fuchs B. M.; Amann R. I. Verrucomicrobiota Are Specialist Consumers of Sulfated Methyl Pentoses during Diatom Blooms. ISME J. 2022, 16 (3), 630–641. 10.1038/s41396-021-01105-7. 34493810 PMC8857213

[ref80] Becker S.; Scheffel A.; Polz M. F.; Hehemann J. H. Accurate Quantification of Laminarin in Marine Organic Matter with Enzymes from Marine Microbes. Appl. Environ. Microbiol. 2017, 83 (9), e03389-16 10.1128/AEM.03389-16. 28213541 PMC5394322

[ref81] Steinke N.; Vidal-Melgosa S.; Schultz-Johansen M.; Hehemann J. Biocatalytic Quantification of Α-glucan in Marine Particulate Organic Matter. Microbiologyopen 2022, 11 (3), e1289 10.1002/mbo3.1289. 35765187 PMC9134812

[ref82] Drula E.; Garron M. L.; Dogan S.; Lombard V.; Henrissat B.; Terrapon N. The Carbohydrate-Active Enzyme Database: Functions and Literature. Nucleic Acids Res. 2022, 50 (D1), D571–D577. 10.1093/nar/gkab1045. 34850161 PMC8728194

[ref83] Vickers C.; Liu F.; Abe K.; Salama-Alber O.; Jenkins M.; Springate C. M. K.; Burke J. E.; Withers S. G.; Boraston A. B. Endo-Fucoidan Hydrolases from Glycoside Hydrolase Family 107 (GH107) Display Structural and Mechanistic Similarities to α-l-Fucosidases from GH29. J. Biol. Chem. 2018, 293 (47), 18296 10.1074/jbc.RA118.005134. 30282808 PMC6254363

[ref84] Mikkelsen M. D.; Tran V. H. N.; Meier S.; Nguyen T. T.; Holck J.; Cao H. T. T.; Van T. T. T.; Thinh P. D.; Meyer A. S.; Morth J. P. Structural and Functional Characterization of the Novel Endo-α(1,4)-Fucoidanase Mef1 from the Marine Bacterium Muricauda Eckloniae. Acta Crystallogr. D Struct Biol. 2023, 79 (11), 1026–1043. 10.1107/S2059798323008732. 37877949 PMC10619423

[ref85] Tran V. H. N.; Nguyen T. T.; Meier S.; Holck J.; Cao H. T. T.; Van T. T. T.; Meyer A. S.; Mikkelsen M. D. The Endo-α(1,3)-Fucoidanase Mef2 Releases Uniquely Branched Oligosaccharides from Saccharina Latissima Fucoidans. Mar Drugs 2022, 20 (5), 305 10.3390/md20050305. 35621956 PMC9147238

[ref86] Alejandre-Colomo C.; Francis B.; Viver T.; Harder J.; Fuchs B. M.; Rossello-Mora R.; Amann R. Cultivable Winogradskyella Species Are Genomically Distinct from the Sympatric Abundant Candidate Species. ISME Communications 2021 1:1 2021, 1 (1), 1–10. 10.1038/s43705-021-00052-w. PMC9723794 36747039

[ref87] Huang G.; Vidal-Melgosa S.; Sichert A.; Becker S.; Fang Y.; Niggemann J.; Iversen M. H.; Cao Y.; Hehemann J. H. Secretion of Sulfated Fucans by Diatoms May Contribute to Marine Aggregate Formation. Limnol Oceanogr 2021, 66 (10), 3768–3782. 10.1002/lno.11917.

