# Supplementary material for: Automated Synthesis of Algal Fucoidan Oligosaccharides
Source: J Am Chem Soc. 2024 Jun 25;146(27):18320–30. doi: 10.1021/jacs.4c02348 (PMC11240576; doi:10.1021/jacs.4c02348)
Supplement: Supplementary file 1 — ja4c02348_si_001.pdf [file ja4c02348_si_001.pdf]

Supporting Information

Automated synthesis of algal fucoidan oligosaccharides

Conor J. Crawford,[1] Mikkel Schultz-Johansen,[2,3] Phuong Luong,[1,4] Silvia Vidal-Melgosa,[2,3] Jan-Hendrik Hehemann,[2,3] Peter H. Seeberger*[1,4]

[1]Max Planck Institute for Colloids and Interfaces, Am Mühlenberg 1, 14476, Potsdam, Germany. [2]Max Planck Institute for Marine Microbiology, Celsiusstraße 1, 28359, Bremen, Germany. [3]MARUM, Center for Marine Environmental Sciences, University of Bremen, 28359, Bremen, Germany. [4]Institute for Chemistry and Biochemistry, Freie Universität Berlin, Arnimallee 22, 14195 Berlin, Germany.

Email: peter.seeberger@mpikg.mpg.de

Table of Contents

Supporting Tables and Figures 7

General Materials and Methods 24

4-Methylphenyl 1-thio-β-L-fucopyranoside (24) 30

4-Methylphenyl 4-*O*-benzoyl-2-*O*-benzyl-1-thio-β-L-fucopyranoside (25) 34

4-Methylphenyl 4-*O*-benzoyl-2-*O*-benzyl-3-*O*-(9-fluorenylmethoxycarbonyl)-1-thio-β-L-fucopyranoside (1) 38

4-Methylphenyl 2-*O*-benzyl-1-thio-β-L-fucopyranoside (26) 42

4-Methylphenyl 2-*O*-benzyl-4-*O*-levulinyl-1-thio-β-L-fucopyranoside (27) 46

4-Methylphenyl 2-*O*-benzyl-3-*O*-(9-fluorenylmethoxycarbonyl)-4-*O*-levulinyl-1-thio- β-L-fucopyranoside (2) 50

4-Methylphenyl 4-*O*-benzoyl-2-*O*-(2-naphthalenylmethyl)-1-thio-β-L-fucopyranoside (28) 54

4-Methylphenyl 4-*O*-benzoyl-3-*O*-(9-fluorenylmethoxycarbonyl)-2-*O*-(2-naphthalenylmethyl)-1-thio-β-L-fucopyranoside (3) 57

4-Methylphenyl 4-*O*-benzoyl-2-*O*-benzyl-3-*O*-(2-napthalenylmethyl)-1-thio-β-L-fucopyranoside (4) 60

Ethyl 1-thio-β-L-fucopyranoside (29) 64

Ethyl 4-*O*-benzoyl-2-*O*-benzyl-1-thio-β-L-fucopyranoside (30) 68

Ethyl 4-*O*-benzoyl-2-*O*-benzyl-3-*O*-(9-fluorenylmethoxycarbonyl)-1-thio-β-L-fucopyranoside (31) 71

Dibutyl 4-*O*-benzoyl-2-*O*-benzyl-3-*O*-(9-fluorenylmethoxycarbonyl)-1-phosphate-L-β-fucopyranoside (32) 75

Ethyl 4,6-*O*-benzylidene-3-*O*-(2-naphthalenylmethyl)-1-thio-β-D-galactoside (33) 80

Ethyl 2-*O*-benzoyl-4,6-*O*-benzylidene-3-*O*-(2-naphthalenylmethyl)-1-thio-β-D-galactoside (34) 84

Ethyl 2-*O*-benzoyl-4-*O*-benzyl-3-*O*-(2-naphthalenylmethyl)-1-thio-β-D-galactoside (35) 88

Ethyl 2-*O*-benzoyl-4-*O*-benzyl-6-*O*-(9-fluorenylmethoxycarbonyl)-3-*O*-(2-naphthalenylmethyl)-1-thio-β-D-galactoside (36) 92

Ethyl 2-*O*-benzoyl-4-*O*-benzyl-6-*O*-(9-fluorenylmethoxycarbonyl)-1-thio-β-D-galactoside (37) 96

Ethyl 2-*O*-benzoyl-4-*O*-benzyl-6-*O*-(9-fluorenylmethoxycarbonyl)-3-*O*-levulinyl-1-thio-β-D-galactoside (38) 100

Dibutyl 2-*O*-benzoyl-4-*O*-benzyl-6-*O*-(9-fluorenylmethoxycarbonyl)-3-*O*-levulinyl-1-phosphate-α,β-D-galactoside (5) 104

Automated glycan assembly 109

Solid-phase synthesis 117

Solution-phase synthesis 118

HPLC analysis and purification 118

LC-MS analysis of oligosaccharides 121

α-L-fucopyranosyl-(1→3)-α-L-fucopyranosyl-(1→3)-α-L-fucopyranosyl-(1→3)-α-L-fucopyranosyl-(1→3)-α-L-fucopyranosyl-(1→3)-α-L-fucopyranose (6) 136

Amino pentyl α-L-fucopyranosyl-(1→3)-α-L-fucopyranosyl-(1→3)-α-L-fucopyranosyl-(1→3)-α-L-fucopyranosyl-(1→3)-α-L-fucopyranoside (7) 140

5-amino pentyl α-L-fucopyranosyl-(1→3)-α-L-fucopyranosyl-(1→3)-α-L-fucopyranosyl-(1→3)-α-L-fucopyranosyl-(1→3)-α-L-fucopyranosyl-(1→3)-α-L-fucopyranosyl-(1→3)-α-L-fucopyranosyl-(1→3)-α-L-fucopyranoside (8) 143

5-Aminopentyl 3,4-di-*O*-sulfonate-α-L-fucopyranosyl-(1→3)-4-*O*-sulfonate-α-L-fucopyranoside (9) 146

3,4-di-*O*-sulfonate-α-L-fucopyranosyl-(1→3)-4-*O*-sulfonate-α-L-fucopyranosyl-(1→3)-4-*O*-sulfonate-α-L-fucopyranosyl-(1→3)-4-*O*-sulfonate-α-L-fucopyranosyl-(1→3)-4-*O*-sulfonate-α-L-fucopyranosyl-(1→3)-4-*O*-sulfonate-α-L-fucopyranose (10) 149

5-Aminopentyl 3,4-di-*O*-sulfonate-α-L-fucopyranoside (11) 153

5-aminopentyl α-L-fucopyranosyl-(1→3)-α-L-fucopyranosyl-(1→3)-α-L-fucopyranosyl-(1→3)-α-L-fucopyranosyl-(1→3)-α-L-fucopyranoside-α-L-fucopyranosyl-(1→3)-α-L-fucopyranosyl-(1→3)-α-L-fucopyranosyl-(1→3)-α-L-fucopyranosyl-(1→3)-α-L-fucopyranoside-α-L-fucopyranosyl-(1→3)-α-L-fucopyranosyl-(1→3)-α-L-fucopyranosyl-(1→3)-α-L-fucopyranosyl-(1→3)-α-

L-fucopyranoside-α-L-fucopyranosyl-(1→3)-α-L-fucopyranosyl-(1→3)-α-L-fucopyranosyl-(1→3)-α-L-fucopyranosyl-(1→3)-α-L-fucopyranoside (12) 156

5-Aminopentyl 3,4-di-*O*-sulfonate-α-L-fucopyranosyl-(1→3)-4-*O*-sulfonate-α-L-fucopyranosyl-(1→3)-4-*O*-sulfonate-α-L-fucopyranosyl-(1→3)-4-*O*-sulfonate-α-L-fucopyranoside (13) 161

5-Aminopentyl 3,4-di-*O*-sulfonate-α-L-fucopyranosyl-(1→3)-4-*O*-sulfonate-α-L-fucopyranosyl-(1→3)-4-*O*-sulfonate-α-L-fucopyranosyl-(1→3)-4-*O*-sulfonate-α-L-fucopyranosyl-(1→3)-4-*O*-sulfonate-α-L-fucopyranosyl-(1→3)-4-*O*-sulfonate-α-L-fucopyranosyl-(1→3)--4-*O*-sulfonate-α-L-fucopyranosyl-(1→3)-4-*O*-sulfonate-α-L-fucopyranosyl-(1→3)-4-*O*-sulfonate-α-L-fucopyranosyl-(1→3)-4-*O*-sulfonate-α-L-fucopyranoside (14) 168

5-aminopentyl α-L-fucopyranosyl-(1→3)-α-L-fucopyranosyl-(1→4)-α-L-fucopyranosyl-(1→3)-α-L-fucopyranoside (15) 175

5-aminopentyl α-L-fucopyranosyl-(1→3)-α-L-fucopyranosyl-(1→4)-α-L-fucopyranosyl-(1→3)-α-L-fucopyranosyl-(1→4)-α-L-fucopyranosyl-(1→3)-α-L-fucopyranoside (16) 178

5-aminopentyl 3,4-di-*O*-sulfonate-α-L-fucopyranosyl-(1→3)-4-*O*-sulfonate-α-L-fucopyranosyl-(1→4)-3-*O*-sulfonate-α-L-fucopyranosyl-(1→3)-4-*O*-sulfonate-α-L-fucopyranosyl-(1→4)-3-*O*-sulfonate-α-L-fucopyranosyl-(1→3)-4-*O*-sulfonate-α-L-fucopyranoside (17) 182

5-aminopentyl α-L-fucopyranosyl-(1→3)-[α-L-fucopyranosyl-(1→2)]-α-L-fucopyranosyl-(1→3)-α-L-fucopyranosyl-(1→3)-α-L-fucopyranosyl-(1→3)-α-L-fucopyranoside (18) 187

5-aminopentyl α-L-fucopyranosyl-(1→3)-[β-D-galactosyl-(1→4)]-α-L-fucopyranosyl-(1→3)-α-L-fucopyranosyl-(1→3)-α-L-fucopyranoside (19) 190

5-aminopentyl β-D-galactosyl-(1→6)-β-D-galactosyl-(1→4)-[α-L-fucopyranosyl-(1→3)]-α-L-fucopyranosyl-(1→3)-α-L-fucopyranosyl-(1→3)-α-L-fucopyranoside (20) 193

5-aminopentyl α-L-fucopyranosyl-(1→3)-α-L-fucopyranosyl-(1→3)-[α-L-fucopyranosyl-(1→3)-α-L-fucopyranosyl-(1→4)]-α-L-fucopyranosyl-(1→3)-α-L-fucopyranosyl-(1→3)-α-L-fucopyranoside (21) 197

References 201

Supporting Tables and Figures

SI Table 1. Optimisation of automated assembly of the fucoidan oligosaccharides.

Entry	T1 (mins)	Donor (Leaving group)	T2 (mins)	Cycle	Activator	Comment
**1**	-30 °C (10)	**31** (SEt)	0 °C (30)	1	NIS-TfOH	Deletion sequences
**2**	-30 °C (10)	**31** (SEt)	-10 °C (20)	2	NIS-TfOH	Deletion sequences
**3**	-40 °C (10)	**31** (SEt)	-10 °C (20)	2	NIS-TfOH	Target mass, irreproducible synthesis.
**4**	-40 °C (10)	**31** (SEt)	-10 °C (20)	1	NIS-TMSOTf	Target mass with deletion sequences, irreproducible synthesis
**5**	−30 °C (10)	**32** (dibutyl phosphate)	−15 °C (25)	1	TMSOTf	Deletion sequences
**6**	−40 °C (15)	**32** (dibutyl phosphate)	−10 °C (25)	1	TMSOTf	Deletion sequences
**7**	-40 °C (10)	**1** (STol)	-10 °C (20)	2	NIS-TfOH	Target mass
**8**	-20 °C (15)	**1** (STol)	0 °C (35)	1	NIS-TfOH	Target mass

SI Table 2. Annotating structural reporter groups with NMR of fucoidan oligosaccharides.

	^{1}H	^{13}C
**Anomeric peaks**		
H-1 α-(reducing end) non-sulfated	5.19	92.0
H-1 β-(free reducing end) non-sulfated	4.57	96.0
H-1 α-(1→3) non-sulfated	5.10 — 4.91	100.4 — 95.1
H-1 α-(1→4) non-sulfated	5.07 — 5.06	96.2 — 95.6
H-1 α-(reducing end) sulfated	5.20	92.4
H-1 β-(free reducing end) sulfated	4.60 — 4.53	96.2
H-1 α-(1→3) 4-*O*-sulfated	5.39 — 5.24	100.4 — 98.4
H-1 α-(1→4) 4-*O*-sulfated	5.35 — 5.32	96.2
H-1 α-(1→2) (**18**)	5.33	91.7
H-1 α-(1→4) (**21**)	5.15	95.5
$H\text{-}1_{gal}$ Gal-β-(1→4)-fuc (**19** and **20**)	4.58 — 4.54	103.4 — 103.3
$H\text{-}1_{gal}$ Gal-β-(1→6)-gal (**20**)	4.41 — 4.37	103.3
***O*-sulfation shifts**		
H-4 *O*-sulfation	5.06 — 4.62	79.6 — 78.2
H-3 *O*-sulfation	4.59 — 4.45	76.8 — 75.3
**Methyl peaks**		
CH_3	1.50 — 1.15	15.9 — 15.2

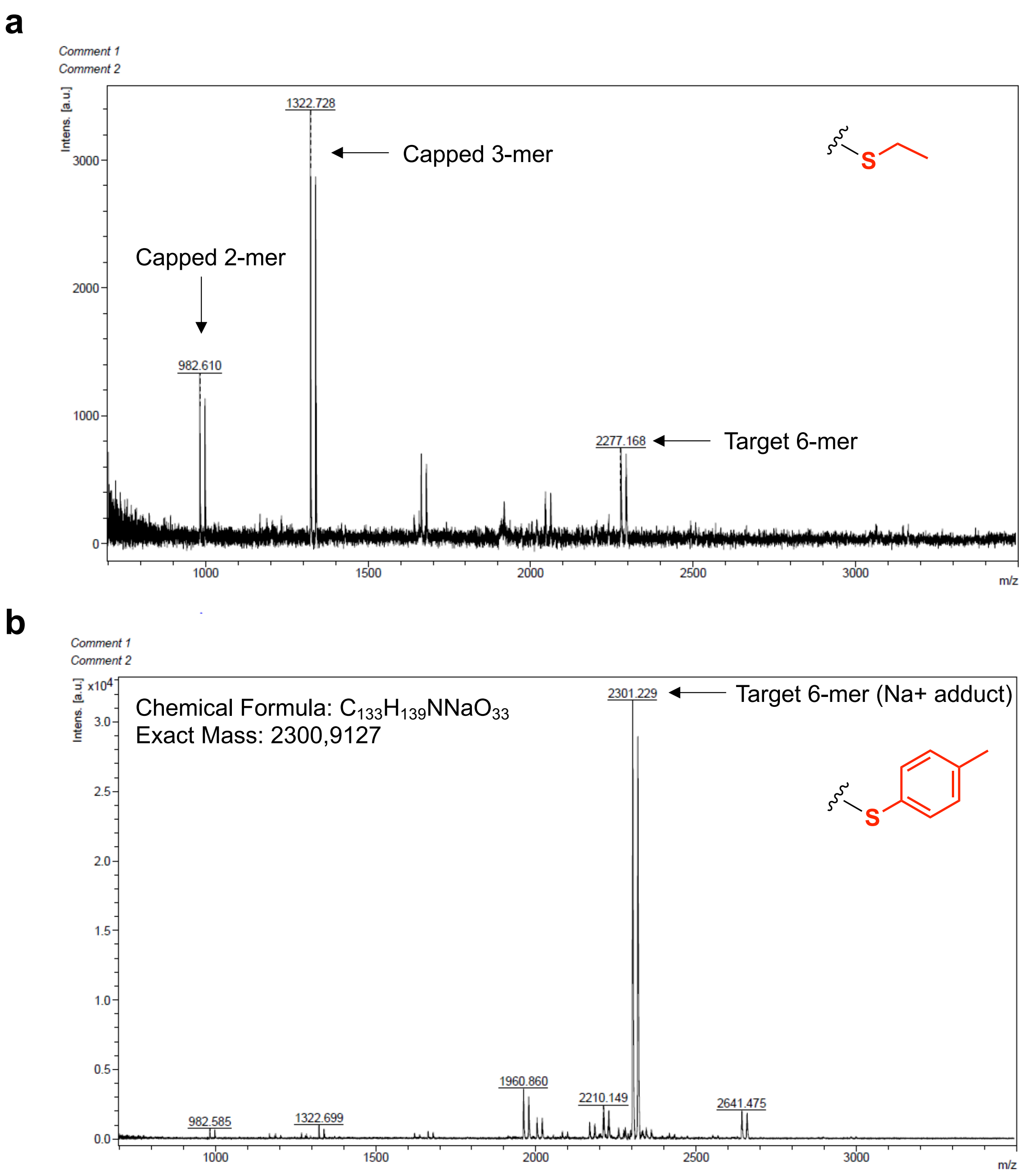

SI Figure 1. MALDI-TOF analysis of automated assembly of 6-mers with different thioglycoside aglycons. a. spectrum of thioethyl donor and **b.** spectrum of 4-methyl phenyl donor.

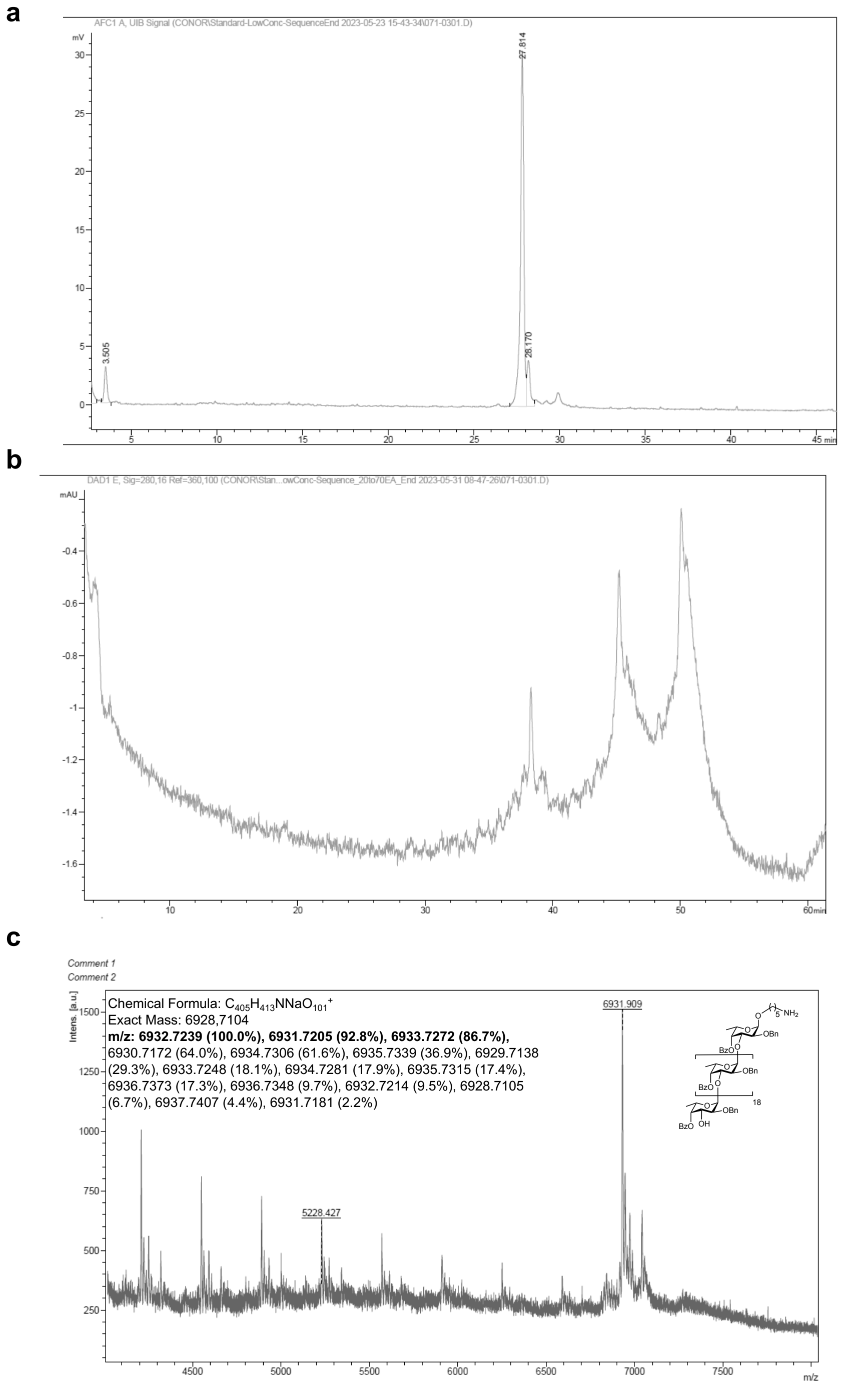

SI Figure 2. Automated assembly of α-fucan 20-mer. a. NP-HPLC (20 to 55EA 35 min), ELSD trace of 10-mer fucan intermediate **b.** NP-HPLC (20 to 70EA 55 min), DAD 280nm trace of 20-mer intermediate **c.** MALDI-TOF MS of protected 20-mer. During the microcleavage loss of the CBz was observed, the potential for this has also been noted in other reports.[1]

a

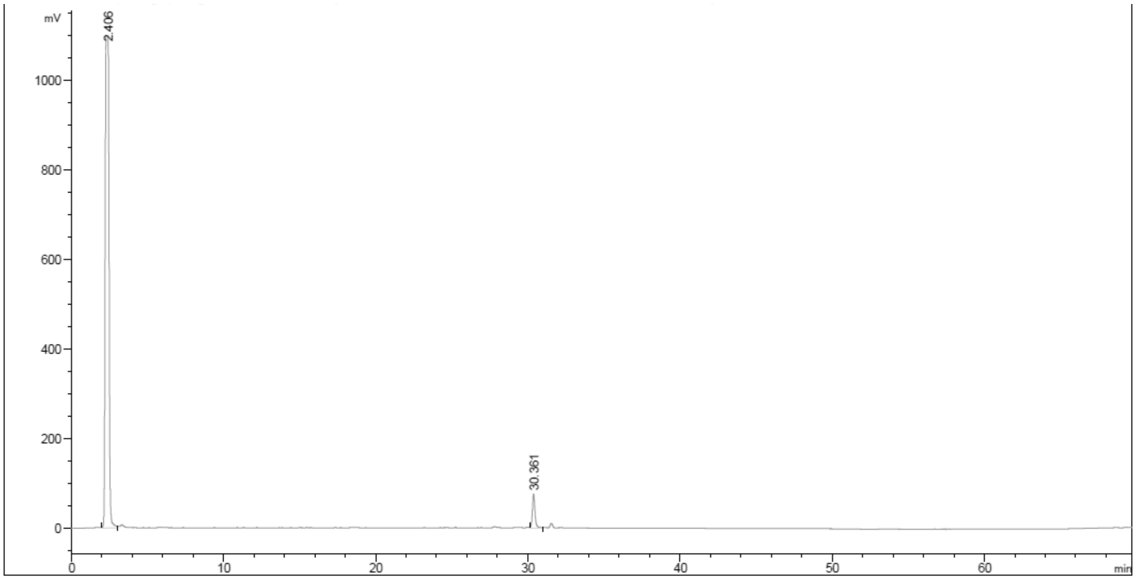

b

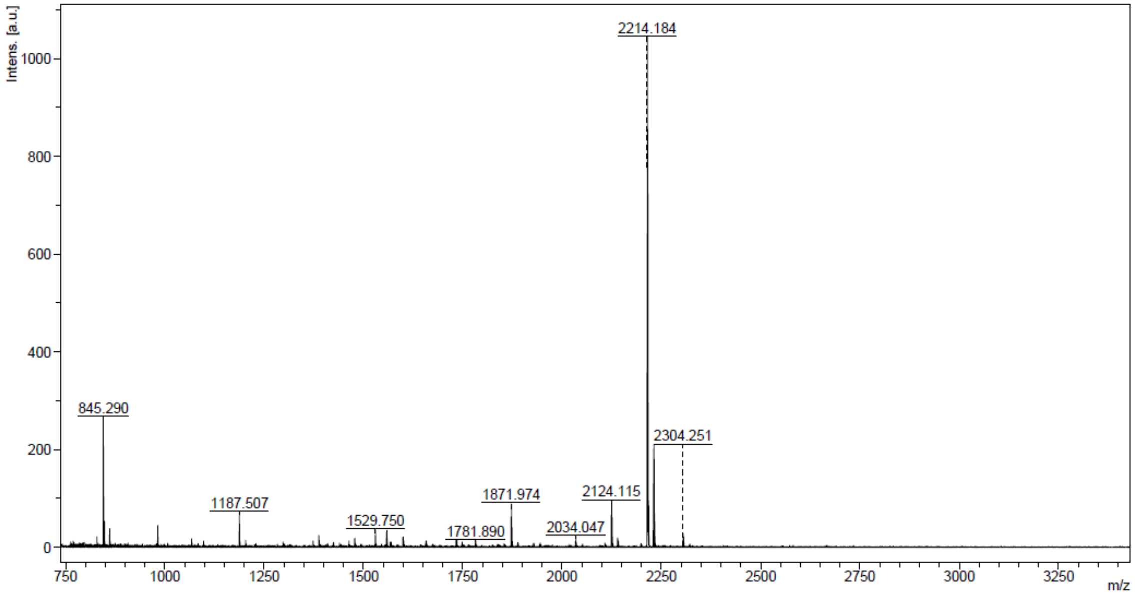

SI Figure 3. Automated assembly of fucoidan oligosaccharide 16. a. NP-HPLC (HPLC Method: 10 to 60 EA, 40 mins), ELSD trace of 6-mer fucan intermediate. **b.** MALDI-TOF MS of protected 6-mer.

a

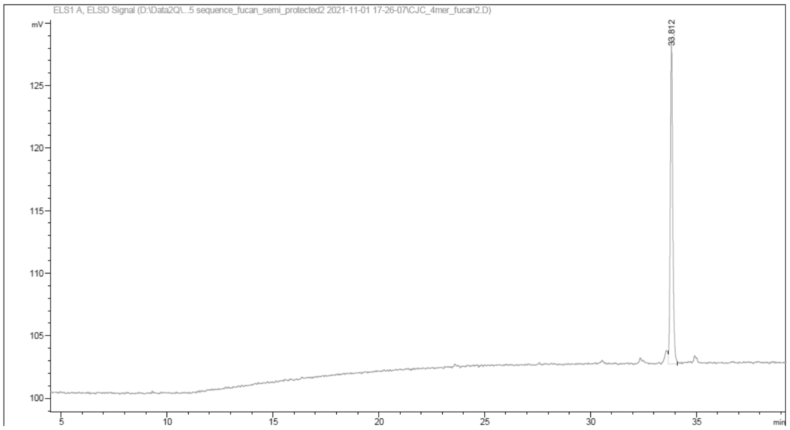

b

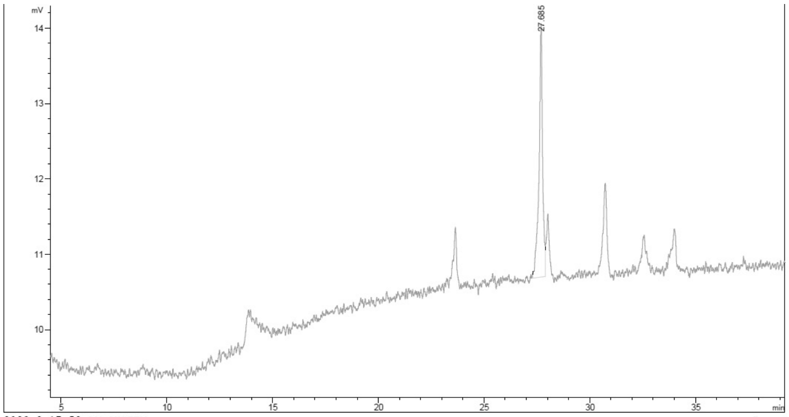

c

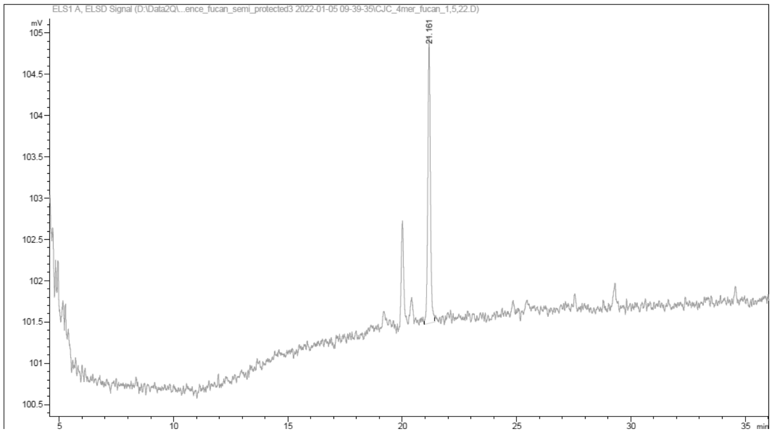

SI Figure 4. Tracking on-resin sulfation by RP-HPLC for 13. Using method, Luna C5 column, 5 to 100 ACN, 35 mins, the progress of sulfation reaction could be tracked. **a.** ELSD trace of the non-sulfated 4-mer fucan starting material. **b.** Incomplete sulfation of the 4-mer intermediate. **c.** Semi-protected sulfated 4-mer fucan intermediate. Decreasing retention time signifies increasing level of sulfation.[2]

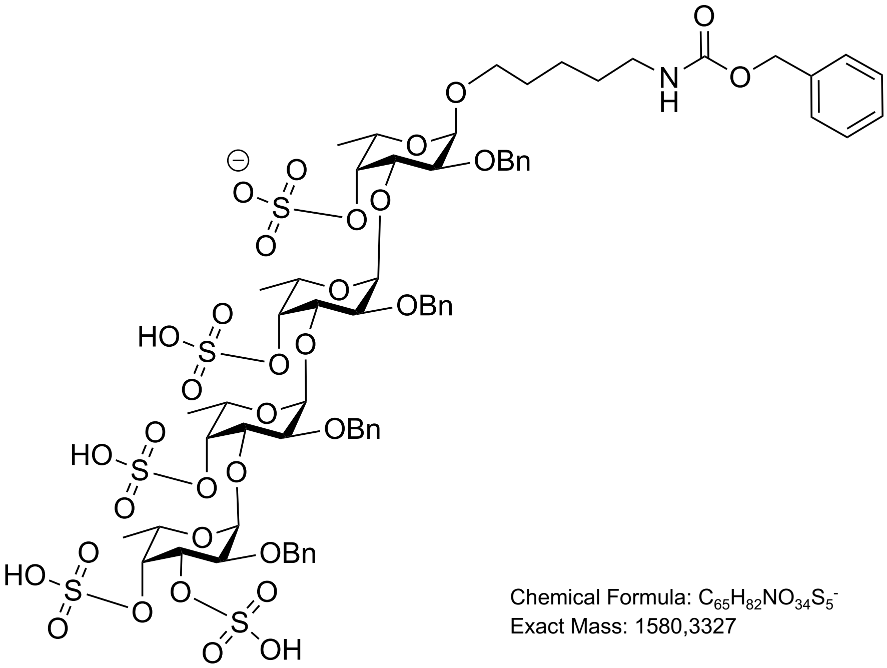

Chemical Formula: $C_{65}H_{82}NO_{34}S_5^-$
Exact Mass: 1580,3327

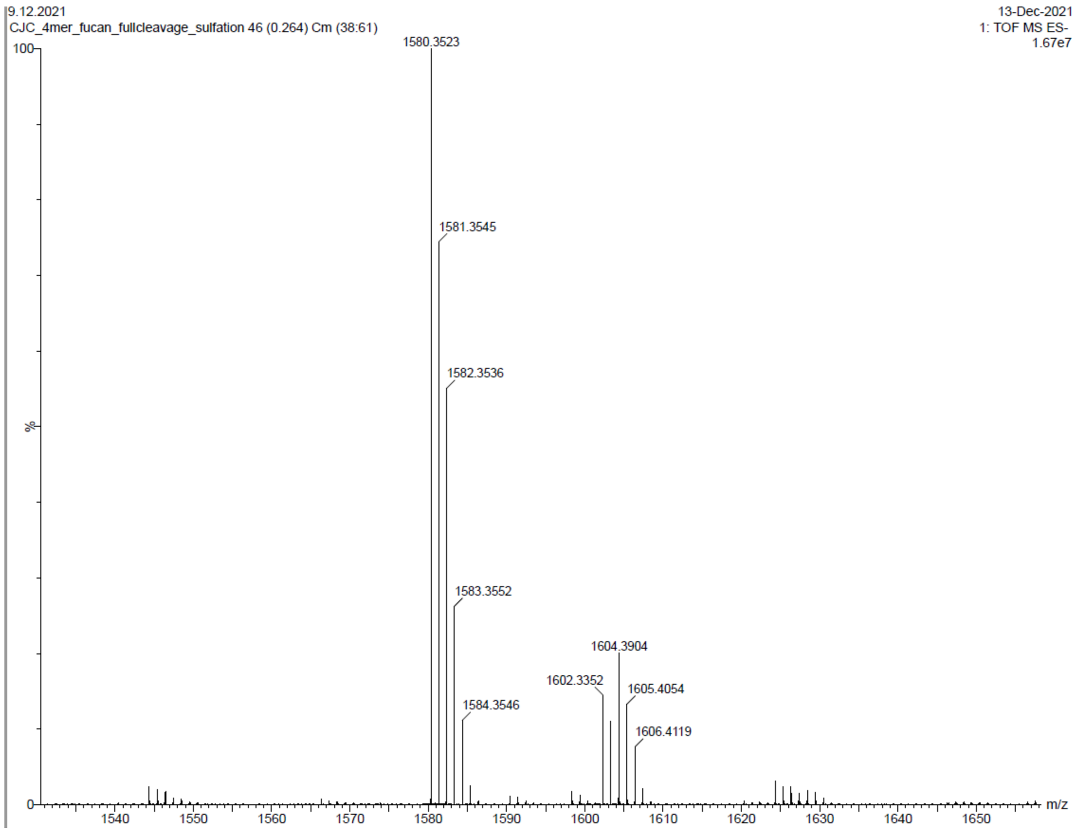

SI Figure 5. An example of on-resin sulfation analysed by Q-TOF.

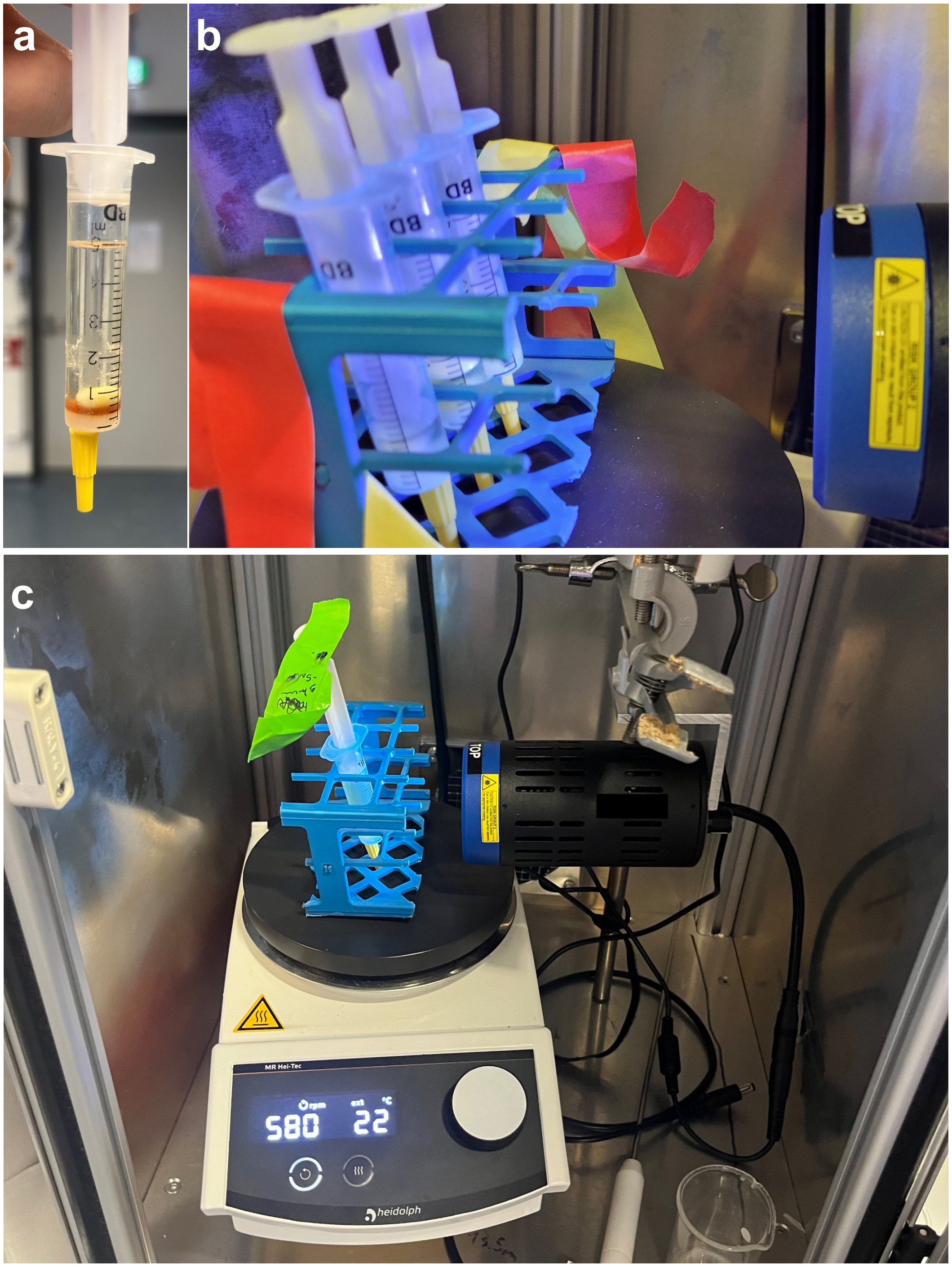

SI Figure 6. Photocleavage of glycans from solid-support in batch. a. Resin with stir bar inside a fritted syringe. **b.** Parallel cleavage of multiple resins. **c.** An overview of the set-up.

a

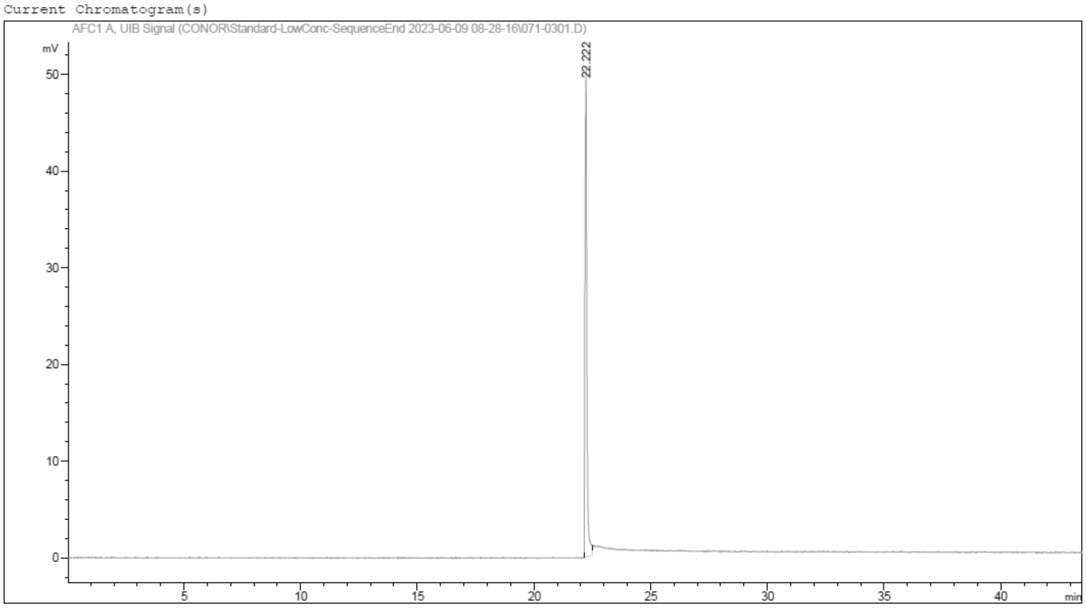

b

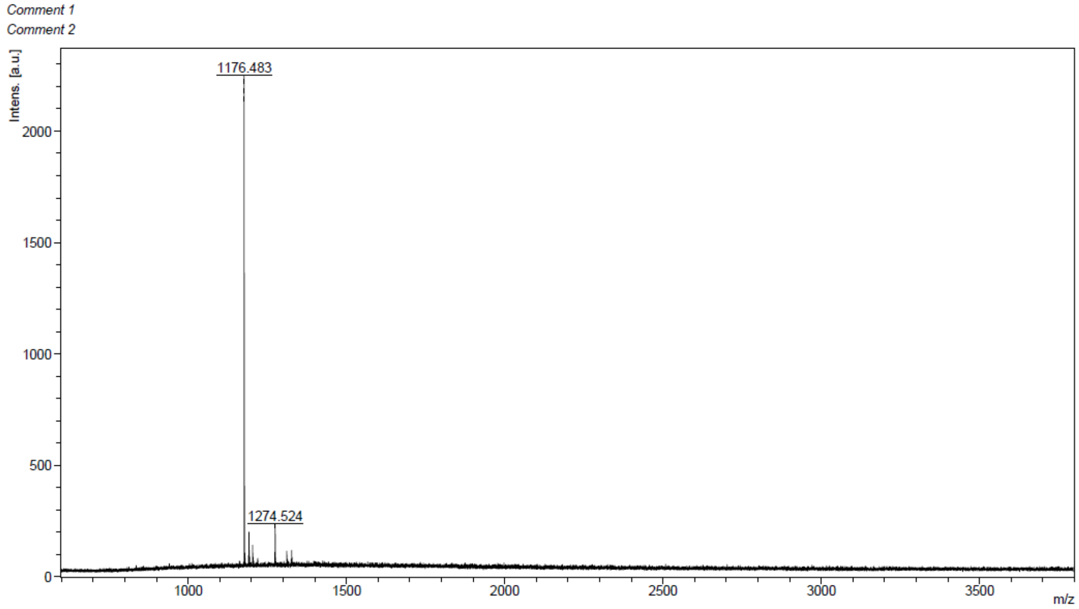

SI Figure 7. Diol intermediate towards the synthesis of an α-1,4 linkage containing fucoidan oligosaccharide. a. NP-HPLC, 20 to 55EA, ELSD Trace. **b.** MALDI-TOF, target mass of diol observed at 1176 *m/z* with a sodium adduct.

a

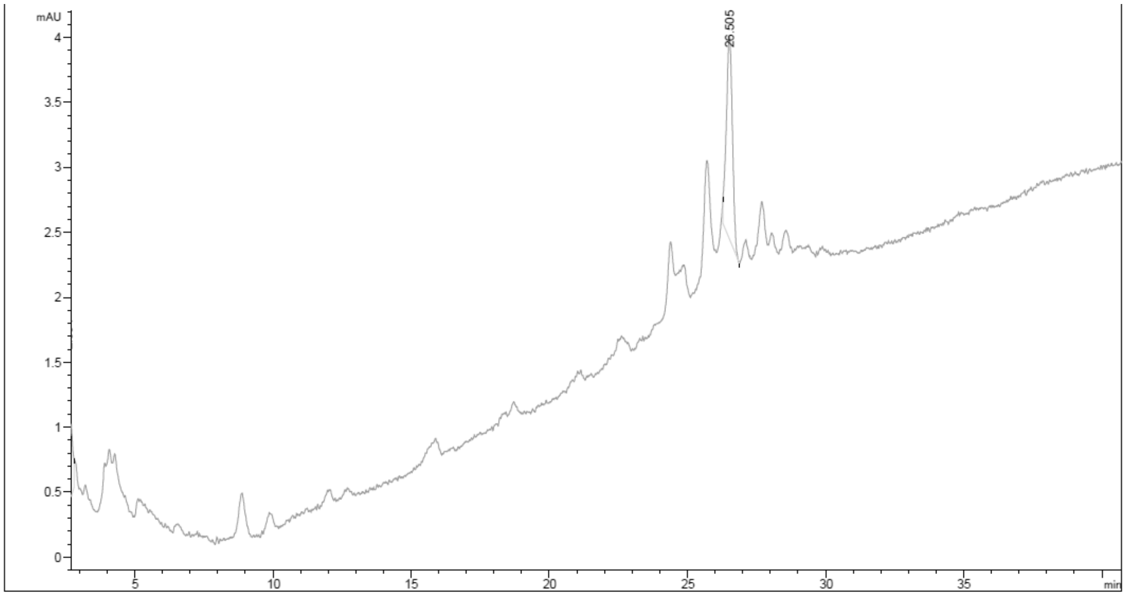

b

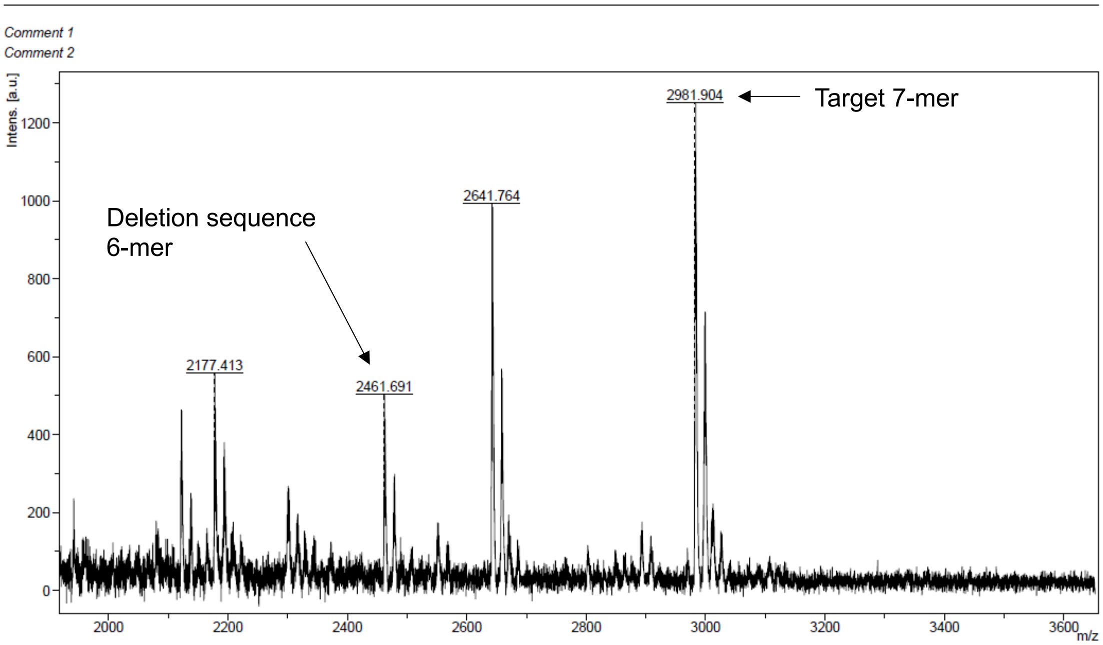

SI Figure 8. Automated assembly of an α-1,4 linkage containing fucoidan oligosaccharide. a. NP-HPLC, 20 to 55EA, UV 270nm trace. **b.** MALDI-TOF spectrum.

a

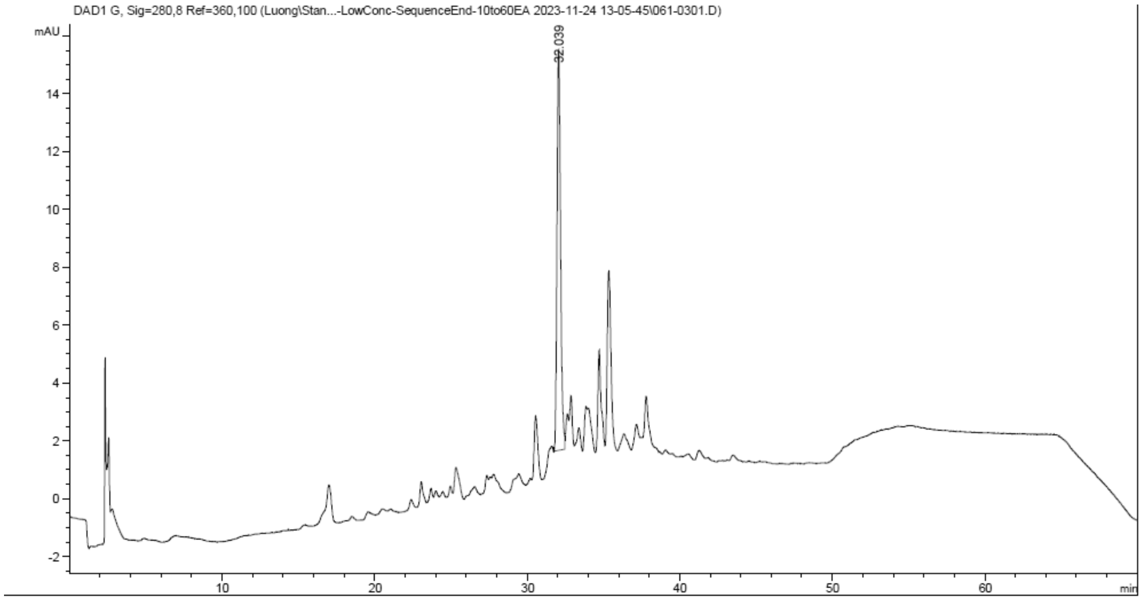

b

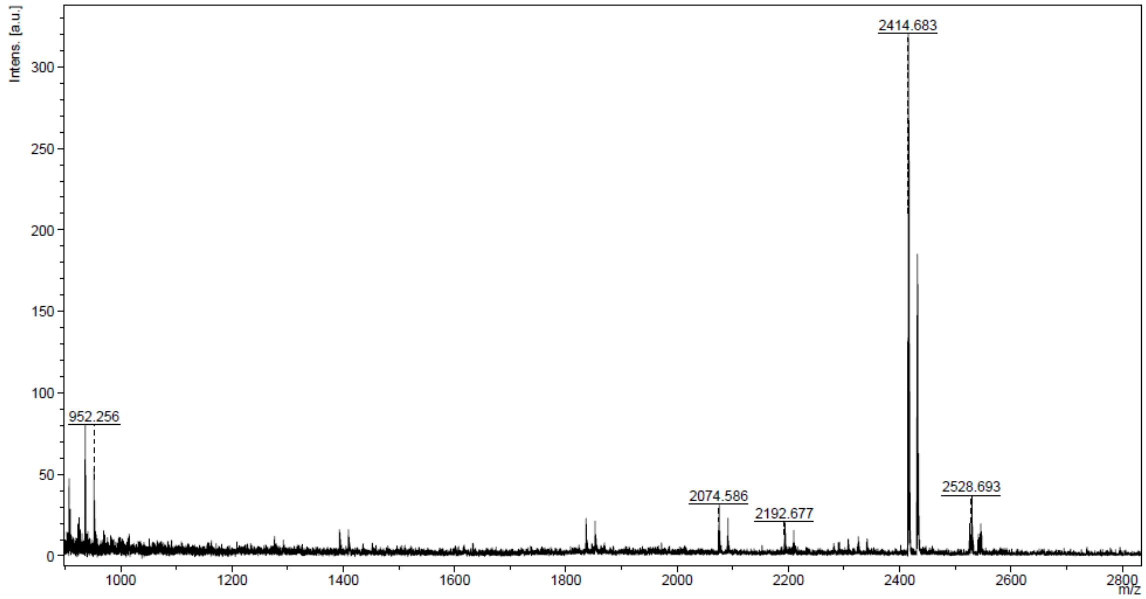

SI figure 9 Automated assembly of fucoidan oligosaccharide 21. a. NP-HPLC, 10 to 60 EA, 40 mins UV 290nm trace. **b.** MALDI-TOF spectrum.

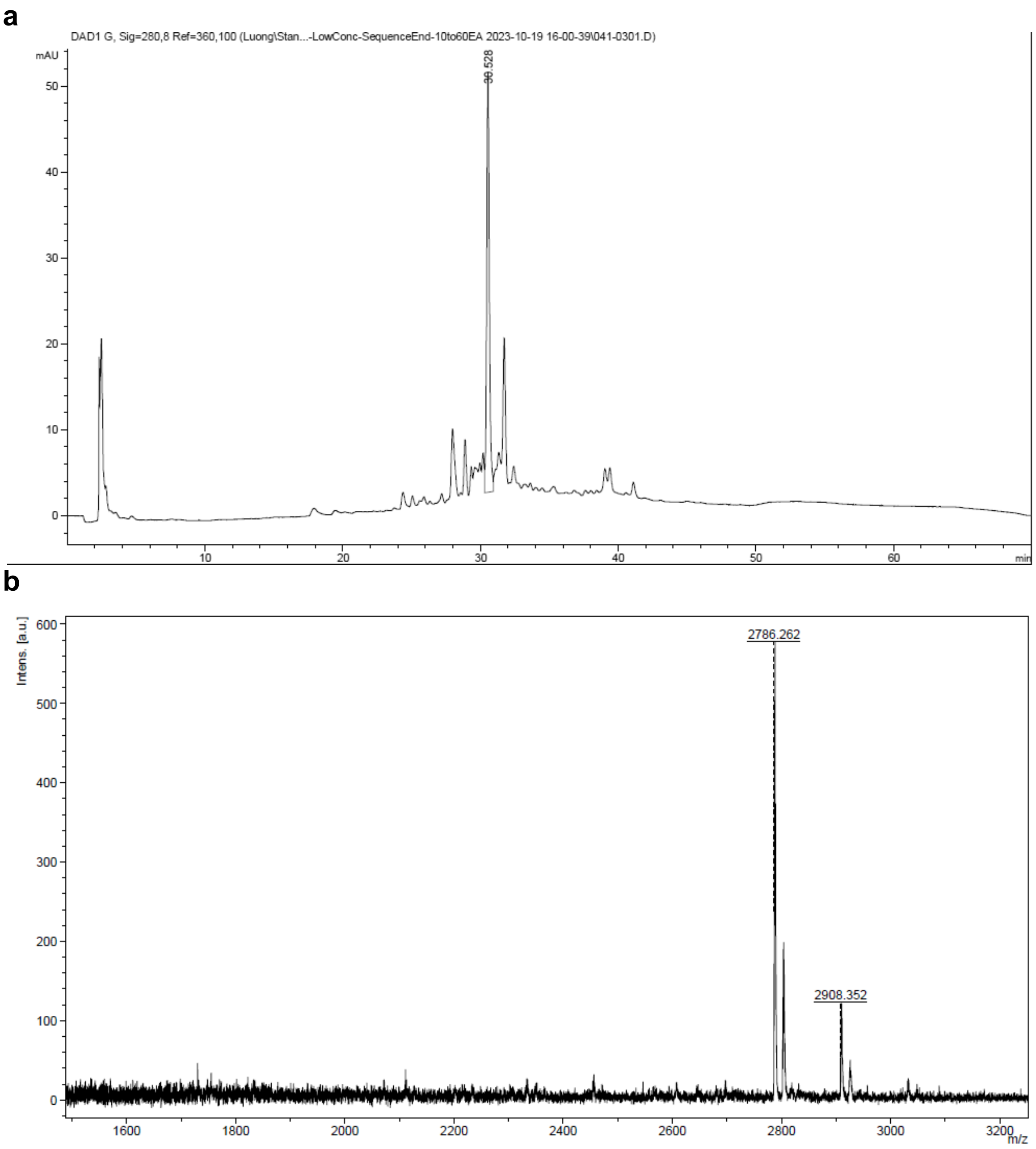

SI Figure 10. Automated assembly of fucoidan oligosaccharide 22. a. NP-HPLC, 10 to 60 EA, 40 mins UV 290nm trace. **b.** MALDI-TOF spectrum.

a

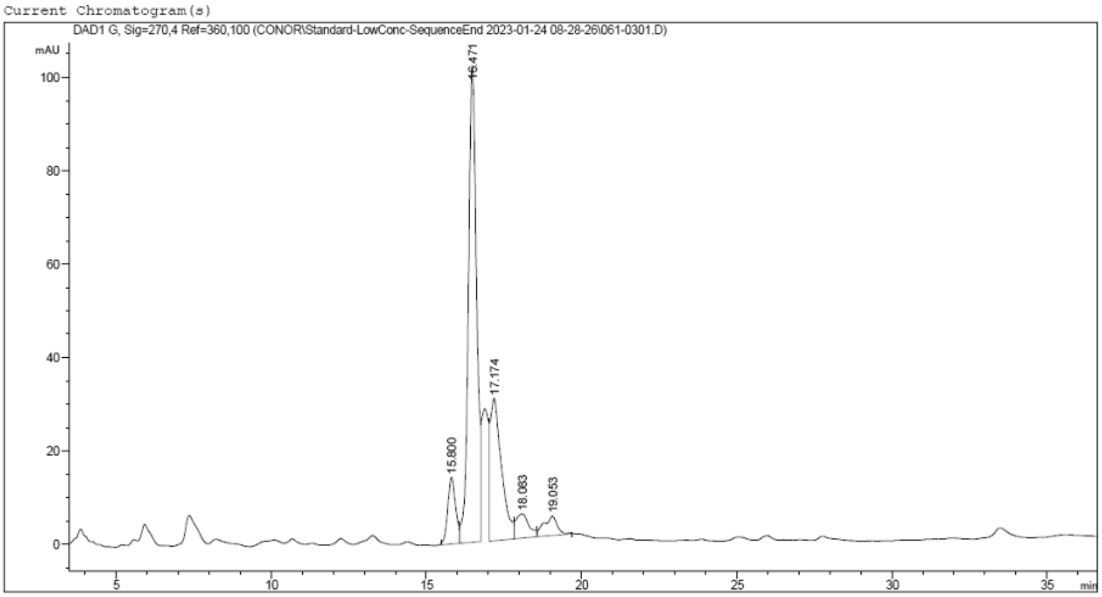

b

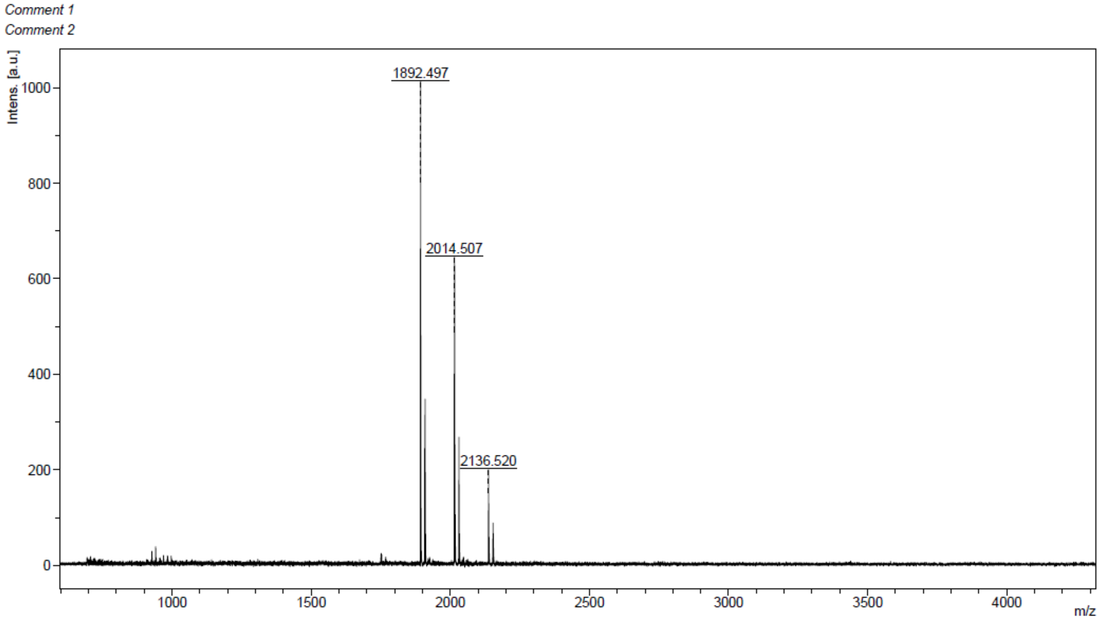

SI Figure 11. Intermediates in the synthesis of fucoidan oligosaccharide 20 containing an α-1,2 linkage. a. NP-HPLC (20 to 55 EA), 270 nm trace of the 4-mer intermediate. **b.** MALDI-TOF of the 4-mer intermediate (1892 *m/z*) with a sodium adduct. Additionally, observed smaller peaks of *m/z* of +122.

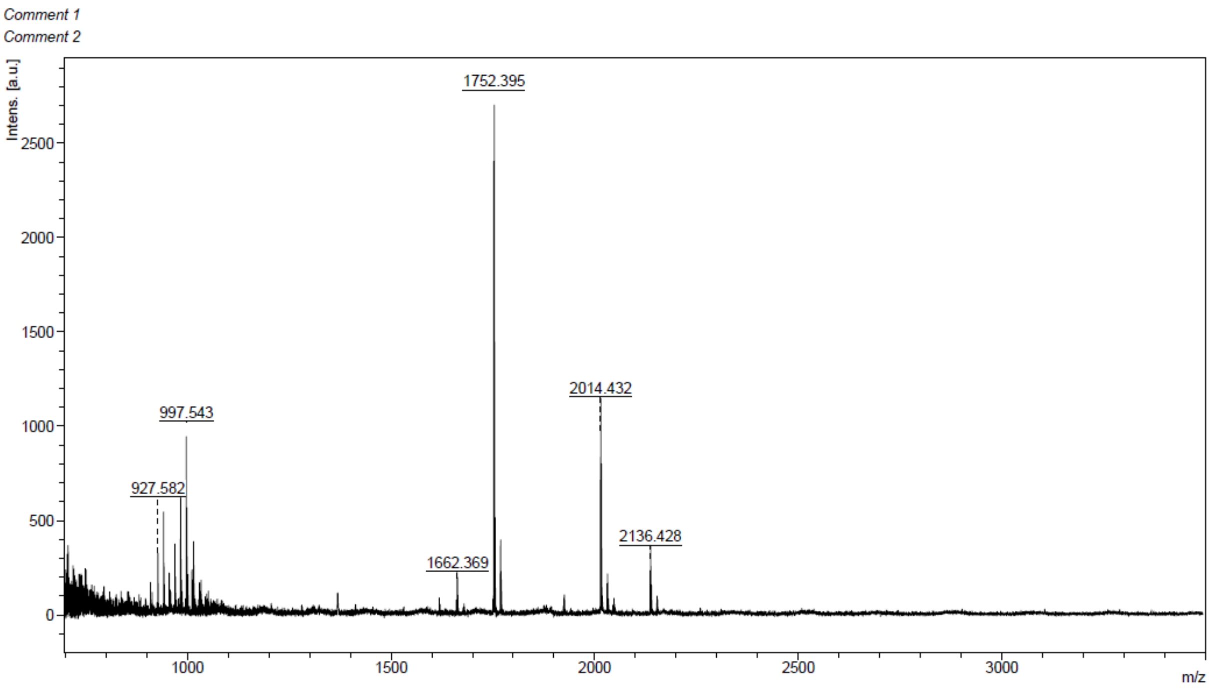

SI Figure 12. MALDI-TOF of the 4-mer intermediate after 2-naphthylmethyl ether cleavage. Target mass observed with sodium adduct at 1752 *m/z*.

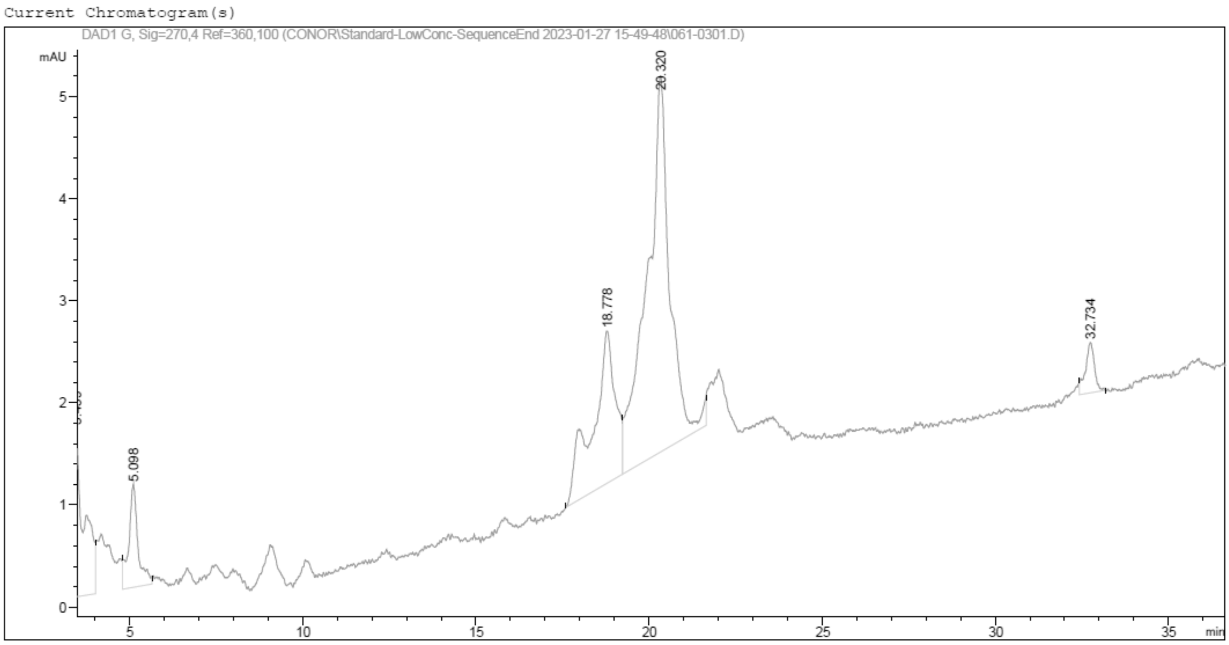

SI Figure 13. NP-HPLC trace of hexasaccharide fucoidan oligosaccharide 20 containing an α-1,2 linkage.

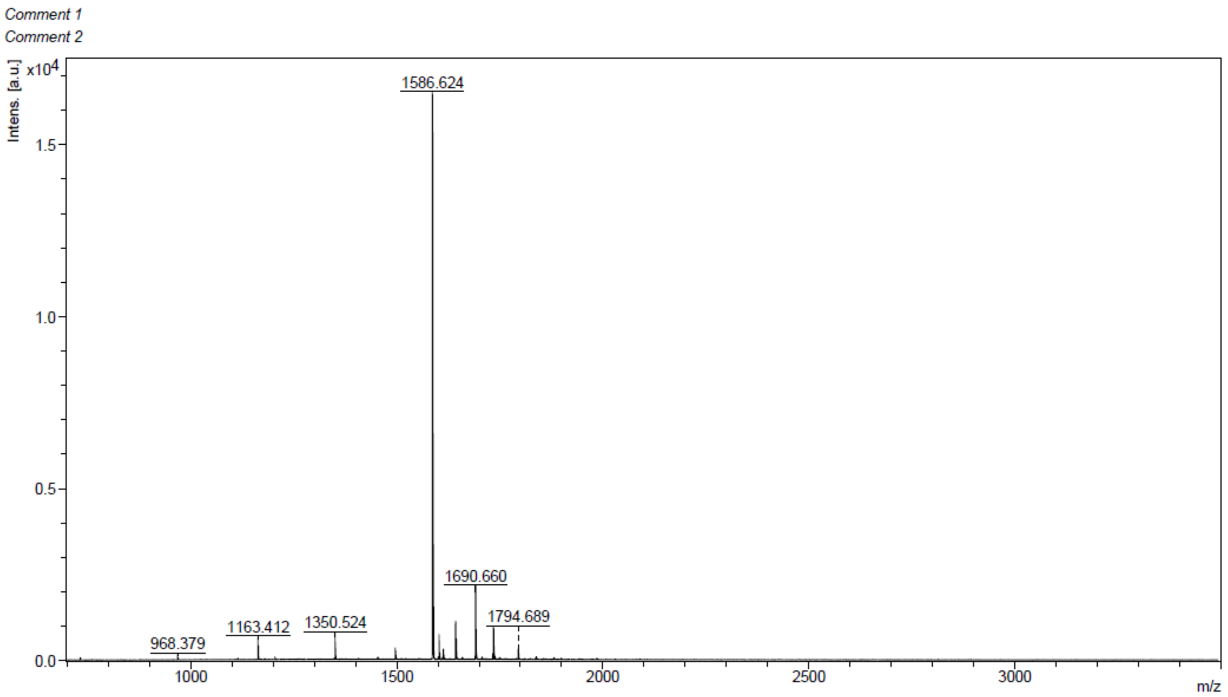

SI Figure 14. MALDI-TOF of fucoidan hexasaccharide 20 after methanolysis. Target mass observed with sodium adduct at 1586 *m/z*.

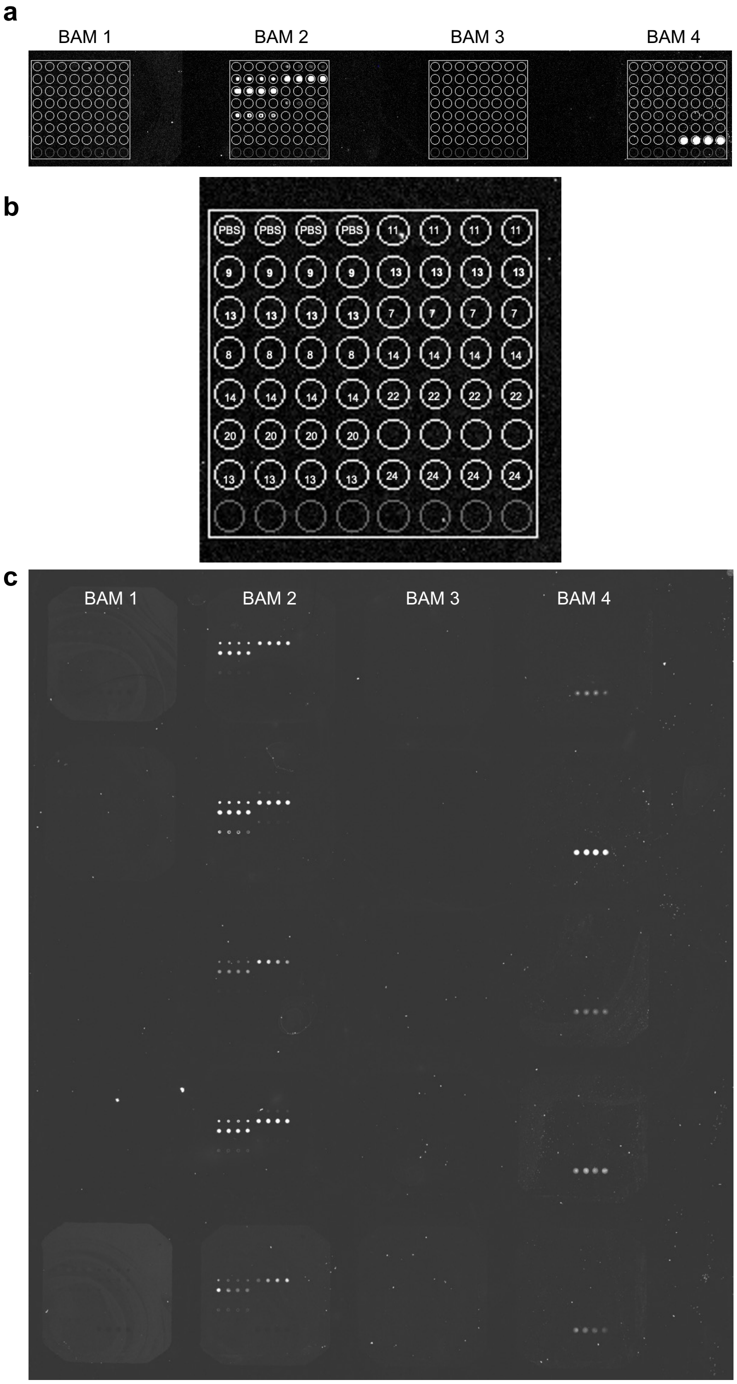

SI Figure 15. Fucoidan microarray. a. grid showing location of oligosaccharide spots. **b.** specific glycan spots. **c.** the binding profiles of BAMs 1-4 across a range of concentrations. Antibodies were diluted in 3% BSA-PBS across a range of concentrations. The first row is a 1:2 dilution, followed by a 1:10, followed by 1:100,

followed by 1:50 dilution and finally no dilution. Image is representative of duplicate experiments.

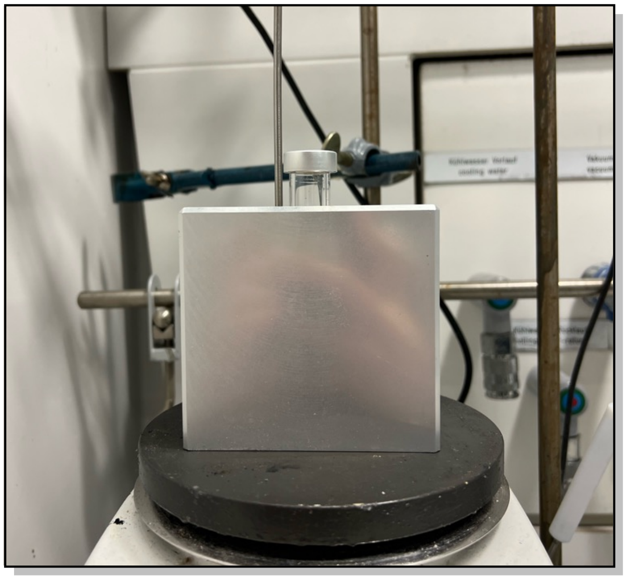

Sulfation module

S

Py.SO_3, DMF:Py (80:20 v/v) 50°C

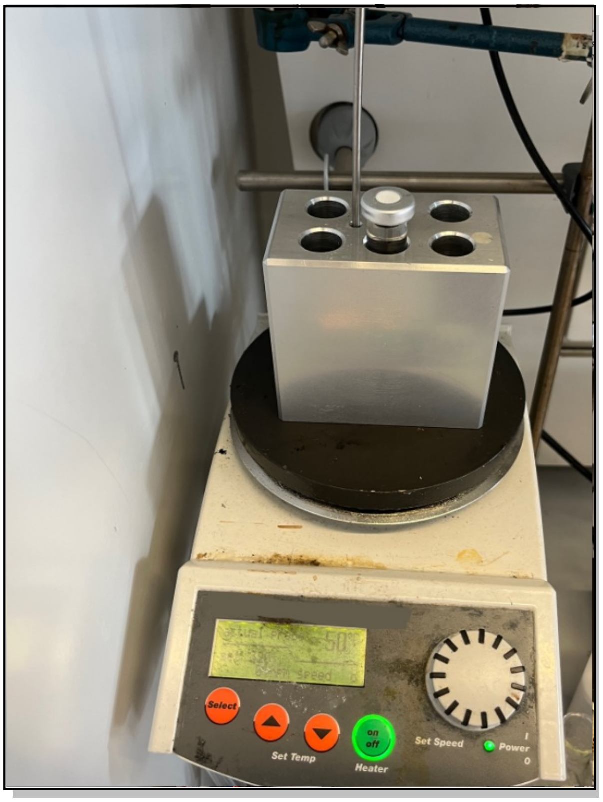

SI Figure 16. Sealable microwave vial used for sulfation reactions with an aluminium heating block.

General Materials and Methods

All chemicals used were reagent grade and used as supplied unless otherwise noted. The automated syntheses were performed on a home-built synthesizer developed at the Max Planck Institute of Colloids and Interfaces. Analytical thin-layer chromatography (TLC) was performed on Merck silica gel 60 F254 plates (0.25 mm). Compounds were visualized by UV irradiation or dipping the plate in a 5% H_2SO_4 ethanol solution. Flash column chromatography was carried out on automated Grace flash chromatography system. Analysis and purification by normal and reverse phase HPLC was performed by using an Agilent 1200 series. Products were lyophilized using a Christ Alpha 2-4 LD plus freeze dryer. ^1H, ^{13}C and HSQC NMR spectra were recorded on a Varian 400MR (400 MHz), Varian 600MR (600 MHz), or Bruker Biospin AVANCE700 (700 MHz) spectrometer. Signals are reported in terms of chemical shift [δ in parts per million (ppm)] relative to tetramethylsilane (TMS) or in D_2O using the solvent as the internal standard in ^1H NMR (D2O: 4.79 ppm ^1H). NMR data is presented as follows: Chemical shift, multiplicity (s = singlet, d = doublet, t = triplet, dd = doublet of doublet, m = multiplet and/or multiple resonances), coupling constant in Hertz (Hz), integration. All NMR signals were assigned on the basis of ^1H NMR, ^{13}C NMR, COSY, TOCSY and HSQC experiments. ‡Carbon assignments were extracted from HSQC experiment. High resolution mass spectra were obtained using a 6210 ESI-TOF mass spectrometer (Agilent) and a MALDI-TOF autoflexTM (Bruker). MALDI and ESI mass spectra were run on IonSpec Ultima instruments. The matrix used for MALDI-MS was 2,5-dihydroxy-benzoic acid (DHB).

Cloning and expression of enzymes

The gene encoding GH107_P5AFcnA from *Psychromonas* sp. SW5A was inserted into a pET28a+ expression vector as previously described.[3]

The gene encoding GH107 Wv323 was identified via a BLASTp search (default settings) against Genbank-NCBI using the protein sequence of GH107_P5AFcnA (acc. no. AYF59291.1) as query and retrieved from the genome of *Winogradskyella vidalii* HL634^T (NZ_JABFDG010000001). The gene sequence (nucleotides 58-1689) was codon-optimized for *E. coli*, and the synthesized GH107 Wv323 gene construct was inserted into a pET28a+ expression vector between the NheI and XhoI sites (BioCat, Germany):

NheI //
CAGCAGGTGTGGTATGAAAATAGTAGCCAGACCAATAATATCCGCTTCAGCAATACCCAGAAAGGCCTGTTCACCACCGATGAA
ACCAATCCGGAAACCACCGGCATTAATACCAATGCAAGCGTTAGCAAATTCGTGCGCGATGGCCAGGAAAATAGCCGCATTATC
TTCGATCTGAGCGTTCCGATTACCGATCTGACCAGCAATACCATTGCACTGAAAGCATATACCAGTATTCAGACCAGCGATCTG
AATACCACCAATAGCCGCATCCGCCTGTTCCTGAAAAATAGCAGCATTGGTGGCAGCAGTAATCTGTATATTCAGGCAAACTTC
AGCGAAGGTGAAACCTGGGAAAGCTTCAGCTTCAACTTCGATCAGGAAACCATTCCGAGCGATGTTCTGGTGGCCGGCGGTTA
TGATCAGATTATGATTGTTCTGGCAAGCGGTGATACCAGTGGTCTGACCAGTACCTATTATATTGATACCATTAGTGGTAGCTTC
GAACAGCCGCTGCGCAATGCACTGTTCCTGAGTGGTAGTTGGGGTGTTCGCTTCAATCTGAATGGTGGTTATCGTCTGGATAAT
AGTAGCAATGATGATTGGGTTGCCGGCGCACAGAATATTGTGGATAATCTGCCGGCCGTGGGTCATGTGATTACCAACTTCACC
CATCCGGCACATGGTTATTATTATACCCTGCGCGATAATCCGTATGTGGATATTGCAAATGAAATTCATCCGGATATGGTGCCGA
CCTTCGAAAATGAACAGATTATTCTGGATGTGATTGATGTGTTCAAAAATAGCGGTAAAAAGGTTATTCTGTACCTGAATGGTGC
AGGTCCGGGCCATCTGCAGGGTAATAGCGATCGTGAATTCGAAATTCTGGCCGCATGGGAAAATTATTATACCACCGAATTCGC
CGGCGATGAAGCACTGGCCTGGCGTACCCTGGTGAAAGGCTATCTGGAACGCTTCCAGGGTCTGGTTGATGGCTATTGGCTG
GATAATCTGGGCAATCTGCCGGGCGAACTGAGCGACTTCATTGCCATGATTCGCGAAGTTGATCCGAATATTGCCATTGCCACC
AATGGCAATAGCACCTATCTGACCGATGAAAATGGTGATGTGCTGTATGTGGATACCGATGCCACCGATGATGAAGATCCGCG
CGATTATCGTATTCGCAACTTCGTGATTAATGATCCGTATATGGACTTCACCGCAGGCCATCCGACCCCGCTGGGTCAGGGTGC
ACCGCCTAATAGTTGGGCATATGAAGAATTCATCTTCCCGCTGATTGCCGAAAGCCCGTGGGGCACCTTCGATGGTAATAAAGA
TGCAATTAAACACTACTTCTGCCCGATTCGCGATCGTTGGAGTGTTGCAAGCGCCGATCTGGTGTTCGAAACCGAACAGGCCTA
TCGCTTCGTTCGTACCCTGACCGATGTGGGTGCAGCAATTACCTATAGCACCACCATTACCGATGGCAGTATTACCGATGATGA
GATGGCAATTATGGAAGCCATTAATGATCGCATTGTTCAGACCCCGAAACCGGATTATATTCCGTATGTGCGTCCGGAAGGTGC
ATATCTGGTTGGCGAAATTCTGAGCGTTGATAGTAATTAA // **XhoI**

Vectors harboring GH107 constructs were transformed into *E. coli* BL21 (DE3) competent cells (New England Biolabs) and recombinant protein was produced as described.[3]

Briefly, *E. coli* BL21 (DE3) transformants with pET28a expression plasmids were inoculated in LB medium supplemented with 50 µg・mL−1 kanamycin, and incubated at 37°C, 150 rpm until an OD600 = 0.6–0.8, and induced by addition of isopropyl β-D-1-thiogalactopyranoside (IPTG, 50 µM final concentration, 16°C, 24 h, 150 rpm). Cells were harvested by centrifugation (6000 x g, 40 min, 4°C) and cell pellets were stored at -20°C until further use. Following chemical cell lysis and centrifugation, the lysate was loaded

onto a 5 mL HiTrap IMAC column (GE Healthcare) using an Äkta Start Chromatography system (GE Healthcare) and eluted using 20 mM Tris-HCl, pH 8; 0.5 M NaCl with increasing imidazole concentrations. IMAC fractions containing GH107 were pooled and concentrated on an Amicon stirred cell (Merck) equipped with a 30 kDa membrane. Imidazole was removed from the concentrated enzyme using a HiTrap Desalting column (Cytiva) equilibrated with 20 mM Tris-HCl, pH 8; 0.5 M NaCl.

Enzyme reactions

Fucoidan from *Laminaria hyperborea* (PROTASEA®, obtained from IFF, Norway) was purified using anion exchange chromatography as previously described.[4]

Purified *Laminaria hyperborea* fucoidan and synthetic oligosaccharide **10** were digested with IMAC purified GH107. Enzyme reactions contained fucoidan or oligosaccharide (0.4 mg/mL) and 1 μM enzyme in 25 mM Bis-Tris, pH=6.5; 250 mM NaCl; 1% seasalts (Sigma). Reactions were incubated for 1 and 24 hours and stopped by heating the samples at 98°C for 15 min. Control digests contained GH107 enzyme that had been heat inactivated at 98°C for 15 min prior to addition of substrate. Enzyme activity was visualized by Carbohydrate Polyacrylamide Gel Electrophoresis (CPAGE) as previously described.[5]

Polysaccharide microarray analysis of *Thalassiosira weissflogii* extracts

Thalassiosira weissflogii diatom cultures were grown in ESAW media as previously described.[6] Non-axenic monospecific cultures were harvested 10 days after inoculation, by centrifugation at 6800 x g for 20 min at 15 °C. Subsequently, diatom biomass (cell pellets) was collected, freeze dried, homogenized with a pestle, and alcohol precipitation was performed to obtain the alcohol-insoluble residue (AIR) enriched in polysaccharides. About 10 mg of diatom biomass were weighed out into 8-strip tubes in triplicates, followed by the addition of 300 µL of autoclaved MilliQ water per each 10 mg of sample. Polysaccharide extraction was carried out using a TissueLyser II (Qiagen, Germany), with samples shaken at 30 oscillations/s for 2 min followed by 2 h at 6 oscillations/s. Samples were spun down at 6000 × g for 15 min at 15 °C.

The *T. weissflogii* water extracts (supernatants) were printed in duplicates onto nitrocellulose membrane (pore size of 0.45 µm, Whatman, UK) using a piezoelectric microarrayer (Sprint, Arrayjet, UK). Microarray printing, probing and analysis was performed as previously described.[6] Briefly, microarrays were blocked in phosphate buffered saline (PBS, 1x) with 5% (w/v) non-fat milk powder (MPBS) for 1 h. Thereafter, microarrays were incubated with monoclonal antibodies (SeaProbes, France; PlantProbes, UK) diluted 1:10 in MPBS for 2 h, washed in PBS and then incubated for 2 h with an anti-rat secondary antibody conjugated to alkaline phosphatase diluted 1:5000 in MPBS. Arrays were washed in PBS and MilliQ water, developed with alkaline phosphatase substrates and the antibody signal was quantified using the software Array-Pro Analyzer 6.3 (Media Cybernetics). Normalization was performed by setting the highest mean signal intensity in the dataset to 100, with all other values adjusted accordingly.

Immunofluorescence microscopy

Laboratory cultures of *T. weissflogii* were grown as previously described.[6] At 10 days after inoculation, 15 mL of a culture were fixed using 37% formaldehyde to a final concentration of 2% (v/v) for 1 h at room temperature. Then, 4 mL of the fixed diatom culture were filtered onto polycarbonate filters with 0.2 μm pore size and 25 mm diameter (GTTP, Merck Millipore) at a pressure of less than 200 mbar with a vacuum pump. Filters were let to air-dry and afterwards were cut into sections and immunolabeled as previously described.[6] In brief, the filter sections were blocked in PBS with 3% (w/v) bovine serum albumin (BSA-PBS) for 1 h. Next, they were incubated with the monoclonal antibody BAM2 (SeaProbes, France) diluted 1:5 in BSA-PBS for 1.5 h. After a washing step with PBS, they were incubated with an anti-rat secondary antibody conjugated to FITC (F1763, Sigma-Aldrich) diluted 1:100 in BSA-PBS for 1.5 h in darkness. Sections were washed in PBS and stained with DAPI at 1 μg/mL for 10 min. Immunolabelling of samples was performed three times independently, yielding consistent results.

The immunolabelled filters were examined on a Zeiss Axio Imager D2 epifluorescence microscope equipped with an AxioCam MRm camera (Carl Zeiss, Germany). Image acquisition was conducted using Zeiss AxioVision software.

Synthetic glycan microarray printing

Amine equipped oligosaccharides (0.1 mM glycan in 50 mM PBS, pH 8.5) were immobilized on commercial *N*-hydroxysuccinimide (NHS) ester-activated microarray slides (CodeLink Activated Slides; SurModics) using a piezoelectric microarray spotting device (S3 Scienion) in 64 identical subarrays on each slide. Slides were incubated in a humid chamber at room temperature for 16 hours. The remaining NHS-esters on the slides were quenched with ethanolamine (100 mM) in sodium phosphate buffer (50 mM, pH 9) for 1 h at room temperature. Slides were washed three times with deionized water, dried by centrifugation (300 x g, 5 min) and stored at -20 °C in vacuum sealed plastic bag until use.

Synthetic glycan microarray scanning

The microarray slides containing the immobilized oligosaccharides was first blocked with 3% bovine serum albumin in PBS for 1 hour at room temperature. The slides were then washed three times with PBS and dried by centrifugation. The BAM monoclonal antibodies (BAM1-4) were prepared in dilutions of 1:2, 1:10, 1:50 and 1:100 in 3% BSA-PBS. A FlexWell 64 grid was applied and each antibody dilution was placed in a well. The array was stored in a humid chamber for 1 h at room temperature. Each well was then washed three times with 0.1 % PBS-Tween. Post-washing the slides were incubated with a fluorescence-labelled secondary antibody (anti-rat IgG FITC) that was 1:400 diluted in in 3% BSA-PBS. The slide was stored in a light-protected humidity chamber for 60 minutes at room temperature. The slides were washed three times with PBS-Tween and the grids were removed. In a Petri dish, the slide was washed with PBS-Tween for 10 minutes and rinsed twice with deionized water. To remove the remaining liquid, the slides were dried by centrifugation (300 x g, 5 min). The slides were scanned with a GenePix 4300A microarray scanner (Molecular Devices, Sunnyvale, CA, USA). Image analysis was carried out using GenePix Pro 7 software (Molecular Devices).

Building block synthesis

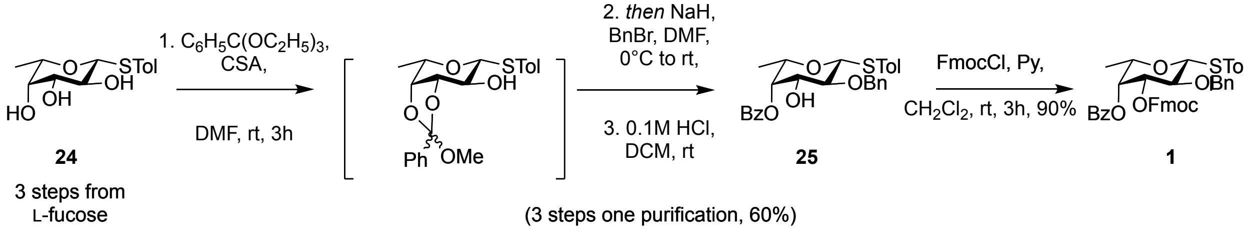

SI Scheme 1. Synthesis of building block **1**.

4-Methylphenyl 1-thio-β-L-fucopyranoside (24)

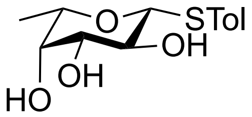

L-Fucose (4 g, 15.7mmol) was suspended in CH_2Cl_2 (0.1M, 160 mL), pyridine (13 ml, 157 7mmol, 10eq.), and acetic acid (7.4 mL, 78.5 mmol, 5 eq.). Subsequently, a catalytic quantity of 4-(dimethylamino)pyridine (20 mg, 0.157 mmol, 0.01 eq.) was added. The mixture was left to stir at room temperature (≈16h) and then concentrated under vacuum to afford a thick yellow gel. To a solution of this crude product in CH_2Cl_2 (0.05M, 300 mL), 4-methoxythiophenol (4 mL, 33 mmol, 2.1 eq.) was added, followed by $BF_3{\cdot}Et_2O$ (6 mL, 47.1 mmol, 3 eq.) at 0°C, under an atmosphere of argon. The temperature was raised to room temperature and stirred until the reaction reached completion (≈16 h), as indicated by thin layer chromatography (TLC). The reaction was quenched by the addition of saturated aqueous $NaHCO_3$, extracted with CH_2Cl_2, washed with brine, dried over $MgSO_4$ and concentrated under vacuum. Purification by flash chromatography (SiO_2, hexane/ethyl acetate) afforded the 4-methylphenyl 2,3,4-*O*-acetyl-tri-1-thio-β-L-fucopyranoside intermediate, which was dissolved in CH_2Cl_2 (0.1M, 160 mL) and the pH was raised to pH 8 with a sodium methoxide (approx. 1 mL, 0.5M solution). Once complete, the reaction was quenched by the addition of Amberlite™ (Hydrogen foam), filtered, and concentrated under vacuum. Purification by flash chromatography (SiO_2, CH_2Cl_2/methanol) afforded **24** (3.3 g, 78%) as a white amorphous solid. R_f = 0.3 (CH_2Cl_2/methanol, 95:5, v/v).

^{1}H NMR (600 MHz, $CDCl_3$) δ 7.41 (d, *J* = 7.9 Hz, 2H), 7.06 (d, *J* = 7.9 Hz, 2H), 4.49 (d, *J* = 9.7 Hz, 1H), 3.81 (d, *J* = 3.0 Hz, 1H), 3.72 (t, *J* = 9.4 Hz, 1H), 3.67 – 3.62 (m, 1H), 3.57 (q, *J* = 6.5 Hz, 1H), 2.30 (s, 3H), 1.28 (d, *J* = 6.3 Hz, 3H). **^{13}C NMR** (151 MHz, $CDCl_3$) δ 137.9, 132.7, 129.8, 129.6, 89.1, 75.3, 74.8, 71.9, 69.8, 21.3, 16.8. **HRMS** QTOF-MS: calcd. $C_{13}H_{18}NaO_4S$ for $[M+Na]^+$ 293.0823, found 293.0829.

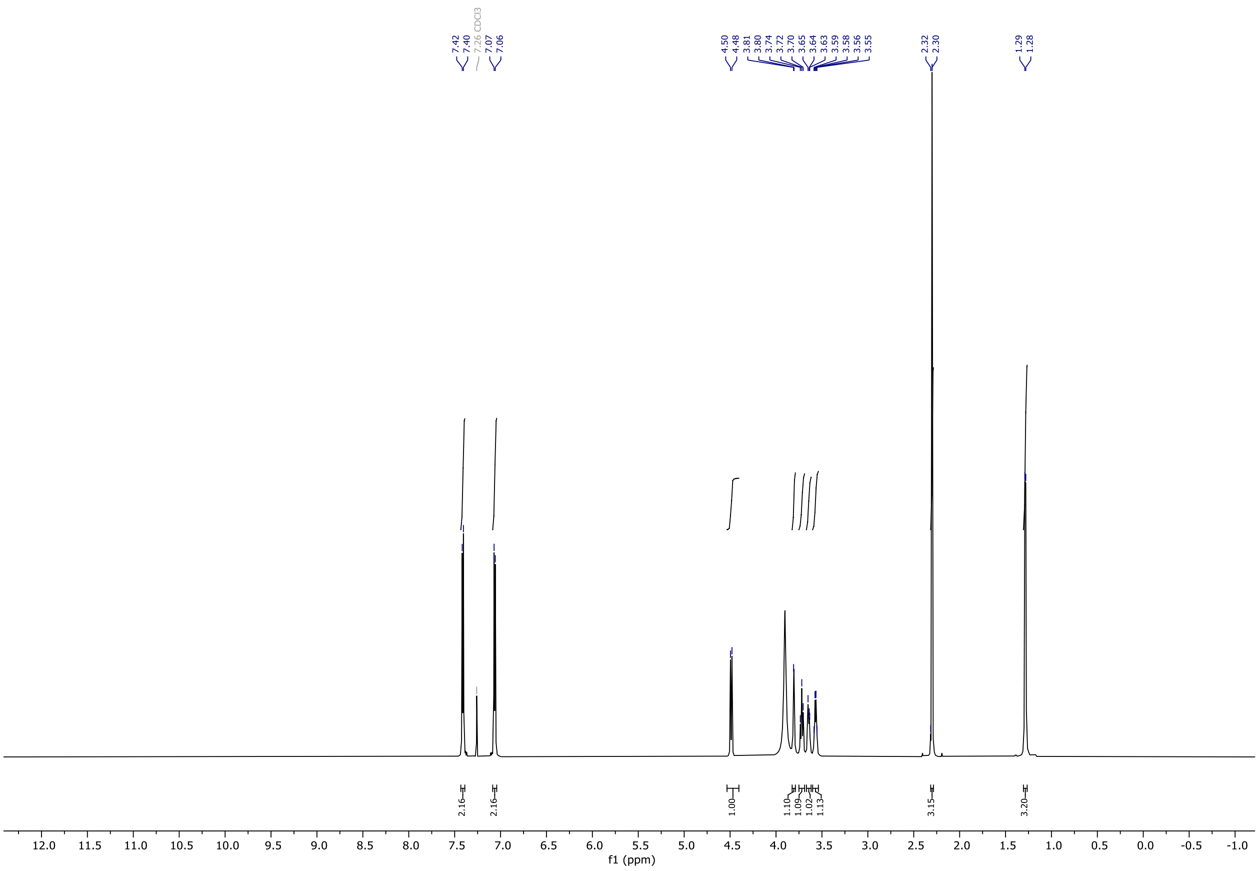

^{1}H NMR

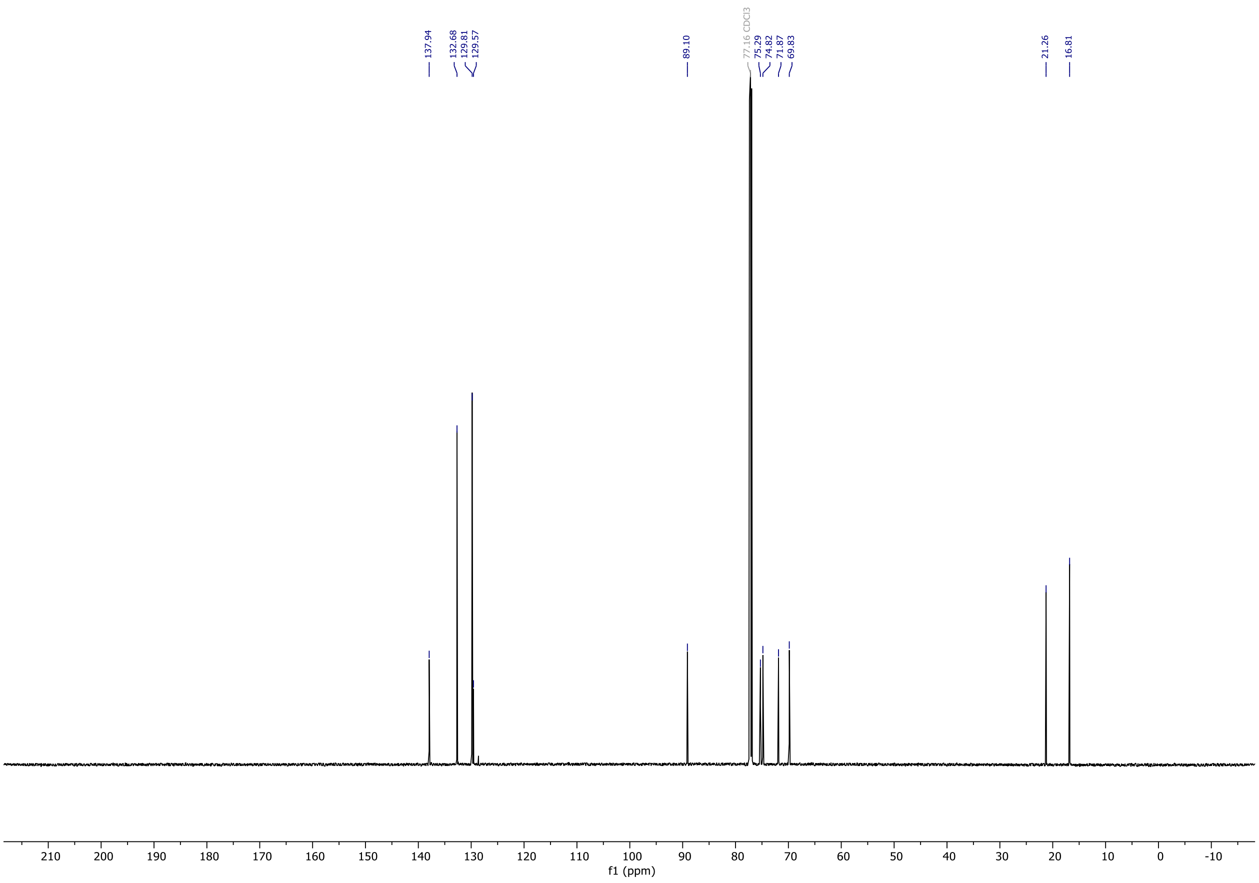
137.94
132.68
129.81
129.57
89.10
77.16 CDCl3
75.29
74.82
71.87
69.83
21.26
16.81
f1 (ppm)

^{13}C NMR

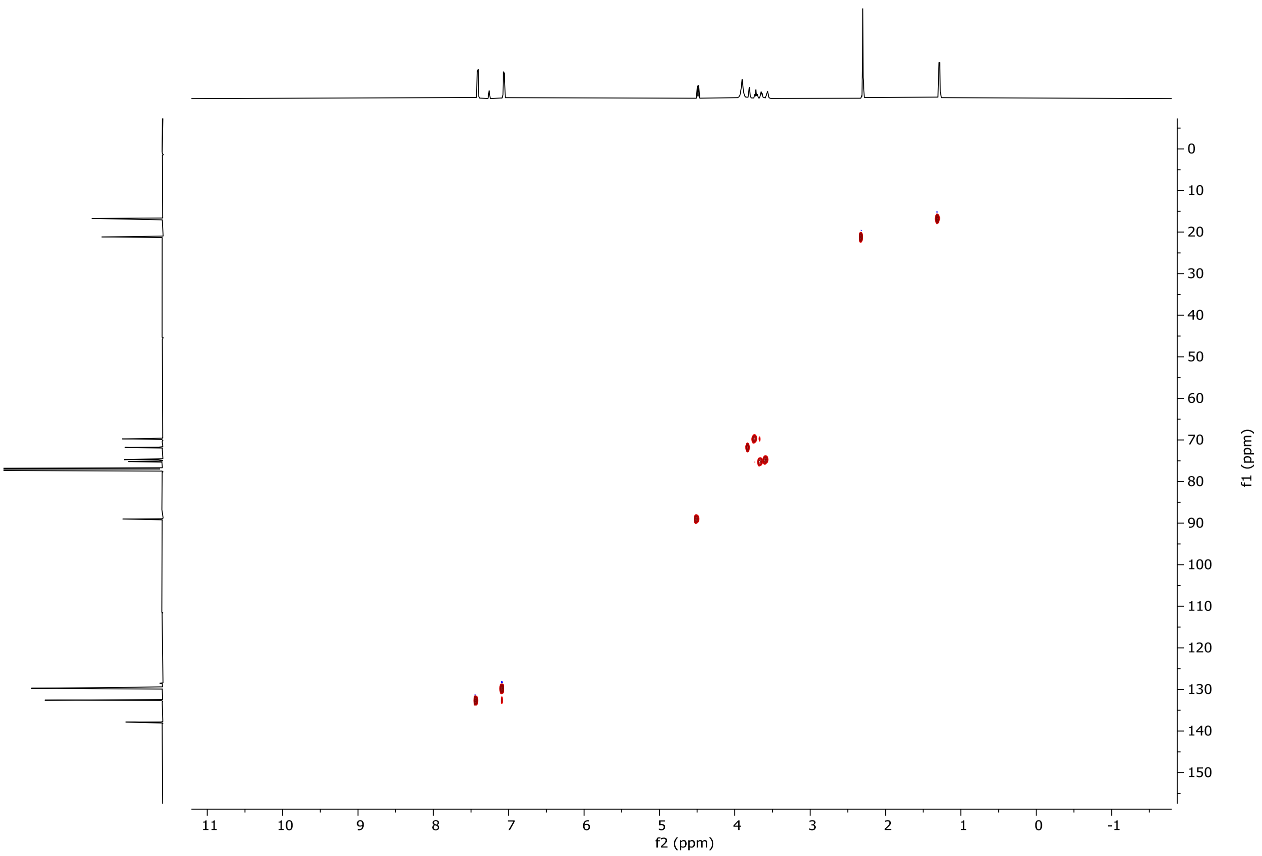

^{1}H-^{13}C HSQC NMR

4-Methylphenyl 4-*O*-benzoyl-2-*O*-benzyl-1-thio-β-L-fucopyranoside (25)

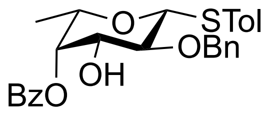

4-Methylphenyl 1-thio-β-L-fucopyranoside **24** (8.7 g, 32.2 mmol) and triethyl ortho-benzoate (8.4 mL, 48.3 mmol, 1.5 eq.) were dissolved in CH_2Cl_2 (0.1M, 320 mL), and camphorsulfonic acid (3 g, 12.9 mmol, 0.4 eq.) was added. The mixture was stirred at room temperature under a nitrogen atmosphere until the reaction reached completion (≈1 h). The reaction was then neutralized with Et_3N (2.2 mL, 16.1 mmol, 0.5 eq.) and concentrated under vacuum. The crude material was re-dissolved in DMF (0.1M, 320 mL), cooled to 0 °C, and sodium hydride (60% dispersion, 1.5 g, 37.1 mmol, 1.2 eq.) was added. After 10 minutes, benzyl bromide (5.5 mL, 46.4 mmol, 1.5 eq.) was added slowly, and the reaction was allowed to raise to room temperature. Once the reaction reached completion (≈2 h), the reaction was quenched by the addition of methanol and left to stir for 20 minutes. The reaction mixture was then concentrated under vacuum. The residue was then re-dissolved in CH_2Cl_2 and successively washed with saturated aqueous $NaHCO_3$, brine, dried over $MgSO_4$, and concentrated under vacuum. Subsequently, the crude material was dissolved in CH_2Cl_2 and stirred with 0.1M HCl solution. Once the reaction reached completion, it was extracted, and washed with saturated aqueous $NaHCO_3$, dried over $MgSO_4$, and concentrated. The residue was purified by flash column chromatography (SiO_2, Hexane/ Ethyl acetate) to give compound **25** (8.9 g, 60% over three steps) as a solid. R_f = 0.4 (Hexane/Ethyl acetate, 70:30, v/v).

^{1}H NMR (400 MHz, $CDCl_3$) δ 7.99 – 7.92 (m, 2H), 7.58 – 7.46 (m, 3H), 7.39 (t, *J* = 7.7 Hz, 2H), 7.34 – 7.17 (m, 6H), 7.09 (d, *J* = 8.0 Hz, 2H), 5.35 – 5.28 (m, 1H), 4.86 (d, *J* = 10.8 Hz, 1H), 4.65 – 4.50 (m, 2H), 3.83 (dd, *J* = 9.1, 3.4 Hz, 1H), 3.80 – 3.69 (m, 1H), 3.55 (t, *J* = 9.4 Hz, 1H), 2.31 (s, 3H), 1.20 (d, *J* = 6.4 Hz, 3H). **^{13}C NMR** 13C NMR (101 MHz, CDCl3) δ 166.8, 138.1, 138.0, 133.5, 133.1, 130.2, 129.9, 129.6, 129.5, 128.7, 128.5, 128.4, 128.2, 87.1, 75.4, 74.4, 73.6, 73.5, 21.4, 17.0. **HRMS** QTOF-MS: calcd. $C_{27}H_{28}NaO_5S$ for $[M+Na]^+$ 487.1555, found 487.1558.

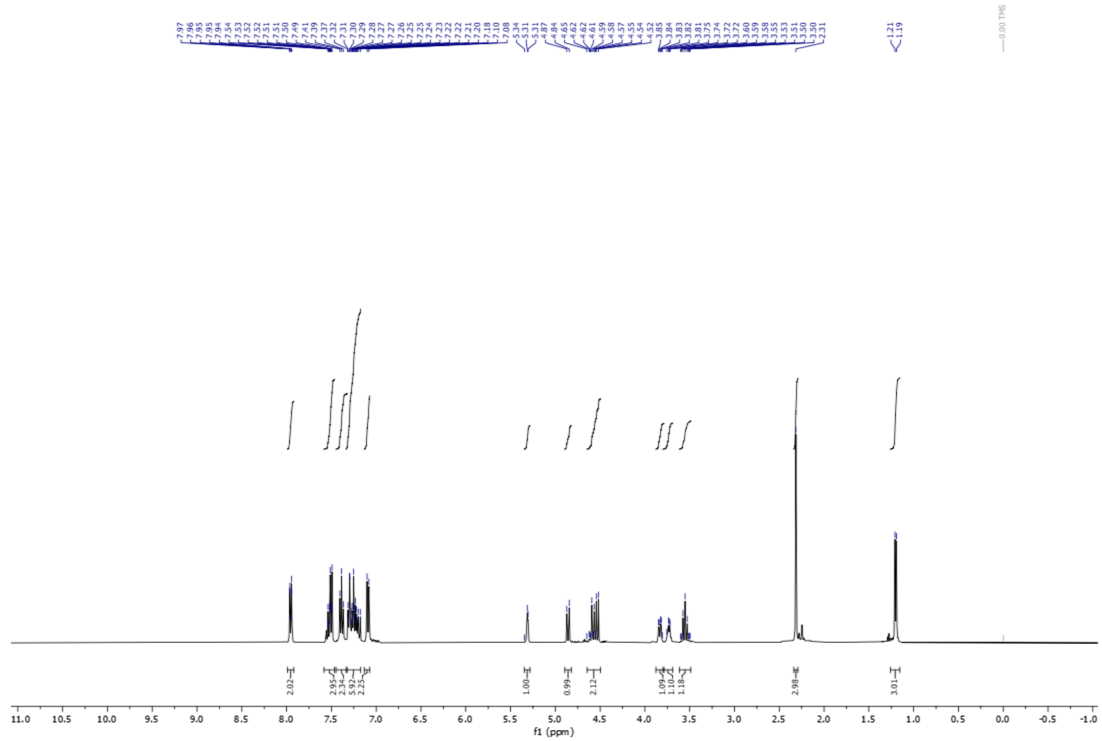

^{1}H NMR

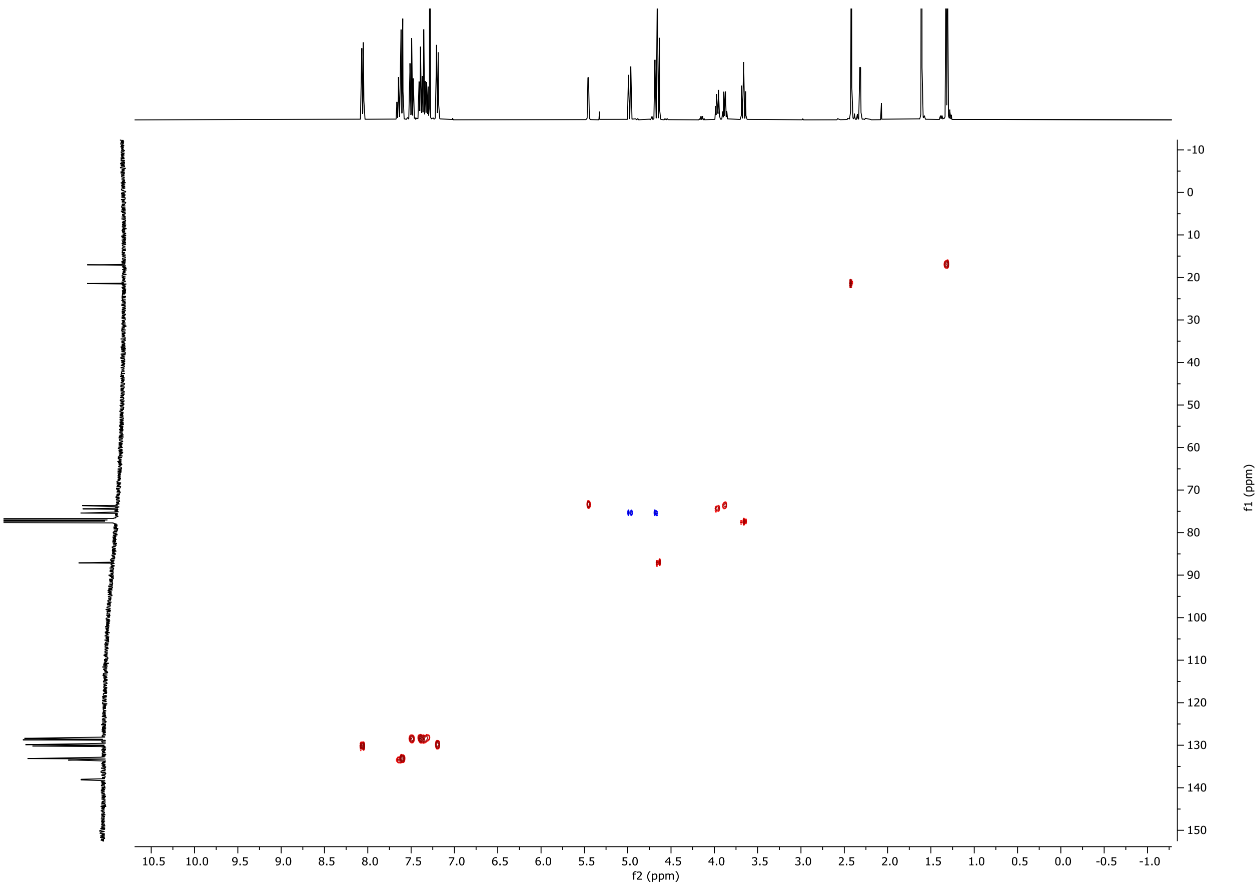

^{1}H-^{13}C HSQC NMR

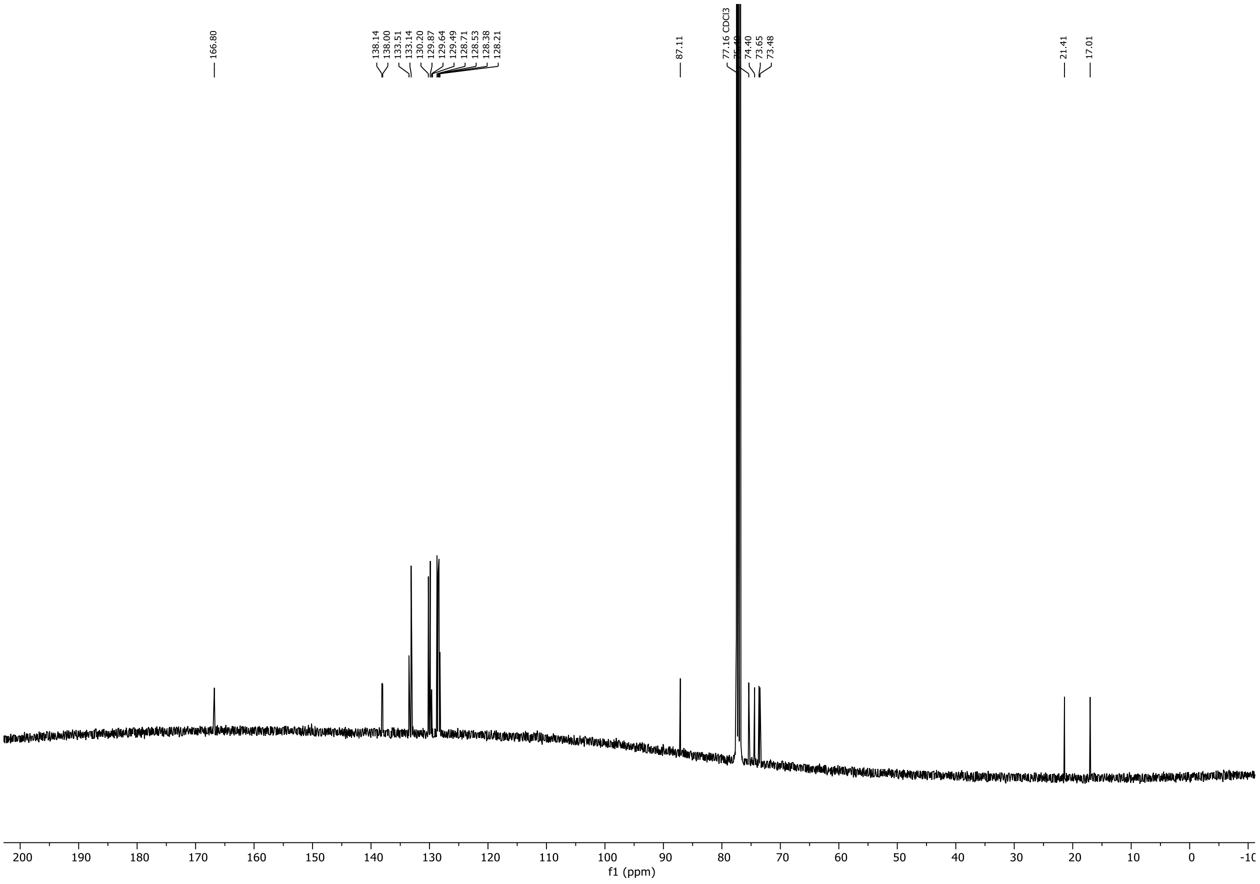
166.80
138.14
138.00
133.51
133.14
130.20
129.87
129.64
129.49
128.71
128.53
128.38
128.21
87.11
77.16 CDCl3
74.40
73.65
73.48
21.41
17.01
f1 (ppm)

^{13}C NMR

4-Methylphenyl 4-*O*-benzoyl-2-*O*-benzyl-3-*O*-(9-fluorenylmethoxycarbonyl)-1-thio-β-L-fucopyranoside (1)

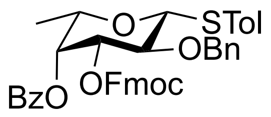

4-Methylphenyl 4-*O*-benzoyl-2-*O*-benzyl-1-thio-β-L-fucopyranoside **25** (3 g, 6.46 mmol) was dissolved in CH_2Cl_2 (0.1M, 65 mL) and pyridine (2 mL, 25.8 mmol, 4 eq.). Subsequently, 9-fluorenylmethoxycarbonylchloride (2 g, 7.75 mmol, 1.2 eq.) was added to the solution at room temperature under nitrogen. Once the reaction reached completion, as indicated by TLC, it was concentrated under vacuum and purified by flash chromatography (Hexane/EtOAc) to give compound **1** (3.9 g, 90%) as a solid. R_f = 0.35 (Hexane/EtOAc, 90:10, v/v).

^{1}H NMR (400 MHz, $CDCl_3$) δ 8.01 – 7.90 (m, 2H), 7.66 (d, *J* = 0.8 Hz, 2H), 7.60 – 7.50 (m, 3H), 7.48 – 7.39 (m, 5H), 7.33 – 7.25 (m, 5H), 7.25 – 7.15 (m, 5H), 7.14 – 7.03 (m, 3H), 5.56 (d, *J* = 1.0 Hz, 1H), 4.91 (dd, *J* = 9.6, 3.3 Hz, 1H), 4.81 – 4.72 (m, 1H), 4.66 – 4.53 (m, 2H), 4.47 (dd, *J* = 10.1, 6.4 Hz, 1H), 4.22 – 3.99 (m, 3H), 3.90 – 3.81 (m, 1H), 3.74 (t, *J* = 9.6 Hz, 1H), 2.33 (s, 3H), 1.23 (d, *J* = 6.4 Hz, 3H). **^{13}C NMR** (101 MHz, $CDCl_3$) δ 165.8, 154.1, 143.5, 142.8, 141.1, 141.0, 138.0, 137.7, 133.5, 133.3, 130.0, 129.6, 129.2, 128.5, 128.3, 128.2, 127.8, 127.7, 127.6, 127.6, 127.0, 126.9, 125.2, 124.9, 119.8, 119.7, 86.7, 74.4, 73.0, 70.8, 70.1, 46.4, 21.1, 16.6. **HRMS** QTOF-MS: calcd. $C_{42}H_{38}NaO_7S$ for $[M+Na]^+$ 709.2236, found 709.2240.

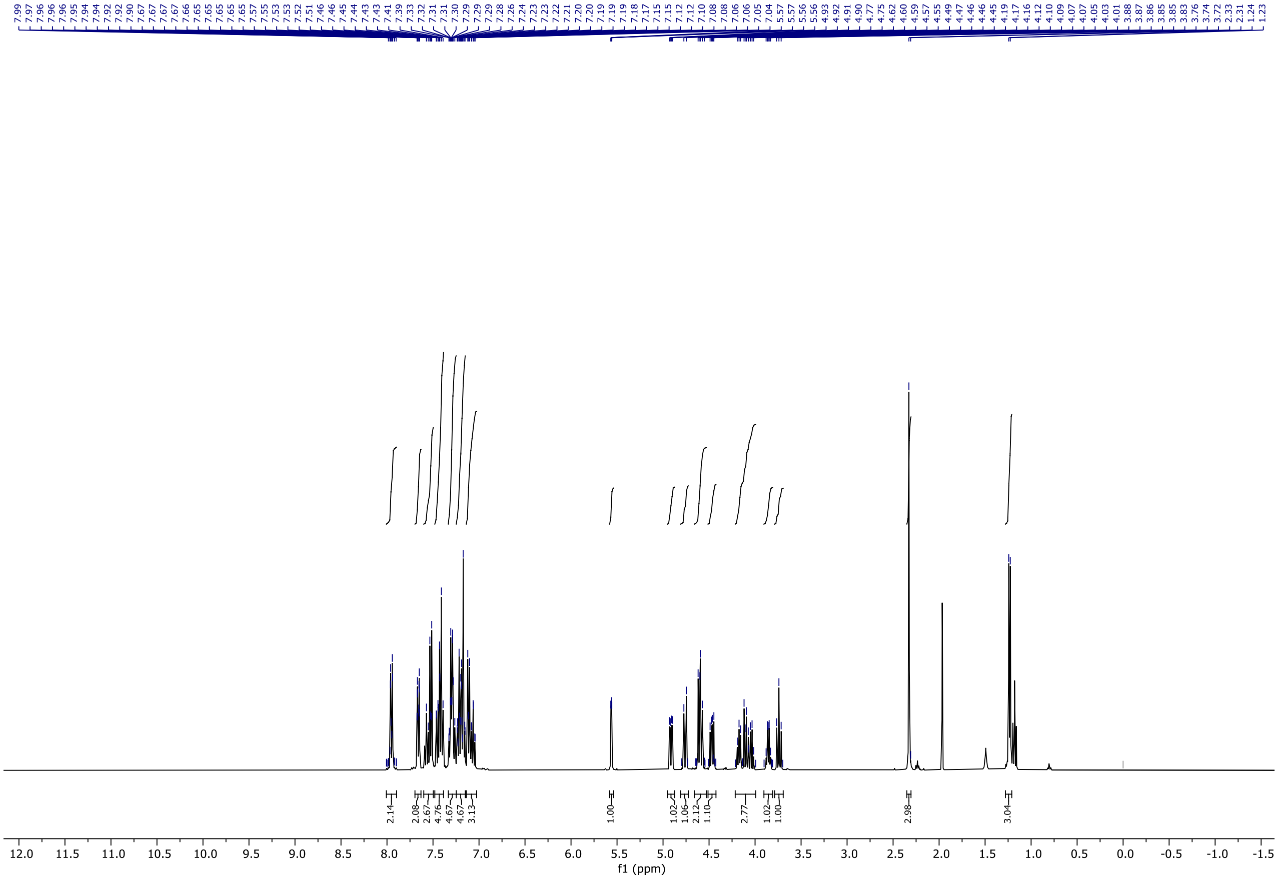

^{1}H NMR

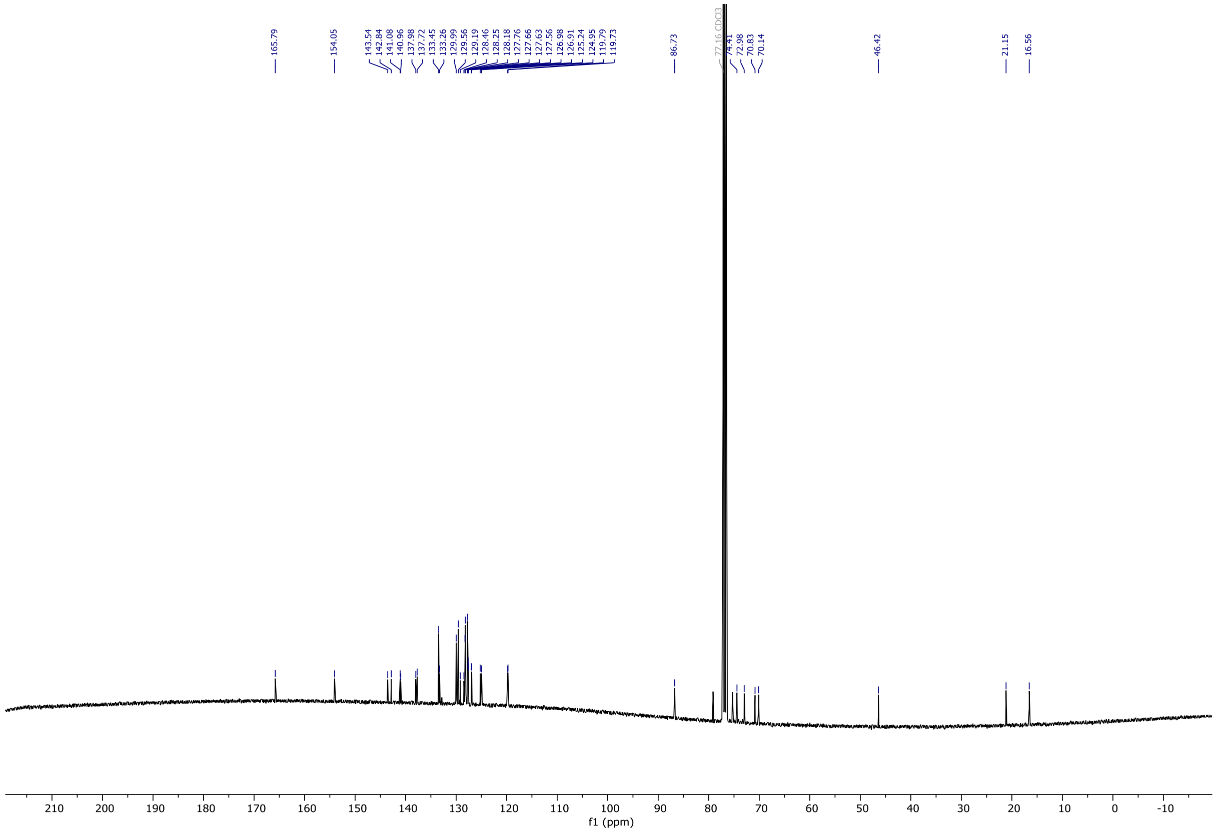

^{13}C NMR

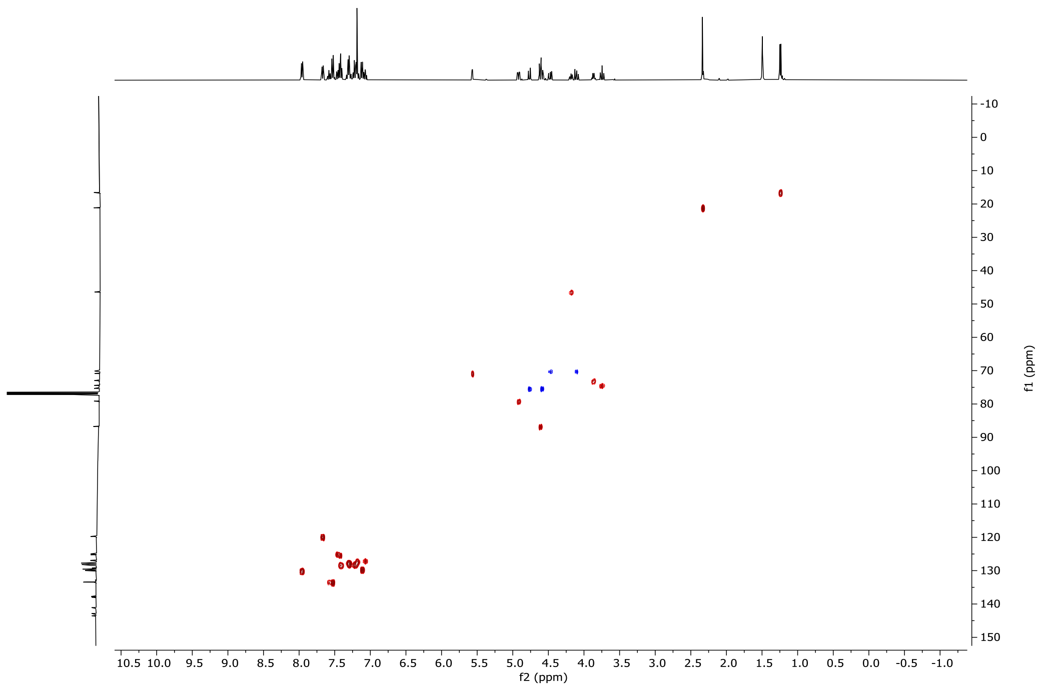

^{1}H-^{13}C HSQC NMR

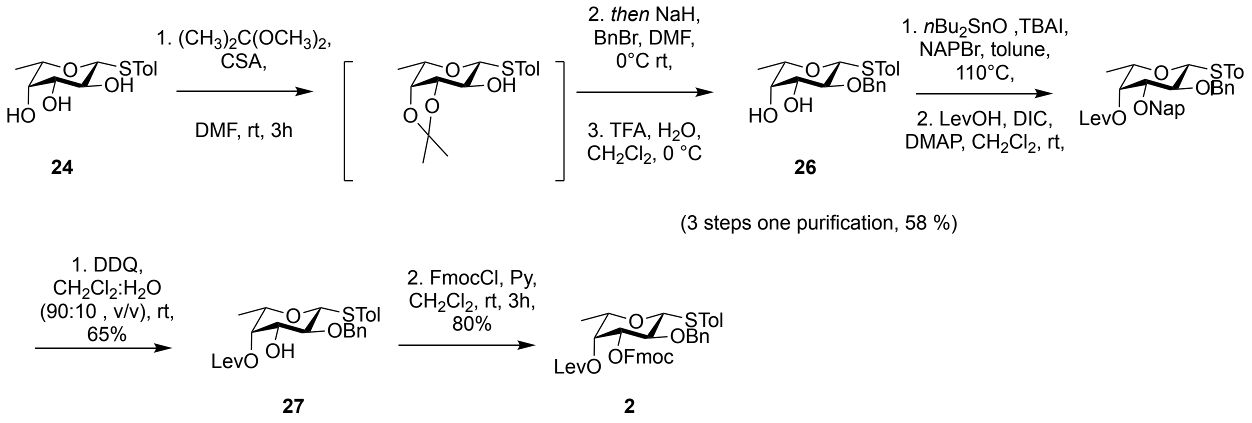

SI Scheme 2. Synthesis of building block **2**.

4-Methylphenyl 2-*O*-benzyl-1-thio-β-L-fucopyranoside (26)

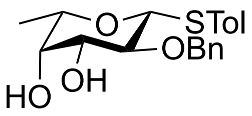

4-Methylphenyl 1-thio-β-L-fucopyranoside **24** (3 g, 11.8 mmol) was dissolved in CH_2Cl_2 (0.1M, 110 mL) under nitrogen. Subsequently, 2,2-dimethoxypropane (3 mL, 23.6 mmol, 2 eq.) and camphorsulfonic acid (0.5 g, 2.3 mmol, 0.2 eq.) were added. After ≈ 2 h, the reaction reached completion. The reaction was quenched with saturated aqueous $NaHCO_3$, the organic fraction was recovered, washed with brine, dried over $MgSO_4$, and concentrated. The crude material was re-dissolved in DMF (0.1M, 110 mL) and cooled to 0 °C. Then, sodium hydride (60% dispersion, 0.6 g, 14.2 mmol, 1.2 eq.) was added. After 10 minutes, benzyl bromide (2.8 mL, 23.6 mmol, 2 eq.) was added and the reaction was allowed to raise to room temperature. Once the reaction reached completion, as indicated by TLC, the reaction was quenched by the addition of methanol, and allowed to stir for 20 minutes. The reaction mixture was then concentrated under vacuum and purified by flash chromatography (SiO_2, Hexane/EtOAc, note: silica was pre-quenched with a solution of 1% NEt_3 in CH_2Cl_2). The intermediate 3,4-*O*-isopropylidene protected compound was then dissolved in CH_2Cl_2 (100mL), water (2 mL) and TFA (4 mL) at 0 °C. The reaction mixture was stirred for ≈ 4 h, and when complete, it was quenched by the addition of saturated aqueous $NaHCO_3$. The recovered organic fraction was then washed with brine, dried over $MgSO_4$, and concentrated. This residue was purified by flash chromatography (SiO_2, Hexane/EtOAc) to give compound **26** (2.3 g, 58%). R_f = 0.35 (Hexane/EtOAc, 50:50, v/v)

^{1}H NMR (400 MHz, $CDCl_3$) δ 7.52 – 7.44 (m, 2H), 7.44 – 7.27 (m, 5H), 7.13 (d, *J* = 7.9 Hz, 2H), 4.97 (d, *J* = 11.0 Hz, 1H), 4.69 (d, *J* = 11.0 Hz, 1H), 4.54 (d, *J* = 9.7 Hz, 1H), 3.74 (m, 1H), 3.68 – 3.57 (m, 2H), 3.50 (t, *J* = 9.3 Hz, 1H), 2.35 (s, 3H), 1.35 (d, *J* = 6.5 Hz, 3H). **^{13}C NMR** (101 MHz, $CDCl_3$) δ 138.2, 137.9, 132.6, 130.1, 129.9, 128.8, 128.5, 128.3, 87.8, 78.2, 75.4, 75.4, 74.5, 71.8, 21.3, 16.8. **HRMS** QTOF-MS: calcd. $C_{20}H_{24}NaO_4S$ for $[M+Na]^+$ 383.1293, found 383.1290.

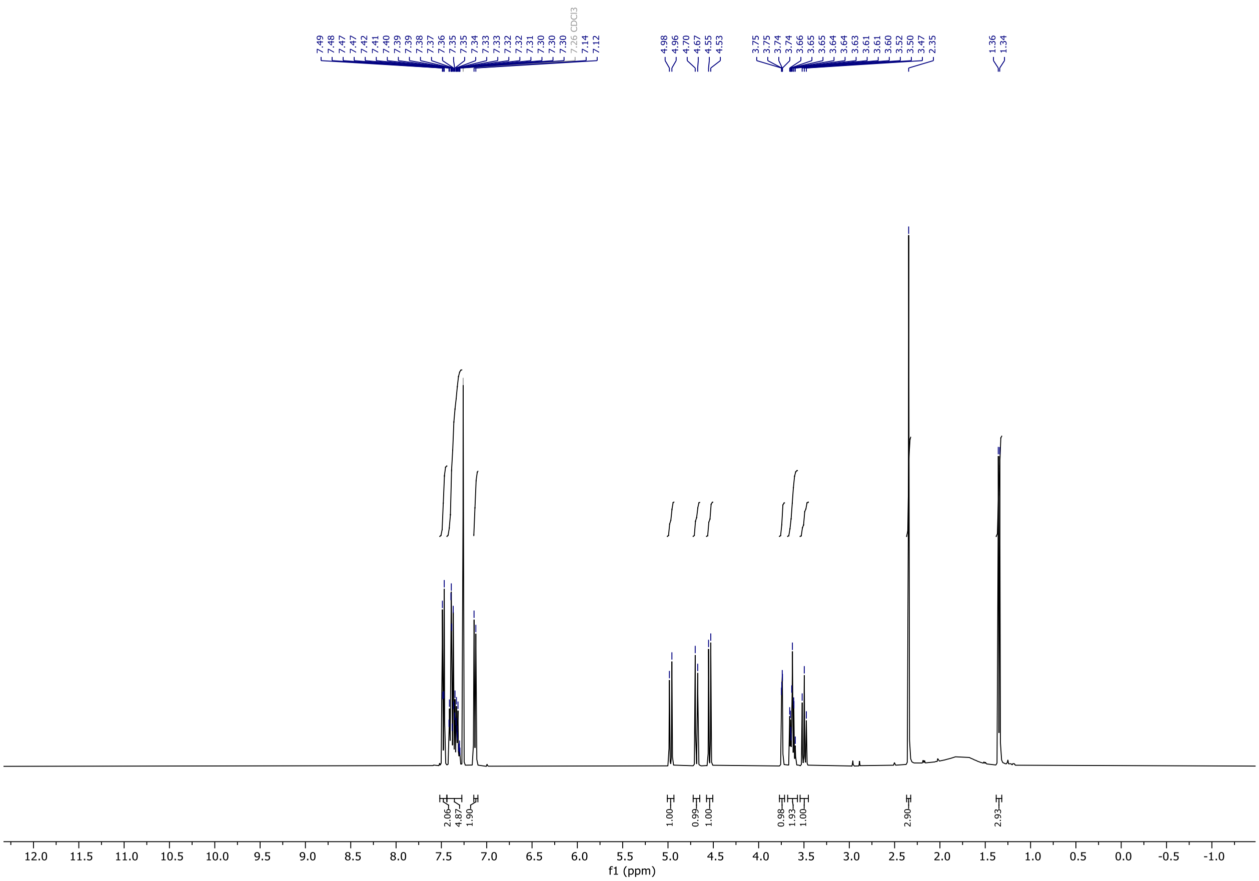
7.49
7.48
7.47
7.47
7.42
7.41
7.40
7.39
7.39
7.38
7.37
7.36
7.35
7.35
7.35
7.34
7.33
7.33
7.33
7.32
7.32
7.31
7.30
7.30
7.30
7.26 CDCl3
7.14
7.12
4.98
4.96
4.70
4.67
4.55
4.53
3.75
3.75
3.74
3.74
3.66
3.65
3.65
3.64
3.64
3.63
3.61
3.61
3.60
3.52
3.50
3.47
2.35
1.36
1.34
2.06
4.87
1.90
1.00
0.99
1.00
0.98
1.93
1.00
2.90
2.93
f1 (ppm)

^{1}H NMR

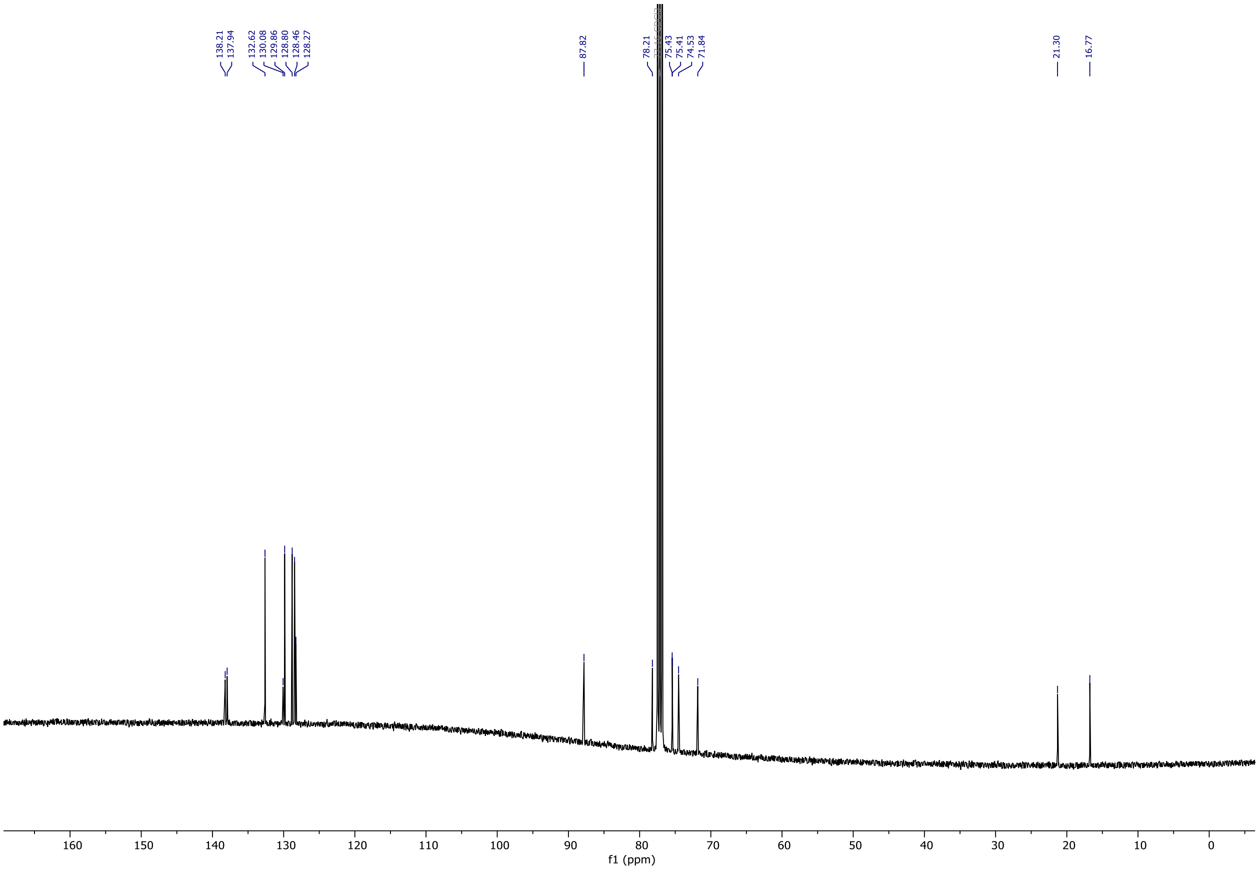
138.21
137.94
132.62
130.08
129.86
128.80
128.46
128.27
87.82
78.21
75.43
75.41
74.53
71.84
21.30
16.77
160
150
140
130
120
110
100
90
80
70
60
50
40
30
20
10
0
f1 (ppm)

^{13}C NMR

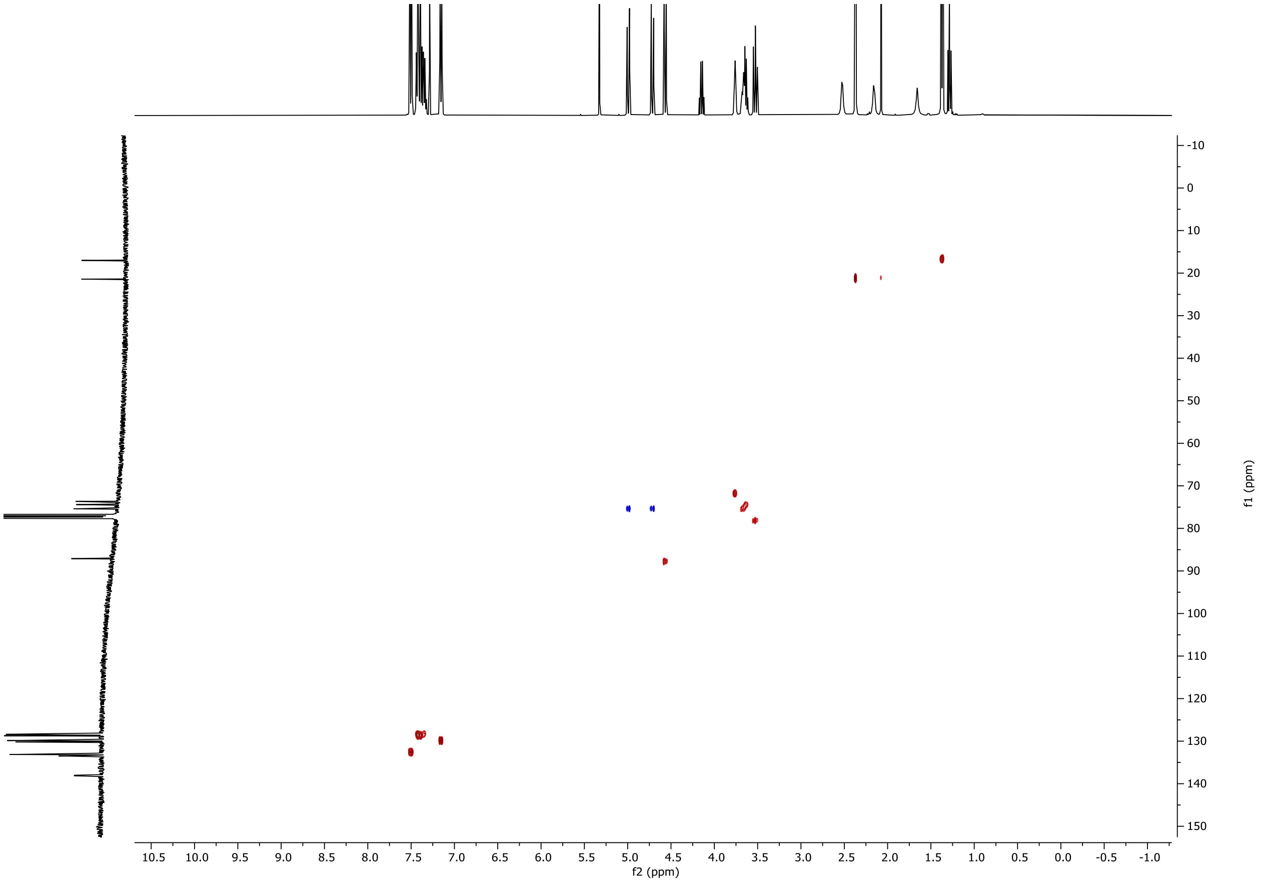

^{1}H-^{13}C HSQC NMR

4-Methylphenyl 2-*O*-benzyl-4-*O*-levulinyl-1-thio-β-L-fucopyranoside (27)

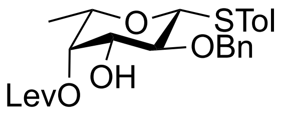

4-Methylphenyl 2-*O*-benzyl-1-thio-β-L-fucopyranoside **26** (2.3 g, 6.38 mmol) was dissolved in anhydrous toluene (0.1M, 65 mL), *n*Bu_2SnO (1.9 g, 7.66 mmol, 1.2 eq.) was added, and the reaction was left to reflux overnight. Subsequently, the reaction was brought to 40 °C. Thereafter, 2-naphthylmethyl bromide (2.8 g, 12.8 mmol, 3 eq.) and TBAI (0.2 g, 0.63 mmol, 0.1 eq.) was added. Once the reaction was complete, the solution was concentrated under vacuum and purified by flash chromatography (SiO_2, hexane/EtOAc). Next, the 4-methylphenyl 2-*O*-benzyl-3-*O*-(2-naphthalenylmethyl)-1-thio-β-L-fucopyranoside intermediate was dissolved in anhydrous CH_2Cl_2 (0.1M, 65 mL). Then, levulinic acid (1.2 mL, 12.8 mmol, 2.0 equiv.), DIC (3 mL, 19.1 mmol, 3.0 equiv.), and DMAP (70 mg, 0.63 mmol, 0.1 equiv.) were added. The reaction was stirred at room temperature under nitrogen. Once the reaction reached completion, it was filtered through a pad of Celite, the organic layer was diluted in CH_2Cl_2 and washed successively with saturated aqueous $NaHCO_3$, brine, dried over $MgSO_4$, and concentrated under vacuum. The crude material was purified by flash chromatography (SiO_2, Hexane/EtOAc). The 4-methylphenyl 2-*O*-benzyl-3-*O*-(2-naphthalenylmethyl)-4-*O*-levulinyl-1-thio-β-L-fucopyranoside intermediate was dissolved in CH_2Cl_2/H_2O (0.05M, 90:10, v/v, 130 mL) at room temperature. Subsequently, 2,3-dichloro-5,6-dicyano-1,4-benzoquinone (DDQ) (1.7 g, 7.6 mmol, 1.2 eq.) was added to the vigorously stirred solution, and it was kept in the dark. Once the reaction reached completion, it was quenched by the addition of a 1:1 solution of 10% aq. $Na_2S_2O_3$ and $NaHCO_3$ (120 mL). The resulting mixture stirred for 10 minutes, then the organic layer was washed sequentially with saturated aqueous $NaHCO_3$, brine (1 x 100 mL), dried over $MgSO_4$, and concentrated under vacuum. The residue was purified by flash column chromatography (SiO_2, hexane/ethyl acetate) to give the compound **27** (1.9 g, 65%). R_f = 0.35 (Hexane/EtOAc, 50:50, v/v).

^{1}H NMR (400 MHz, $CDCl_3$) δ 7.49 (d, *J* = 8.2 Hz, 2H), 7.46 – 7.41 (m, 2H), 7.40 – 7.28 (m, 3H), 7.12 (d, *J* = 8.0 Hz, 2H), 5.21 (dd, *J* = 3.5, 1.1 Hz, 1H), 4.92 (d, *J* = 10.7 Hz, 1H), 4.74 (d, *J* = 10.7 Hz, 1H), 4.58 (d, *J* = 9.6 Hz, 1H), 3.86 – 3.78 (m, 1H), 3.76 – 3.67 (m, 1H), 3.51 (t, *J* = 9.4 Hz, 1H), 2.87 – 2.78 (m, 2H), 2.74 – 2.61 (m, 2H), 2.35 (s, 3H), 2.20 (s, 3H), 1.22 (d, *J* = 6.4 Hz, 3H). **^{13}C NMR** (101 MHz, $CDCl_3$) δ 207.6, 173.0, 138.2, 137.9, 132.6, 129.8, 128.7, 128.4, 128.1, 87.9, 78.3, 75.6, 74.4, 73.4, 73.3, 38.5, 30.0, 28.2, 21.3, 16.9. **HRMS** QTOF-MS: calcd. $C_{25}H_{30}NaO_6S$ for $[M+Na]^+$ 481.1661, found 481.1665.

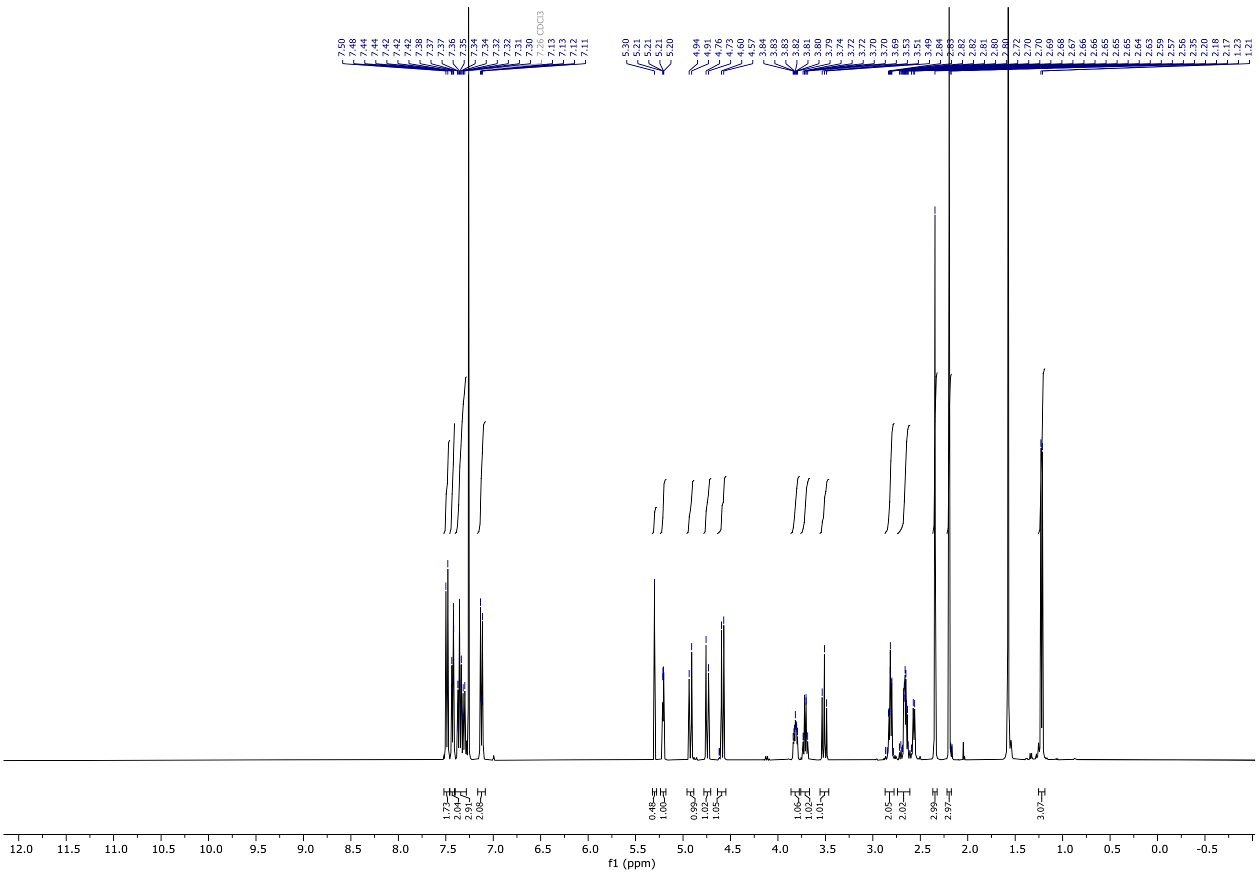

^{1}H NMR

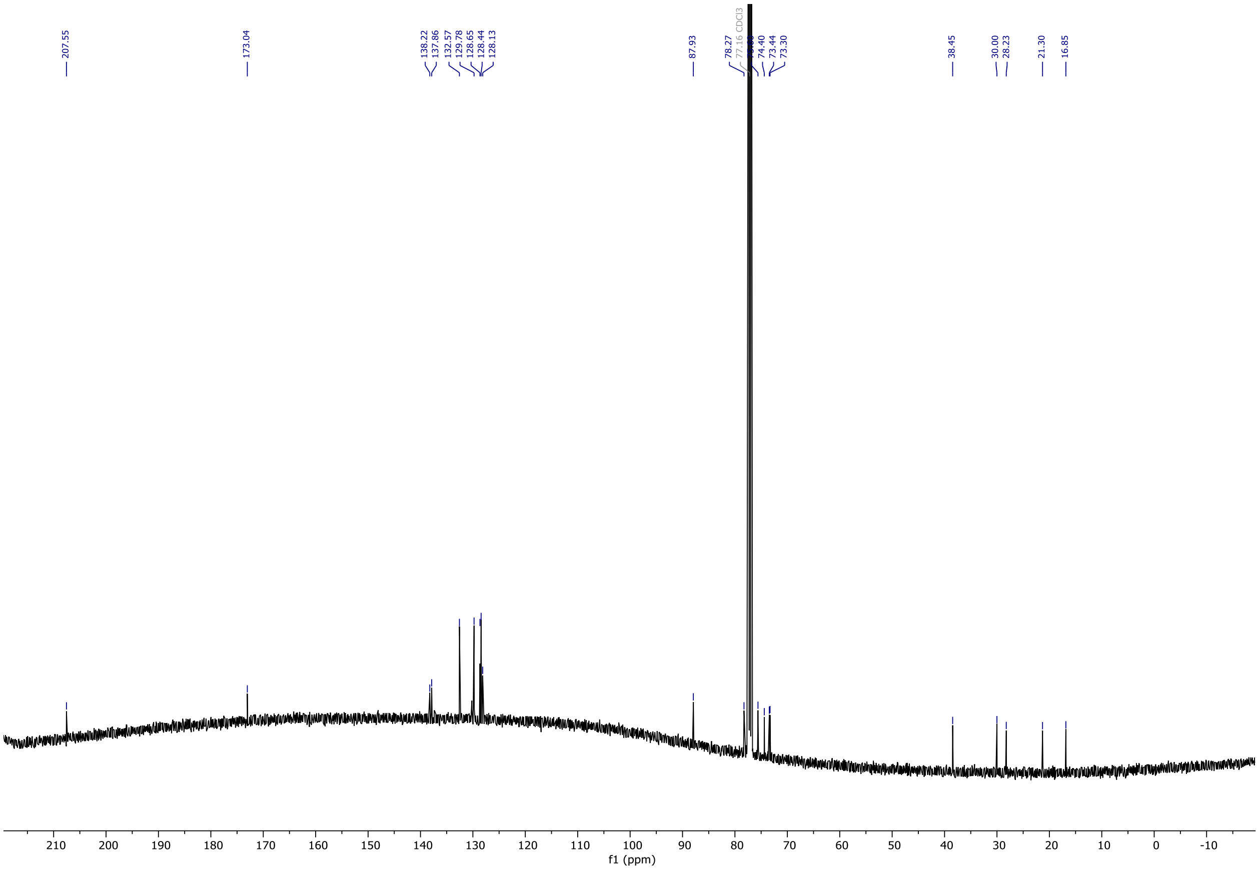
207.55
173.04
138.22
137.86
132.57
129.78
128.65
128.44
128.13
87.93
78.27
77.16 CDCl3
74.40
73.44
73.30
38.45
30.00
28.23
21.30
16.85
f1 (ppm)

^{13}C NMR

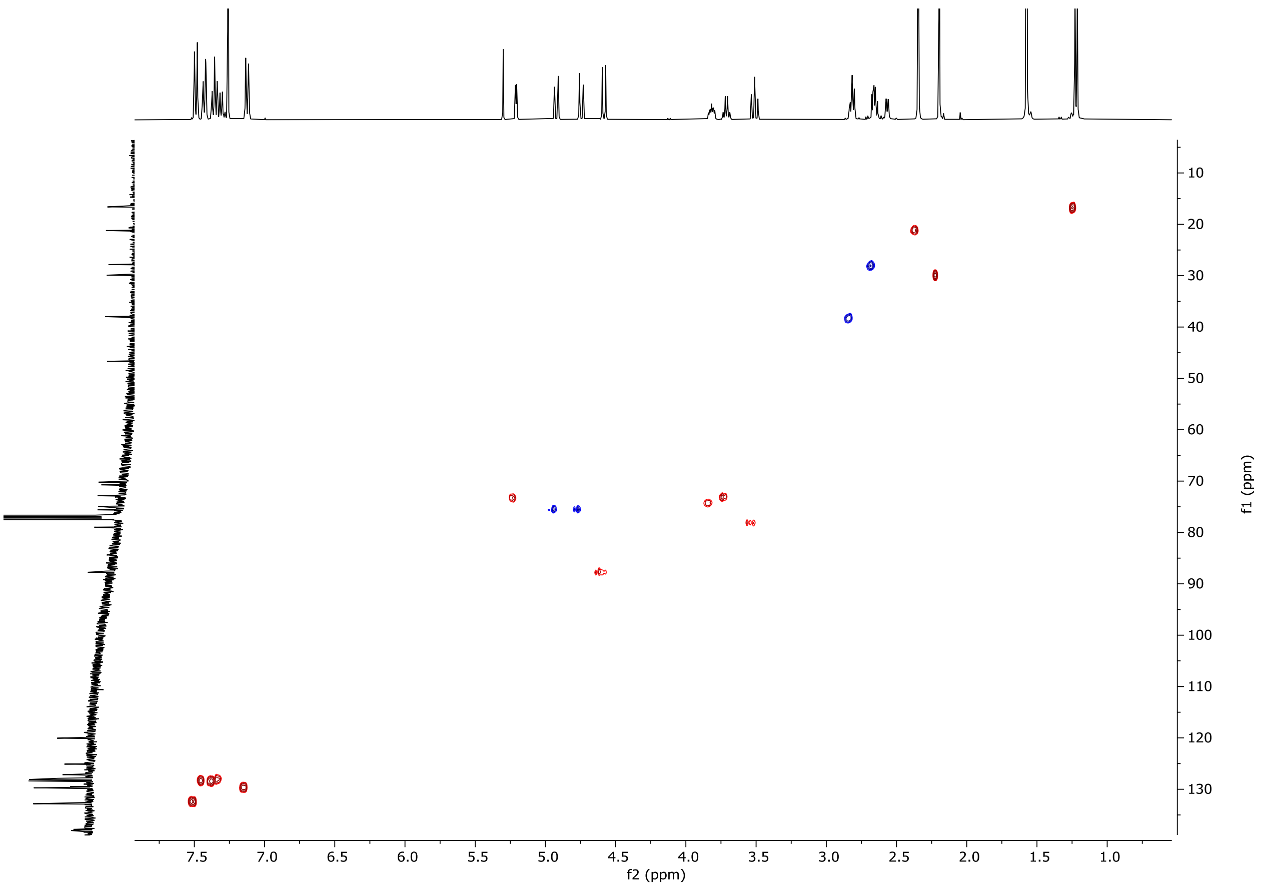

^{1}H-^{13}C HSQC NMR

4-Methylphenyl 2-*O*-benzyl-3-*O*-(9-fluorenylmethoxycarbonyl)-4-*O*-levulinyl-1-thio- β-L-fucopyranoside (2)

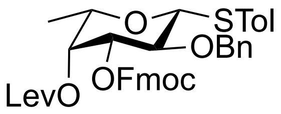

4-Methylphenyl 4-*O*-levulinyl-2-*O*-benzyl-1-thio-β-L-fucopyranoside **27** (1.9 g, 4.14 mmol) was dissolved in CH_2Cl_2 (0.1M, 40 mL), pyridine (1.3 mL, 16.6 mmol, 4 eq.), and 9-fluorenylmethoxycarbonylchloride (Fmoc-Cl) (1.29 g, 4.97 mmol, 1.2 eq.) was added. The mixture was stirred at room temperature until the reaction reached completion, as indicated by TLC. The reaction was concentrated under vacuum and purified by flash chromatography (SiO_2, Hexane/EtOAc) to give compound **2** (2.2 g, 80%) as a solid. R_f = 0.4 (Hexane/EtOAc, 70:30, v/v).

^{1}H NMR (400 MHz, $CDCl_3$) δ 7.76 (d, *J* = 7.5 Hz, 2H), 7.59 (dd, *J* = 12.4, 7.5 Hz, 2H), 7.53 – 7.48 (m, 2H), 7.44 – 7.34 (m, 4H), 7.33 – 7.27 (m, 4H), 7.14 (d, *J* = 7.9 Hz, 2H), 5.37 (d, *J* = 3.2 Hz, 1H), 4.89 – 4.81 (m, 2H), 4.71 – 4.60 (m, 2H), 4.56 – 4.44 (m, 1H), 4.33 – 4.21 (m, 2H), 3.82 – 3.70 (m, 2H), 2.85 – 2.61 (m, 4H), 2.35 (s, 3H), 2.16 (s, 3H), 1.27 – 1.22 (m, 4H). **^{13}C NMR** (101 MHz, $CDCl_3$) δ 206.3, 172.6, 154.3, 143.8, 143.2, 141.4, 138.1, 137.9, 132.9, 129.8, 129.6, 128.5, 128.2, 128.0, 128.0, 127.3, 127.3, 125.4, 125.2, 120.2, 87.9, 79.1, 75.7, 75.0, 73.0, 70.8, 70.3, 46.8, 38.1, 30.0, 21.3. **HRMS** QTOF-MS: calcd. $C_{40}H_{40}NaO_8S$ for $[M+Na]^+$ 703.2342, found 703.2343.

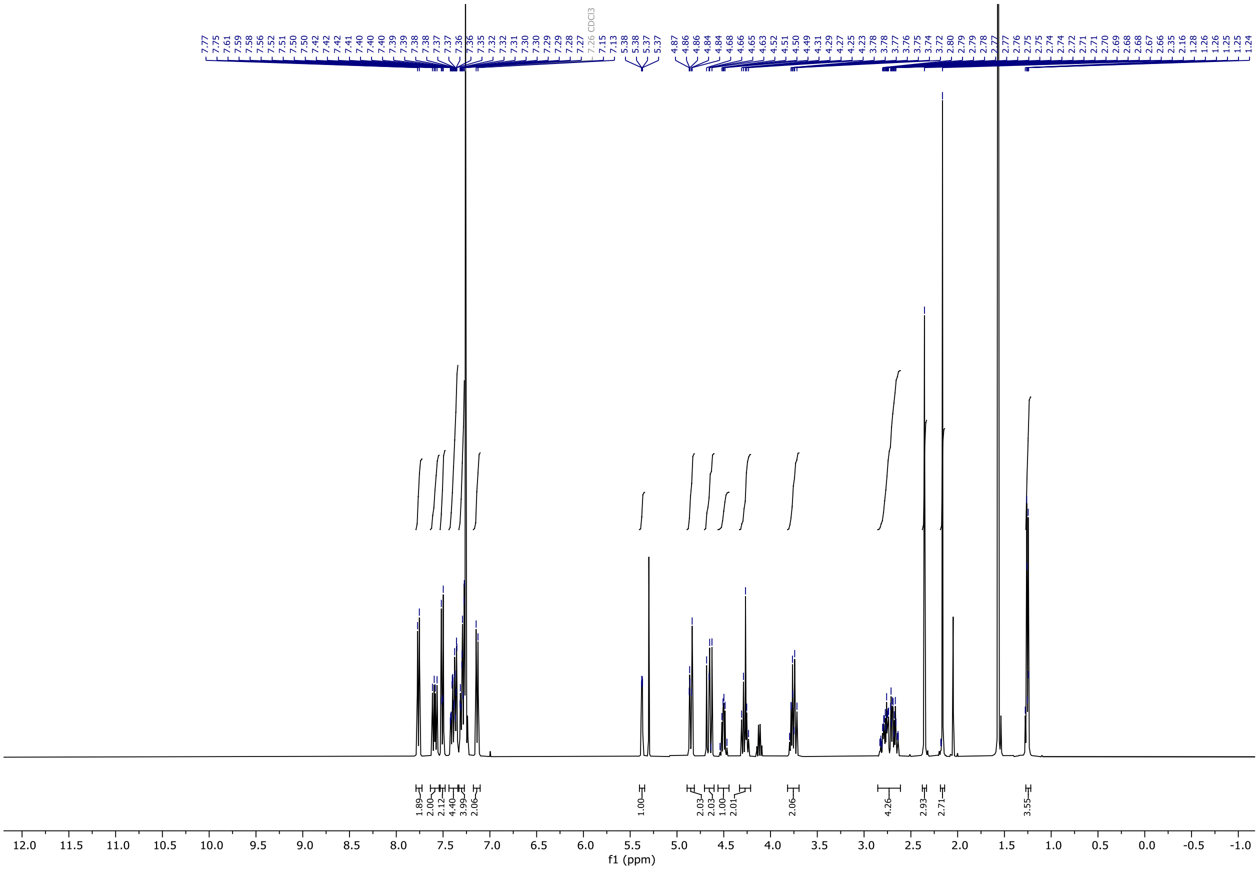

^{1}H NMR

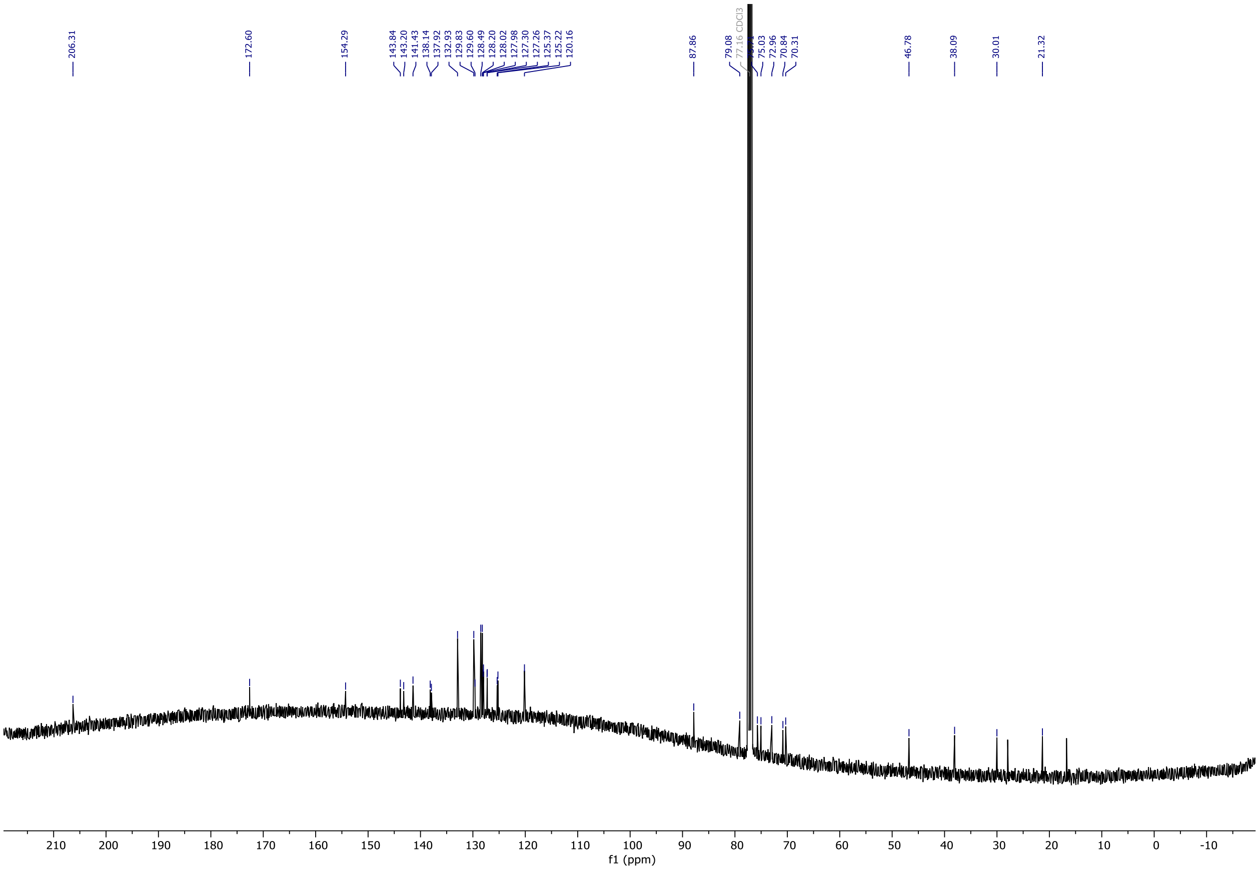

^{13}C NMR

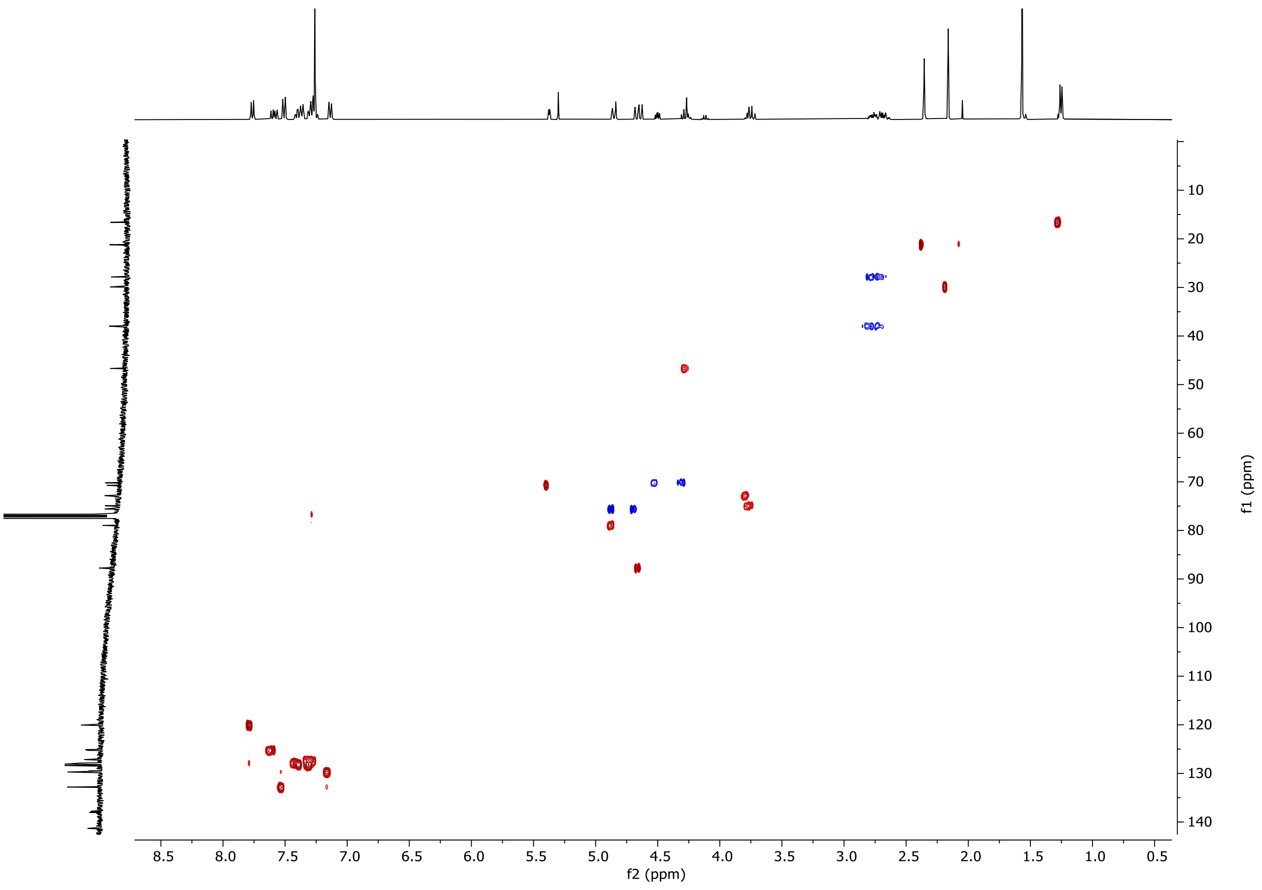

^{1}H-^{13}C HSQC NMR

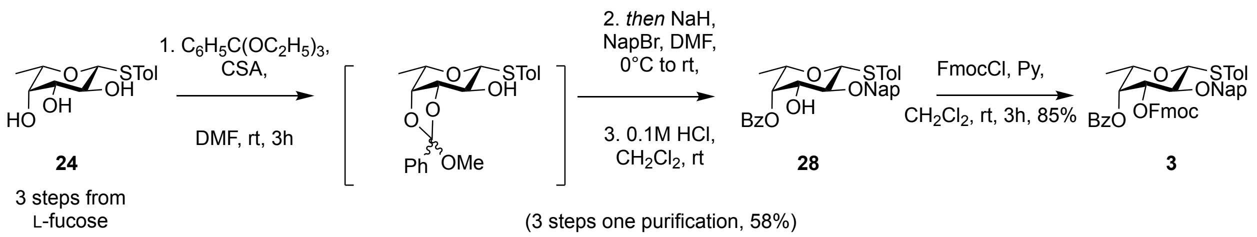

SI Scheme 3. Synthesis of building block **3**.

4-Methylphenyl 4-*O*-benzoyl-2-*O*-(2-naphthalenylmethyl)-1-thio-β-L-fucopyranoside (28)

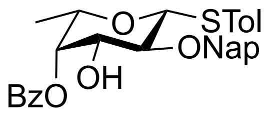

4-Methylphenyl 1-thio-β-L-fucopyranoside **24** (2 g, 7.4mmol) and triethyl ortho-benzoate (2.5 mL, 11.1 mmol, 1.5 eq.) were dissolved in CH_2Cl_2 (0.1M, 75 mL), and camphorsulfonic acid (0.35 g, 1.48 mmol, 0.2 eq.) was added. The mixture was stirred at room temperature under a nitrogen atmosphere until the reaction was complete, as indicated by TLC (≈1 h). Subsequently, the reaction was neutralized with Et_3N (0.4 mL, 2.96 mmol, 0.4 eq.) and concentrated under vacuum. The crude material was then re-dissolved in DMF (0.1M, 75 mL), cooled to 0 °C and sodium hydride (60% dispersion, 0.35 g, 8.8 mmol, 1.2 eq.) was added. After 10 minutes, 2-naphthylmethyl bromide (3.2 mL, 14.8 mmol, 2 eq.) was added. Once complete, as indicated by TLC analysis (≈3 h), the reaction was quenched by the addition of methanol, and stirred for 20 minutes. The reaction mixture was then concentrated under vacuum. The residue was then re-dissolved in CH_2Cl_2 and successively washed with saturated aqueous $NaHCO_3$, brine, dried over $MgSO_4$, and concentrated under vacuum. Subsequently, the crude material was dissolved in CH_2Cl_2 and stirred with 0.1M HCl solution. Once the reaction reached completion, it was extracted, and washed with saturated aqueous $NaHCO_3$, brine, dried over $MgSO_4$, and concentrated. The residue was purified by flash column chromatography (SiO_2, Hexane/ Ethyl acetate) to give compound **28** (2.2 g, 58% over three steps) as a solid. R_f = 0.38 (Hexane/Ethyl acetate, 60:40, v/v).

^{1}H NMR (400 MHz, $CDCl_3$) δ 8.04 (d, 2H), 7.67 – 7.55 (m, 3H), 7.51 – 7.42 (m, 2H), 7.41 – 7.27 (m, 5H), 7.22 – 7.11 (m, 2H), 5.43 (s, 1H), 4.95 (d, *J* = 10.7 Hz, 1H), 4.68 – 4.58 (m, 2H), 3.94 (dd, *J* = 9.2, 3.4 Hz, 1H), 3.85 (qd, *J* = 6.4, 1.1 Hz, 1H), 3.64 (t, *J* = 9.4 Hz, 1H), 2.39 (s, 3H), 1.29 (t, *J* = 6.4 Hz, 3H). **^{13}C NMR** (101 MHz, $CDCl_3$) δ 166.8, 138.2, 138.0, 133.5, 133.1, 130.2, 129.9, 128.7, 128.5, 128.4, 128.2, 87.1, 75.4, 74.4, 73.7, 73.5, 21.4, 17.0. **HRMS** QTOF-MS: calcd. $C_{31}H_{30}NaO_5S$ for $[M+Na]^+$ 537.1712, found 537.1716.

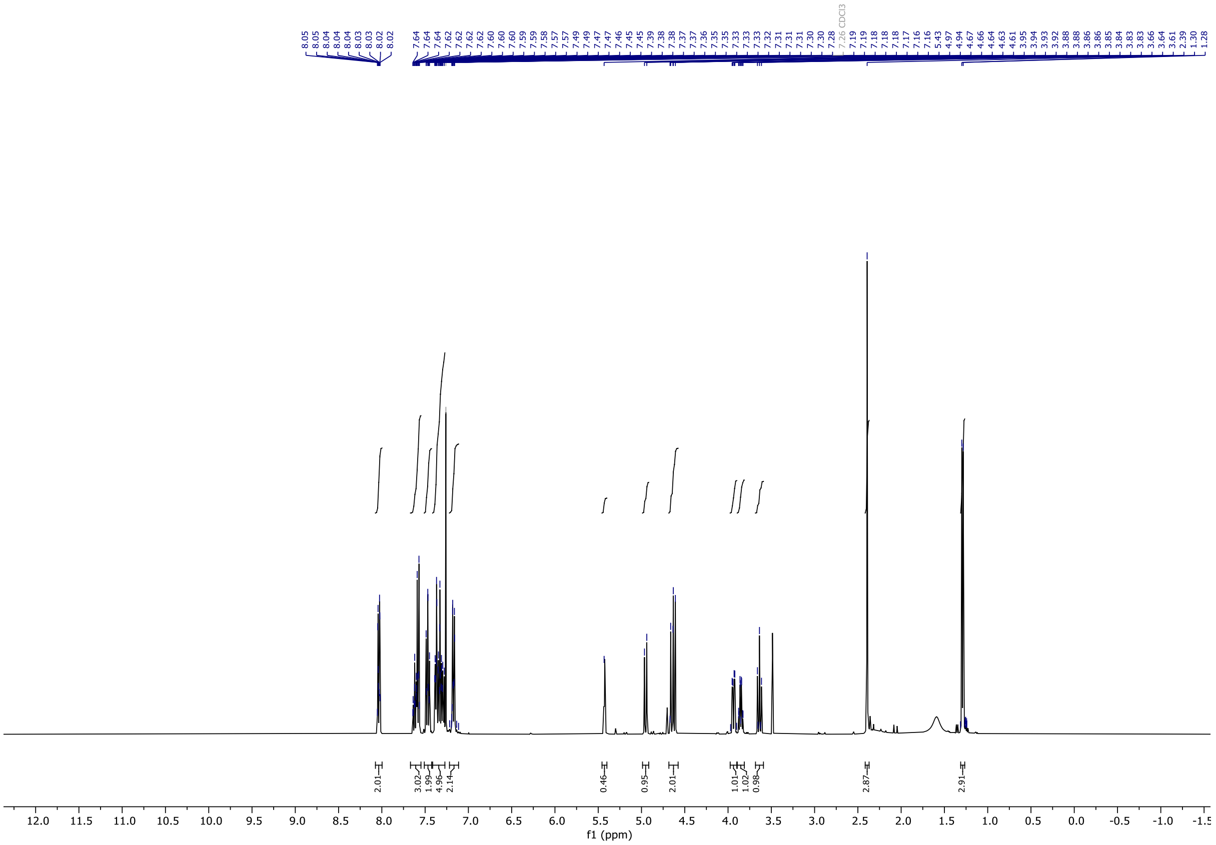

^{1}H NMR

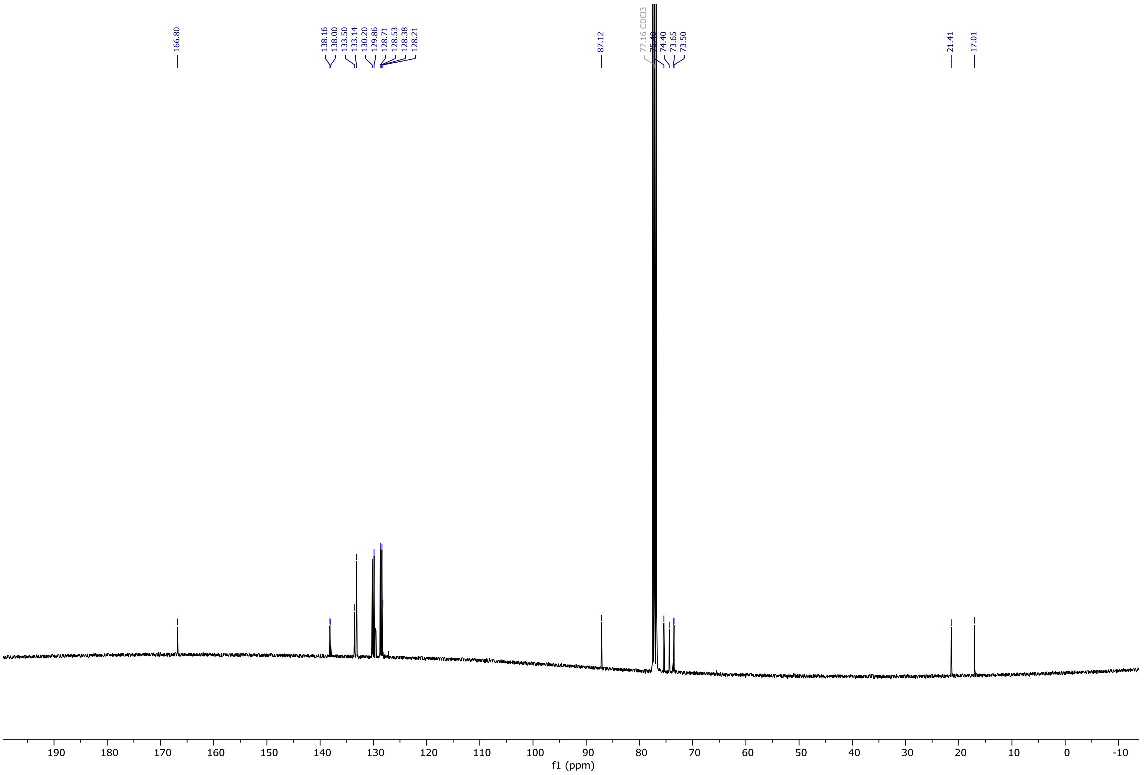

^{13}C NMR

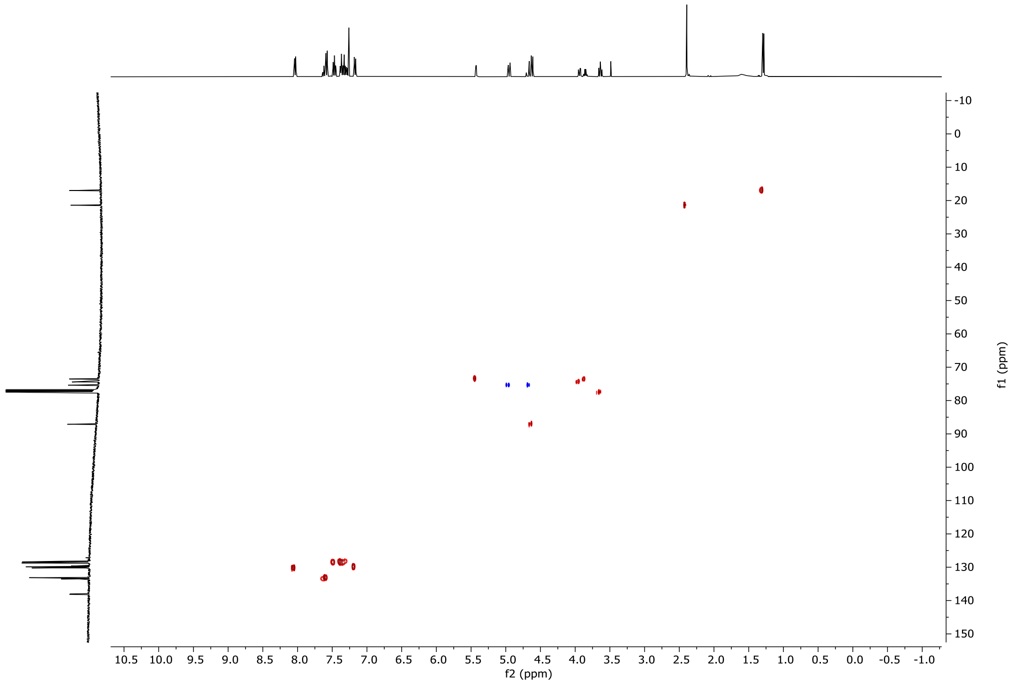

^{1}H-^{13}C HSQC NMR

4-Methylphenyl 4-*O*-benzoyl-3-*O*-(9-fluorenylmethoxycarbonyl)-2-*O*-(2-naphthalenylmethyl)-1-thio-β-L-fucopyranoside (3)

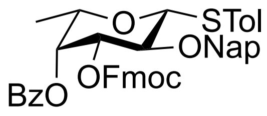

4-Methylphenyl 4-*O*-benzoyl-2-*O*-(2-naphthalenylmethyl)-1-thio-β-L-fucopyranoside **28** (2.0 g, 3.89 mmol) was dissolved in CH_2Cl_2 (0.1M, 40 mL) and pyridine (1.4 mL, 15.6 mmol, 4 eq.). Subsequently, 9-fluorenylmethoxycarbonylchloride (1.2 g, 4.67 mmol, 1.2 eq.) was added to the solution at room temperature under nitrogen. Once the reaction reached completion, as indicated by TLC, it was concentrated under vacuum and purified by flash chromatography (Hexane/EtOAc) to give compound **3** (2.4 g, 85%) as a solid. R_f = 0.35 (Hexane/EtOAc, 90:10, v/v).

^{1}H NMR (400 MHz, $CDCl_3$) δ 7.98 – 7.91 (m, 2H), 7.74 – 7.64 (m, 6H), 7.61 – 7.51 (m, 3H), 7.47 – 7.34 (m, 7H), 7.35 – 7.21 (m, 2H), 7.17 – 7.10 (m, 3H), 7.08 – 7.02 (m, 1H), 5.57 (d, *J* = 1.0 Hz, 1H), 5.00 – 4.90 (m, 2H), 4.75 (d, *J* = 11.1 Hz, 1H), 4.66 (d, *J* = 9.6 Hz, 1H), 4.43 (dd, *J* = 10.2, 6.7 Hz, 1H), 4.13 (t, *J* = 7.4 Hz, 1H), 4.07 – 3.96 (m, 1H), 3.94 – 3.77 (m, 2H), 2.34 (s, 3H), 1.25 (d, *J* = 6.4 Hz, 3H). **^{13}C NMR** (101 MHz, $CDCl_3$) δ 166.1, 154.4, 143.8, 143.2, 141.4, 141.3, 138.3, 135.5, 133.7, 133.6, 133.3, 133.1, 130.3, 129.9, 129.5, 128.8, 128.6, 128.2, 128.1, 128.0, 127.9, 127.8, 127.3, 127.2, 126.7, 126.1, 126.1, 126.0, 125.5, 125.3, 120.1, 120.0, 87.1, 79.4, 75.7, 74.8, 73.3, 71.2, 70.4, 46.7, 21.5, 16.9. **HRMS** QTOF-MS: calcd. $C_{46}H_{40}NaO_7S$ for $[M+Na]^+$ 759.2392, found 759.2398.

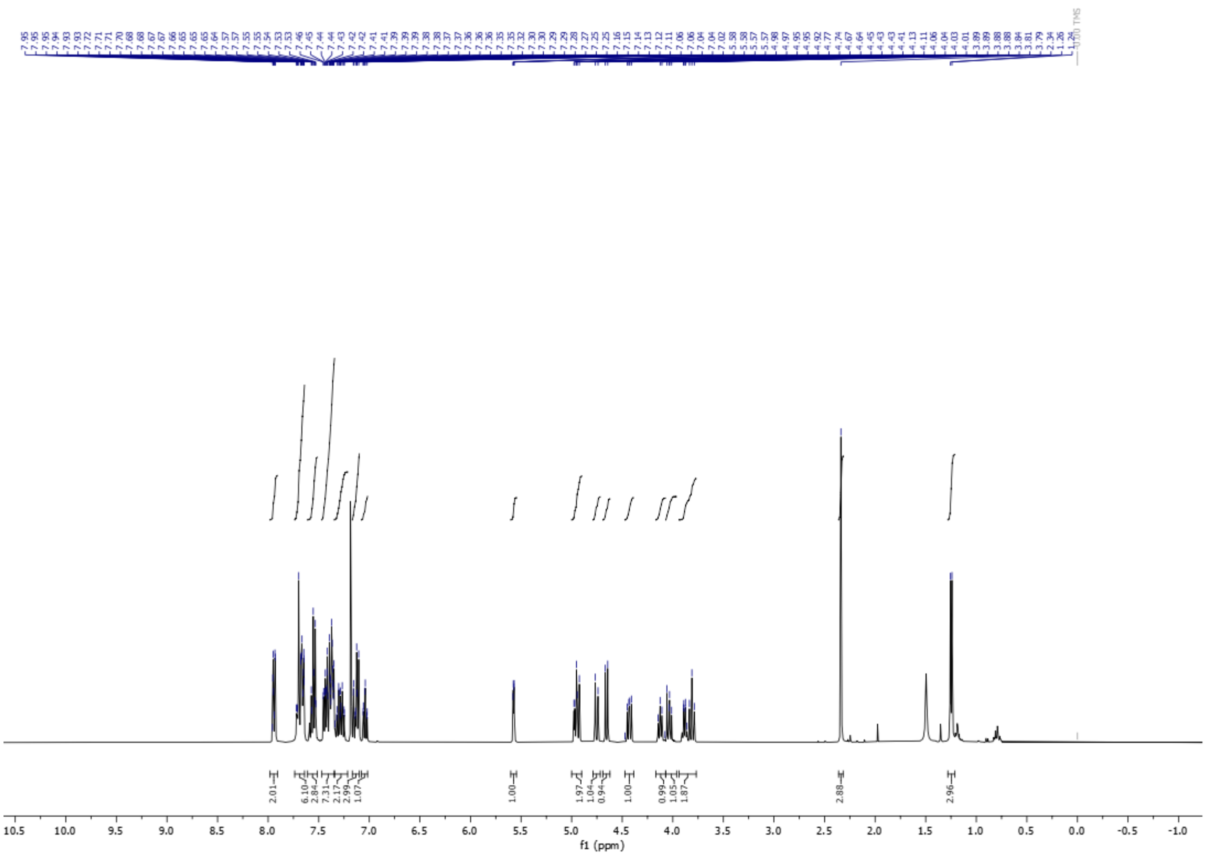

^{1}H NMR

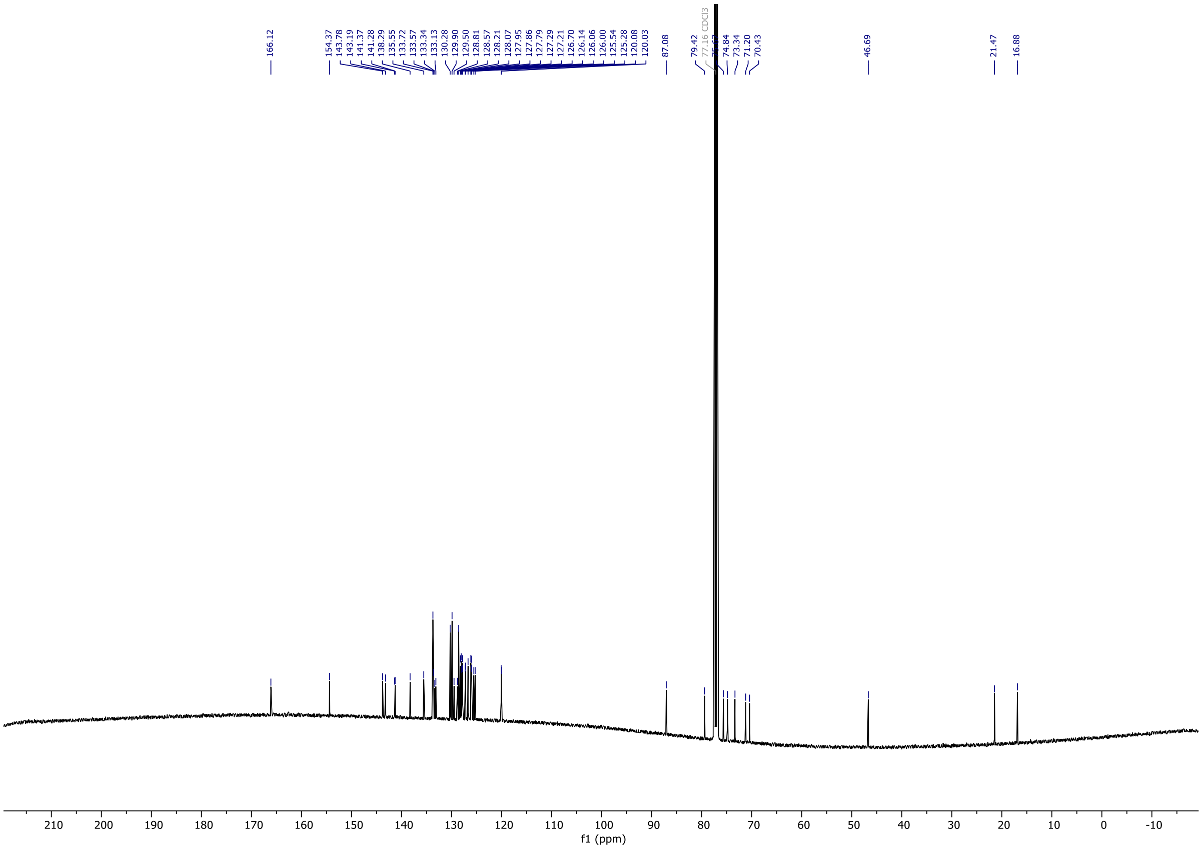

^{13}C NMR

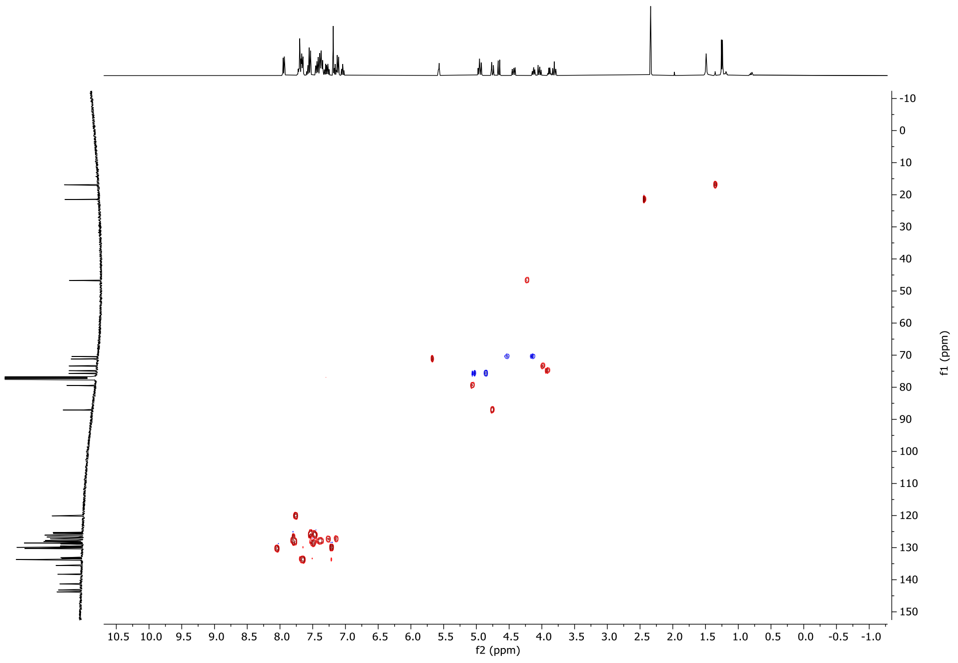

^{1}H-^{13}C HSQC NMR

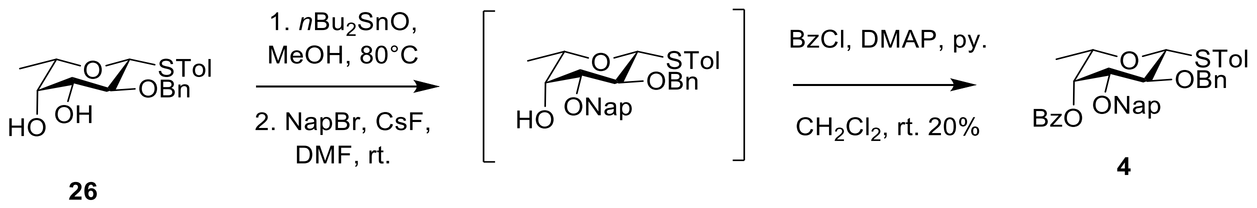

SI Scheme 4. Synthesis of building block **4**.

4-Methylphenyl 4-*O*-benzoyl-2-*O*-benzyl-3-*O*-(2-napthalenylmethyl)-1-thio-β-L-fucopyranoside (4)

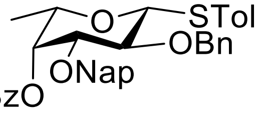

4-Methylphenyl 2-O-benzyl-1-thio-β-L-fucopyranoside **26** (0.6 g, 1.67 mmol) and dibutyltin (IV) oxide (0.5 g, 2.0 mmol, 1.2 eq.) were dissolved in methanol (0.1 M, 20 mL). The mixture was heated to 80 °C in an oil bath and refluxed under an argon atmosphere for 3 hours. Subsequently, the reaction was concentrated under vacuum. The crude material was then re-dissolved in DMF (0.1 M, 20 mL), cooled to °C, and 2-(bromomethyl)naphthalene (0.52 g, 2.5 mmol, 1.5 eq.) and cesium fluoride (0.75 g, 5.0 mmol, 3 eq.) were added. The mixture was stirred at room temperature under an argon atmosphere overnight. Once complete, as indicated by TLC analysis (≈16 h), the reaction was concentrated under vacuum. The residue was then re-dissolved in ethyl acetate and successively washed with H_2O, dried over Na_2SO_4, and concentrated. The residue was purified by flash chromatography (SiO_2, Hexane/Ethyl acetate). The 4-methylphenyl 3-*O*-(2-naphthalenylmethyl)-2-*O*-benzyl-1-thio-β-L-fucopyranoside intermediate was dissolved in CH_2Cl_2 (0.1 M, 20 mL) at 0 °C. Subsequently, benzoyl chloride (0.13 mL, 1.0 mmol, 2 eq.), pyridine (0.2 mL, 2.5 mmol, 5 eq.) and 4-dimethylaminopyridine (12 mg, 0.1 mmol, 0.2 eq.) were added. The mixture was stirred at room temperature under argon atmosphere. Once complete, as indicated by TLC analysis (≈16 h), the reaction was quenched with citric acid (10% w/v) and extracted. The organic layer was washed with saturated aqueous $NaHCO_3$, brine, dried over Na_2SO_4, and concentrated. The residue was purified by flash chromatography (SiO_2, Hexane/Ethyl acetate) to give compound **4**

(0.2 g, 0.33 mmol, 20% over 2 steps) as white solid. R_f = 0.91 (Hexane/Ethyl acetate, 50:50, v/v).

^{1}H NMR (400 MHz, $CDCl_3$) δ 8.09 (m, *J* = 17.1, 7.0, 1.4 Hz, 3H), 7.81 – 7.73 (m, 1H), 7.66 – 7.54 (m, 4H), 7.53 – 7.26 (m, 13H), 7.15 (d, *J* = 8.0 Hz, 2H), 5.66 (m, *J* = 3.3, 1.0 Hz, 1H), 4.94 (d, *J* = 11.5 Hz, 1H), 4.80 – 4.68 (m, 3H), 4.61 (d, *J* = 9.5 Hz, 1H), 3.86 – 3.77 (m, 2H), 3.71 (t, *J* = 9.3 Hz, 1H), 2.38 (s, 3H), 1.31 (d, *J* = 6.4 Hz, 3H). **^{13}C NMR** (101 MHz, $CDCl_3$) δ 166.4, 138.6, 138.0, 135.2, 133.9, 133.7, 133.4, 133.3, 133.1, 130.3, 130.2, 129.9, 129.8, 129.2, 128.6, 128.5, 128.3, 128.3, 128.0, 127.9, 127.7, 127.1, 126.2, 126.1, 126.0, 87.2, 81.4, 76.3, 75.7, 73.5, 71.9, 70.5, 21.4, 17.1. **HRMS** QTOF-MS: calcd. $C_{38}H_{36}NaO_5S$ for $[M+Na]^+$ 627.2181, found 627.2203.

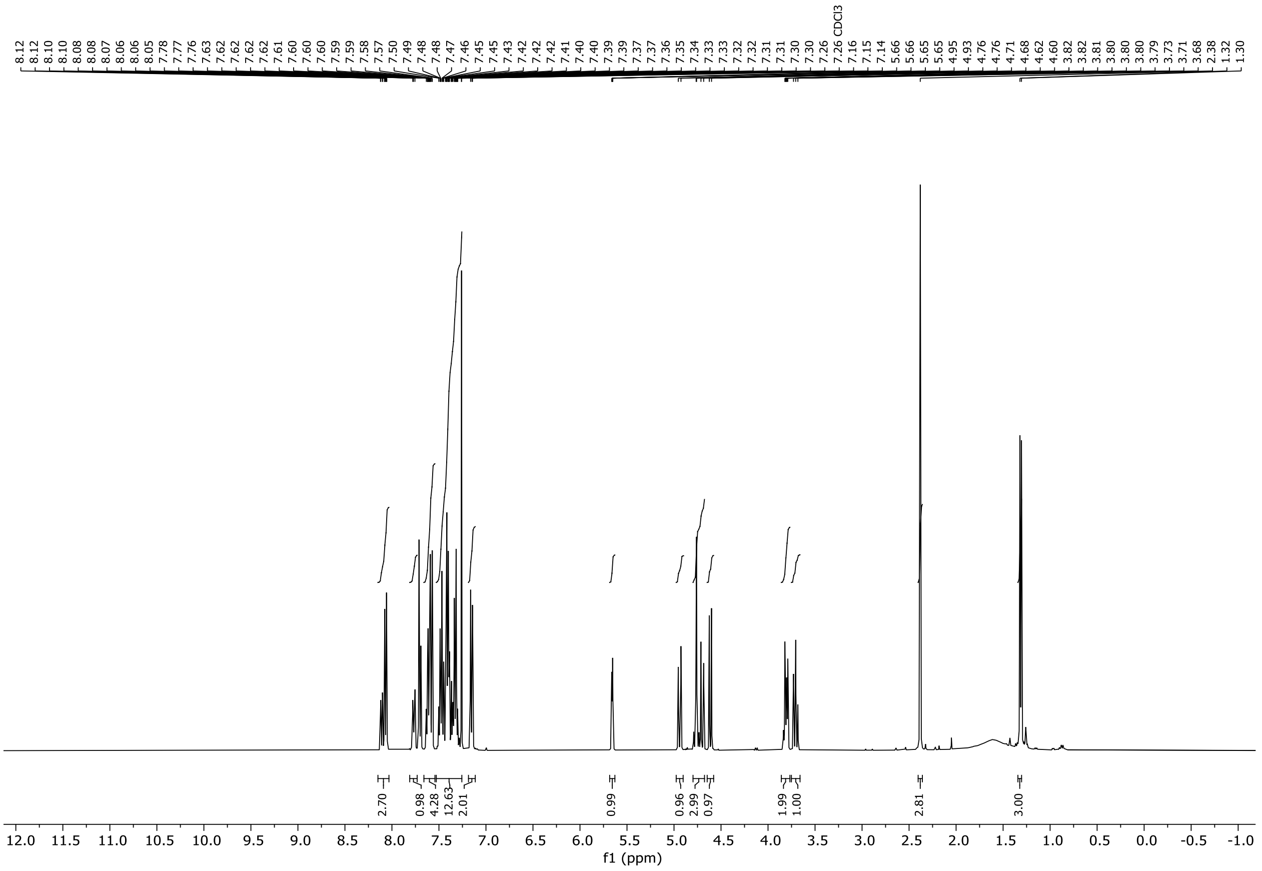

^{1}H NMR

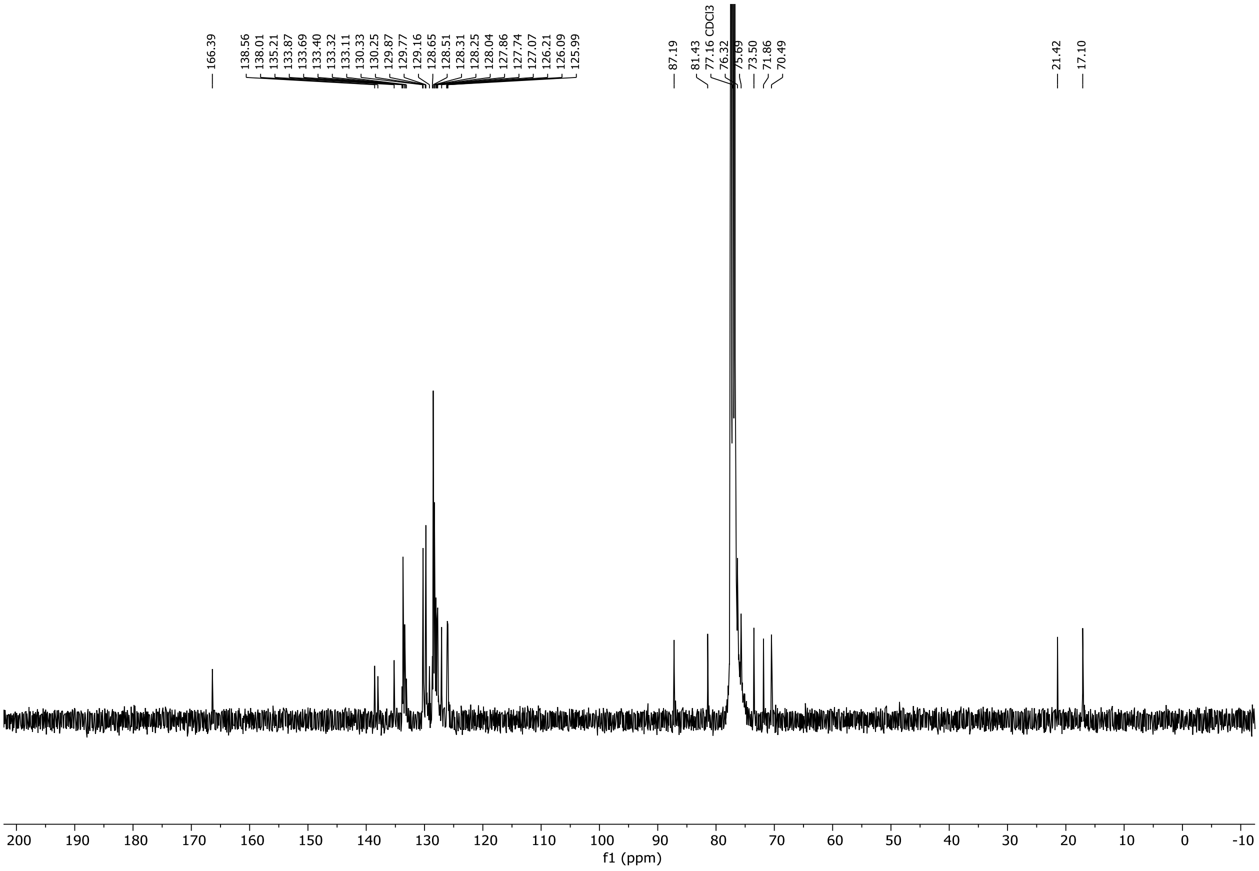
166.39
138.56
138.01
135.21
133.87
133.69
133.40
133.32
133.11
130.33
130.25
129.87
129.77
129.16
128.65
128.51
128.31
128.25
128.04
127.86
127.74
127.07
126.21
126.09
125.99
87.19
81.43
77.16 CDCl3
76.32
75.69
73.50
71.86
70.49
21.42
17.10
f1 (ppm)

^{13}C NMR

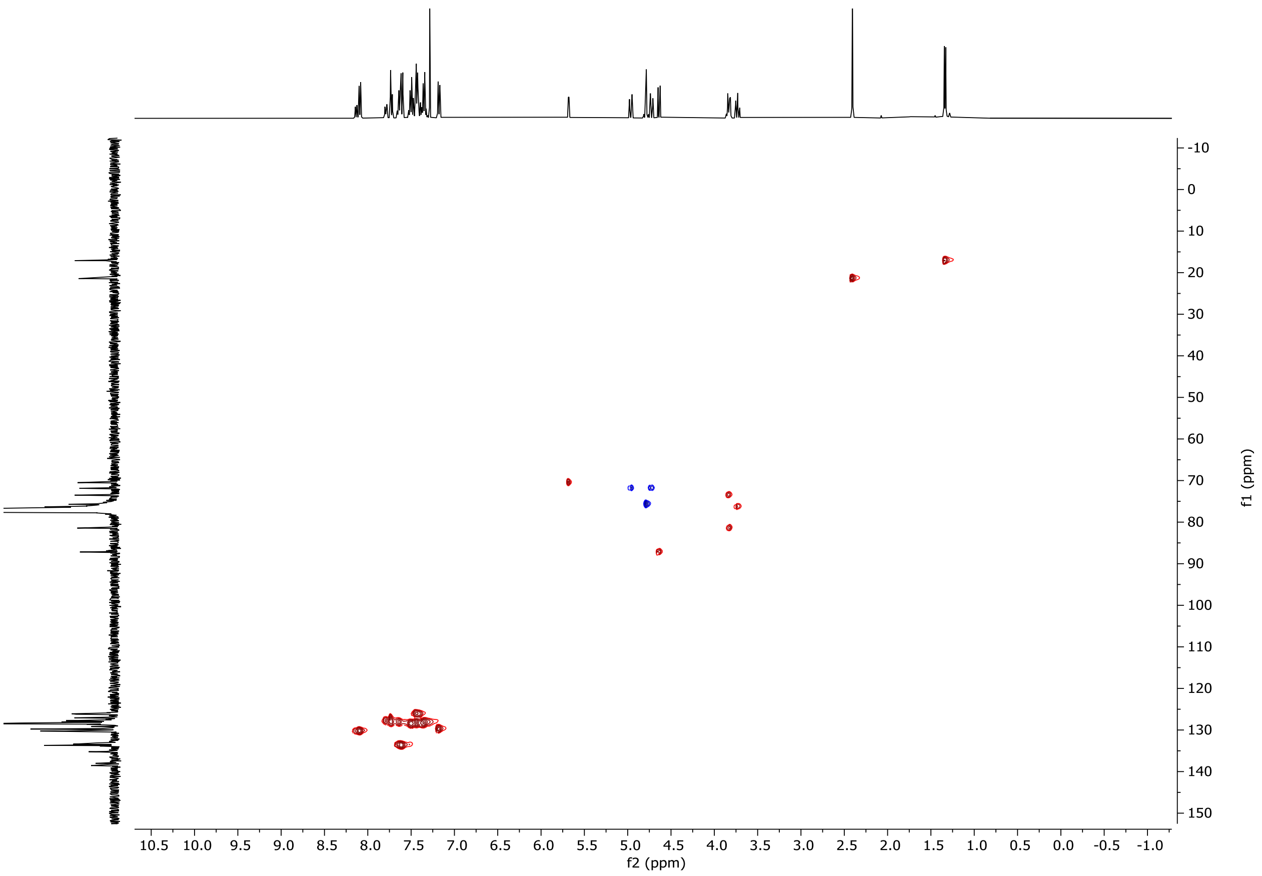

^{1}H-^{13}C HSQC NMR

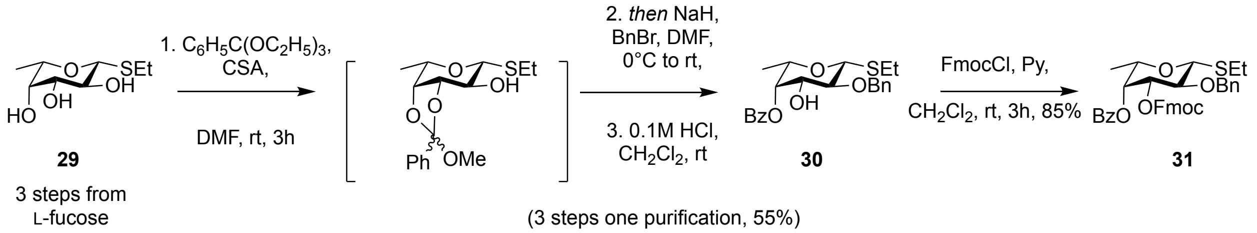

SI Scheme 5. Synthesis of building block **31**.

Ethyl 1-thio-β-L-fucopyranoside (29)

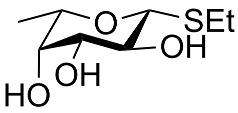

L-Fucose (3 g, 18.3 mmol) was suspended in CH_2Cl_2 (0.1M, 180 mL), pyridine (30 ml, 366 mmol, 20eq.), and acetic acid (10 mL, 110 mmol, 6 eq.). Subsequently, a catalytic quantity of 4-(dimethylamino)pyridine (200 mg, 1.8 mmol, 0.1 eq.) was added. The mixture left to stir at room temperature (≈16h) and then concentrated under vacuum to afford a thick yellow gel. To a solution of this crude product in CH_2Cl_2 (0.05M, 360 mL), ethanethiol (2.9 mL, 38.4 mmol, 2.1 eq.) was added. The reaction was cooled to at 0°C and $BF_3{\cdot}Et_2O$ (7 mL, 55 mmol, 3 eq.) was added slowly. The temperature was allowed to rise to room temperature. Once complete (≈16 h), the reaction was quenched by the addition of saturated aqueous $NaHCO_3$, extracted with CH_2Cl_2, washed with brine, dried over $MgSO_4$ and concentrated under vacuum. Purification by flash chromatography (SiO_2, hexane/ethyl acetate) afforded the ethyl 2,3,4-*O*-acetyl-tri-1-thio-β-L-fucopyranoside intermediate, which was dissolved in CH_2Cl_2 (0.1M, 180 mL) and the pH was raised to pH 8 with a sodium methoxide (approx. 1 mL, 0.5M solution). Once complete, the reaction was quenched by the addition of Amberlite™ (Hydrogen foam), filtered and concentrated under vacuum. Purification by flash chromatography (SiO_2, CH_2Cl_2/methanol) afforded **29** (2 g, 52%) as a white amorphous solid. R_f = 0.3 (CH_2Cl_2/methanol, 95:5, v/v).

^{1}H NMR (400 MHz, MeOD) δ 4.26 (d, *J* = 8.9 Hz, 1H), 3.63 – 3.58 (m, 2H), 3.50 – 3.37 (m, 2H), 2.77 – 2.57 (m, 3H), 1.27 – 1.20 (m, 5H). **^{13}C NMR** (101 MHz, MeOD) δ 87.2, 76.4, 76.0, 73.2, 71.1, 25.0, 17.0, 15.5. **HRMS** QTOF-MS: calcd. $C_8H_{16}NaO_4S$ for $[M+Na]^+$ 231.0667, found 231.0670.

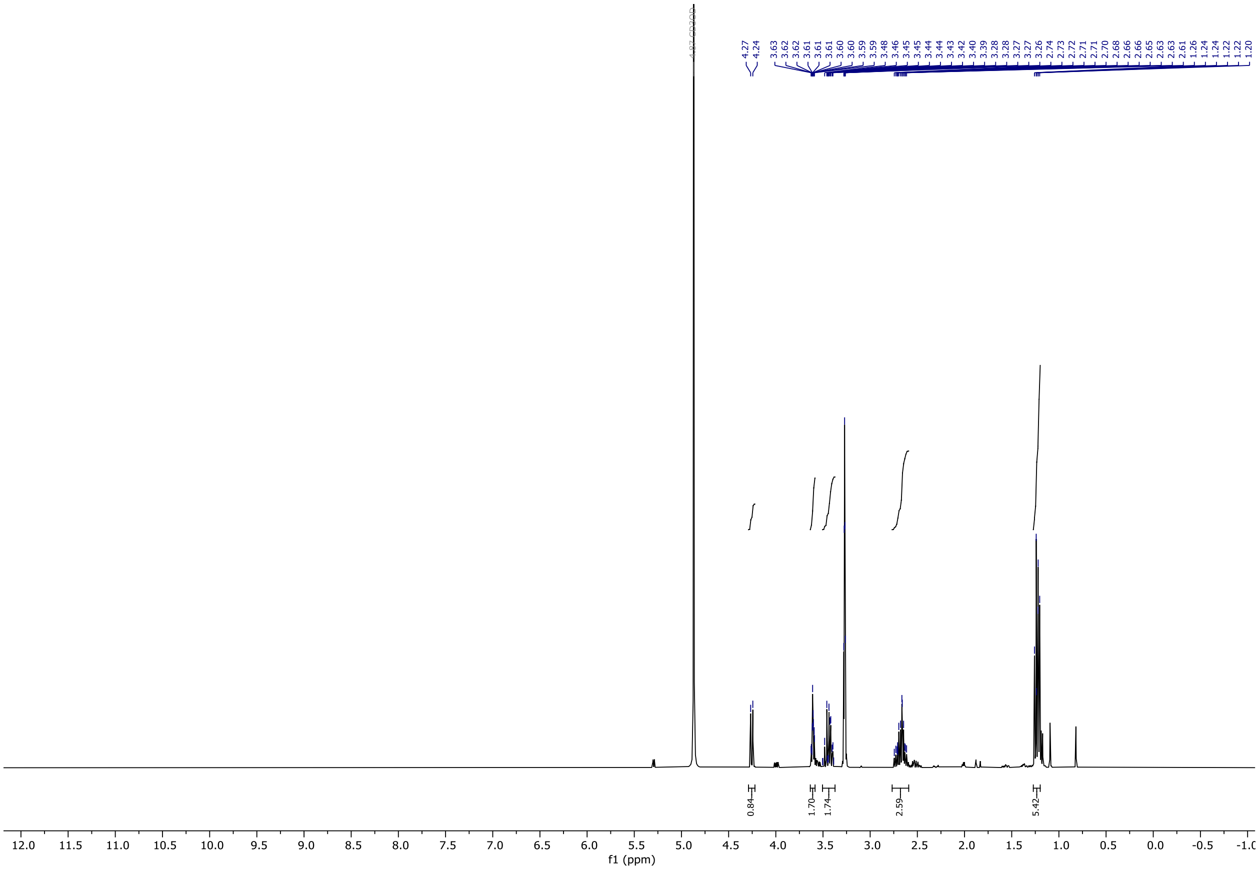

^{1}H NMR

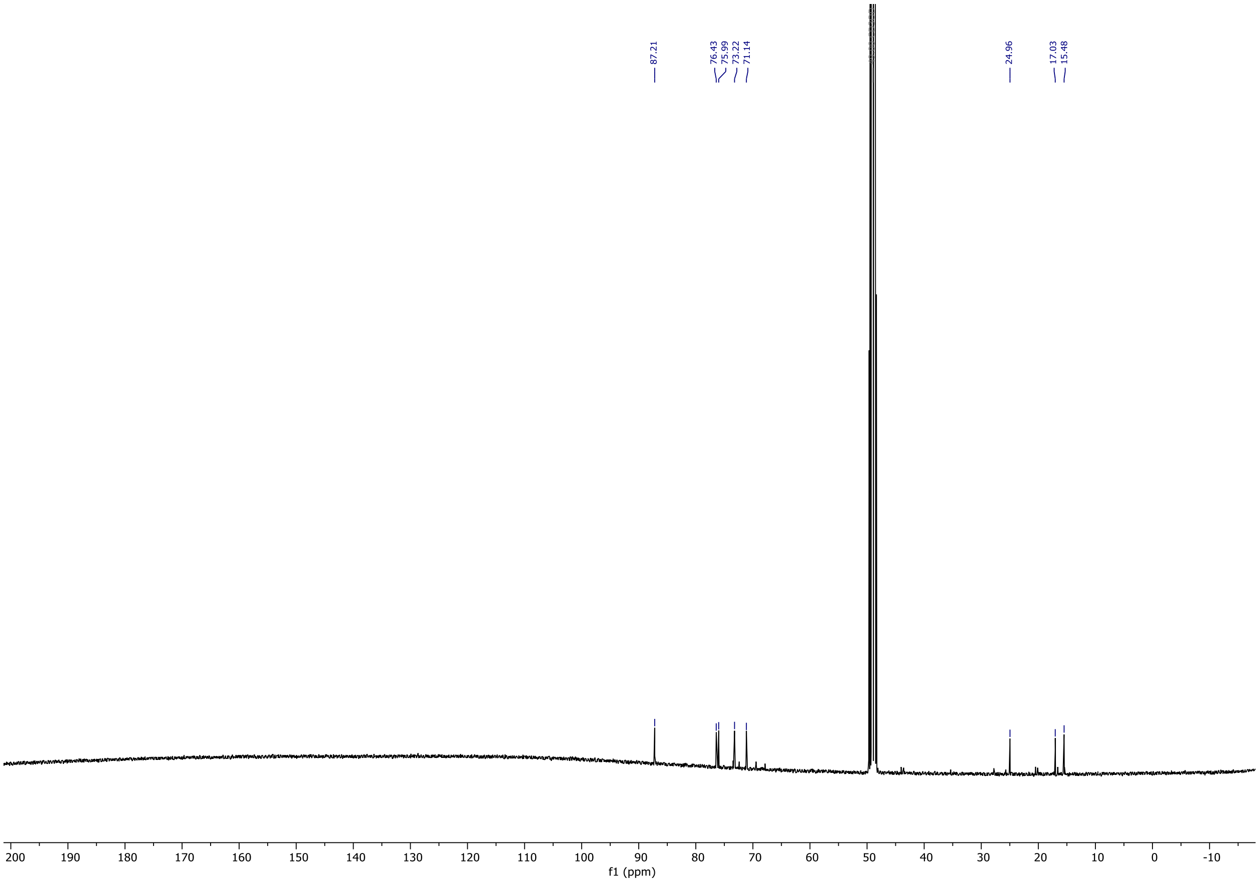

^{13}C NMR

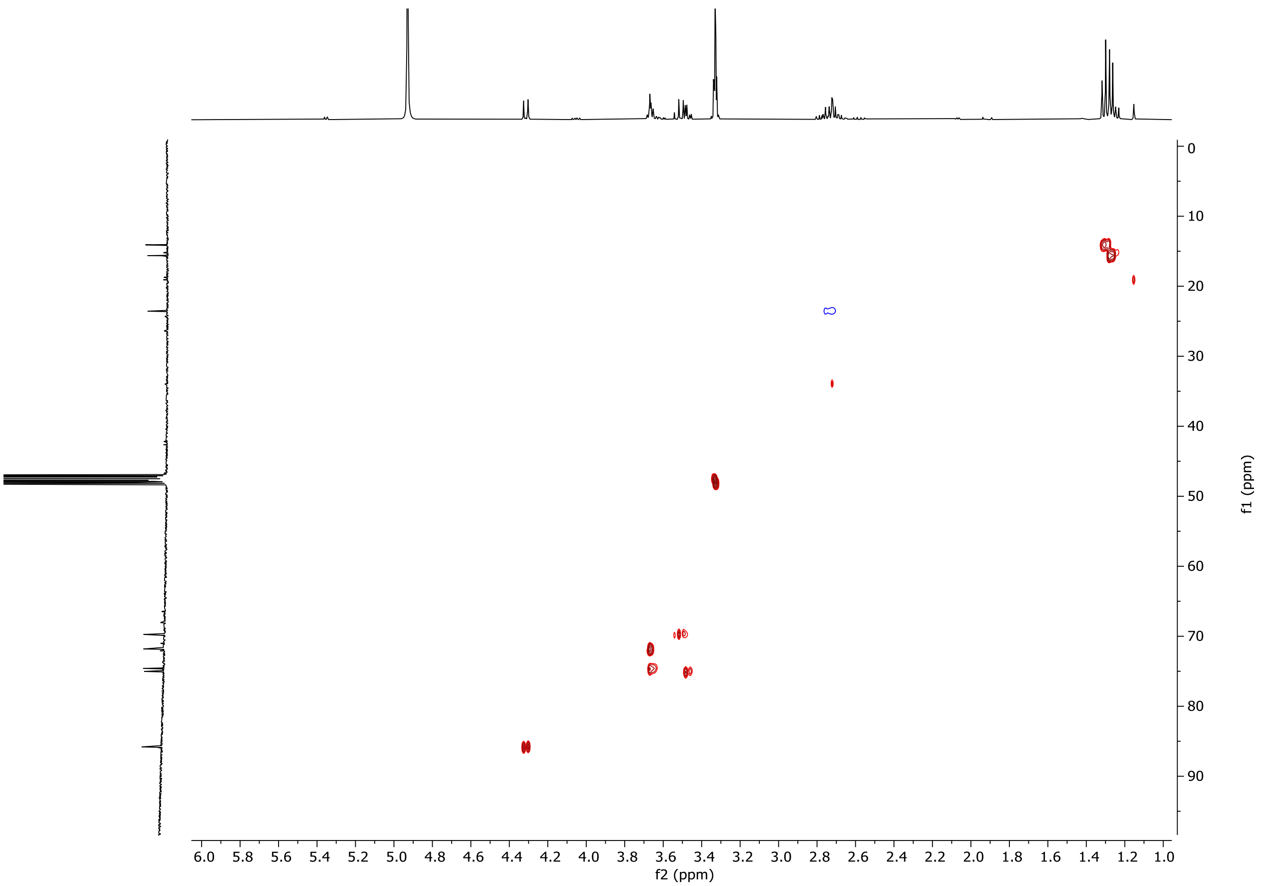

^{1}H-^{13}C HSQC NMR

Ethyl 4-*O*-benzoyl-2-*O*-benzyl-1-thio-β-L-fucopyranoside (30)

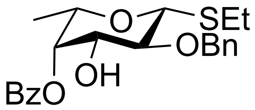

Ethyl 1-thio-β-L-fucopyranoside **29** (3 g, 14.4 mmol) and triethyl ortho-benzoate (5 mL, 21.6 mmol, 1.5 eq.) were dissolved in CH_2Cl_2 (0.1M, 140 mL), and camphorsulfonic acid (0.3 g, 1.4 mmol, 0.1 eq.) was added. The mixture was stirred at room temperature under a nitrogen atmosphere until the reaction was complete. Then the reaction was neutralized with Et_3N (1 mL, 7.2 mmol, 0.5 eq.) and concentrated under vacuum. The crude material was then re-dissolved in DMF (0.1M, 140 mL), cooled to 0 °C, and sodium hydride (60% dispersion, 0.7 g, 17.3 mmol, 1.2 eq.) was added. After 10 minutes, benzyl bromide (2.6 mL, 21.6 mmol, 1.5 eq.) was added and the reaction was allowed to raise to room temperature. The reaction was quenched by the addition of methanol, stirred for 20 minutes and concentrated under vacuum. The crude material was re-dissolved in CH_2Cl_2, and washed with saturated aqueous $NaHCO_3$, brine, dried over $MgSO_4$, and concentrated under vacuum. The crude material was then dissolved in CH_2Cl_2 and stirred with 0.1M HCl solution. Once the reaction reached completion, it was extracted, and washed with saturated aqueous $NaHCO_3$, brine, dried over $MgSO_4$, and concentrated. The residue was purified by flash column chromatography (SiO_2, Hexane/ Ethyl acetate) to give compound **30** (3.1 g, 55% over three steps) as a solid. R_f = 0.45 (Hexane/Ethyl acetate, 60:40, v/v).

^{1}H NMR (400 MHz, $CDCl_3$) δ 8.10 – 8.04 (m, 2H), 7.88 – 7.73 (m, 6H), 7.62 – 7.53 (m, 2H), 7.51 – 7.40 (m, 5H), 5.40 (d, *J* = 3.6 Hz, 1H), 5.15 (d, *J* = 10.9 Hz, 1H), 4.93 – 4.83 (m, 2H), 4.52 (d, *J* = 9.6 Hz, 1H), 4.12 (q, *J* = 7.2 Hz, 1H), 3.92 (dd, *J* = 9.2, 3.5 Hz, 1H), 3.81 – 3.71 (m, 1H), 3.71 – 3.63 (m, 1H), 2.91 – 2.76 (m, 2H), 1.38 (t, *J* = 7.5 Hz, 3H), 1.23 (d, *J* = 6.4 Hz, 3H). **^{13}C NMR**‡ (101 MHz, $CDCl_3$) δ 133.4, 130.1, 128.5, 128.2, 127.4, 126.3, 126.1, 85.0, 78.7, 75.6, 74.2, 73.5, 73.5, 73.4, 25.0, 16.6, 15.0. **HRMS** QTOF-MS: calcd. $C_{22}H_{26}NaO_5S$ for $[M+Na]^+$ 425.1399, found 425.1401.

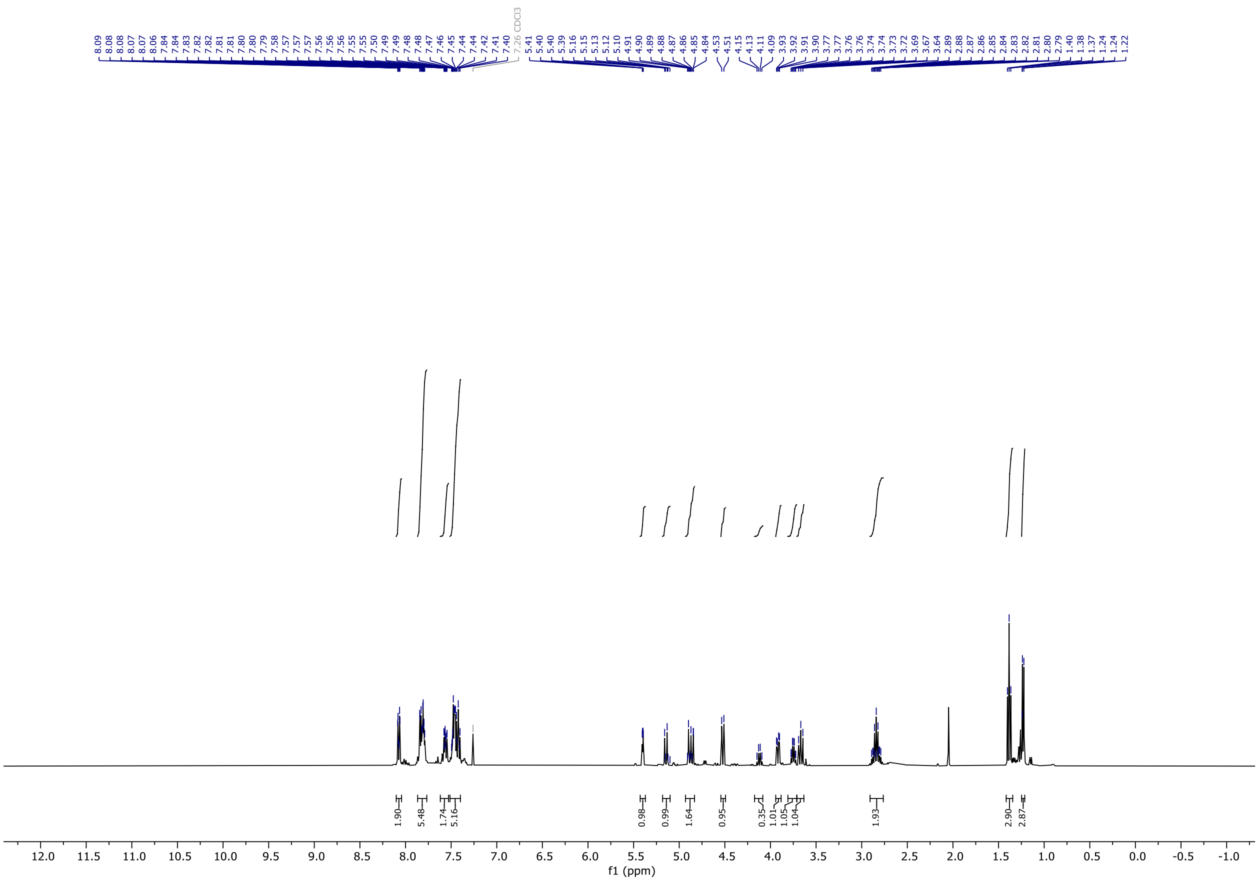

^{1}H NMR

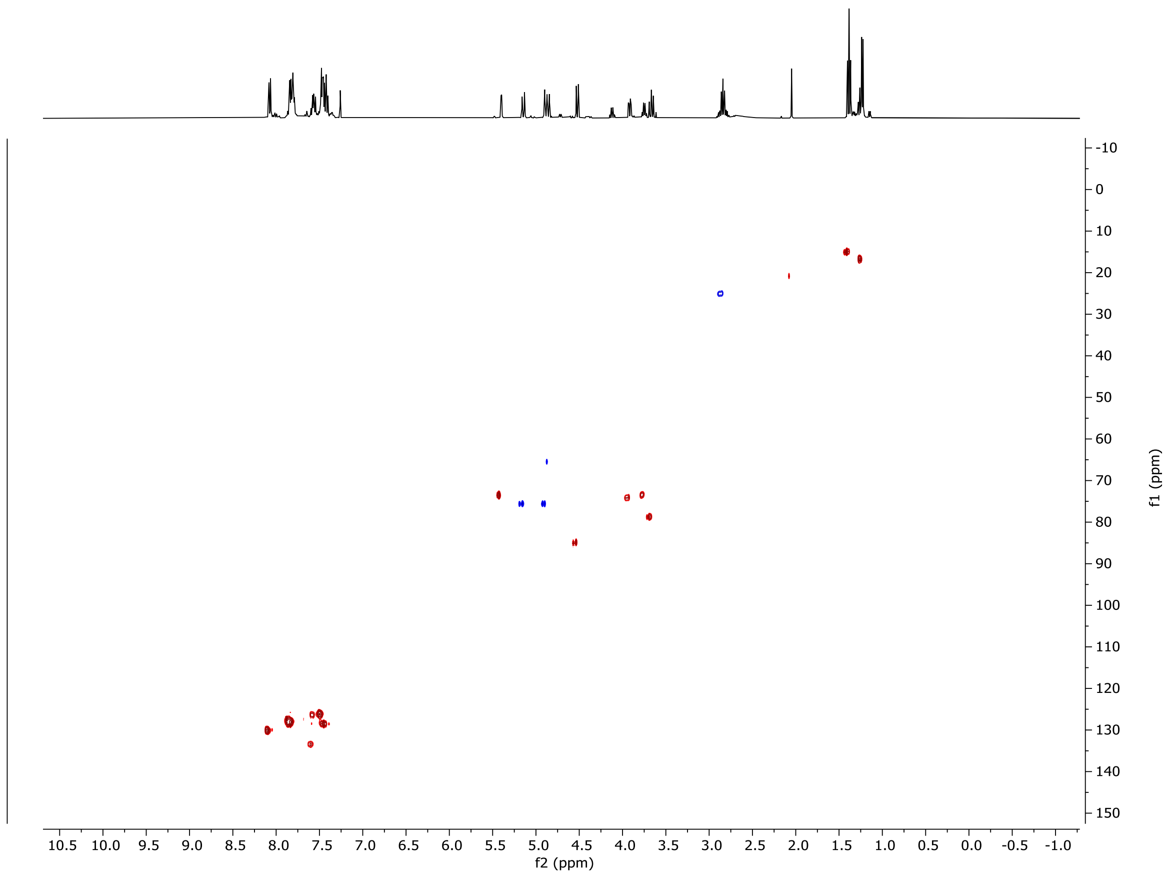

^{1}H-^{13}C HSQC NMR

Ethyl 4-*O*-benzoyl-2-*O*-benzyl-3-*O*-(9-fluorenylmethoxycarbonyl)-1-thio-β-L-fucopyranoside (31)

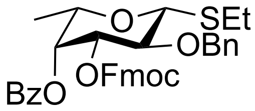

Ethyl 4-*O*-benzoyl-2-*O*-benzyl-1-thio-β-L-fucopyranoside **30** (2.5 g, 6.21 mmol) was dissolved in CH_2Cl_2 (0.1M, 60 mL) and pyridine (2 mL, 24.8 mmol, 4 eq.). Subsequently, 9-fluorenylmethoxycarbonylchloride (1.9 g, 7.45 mmol, 1.2 eq.) was added to the solution at room temperature under nitrogen. Once complete, the reaction was concentrated under vacuum and purified by flash chromatography (Hexane/EtOAc) to give compound **31** (2.4 g, 85%) as a solid. R_f = 0.35 (Hexane/EtOAc, 90:10, v/v).

^{1}H NMR (400 MHz, $CDCl_3$) δ 8.17 – 8.07 (m, 2H), 7.79 – 7.72 (m, 2H), 7.70 – 7.59 (m, 1H), 7.59 – 7.46 (m, 5H), 7.45 – 7.33 (m, 5H), 7.29 (d, *J* = 6.2 Hz, 2H), 7.16 (td, *J* = 7.5, 1.1 Hz, 1H), 5.66 (dd, *J* = 3.4, 1.0 Hz, 1H), 4.99 (dd, *J* = 9.6, 3.4 Hz, 1H), 4.93 – 4.88 (m, 1H), 4.75 – 4.69 (m, 1H), 4.63 – 4.52 (m, 2H), 4.28 (t, *J* = 7.4 Hz, 1H), 4.19 (dd, *J* = 10.1, 8.2 Hz, 1H), 3.91 (dd, *J* = 6.4, 1.1 Hz, 1H), 3.82 (t, *J* = 9.7 Hz, 1H), 2.93 – 2.77 (m, 2H), 1.38 (t, *J* = 7.4 Hz, 3H), 1.28 (d, *J* = 6.4 Hz, 3H). **^{13}C NMR** (101 MHz, $CDCl_3$) δ 166.2, 154.5, 143.9, 143.2, 141.4, 141.3, 137.8, 133.6, 130.3, 129.6, 128.7, 128.5, 128.3, 128.0, 127.9, 127.3, 127.2, 125.6, 125.3, 120.1, 120.1, 85.2, 79.1, 76.8, 76.0, 75.8, 73.2, 71.2, 70.5, 46.8, 25.2, 16.8, 15.1. **HRMS** QTOF-MS: calcd. $C_{37}H_{36}NaO_7S$ for $[M+Na]^+$ 647.2079, found 647.2081.

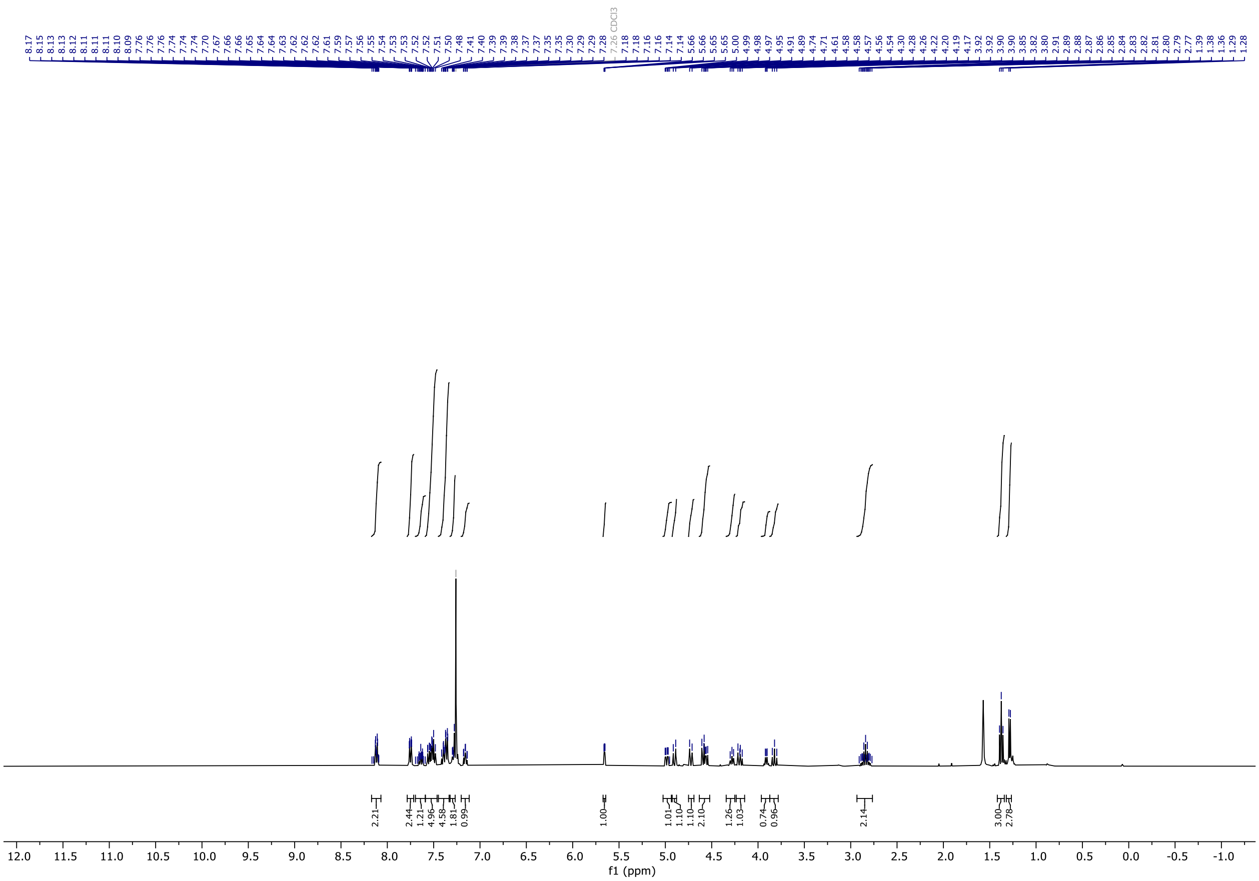

^{1}H NMR

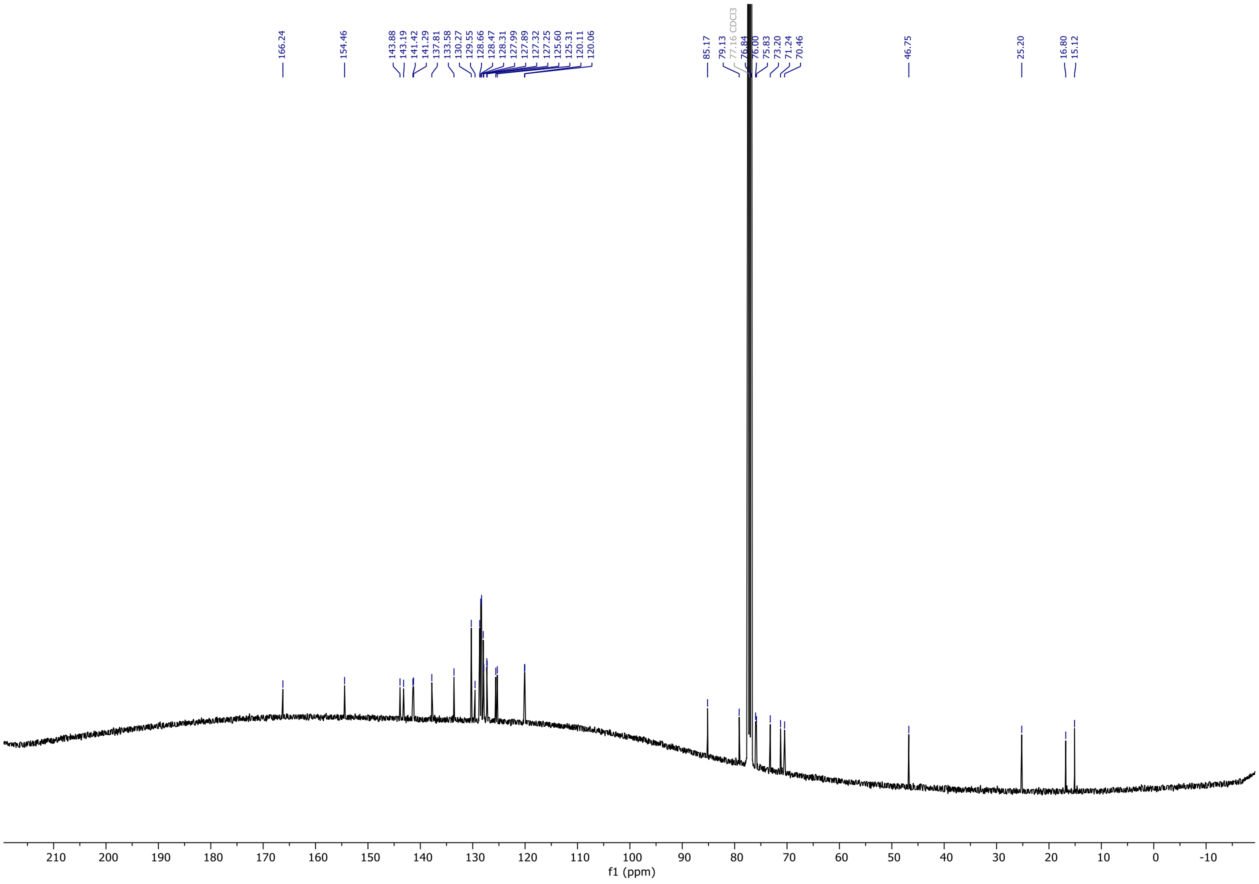

^{13}C NMR

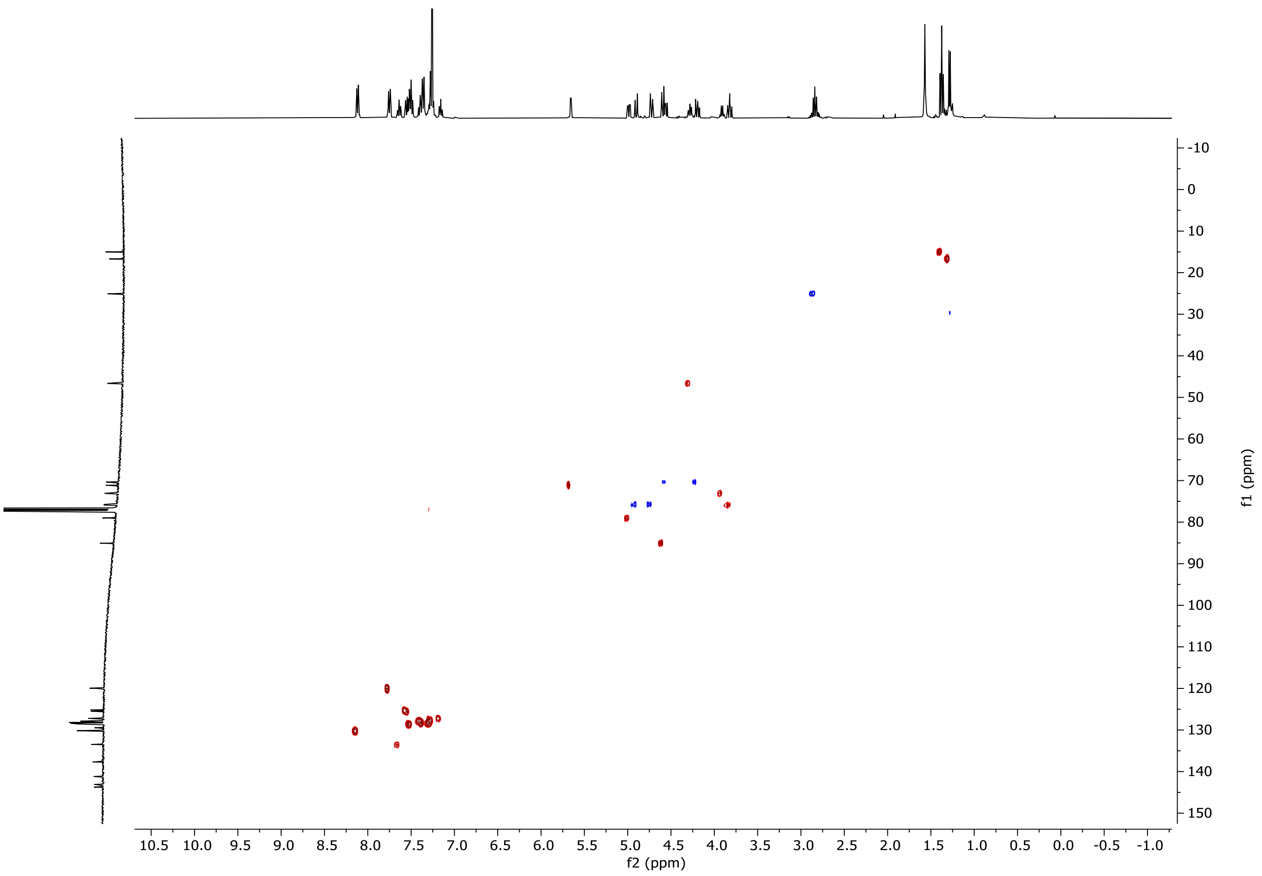

^{1}H-^{13}C HSQC NMR

Dibutyl 4-*O*-benzoyl-2-*O*-benzyl-3-*O*-(9-fluorenylmethoxycarbonyl)-1-phosphate-L-β-fucopyranoside (32)

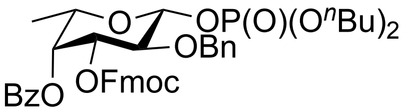

To a solution of thioglycoside **31** (0.8 g, 1.28 mmol) and dibutyl hydrogen phosphate (0.5 mL, 2.56 mmol, 2.0 equiv.) in anhydrous CH_2Cl_2 (0.1 M 15 mL), NIS (0.5 g, 2.3 mmol, 1.8 equiv.) and TfOH (25 µL, 0.256 mmol, 0.3 equiv.) were added at 0 °C. Once the reaction was complete, as indicated by TLC, it was diluted with CH_2Cl_2 and 10% sodium thiosulfate solution was added. The mixture was extracted with CH_2Cl_2, dried over $MgSO_4$, filtered and concentrated. Compound **32** (0.6 g, 60%) was obtained as a white solid after purification by column chromatography (SiO_2, Hex/EtOAc = 60:40).

^{1}H NMR (400 MHz, $CDCl_3$) δ 7.99 – 7.94 (m, 2H), 7.66 (m, 2H), 7.59 – 7.37 (m, 6H), 7.35 – 7.26 (m, 4H), 7.25 – 7.14 (m, 6H), 7.08 (td, *J* = 7.5, 1.1 Hz, 1H), 5.93 (dd, *J* = 6.8, 3.4 Hz, 1H), 5.66 – 5.59 (m, 1H), 5.20 (dd, *J* = 10.4, 3.3 Hz, 1H), 4.75 – 4.66 (m, 1H), 4.42 – 4.31 (m, 1H), 4.25 – 4.13 (m, 2H), 4.07 – 3.90 (m, 6H), 1.69 – 1.44 (m, 5H), 1.40 – 1.29 (m, 3H), 1.28 – 1.16 (m, 3H), 1.14 (d, *J* = 6.5 Hz, 3H), 0.90 – 0.83 (m, 4H), 0.78 (td, *J* = 7.4, 2.0 Hz, 4H). **^{13}C NMR** (101 MHz, $CDCl_3$) δ 170.3, 166.1, 154.4, 143.8, 143.2, 141.4, 141.3, 137.5, 133.6, 133.5, 130.1, 129.9, 129.4, 128.7, 128.7, 128.5, 128.4, 128.1, 128.0, 127.9, 127.9, 127.8, 127.3, 127.2, 125.5, 125.3, 120.1, 120.0, 95.6, 95.6, 95.5, 74.1, 73.1, 72.9, 72.9, 72.8, 71.6, 71.2, 70.4, 68.1, 68.0, 68.0, 68.0, 67.7, 67.6, 67.6, 66.9, 46.7, 32.4, 32.3, 32.2, 32.2, 20.9, 18.8, 18.6, 16.1, 16.1, 13.7, 13.7. **^{31}P NMR** (162 MHz, $CDCl_3$) δ -1.83. **HRMS** QTOF-MS: calcd. $C_{43}H_{50}NaO_{11}P$ for $[M+H]^+$ 773.3091, found 773.3095.

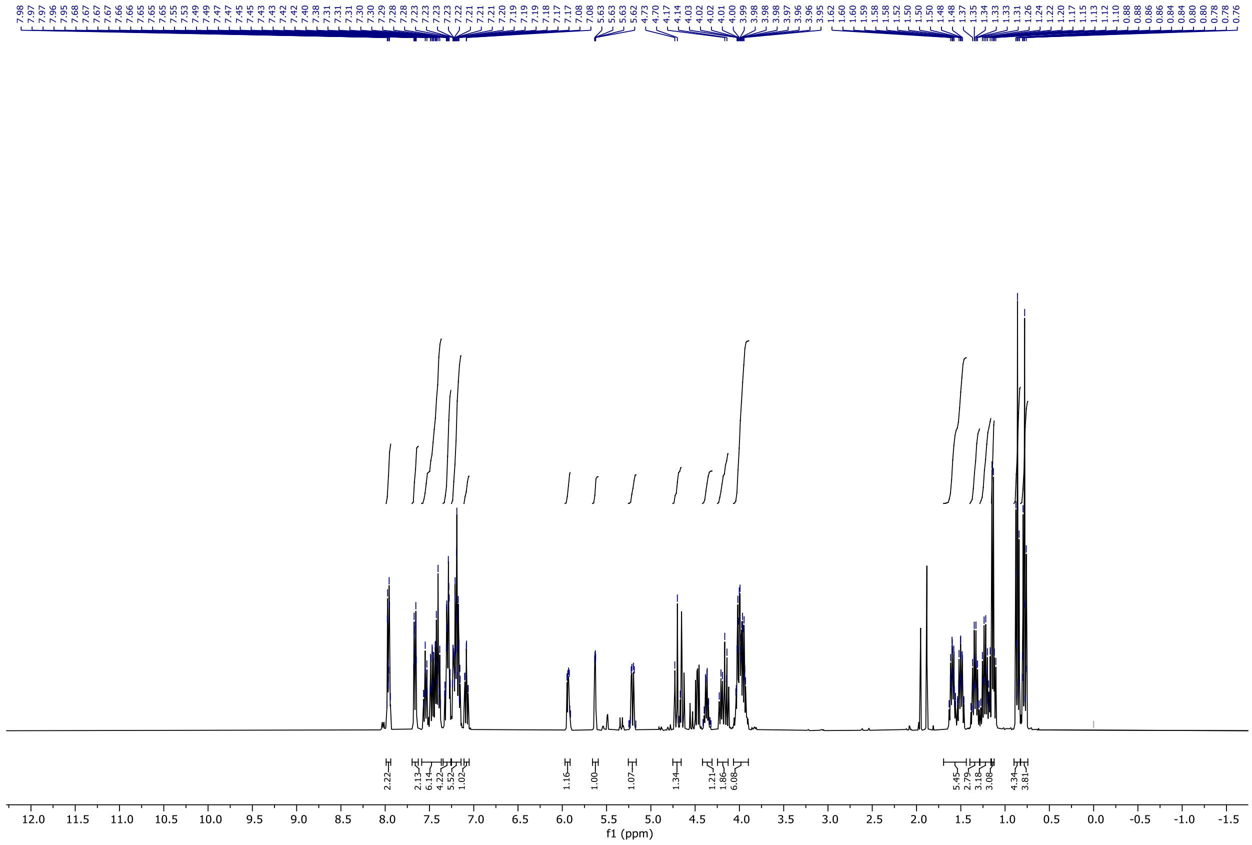

^{1}H NMR

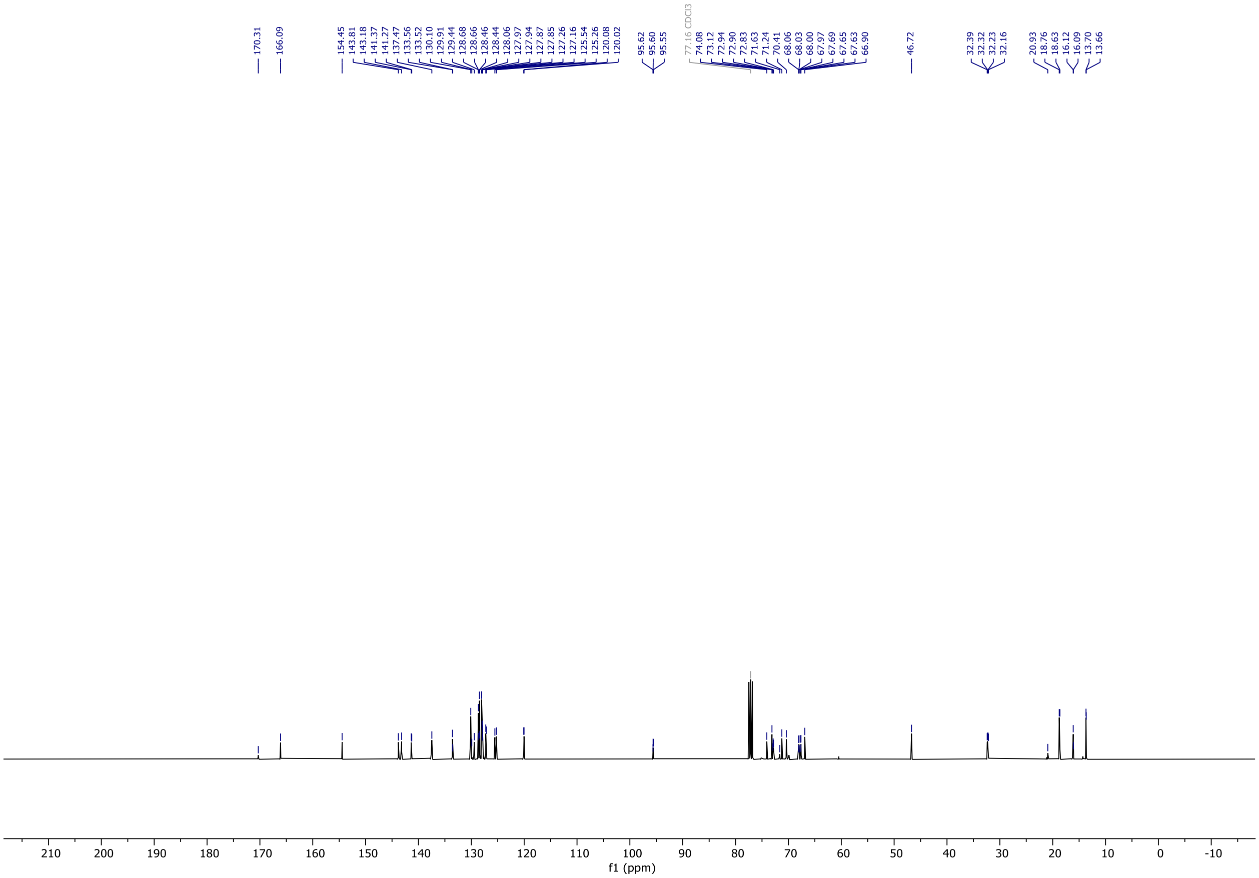

^{13}C NMR

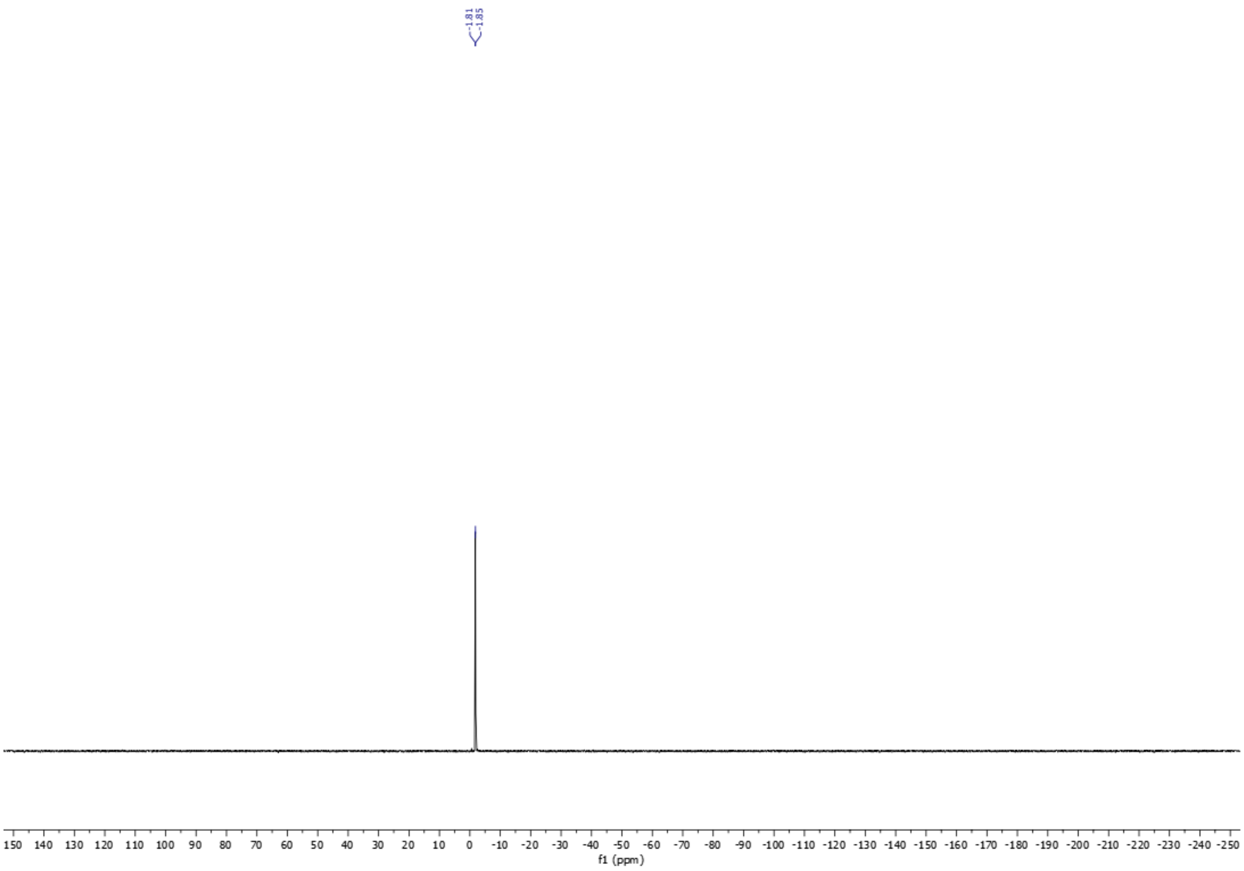
-1.81
-1.85
150 140 130 120 110 100 90 80 70 60 50 40 30 20 10 0 -10 -20 -30 -40 -50 -60 -70 -80 -90 -100 -110 -120 -130 -140 -150 -160 -170 -180 -190 -200 -210 -220 -230 -240 -250
f1 (ppm)

^{31}P NMR

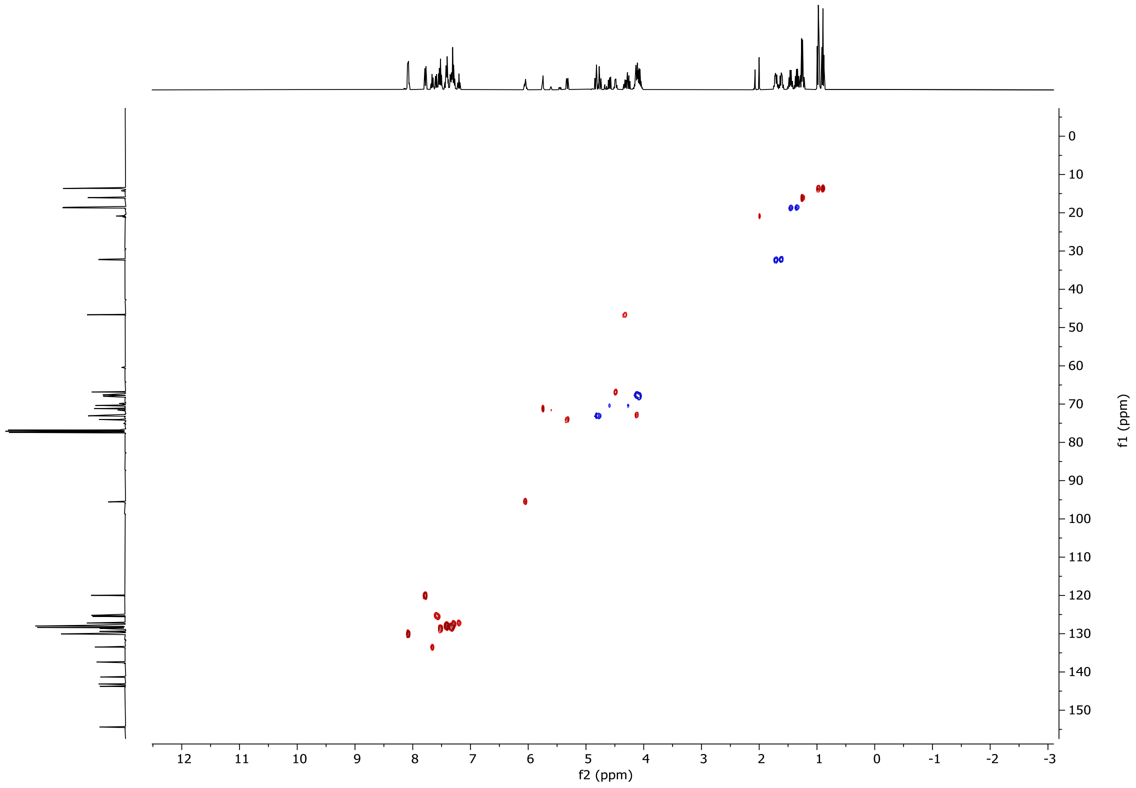

^{1}H-^{13}C HSQC

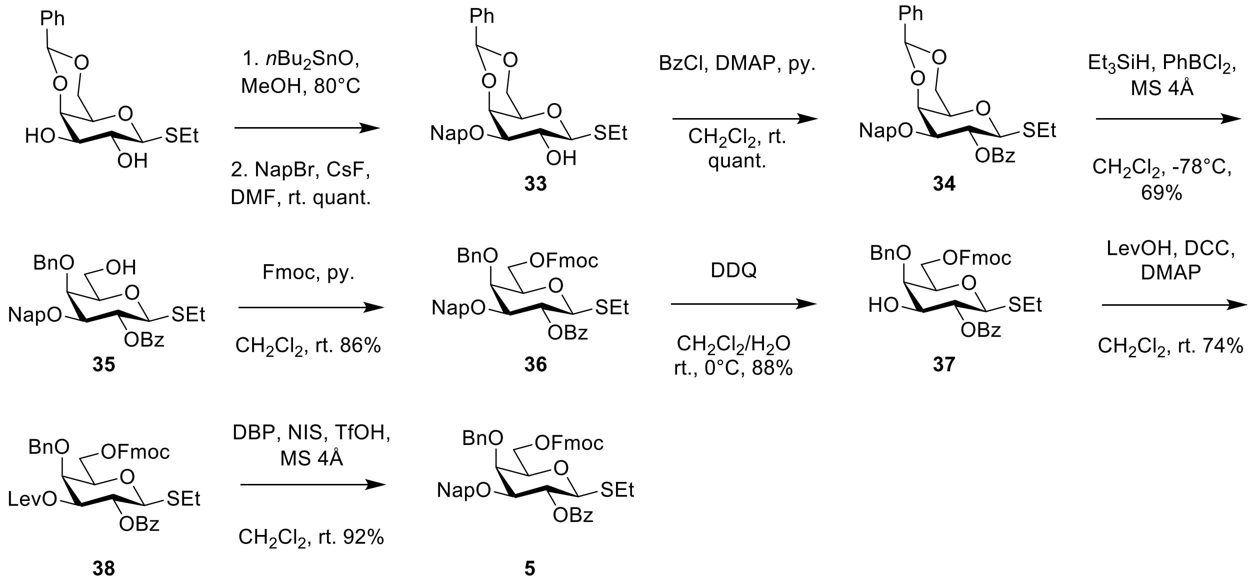

SI Scheme 6. Synthesis of building block **5**.

Ethyl 4,6-*O*-benzylidene-3-*O*-(2-naphthalenylmethyl)-1-thio-β-D-galactoside (33)

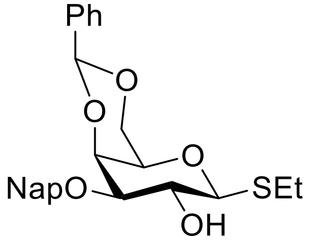

Ethyl 4,6-*O*-benzylidene-1-thio-β-D-galactoside (13g, 41.6 mmol) and dibutyltin (IV) oxide (12.4g, 49.9 mmol, 1.2 eq.) were dissolved in methanol (0.2 M, 208 mL). The mixture was heated to 80 °C in an oil bath and refluxed under an argon atmosphere overnight. Subsequently, the reaction was concentrated under vacuum. The crude material was then re-dissolved in DMF (0.2 M, 208 mL), cooled to 0 °C, and 2-(bromomethyl)naphthalene (13.8 g, 62.4 mmol, 1.5 eq.) and cesium fluoride (19 g, 125 mmol, 3 eq.) were added. The mixture was stirred at room temperature under an argon atmosphere overnight. Once complete, as indicated by TLC analysis (≈16 h), the reaction was concentrated under vacuum. The residue was then re-dissolved in ethyl acetate and successively washed with H_2O, dried over Na_2SO_4, and concentrated. The residue was precipitated in hexane

at 0 °C to give compound **33** (18.8 g, 41.5 mmol, quant.) as white solid. R_f = 0.22 (Hexane/Ethyl acetate, 60:40, v/v).

^{1}H NMR (400 MHz, $CDCl_3$) δ 7.85 – 7.76 (m, 4H, Ar), 7.56 – 7.45 (m, 5H), 7.38 (d, *J* = 4.2 Hz, 1H), 7.37 – 7.35 (m, 2H), 5.44 (s, 1H), 4.35 (d, *J* = 9.6 Hz, 1H), 4.33 – 4.27 (m, 1H), 4.22 – 4.17 (m, 1H), 4.15 – 4.06 (m, 1H), 3.98 – 3.92 (m, 1H), 3.59 – 3.51 (m, 1H), 3.43 – 3.37 (m, 1H), 2.89 – 2.69 (m, 2H), 1.34 (t, *J* = 7.5 Hz, 3H). **^{13}C NMR** (101 MHz, $CDCl_3$) δ 137.9, 135.7, 133.3, 133.2, 129.2, 128.5, 128.4, 128.0, 127.9, 126.9, 126.6, 126.4, 126.2, 125.9, 101.5, 85.5, 80.3, 73.8, 70.3, 69.6, 68.2, 23.1, 15.4. **HRMS** QTOF-MS: calcd. $C_{26}H_{28}NaO_5S$ for $[M+Na]^+$ 475.1555, found 475.1582.

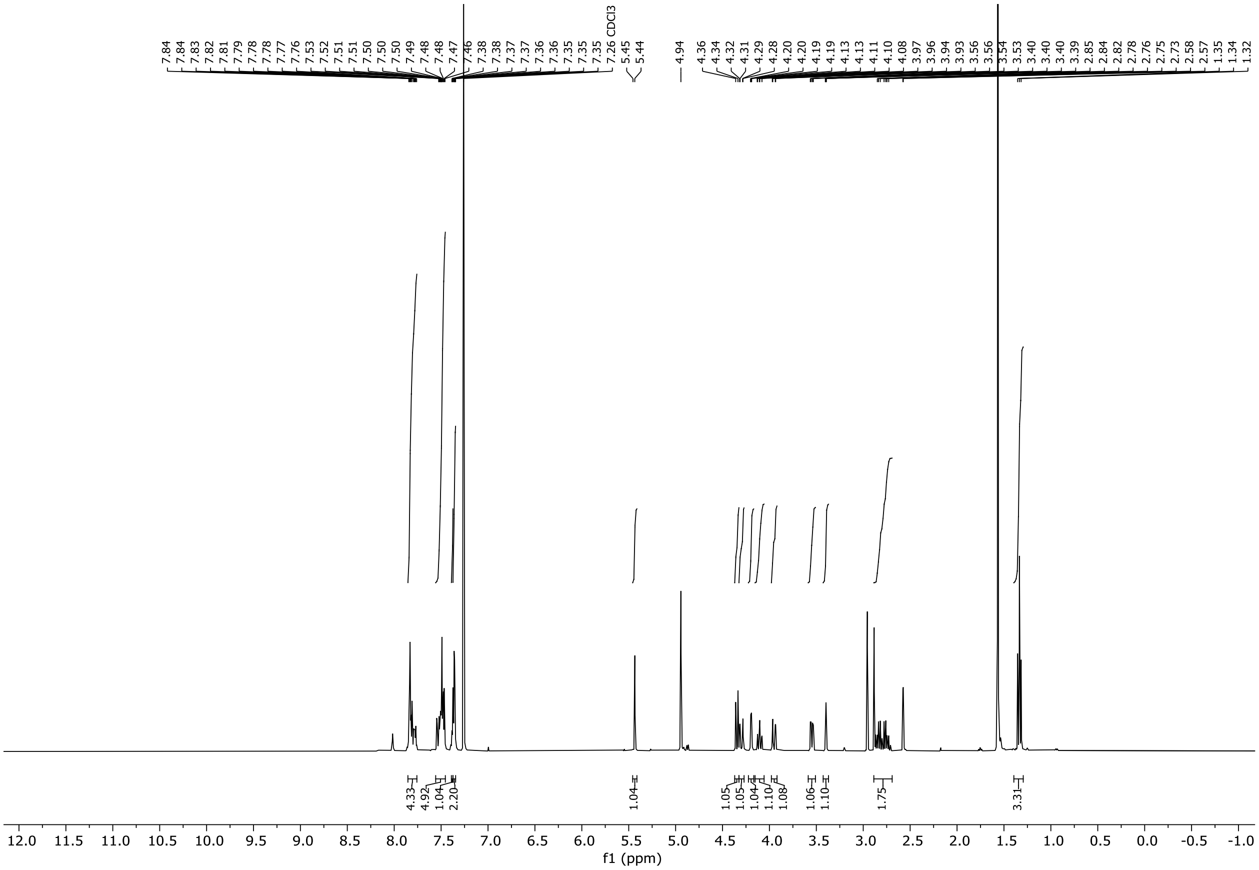

^{1}H NMR

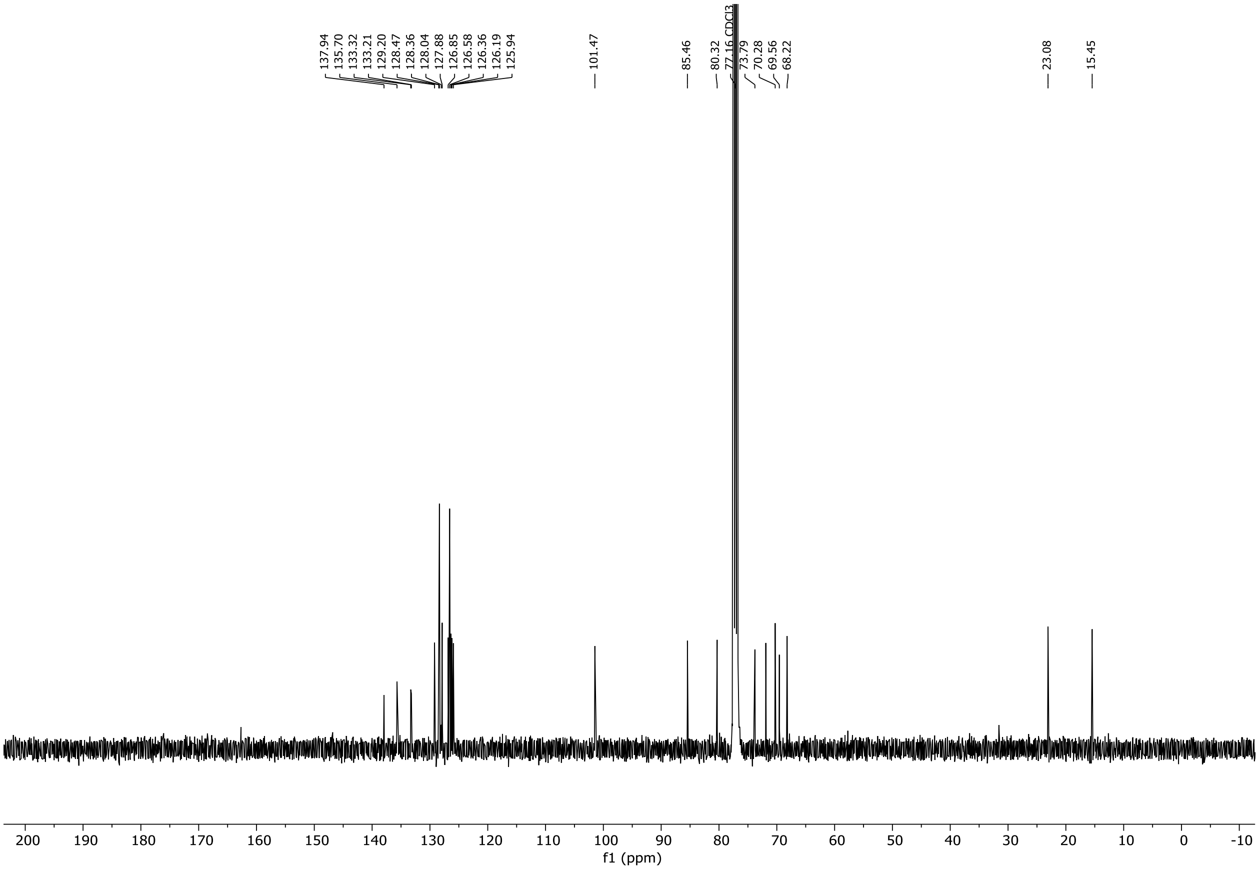
137.94
135.70
133.32
133.21
129.20
128.47
128.36
128.04
127.88
126.85
126.58
126.36
126.19
125.94
101.47
85.46
80.32
77.16 CDCl3
73.79
70.28
69.56
68.22
23.08
15.45
f1 (ppm)

^{13}C NMR

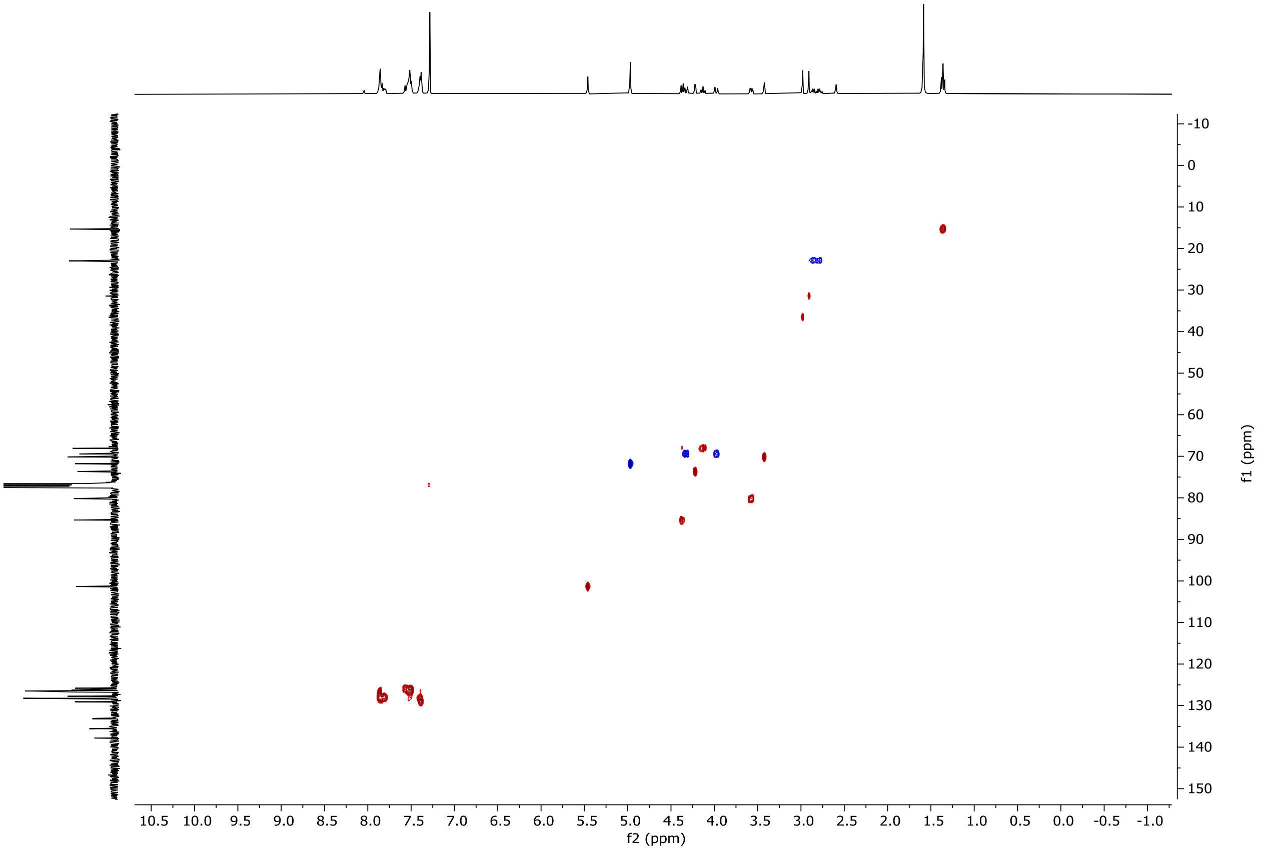

^{1}H-^{13}C HSQC NMR

Ethyl 2-*O*-benzoyl-4,6-*O*-benzylidene-3-*O*-(2-naphthalenylmethyl)-1-thio-β-D-galactoside (34)

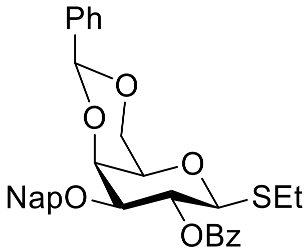

Ethyl 4,6-*O*-benzylidene-3-*O*-(2-naphthalenylmethyl)-1-thio-β-D-galactoside **33** (18.8 g, 41.5 mmol) was dissolved in CH_2Cl_2 (0.2 M, 208 mL) and cooled to 0 °C. Subsequently, benzoyl chloride (9.64 mL, 83 mmol, 2 eq.), pyridine (16.8 mL, 208 mmol, 5 eq.) and 4-dimethylaminopyridine (1.02 g, 8.3 mmol, 0.2 eq.) were added. The mixture was stirred at room temperature under argon atmosphere. Once complete, as indicated by TLC analysis (≈8 h), the reaction was quenched with citric acid (10% w/v) and extracted. The organic layer was washed with saturated aqueous $NaHCO_3$, brine, dried over Na_2SO_4, and concentrated. The residue was precipitated in hexane at 0 °C to give compound **34** (23.1 g, 41.5 mmol, quant.) as light yellow solid. R_f = 0.55 (Hexane/Ethyl acetate, 50:50, v/v).

^{1}H NMR (400 MHz, $CDCl_3$) δ 8.04 – 8.01 (m, 2H), 7.80 – 7.73 (m, 1H), 7.65 – 7.52 (m, 6H), 7.48 – 7.32 (m, 8H), 5.76 (t, *J* = 9.7 Hz, 1H), 4.88 – 4.74 (m, 2H), 4.52 (d, *J* = 9.8 Hz, 1H), 4.39 – 4.26 (m, 2H), 4.01 (m, *J* = 12.3, 1.7 Hz, 1H), 3.78 (m, *J* = 9.6, 3.4 Hz, 1H), 2.92 (m, *J* = 12.3, 7.4 Hz, 1H), 2.77 (m, *J* = 12.3, 7.5 Hz, 1H), 1.27 (t, *J* = 7.5 Hz, 3H). **^{13}C NMR** (101 MHz, $CDCl_3$) δ 165.4, 137.8, 135.4, 133.2, 133.1, 130.2, 130.0, 129.3, 128.5, 128.4, 128.4, 127.9, 127.8, 126.7, 126.3, 126.1, 125.9, 101.6, 83.0, 78.1, 73.5, 71.3, 70.3, 69.5, 68.8, 22.8, 15.0. **HRMS** QTOF-MS: calcd. $C_{33}H_{32}NaO_6S$ for $[M+Na]^+$ 579.1817, found 579.1841.

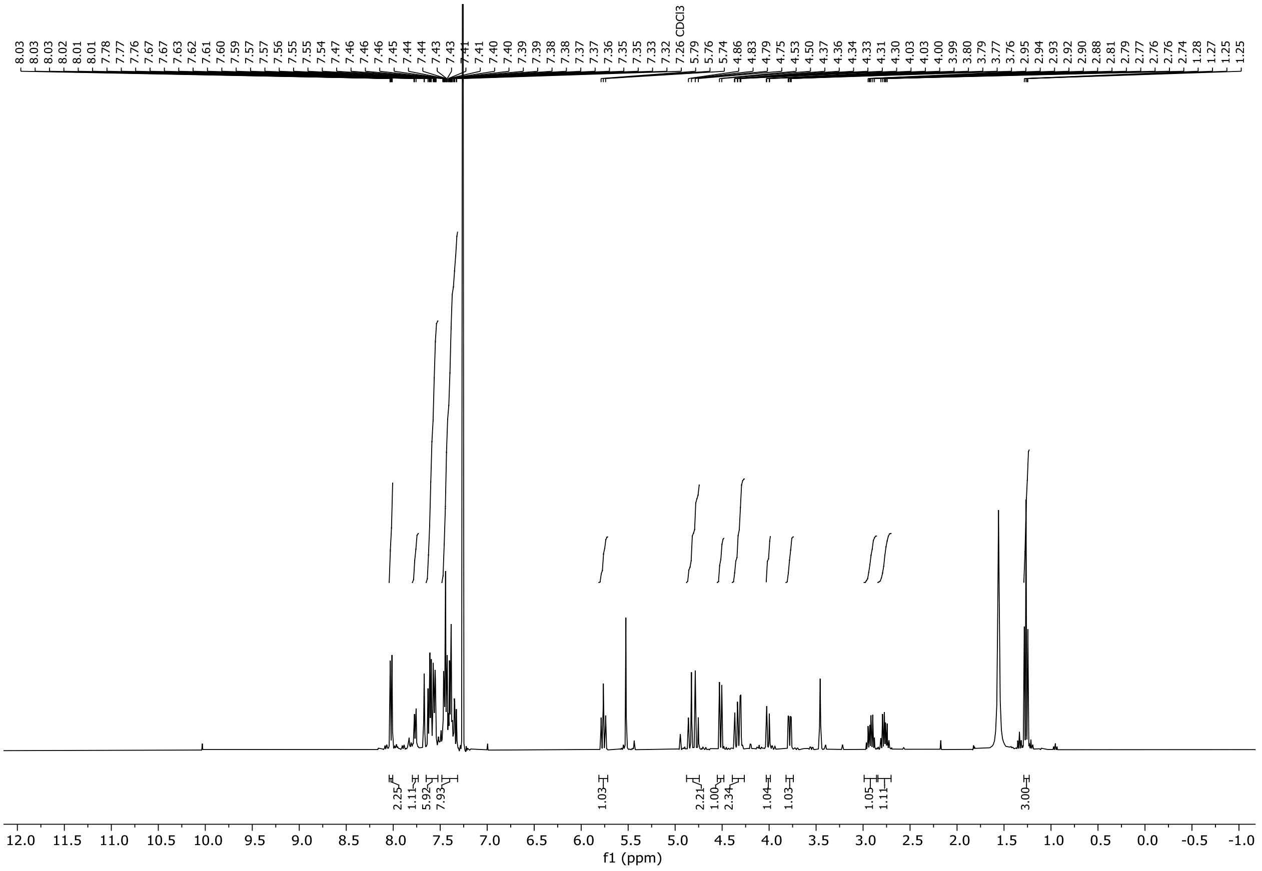

^{1}H NMR

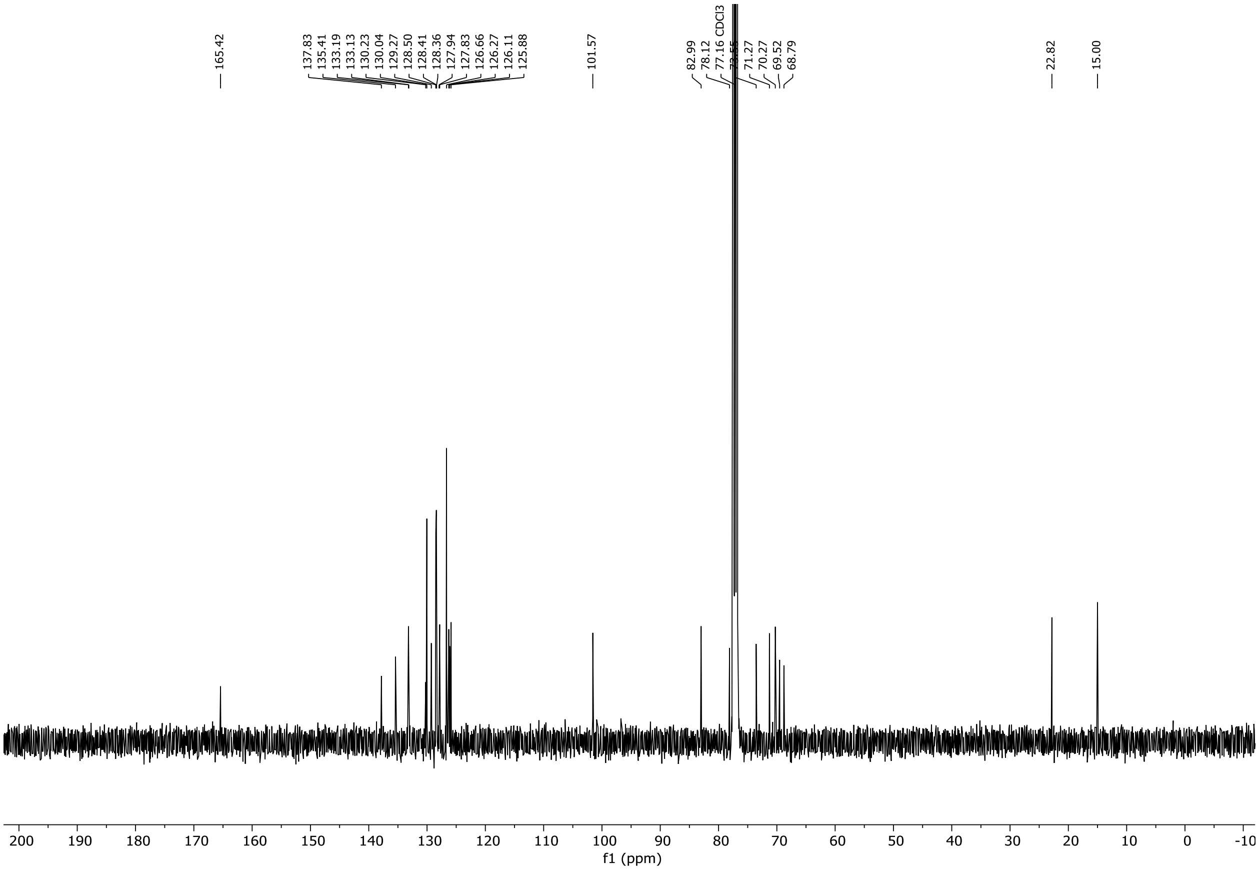

165.42
137.83
135.41
133.19
133.13
130.23
130.04
129.27
128.50
128.41
128.36
127.94
127.83
126.66
126.27
126.11
125.88
101.57
82.99
78.12
77.16 CDCl3
71.27
70.27
69.52
68.79
22.82
15.00
f1 (ppm)

^{13}C NMR

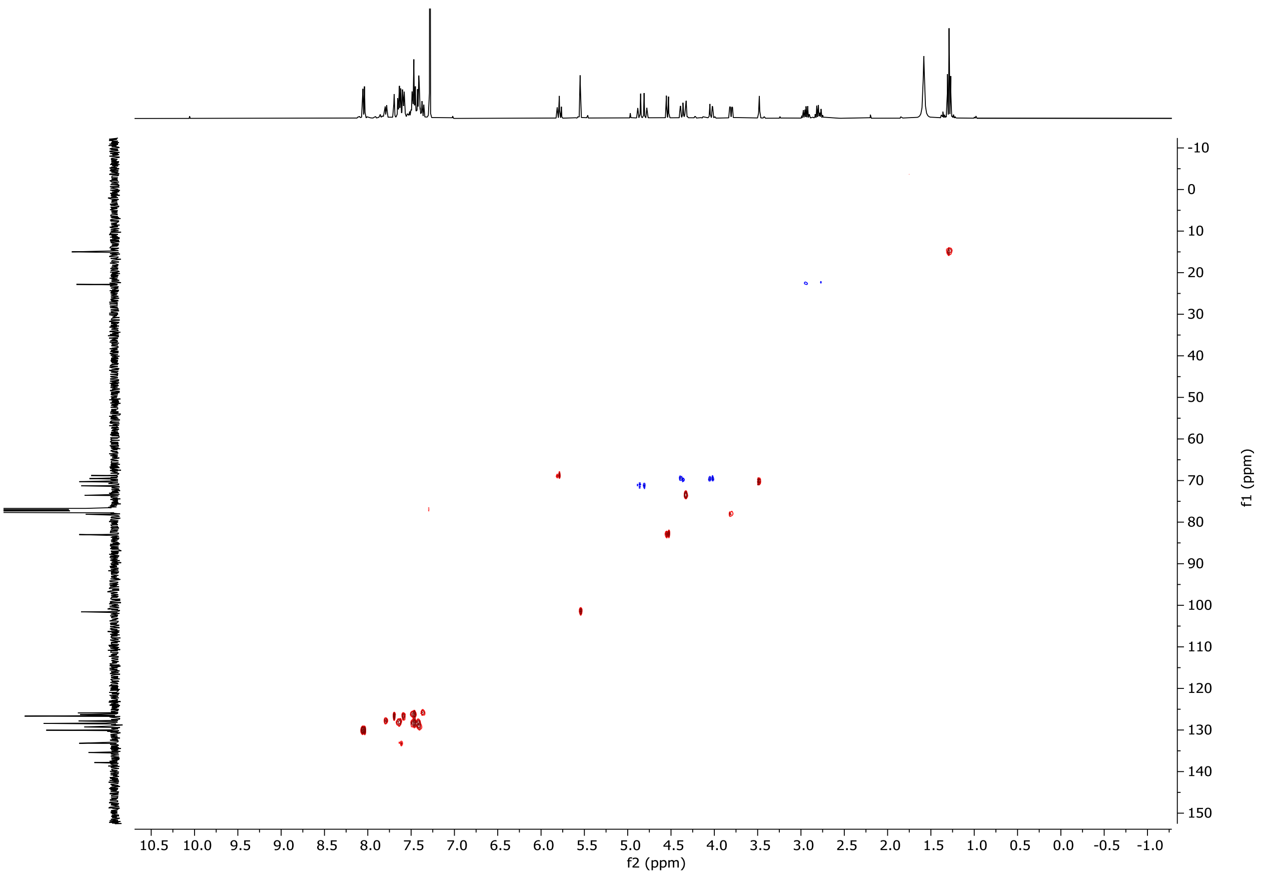

^{1}H-^{13}C HSQC NMR

Ethyl 2-*O*-benzoyl-4-*O*-benzyl-3-*O*-(2-naphthalenylmethyl)-1-thio-β-D-galactoside (35)

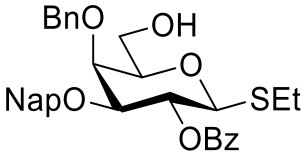

Ethyl 2-*O*-benzoyl-4,6-*O*-benzylidene-3-*O*-(2-naphthalenylmethyl)-1-thio-β-D-galactoside **34** (23.1 g, 41.5 mmol) was dissolved in anhydrous CH_2Cl_2 (0.2 M, 208 mL) and 4Å molecular sieves were added. The mixture was stirred at room temperature under an argon atmosphere for 20 minutes. Subsequently, the mixture was cooled to -78 °C, and triethylsilane (13.2 mL, 83 mmol, 2 eq.) and dichlorophenylborane (10.8 mL, 83 mmol, 2 eq.) were added. The reaction was maintained at -78 °C until the complete, as indicated by TLC analysis (≈20 minutes). The reaction was neutralized with Et_3N (0.4 mL, 2.96 mmol, 0.4 eq.) and the mixture was filtered through a pad of Celite®. The filtrate was washed with saturated aqueous $NaHCO_3$, brine, dried over Na_2SO_4, and concentrated. The residue was purified by flash chromatography (SiO_2, Hexane/Ethyl acetate) to give compound **35** (16 g, 28.6 mmol, 69%) as white solid. R_f =0.44 (Hexane/Ethyl acetate, 50:50, v/v).

^{1}H NMR (400 MHz, $CDCl_3$) δ 8.04 – 7.96 (m, 2H), 7.80 – 7.71 (m, 1H), 7.66 – 7.54 (m, 4H), 7.48 – 7.27 (m, 10H), 5.73 (t, *J* = 9.8 Hz, 1H), 5.05 (d, *J* = 11.9 Hz, 1H), 4.85 (d, *J* = 12.4 Hz, 1H), 4.71 (m, *J* = 12.2, 4.1 Hz, 2H), 4.48 (d, *J* = 9.9 Hz, 1H), 3.97 (m, *J* = 2.8, 1.0 Hz, 1H), 3.84 (m, *J* = 10.9, 6.5 Hz, 1H), 3.74 (m, *J* = 9.6, 2.7 Hz, 1H), 3.59 – 3.46 (m, 2H), 2.72 (m, *J* = 12.4, 7.4, 4.9 Hz, 2H), 1.21 (t, *J* = 7.5 Hz, 3H). **^{13}C NMR** (101 MHz, $CDCl_3$) δ 165.6, 138.3, 135.1, 133.2, 133.2, 133.1, 130.2, 130.0, 128.7, 128.6, 128.5, 128.5, 128.2, 128.0, 127.8, 126.8, 126.3, 126.2, 125.9, 84.0, 81.2, 79.1, 74.3, 72.3, 72.1, 70.3, 62.3, 23.9, 15.0. **HRMS** QTOF-MS: calcd. $C_{33}H_{34}NaO_6S$ for $[M+Na]^+$ 581.1974, found 581.1996.

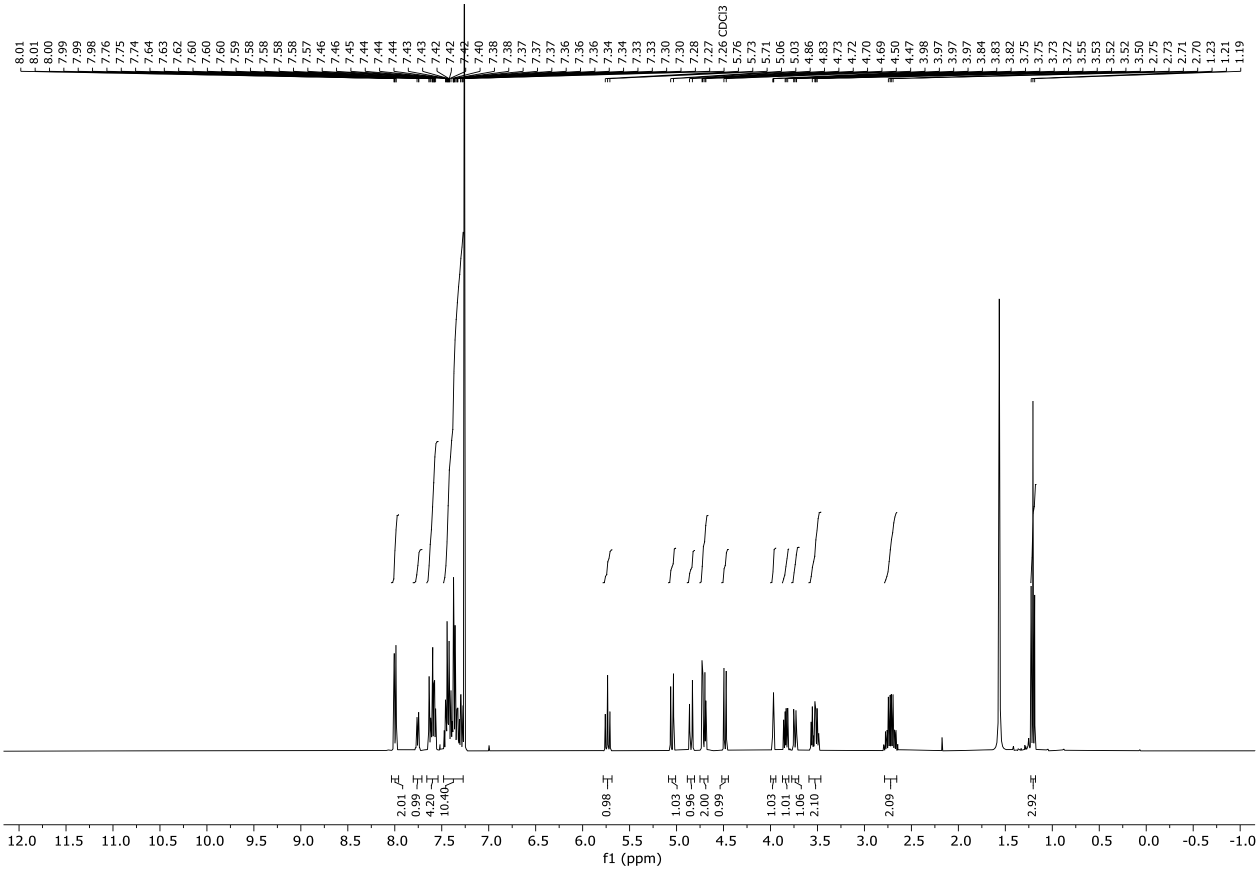

^{1}H NMR

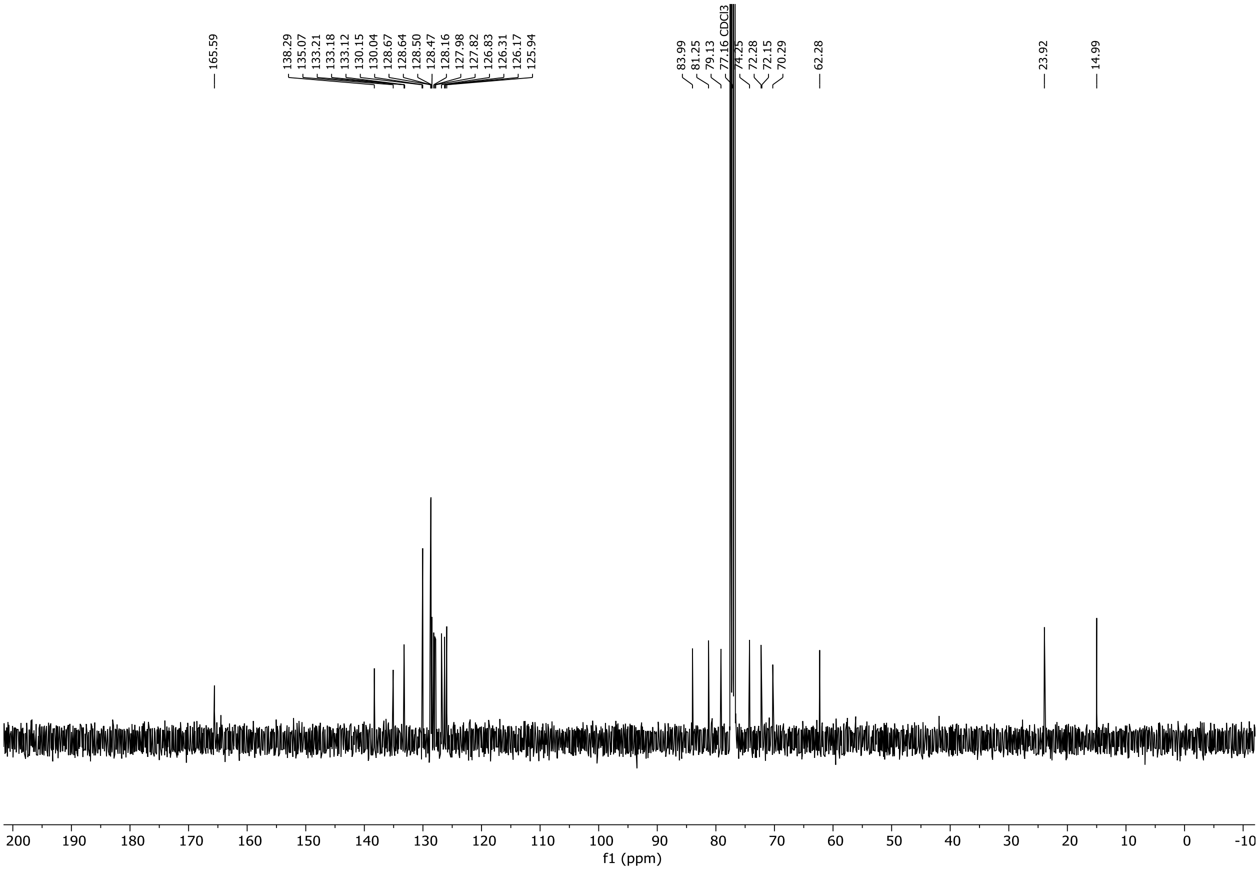
165.59
138.29
135.07
133.21
133.18
133.12
130.15
130.04
128.67
128.64
128.50
128.47
128.16
127.98
127.82
126.83
126.31
126.17
125.94
83.99
81.25
79.13
77.16 CDCl3
74.25
72.28
72.15
70.29
62.28
23.92
14.99
200 190 180 170 160 150 140 130 120 110 100 90 80 70 60 50 40 30 20 10 0 -10
f1 (ppm)

^{13}C NMR

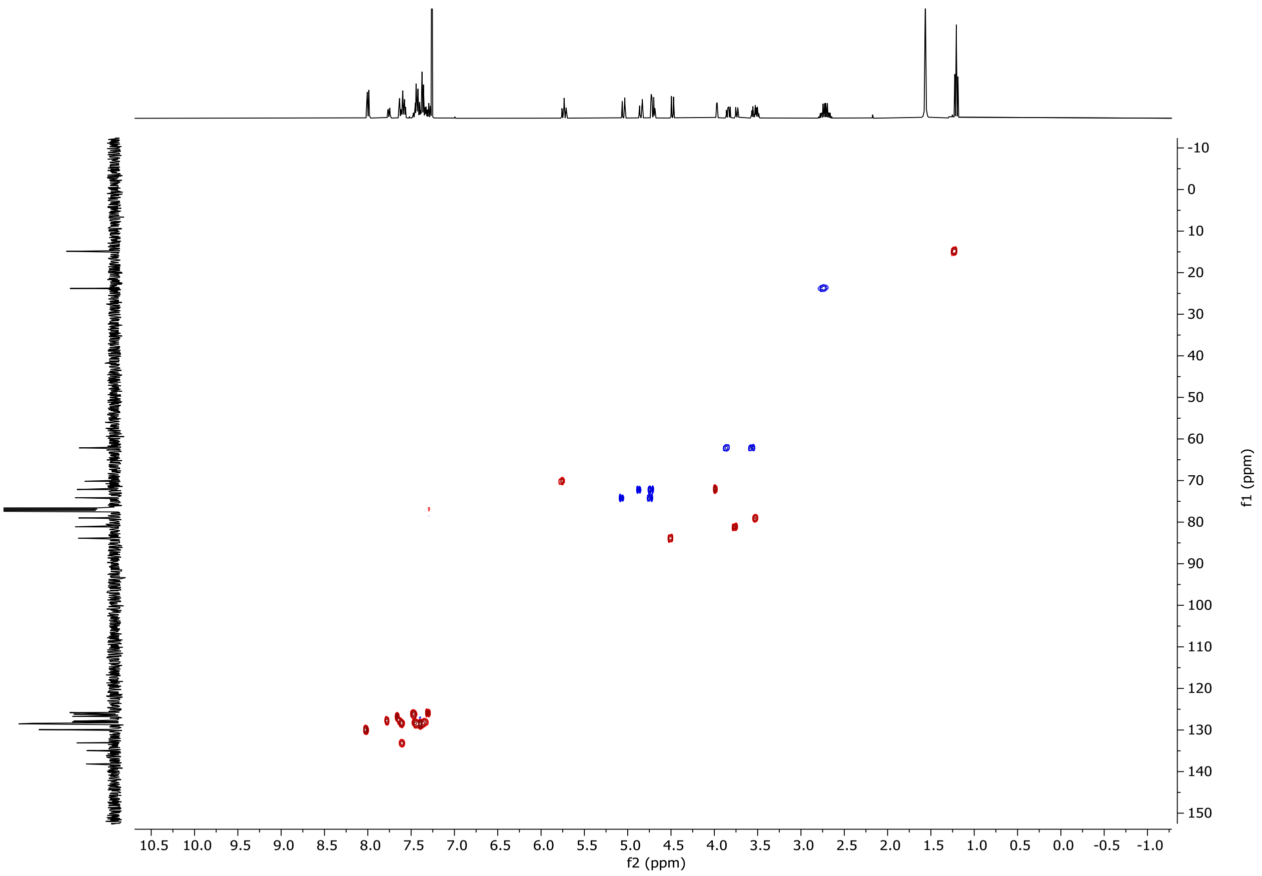

^{1}H-^{13}C HSQC NMR

Ethyl 2-*O*-benzoyl-4-*O*-benzyl-6-*O*-(9-fluorenylmethoxycarbonyl)-3-*O*-(2-naphthalenylmethyl)-1-thio-β-D-galactoside (36)

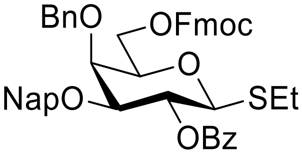

Ethyl 2-*O*-benzoyl-4-*O*-benzyl-3-*O*-(2-naphthalenylmethyl)-1-thio-β-D-galactoside **35** (16 g, 28.6 mmol) was dissolved in CH_2Cl_2 (0.2 M, 143 mL) and cooled to 0 °C. Subsequently, pyridine (23 mL, 286 mmol, 10 eq.) and 9-fluorenylmethoxycarbonylchloride (18.5 g, 71.5 mmol, 2.5 eq.) were added. The mixture was stirred at room temperature overnight. Once complete, as indicated by TLC analysis (≈16 h), the reaction was quenched with citric acid (10% w/v) and extracted. The organic layer was washed with saturated aqueous $NaHCO_3$, brine, dried over Na_2SO_4, and concentrated. The residue was purified by flash chromatography (SiO_2, Hexane/Ethyl acetate) to give compound **36** (19.3 g, 24.7 mmol, 86%) as yellow solid. R_f =0.91 (Hexane/Ethyl acetate, 50:50, v/v).

^{1}H NMR (400 MHz, $CDCl_3$) δ 8.02 – 7.97 (m, 2H), 7.78 (d, *J* = 7.5 Hz, 2H), 7.76 – 7.73 (m, 1H), 7.64 – 7.56 (m, 6H), 7.47 – 7.27 (m, 15H), 5.74 (t, *J* = 9.8 Hz, 1H), 5.07 (d, *J* = 11.7 Hz, 1H), 4.85 (d, *J* = 12.4 Hz, 1H), 4.71 (m, *J* = 12.0, 10.9 Hz, 2H), 4.50 (d, *J* = 9.9 Hz, 1H), 4.25 (m, *J* = 7.3 Hz, 1H), 4.19 (m, *J* = 11.1, 5.8 Hz, 1H), 4.02 (m, *J* = 2.8, 1.1 Hz, 1H), 3.79 – 3.69 (m, 2H), 2.82 – 2.62 (m, 2H), 1.21 (t, *J* = 7.4 Hz, 3H). **^{13}C NMR** (101 MHz, $CDCl_3$) δ 165.5, 154.9, 143.4, 143.4, 141.4, 138.1, 135.0, 133.2, 133.2, 133.1, 130.1, 130.0, 128.5, 128.5, 128.5, 128.4, 128.1, 128.0, 127.8, 127.3, 126.8, 126.3, 126.1, 125.9, 125.3, 120.3, 84.0, 81.0, 76.2, 74.5, 72.4, 72.3, 70.2, 70.1, 66.8, 46.8, 24.1, 15.0. **HRMS** QTOF-MS: calcd. $C_{48}H_{44}NaO_8S$ for $[M+Na]^+$ 803.2655, found 803.2690.

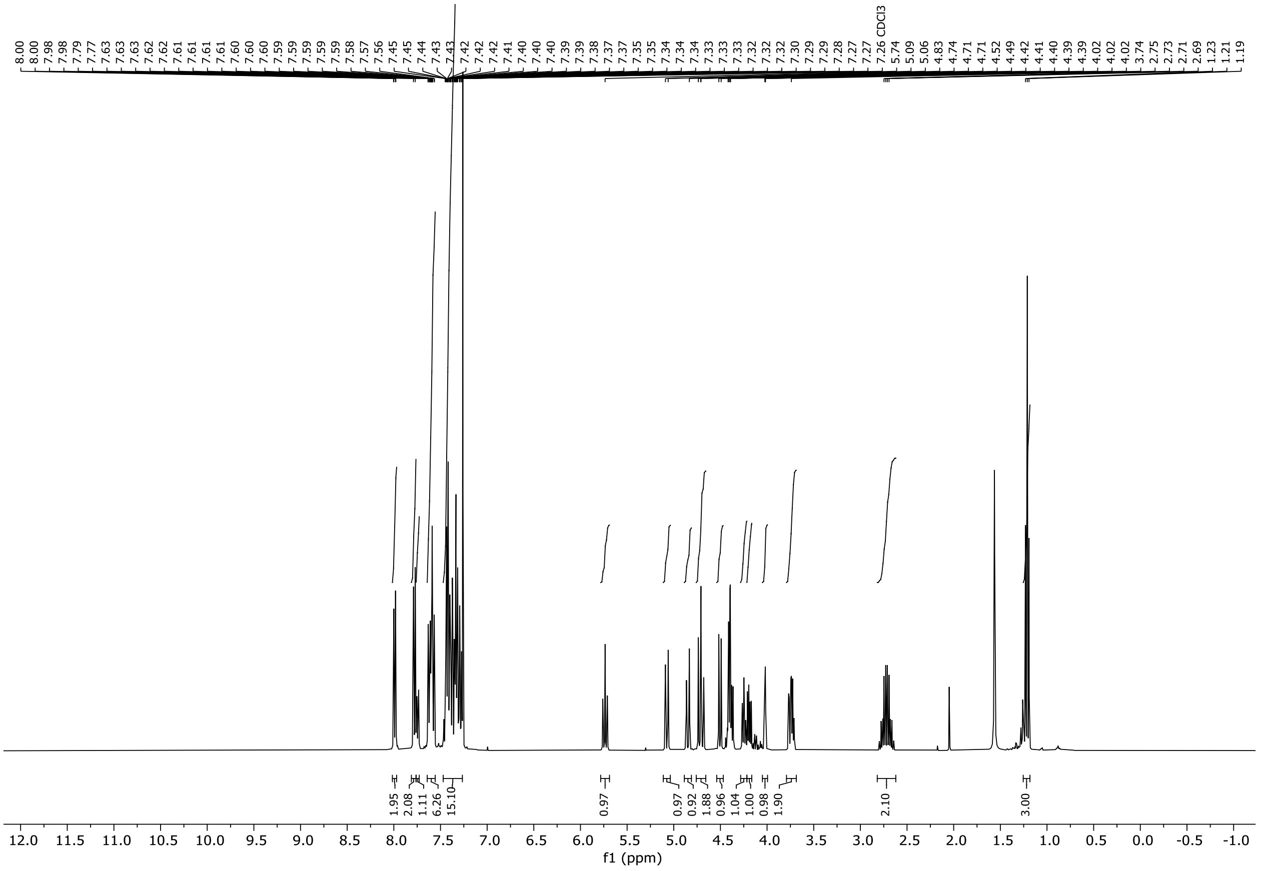

^{1}H NMR

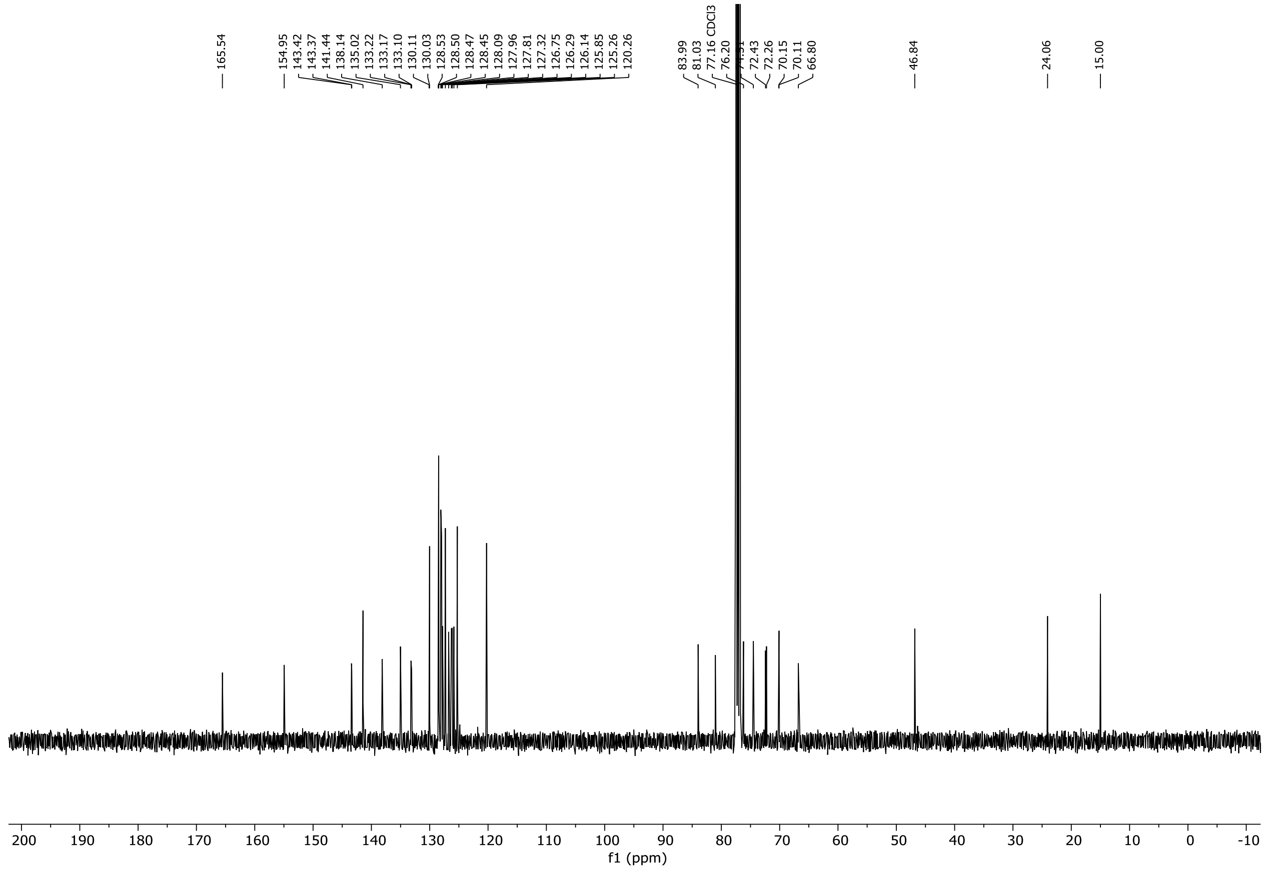

^{13}C NMR

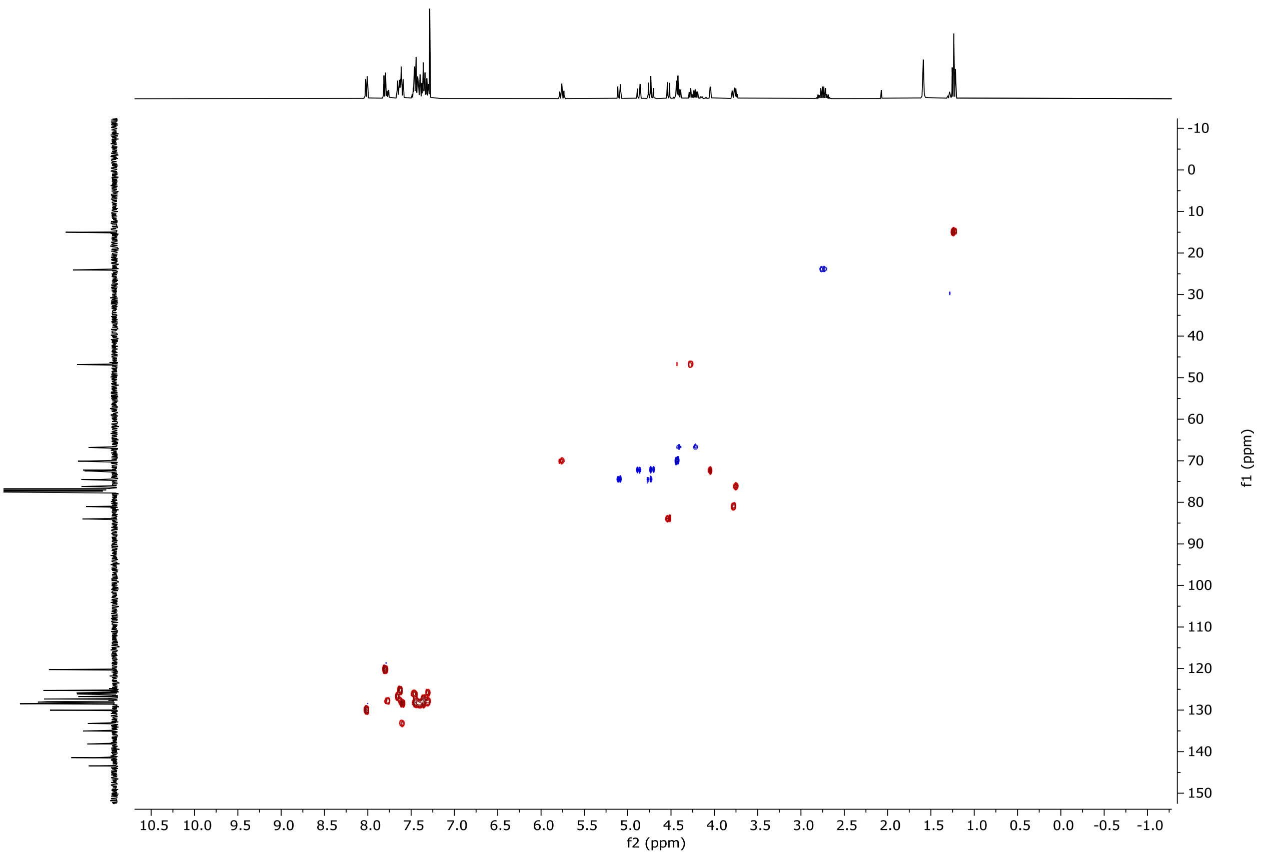

^{1}H-^{13}C HSQC NMR

Ethyl 2-*O*-benzoyl-4-*O*-benzyl-6-*O*-(9-fluorenylmethoxycarbonyl)-1-thio-β-D-galactoside (37)

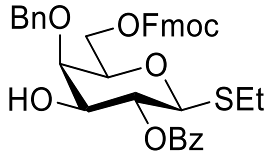

Ethyl 2-*O*-benzoyl-4-*O*-benzyl-6-*O*-(9-fluorenylmethoxycarbonyl)-3-*O*-(2-naphthalenylmethyl)-1-thio-β-D-galactoside **36** (19.3 g, 24.7 mmol) was dissolved in CH_2Cl_2/H_2O (0.2 M, 90:10, v/v, 124 mL) and cooled to 0 °C. Subsequently, 2,3-dichloro-5,6-dicyano-1,4-benyoquinone (16.8 g, 74.1 mmol, 3 eq.) were added. The mixture was kept in the dark, vigorously stirred at 0 °C for the first 2 hours and then at room temperature until the reaction was complete, as indicated by TLC analysis (≈1 h). The reaction was quenched by the addition of a 1:1 solution of 10% aq. $Na_2S_2O_3$ and $NaHCO_3$ (120 mL), and stirred for 10 minutes. The organic layer was then washed with saturated aqueous $NaHCO_3$, brine, dried over Na_2SO_4, and concentrated. The residue was purified by flash chromatography (SiO_2, Hexane/Ethyl acetate) to give compound **37** (14 g, 21.8 mmol, 88%) as white solid. R_f =0.4 (Hexane/Ethyl acetate, 60:40, v/v).

^{1}H NMR (400 MHz, $CDCl_3$) δ 8.10 – 8.03 (m, 2H), 7.78 (m, *J* = 7.5, 1.8, 0.9 Hz, 2H), 7.65 – 7.54 (m, 3H), 7.50 – 7.28 (m, 12H), 5.32 (t, *J* = 9.7 Hz, 1H), 4.78 (d, *J* = 1.4 Hz, 2H), 4.58 (d, *J* = 9.9 Hz, 1H), 4.51 – 4.37 (m, 3H), 4.30 – 4.14 (m, 2H), 3.91 (m, *J* = 3.4, 1.1 Hz, 1H), 3.81 (m, *J* = 6.5, 1.2 Hz, 1H), 2.83 – 2.62 (m, 2H), 1.25 (t, *J* = 7.5 Hz, 4H). **^{13}C NMR** (101 MHz, $CDCl_3$) δ 166.9, 154.9, 143.4, 143.3, 141.5, 137.7, 133.5, 130.1, 129.7, 128.8, 128.6, 128.3, 128.1, 127.3, 127.3, 125.2, 125.2, 120.3, 83.6, 76.5, 76.1, 75.8, 74.5, 72.4, 70.1, 66.2, 46.9, 24.2, 15.1. **HRMS** QTOF-MS: calcd. $C_{37}H_{36}NaO_8S$ for $[M+Na]^+$ 663.2029, found 663.2045.

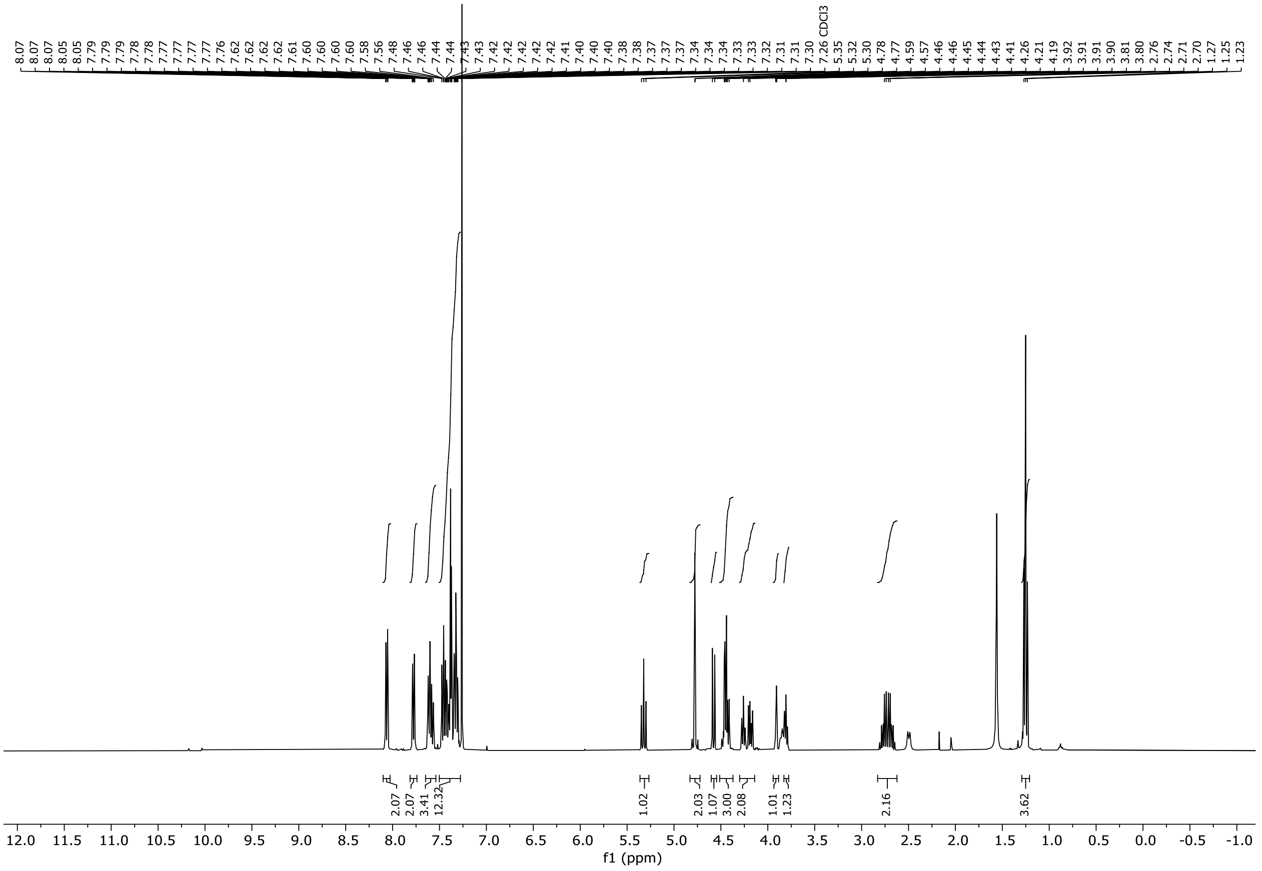

¹H NMR

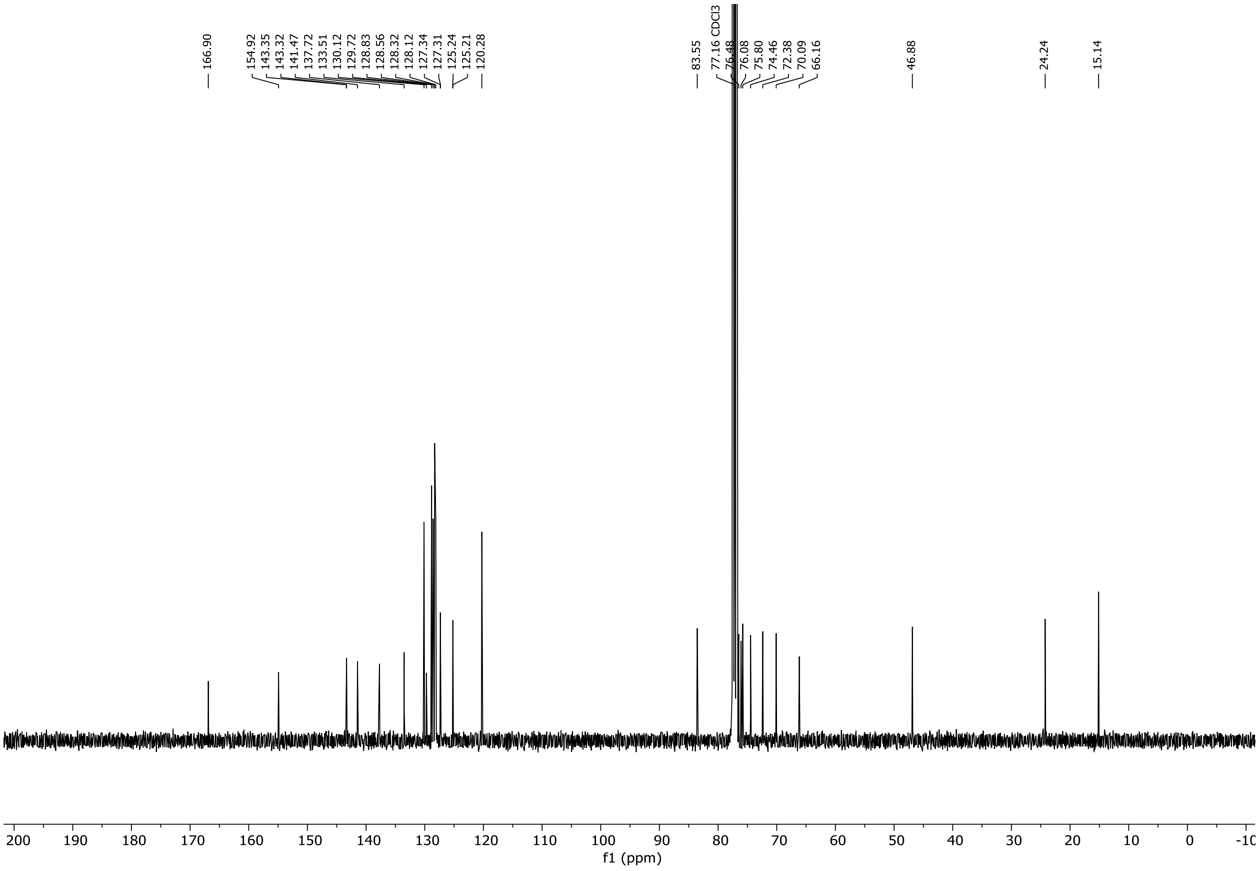
166.90
154.92
143.35
143.32
141.47
137.72
133.51
130.12
129.72
128.83
128.56
128.32
128.12
127.34
127.31
125.24
125.21
120.28
83.55
77.16 CDCl3
76.48
76.08
75.80
74.46
72.38
70.09
66.16
46.88
24.24
15.14
f1 (ppm)

^{13}C NMR

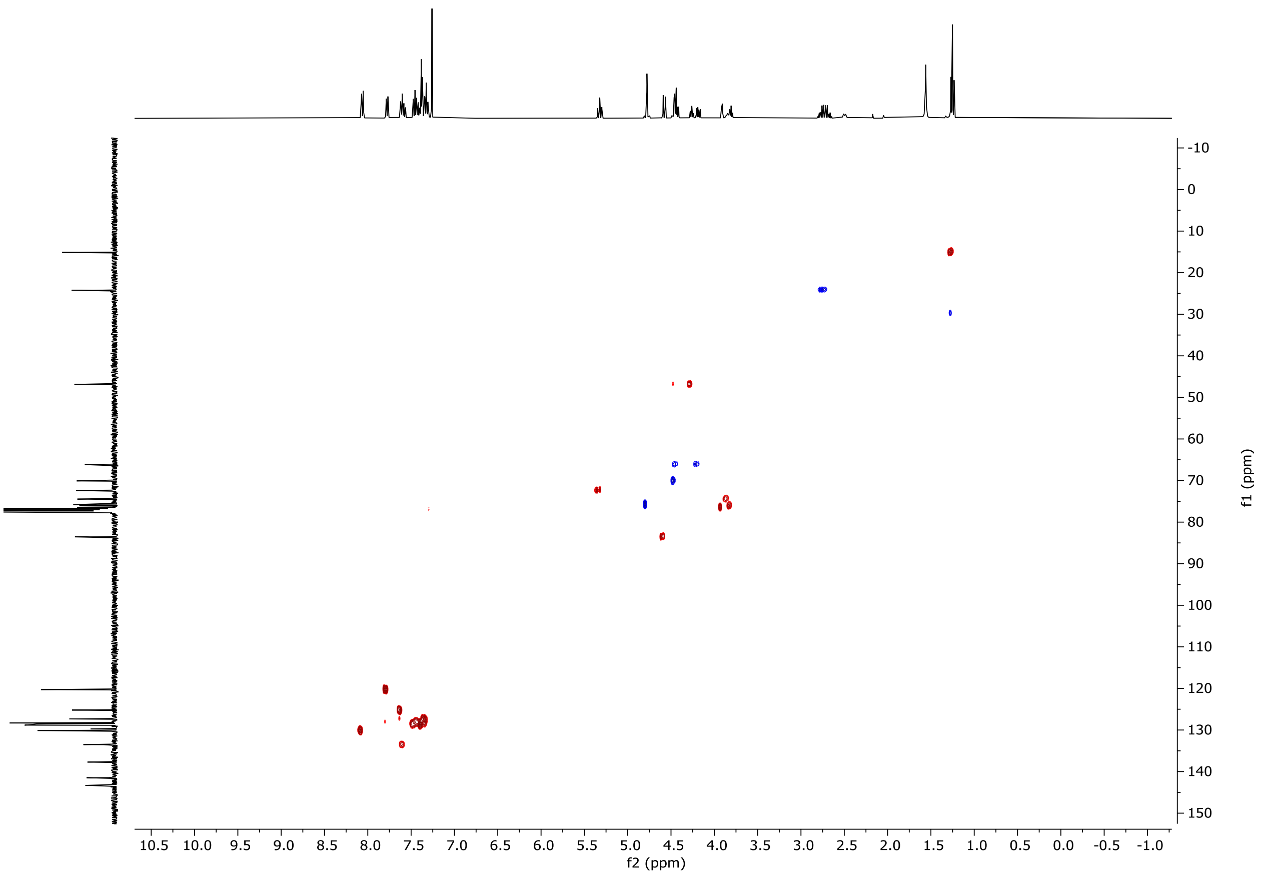

^{1}H-^{13}C HSQC NMR

Ethyl 2-*O*-benzoyl-4-*O*-benzyl-6-*O*-(9-fluorenylmethoxycarbonyl)-3-*O*-levulinyl-1-thio-β-D-galactoside (38)

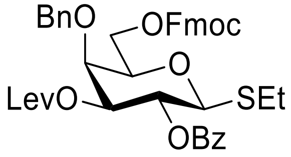

Ethyl 2-*O*-benzoyl-4-*O*-benzyl-6-*O*-(9-fluorenylmethoxycarbonyl)-1-thio-β-D-galactoside **37** (14g, 21.8 mmol) was dissolved in CH_2Cl_2 (0.2 M, 109 mL) and cooled to 0 °C. Subsequently, 4-dimethylaminopyridine (0.26 g, 2.18 mmol, 0.1 eq.), 1-ethyl-3-(3-dimethylaminopropyl)carbodiimide hydrochloride (12.5 g, 65.4 mmol, 3 eq.) and levulinic acid (4.48 mL, 42.6 mmol, 2 eq.) were added. The mixture was stirred at room temperature until the reaction was complete, as indicated by TLC analysis (≈3 h). The reaction was neutralized with citric acid (10% w/v) and extracted. The organic layer was washed with saturated aqueous $NaHCO_3$, brine, dried over Na_2SO_4, and concentrated. The residue was purified by flash chromatography (SiO_2, Hexane/Ethyl acetate) to give compound **38** (12 g, 16.2 mmol, 74%) as transparent solid. R_f =0.57 (Hexane/Ethyl acetate, 60:40, v/v).

^{1}H NMR (400 MHz, $CDCl_3$) δ 8.06 – 7.99 (m, 2H), 7.79 (d, *J* = 7.5 Hz, 2H), 7.65 – 7.55 (m, 3H), 7.50 – 7.28 (m, 13H), 5.70 (t, *J* = 9.9 Hz, 1H), 5.24 (m, *J* = 10.0, 3.0 Hz, 1H), 4.88 (d, *J* = 11.7 Hz, 1H), 4.62 (m, *J* = 10.8, 5.8 Hz, 2H), 4.49 – 4.34 (m, 3H), 4.26 (m, *J* = 7.3 Hz, 1H), 4.12 (m, *J* = 11.0, 6.2 Hz, 1H), 3.87 (m, *J* = 6.3 Hz, 1H), 2.85 – 2.58 (m, 3H), 2.57 – 2.44 (m, 2H), 2.40 – 2.28 (m, 1H), 2.03 (s, 3H), 1.24 (t, *J* = 7.4 Hz, 3H). **^{13}C NMR** (101 MHz, $CDCl_3$) δ 206.1, 172.3, 165.5, 154.9, 143.4, 143.3, 141.4, 137.8, 133.5, 130.0, 129.6, 128.6, 128.3, 128.1, 128.0, 127.3, 127.3, 125.3, 125.3, 120.2, 84.0, 76.0, 75.1, 75.1, 74.0, 70.1, 68.6, 66.1, 46.8, 37.8, 29.7, 28.1, 24.3, 15.0. **HRMS** QTOF-MS: calcd. $C_{42}H_{42}NaO_{10}S$ for $[M+Na]^+$ 761.2396, found 761.2423.

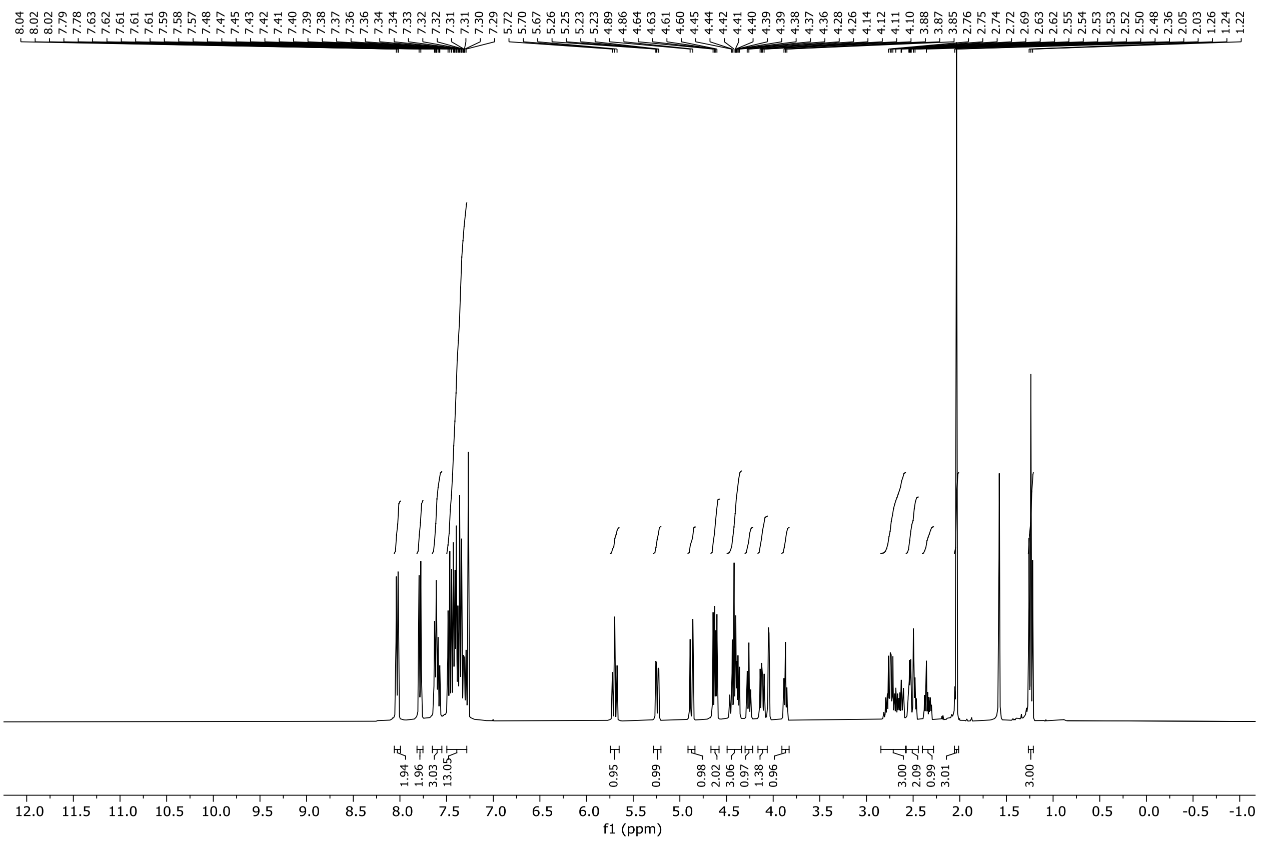

^{1}H NMR

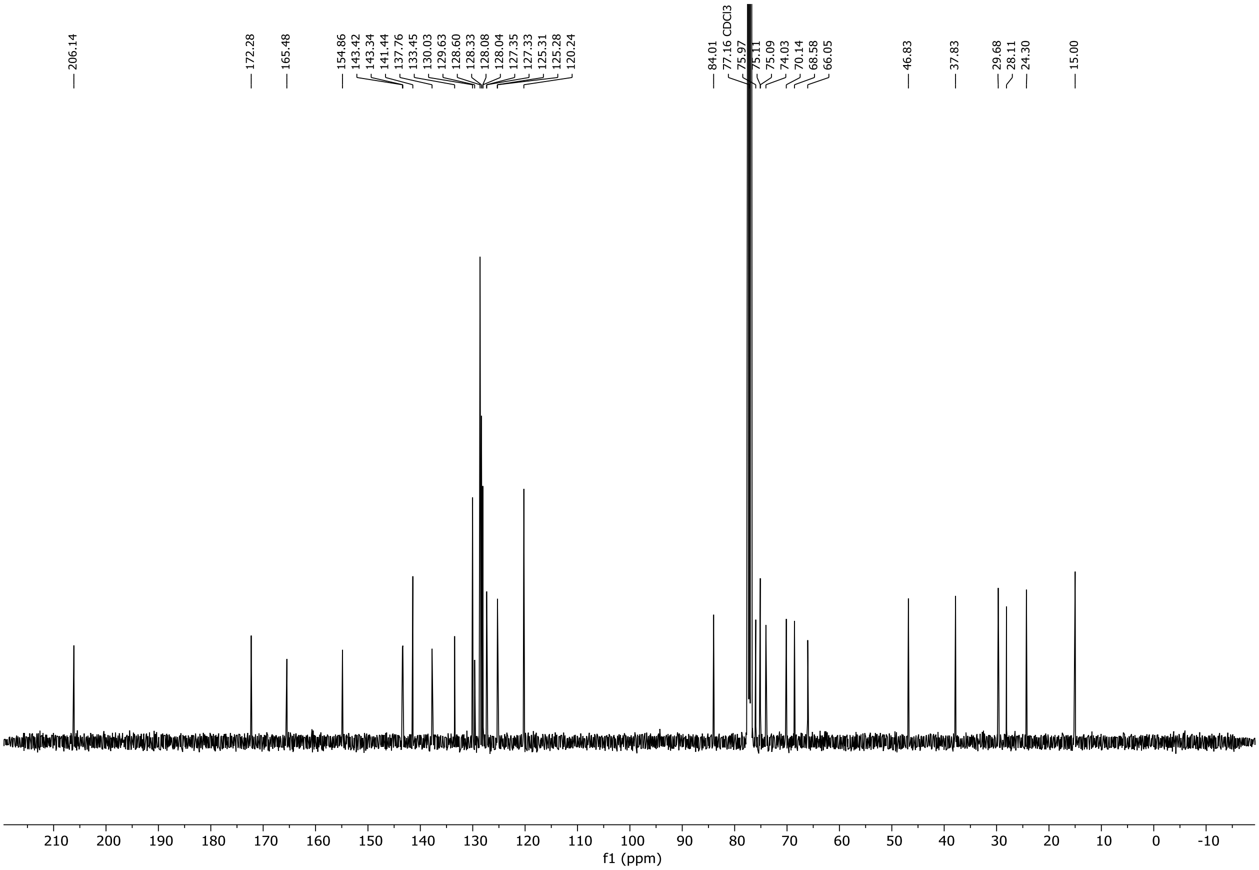
206.14
172.28
165.48
154.86
143.42
143.34
141.44
137.76
133.45
130.03
129.63
128.60
128.33
128.08
128.04
127.35
127.33
125.31
125.28
120.24
84.01
77.16 CDCl3
75.97
75.11
75.09
74.03
70.14
68.58
66.05
46.83
37.83
29.68
28.11
24.30
15.00
f1 (ppm)

^{13}C NMR

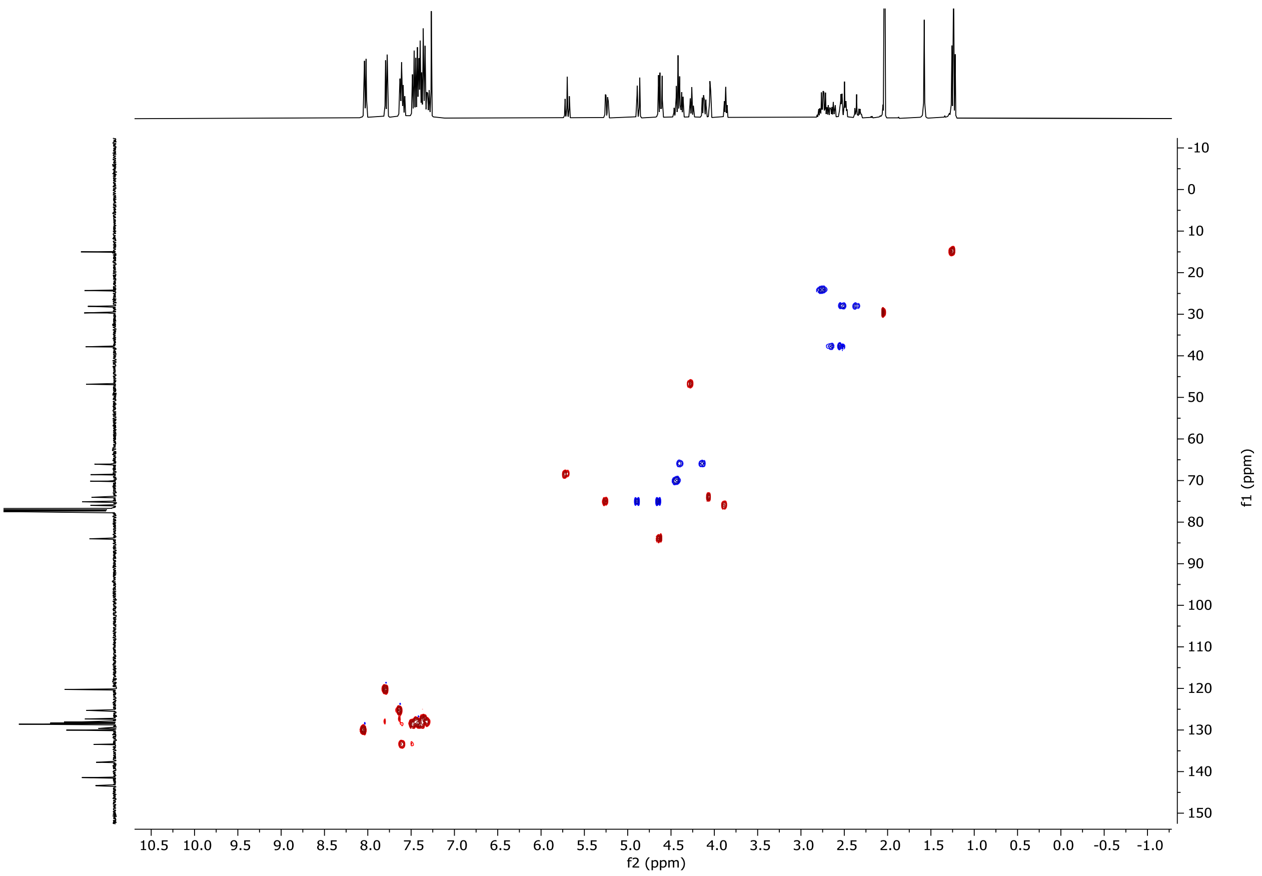

^{1}H-^{13}C HSQC NMR

Dibutyl 2-*O*-benzoyl-4-*O*-benzyl-6-*O*-(9-fluorenylmethoxycarbonyl)-3-*O*-levulinyl-1-phosphate-α,β-D-galactoside (5)

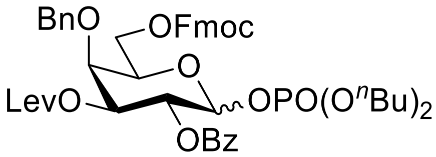

Ethyl 2-*O*-benzoyl-4-*O*-benzyl-6-*O*-(9-fluorenylmethoxycarbonyl)-3-*O*-levulinyl-1-thio-β-D-galactoside **38** (9 g, 12.2 mmol) was dissolved in anhydrous CH_2Cl_2 (0.1 M, 122 mL) and 4Å molecular sieves were added. The mixture was stirred at room temperature under an argon atmosphere for 20 minutes. Subsequently, the mixture was cooled to 0 °C, and *N*-iodosuccinimide (4.49 mL, 22 mmol, 1.8 eq.) and trifluoromethanesulfonic acid (0.22 mL, 2.44 mmol, 0.2 eq.) were added. The mixture was stirred at the same temperature until the reaction was complete, as indicated by TLC analysis (≈20 minutes). Once complete, the reaction was filtered through a pad of Celite®. The filtrate was neutralized by the addition of a 1:1 solution of 10% aq. $Na_2S_2O_3$ and $NaHCO_3$ (120 mL), and stirred for 10 minutes. The organic layer was then washed with saturated aqueous $NaHCO_3$, brine, dried over Na_2SO_4, and concentrated. The residue was purified by flash chromatography (SiO_2, Hexane/Ethyl acetate) to give compound **5** (10 g, 11.3 mmol, 92%) as transparent viscous gel. R_f =0.44 (Hexane/Ethyl acetate, 50:50, v/v).

^{1}H NMR (400 MHz, $CDCl_3$) δ 8.09 – 8.00 (m, 2H), 7.78 (m, *J* = 7.6, 1.0 Hz, 2H), 7.65 – 7.53 (m, 3H), 7.50 – 7.25 (m, 12H), 5.76 (m, *J* = 10.5, 8.0 Hz, 1H), 5.44 (t, *J* = 7.7 Hz, 1H), 5.20 (m, *J* = 10.5, 2.9 Hz, 1H), 4.88 (d, *J* = 11.7 Hz, 1H), 4.62 (d, *J* = 11.6 Hz, 1H), 4.43 – 4.38 (m, 2H), 4.36 – 4.28 (m, 1H), 4.24 (t, *J* = 7.3 Hz, 1H), 4.18 (m, *J* = 11.0, 6.3 Hz, 1H), 4.11 – 3.92 (m, 4H), 3.81 – 3.62 (m, 2H), 2.68 (m, *J* = 20.6, 10.8, 5.4 Hz, 1H), 2.59 – 2.47 (m, 2H), 2.04 (s, 3H), 1.69 – 1.52 (m, 2H), 1.43 – 1.21 (m, 4H), 1.08 – 0.95 (m, 2H), 0.87 (t, *J* = 7.4 Hz, 3H), 0.68 (t, *J* = 7.4 Hz, 3H). **^{13}C NMR** (101 MHz, $CDCl_3$) δ 206.1, 172.2, 165.3, 154.8, 143.3, 141.4, 137.5, 133.6, 130.0, 129.3, 129.2, 128.7, 128.5, 128.4, 128.2, 128.1, 127.4, 125.4, 125.3, 125.3, 120.2, 96.8, 75.3, 73.5, 73.0, 70.2, 70.0, 70.0, 68.1, 68.0, 68.0, 65.5, 46.8, 37.8, 32.2, 32.1, 31.9, 31.8, 29.7, 28.0, 21.6, 18.7, 18.4,

13.7, 13.5. **^{31}P NMR** (162 MHz, $CDCl_3$) δ -2.80 (d, *J* = 6.9 Hz). **HRMS** QTOF-MS: calcd. $C_{48}H_{55}NaO_{14}P$ for $[M+Na]^+$ 909.3227, found 909.3260.

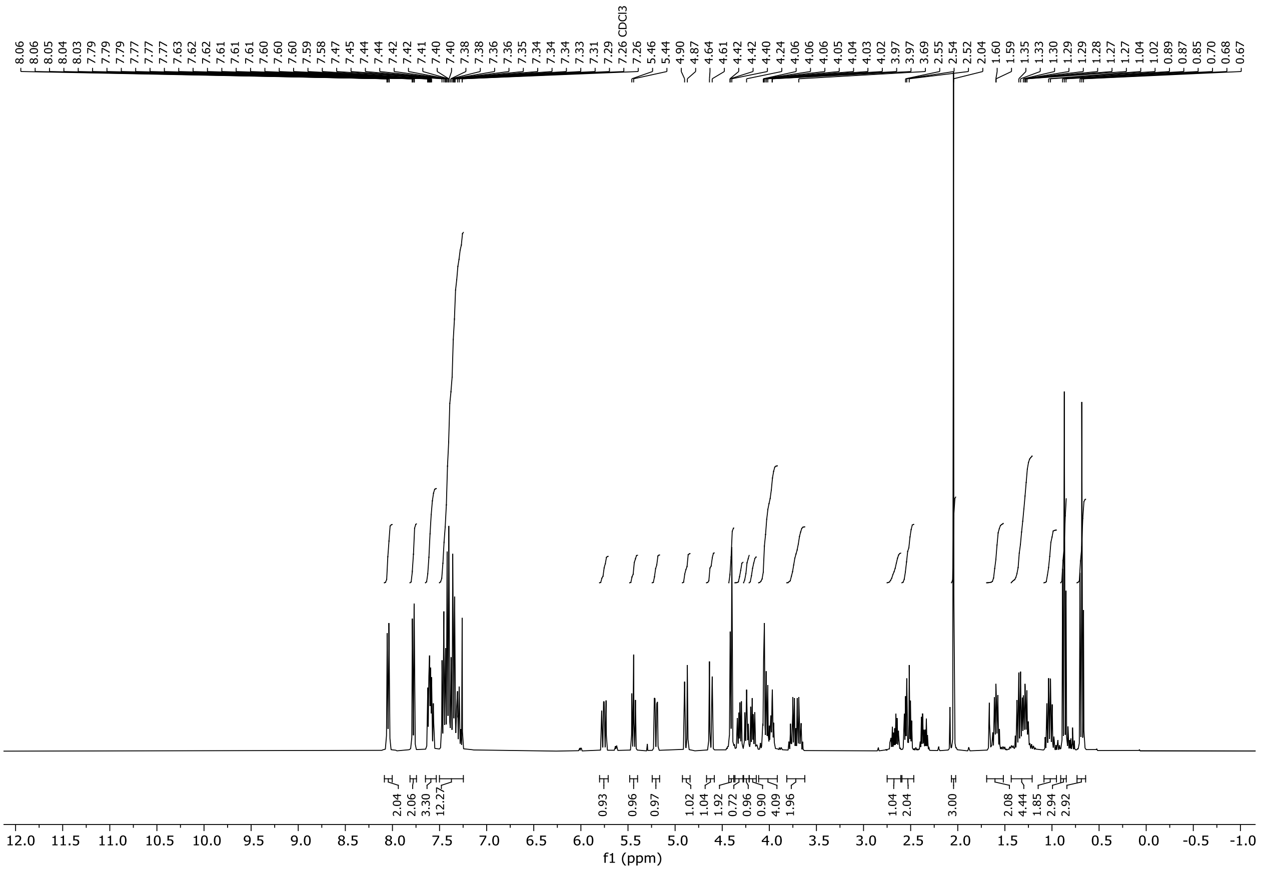

^{1}H NMR

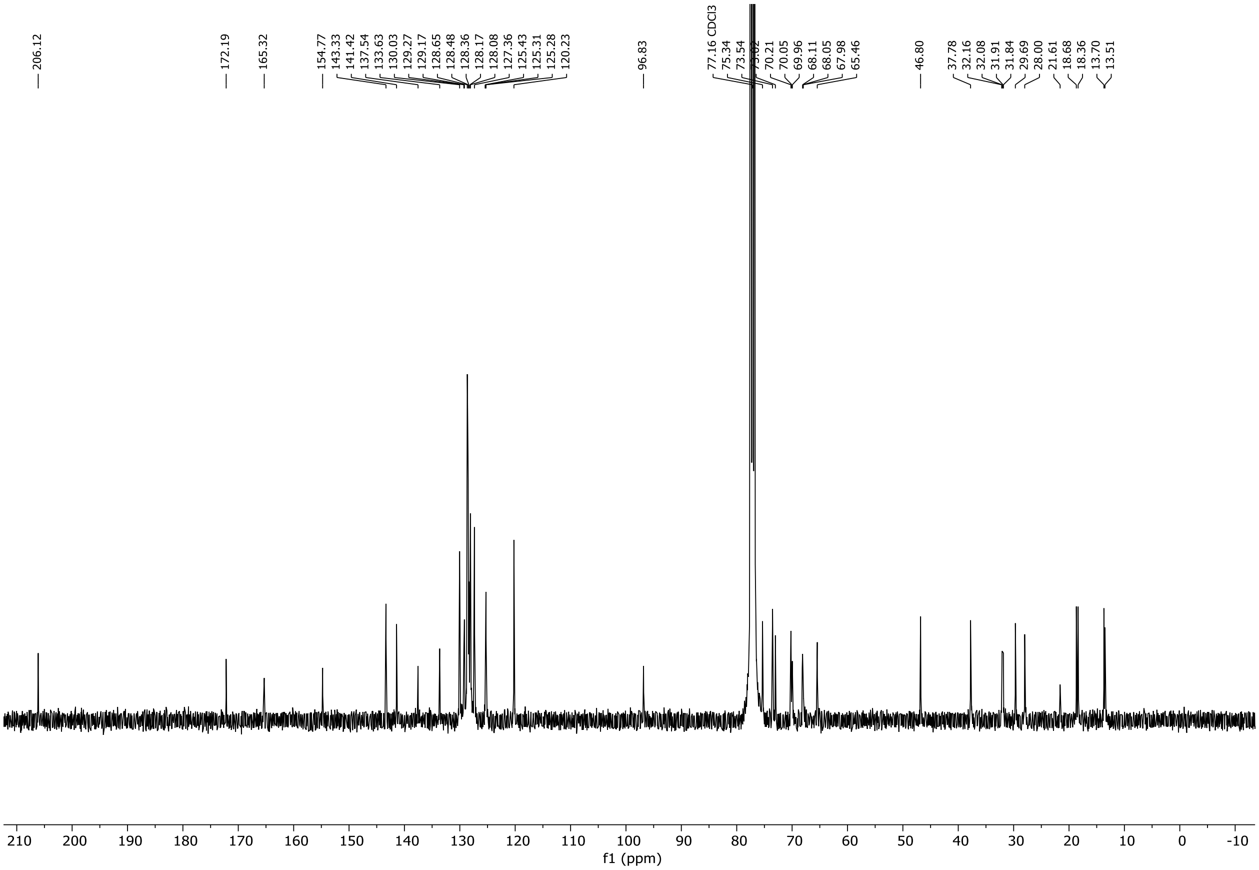
206.12
172.19
165.32
154.77
143.33
141.42
137.54
133.63
130.03
129.27
129.17
128.65
128.48
128.36
128.17
128.08
127.36
125.43
125.31
125.28
120.23
96.83
77.16 CDCl3
75.34
73.54
73.02
70.21
70.05
69.96
68.11
68.05
67.98
65.46
46.80
37.78
32.16
32.08
31.91
31.84
29.69
28.00
21.61
18.68
18.36
13.70
13.51
210
200
190
180
170
160
150
140
130
120
110
100
90
80
70
60
50
40
30
20
10
0
-10
f1 (ppm)

^{13}C NMR

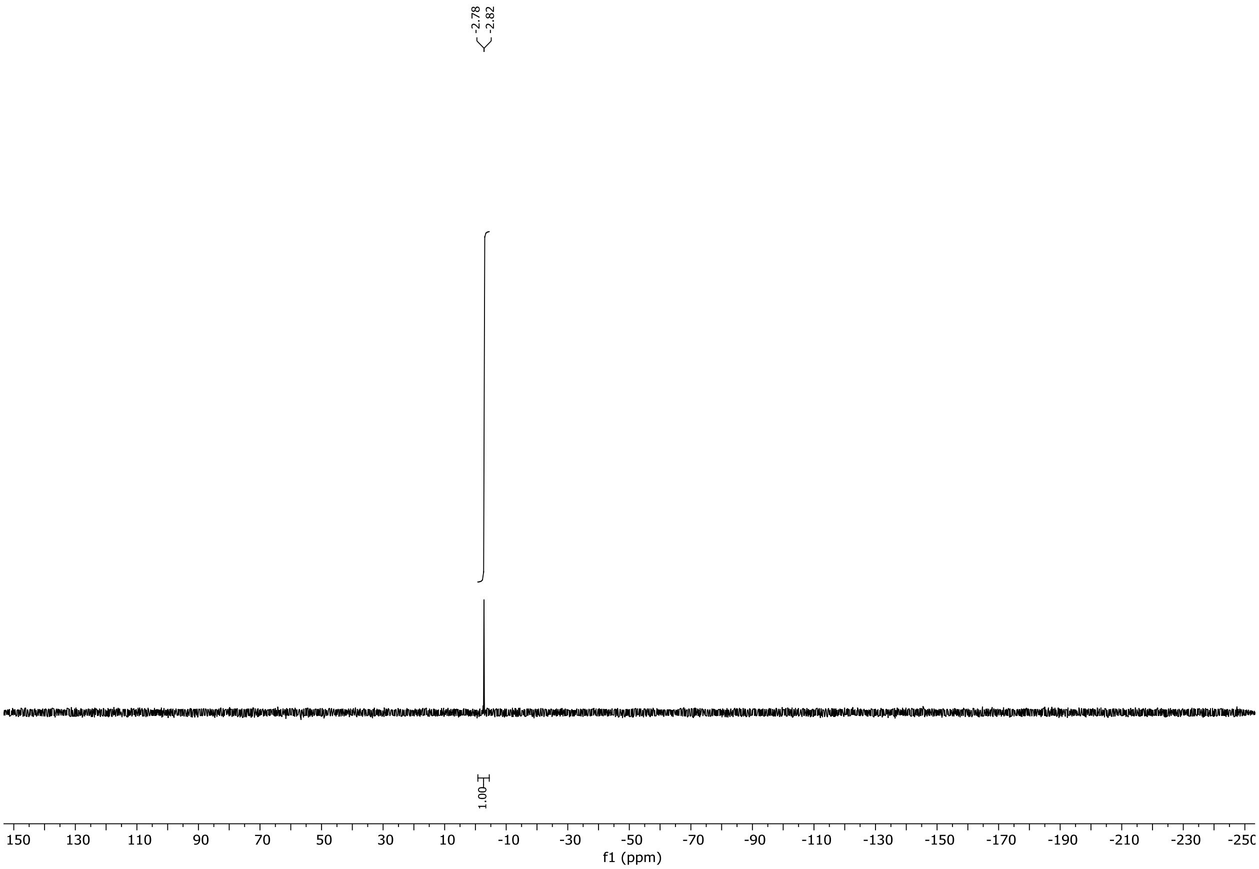
-2.78
-2.82
1.00
150
130
110
90
70
50
30
10
-10
-30
-50
-70
-90
-110
-130
-150
-170
-190
-210
-230
-250
f1 (ppm)

^{31}P NMR

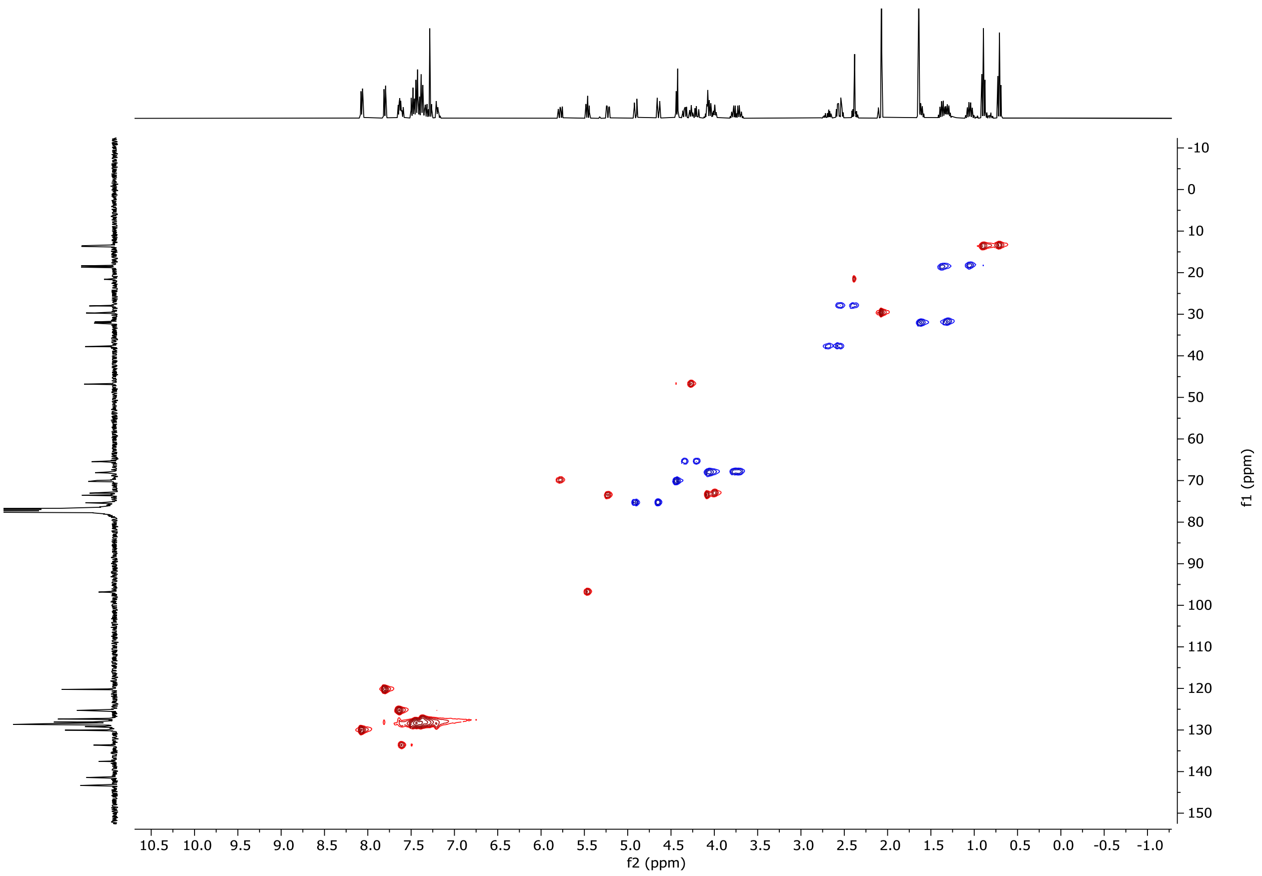

^{1}H-^{13}C HSQC NMR

Automated glycan assembly

Solvents were taken from an anhydrous solvent system (JC Meyer-solvent systems) to prepare activator, acid wash (TMSOTf), and capping solutions. The building blocks were co-evaporated once with toluene and dried under a high vacuum before use. All solutions were freshly prepared and kept under argon during the automation run. Final yields were calculated based on the resin loading. Resin loading was determined by performing a double glycosylation followed by DBU-promoted Fmoc-cleavage and determination of dibenzofulvene formation by measuring its UV absorbance.

Preparation of reagent solutions

Building block solution: Building block (0.09 mmol) was dissolved in CH_2Cl_2 (1 mL).

NIS/TfOH activator solution: Recrystallized NIS (1.56 g) was dissolved in 40 mL of a 2:1 v/v mixture of anhydrous CH_2Cl_2 and anhydrous dioxane. Then, triflic acid (55µL, 0.6 mmol) was added. The solution was kept on an ice bath for the duration of the automation run.

Fmoc deprotection solution: Either a solution of 20% piperidine in DMF (v/v) (module E1) or a solution of 20% triethylamine in DMF (v/v) was prepared (module E2).

Acid wash solution: TMSOTf (0.45 mL, 2.49 mmol) was dissolved in CH_2Cl_2 (40 mL).

Capping solution: A 50 mL solution of 10% acetic anhydride and 2% methanesulfonic acid in CH_2Cl_2 (v/v) was prepared.

Lev deprotection solution: A solution of hydrazine acetate (725 mg) in the mixture of pyridine (40 mL), acetic acid (10 mL) and water (2.5 mL) was prepared.

Modules for Automated Solid-Phase Synthesis

Resin preparation for synthesis

1. Conjugation-ready linker (Linker 1) with a loading of 0.4 mmol/g.

2. Traceless linker with a loading (Linker 2) of 0.4 mmol/g.

40mg of resin was placed in the reaction vessel and swollen in CH_2Cl_2 for 20 min at room temperature prior to synthesis. During this time, all reagent lines needed for the synthesis were washed and primed. Before the first glycosylation, the resin was washed with the DMF, THF, and CH_2Cl_2 (three times each with 2 mL for 25 s).

TMSOTf acidic wash solution (Module a): The resin was swollen in CH_2Cl_2 (2 mL) and the temperature of the reaction vessel was adjusted to -20 °C. Upon reaching the low temperature, TMSOTf solution (1 mL, 0.06 mmol) was added dropwise to the reaction vessel. After bubbling for 3 min, the acidic solution was drained and the resin was washed with CH_2Cl_2 (2 mL) for 25 s.

Action	Cycles	Solution	Amount	T (°C)	Incubation time
Cooling	-	-	-	-20	-
Deliver	1	CH_2Cl_2	2 mL	-20	-
Deliver	1	TMSOTf solution	1 mL	-20	3 min
Wash	1	CH_2Cl_2	1 mL	-20	25 s

Thioglycoside glycosylation (Module b1): The building block solution (0.1 mmol of BB in 1 mL of CH_2Cl_2 per glycosylation) was delivered to the reaction vessel. After the set temperature was reached, the reaction was started by dropwise addition of the activator solution (1.0 mL, excess). After completion of the reaction, the solution is drained and the resin was washed with CH_2Cl_2, CH_2Cl_2/dioxane (1:2, v/v, 2 mL for 20 s), and CH_2Cl_2 (twice, each with 2 mL for 25 s). The temperature of the reaction vessel is increased to 25°C for the next module.

Action	Cycles	Solution	Amount	T (°C)	Incubation time
Cooling	-	-	-	-20	-
Deliver	1	BB solution	1 mL	-20	-
Deliver	1	activator solution	1 mL	-20	-
Reaction time	1			-20	15 min
				to 0	35 min
Wash	1	CH_2Cl_2	2 mL	0	25 sec
Wash	1	CH_2Cl_2:Dioxane	2 mL	0	20 sec
Heating	-	-	-	25	-
Wash	1	CH_2Cl_2	2 mL	>0	25 sec

Phosphate glycosylation (Module b2): The building block solution (0.1 mmol of BB in 1 mL of CH_2Cl_2 per glycosylation) was delivered to the reaction vessel. After the set temperature was reached, the reaction was started by dropwise addition of the activator solution (1.0 mL, excess). After completion of the reaction, the solution is drained and the resin was washed with CH_2Cl_2, CH_2Cl_2 /dioxane (1:2, v/v, 2 mL for 20 s), and CH_2Cl_2 (twice, each with 2 mL for 25 s). The temperature of the reaction vessel is increased to 25°C for the next module.

Action	Cycles	Solution	Amount	T (°C)	Incubation time
Cooling	-	-	-	-35	-
Deliver	1	BB solution	1 mL	-35	-
Deliver	1	activator solution	1 mL	-35	-
Reaction time	1			-35<br>to -20	5 min<br>30 min
Wash	1	CH_2Cl_2	2 mL	0	25 sec
Wash	1	CH_2Cl_2:Dioxane	2 mL	0	20 sec
Heating	-	-	-	25	-
Wash	1	CH_2Cl_2	2 mL	>0	25 sec

Capping (Module c): The resin was washed twice with DMF (2 mL, 25 s) and the temperature of the reaction vessel was adjusted to 25 °C. Pyridine solution (2 mL, 10% in DMF) was delivered into the reaction vessel. After 1 min, the reaction solution was drained and the resin was washed with CH_2Cl_2 (three times with 3 mL for 25 s). Capping solution (4 mL) was delivered into the reaction vessel. After 20 min, the reaction solution was drained and the resin was washed with CH_2Cl_2 (three times with 3 mL for 25 s).

Action	Cycles	Solution	Amount	T (°C)	Incubation time
Heating	-	-	-	25	-
Wash	2	DMF	2 mL	25	25 s
Deliver	1	10% Py./DMF	2 mL	25	1 min
Wash	3	CH_2Cl_2	2 mL	25	25 s
Deliver	1	Capping solution	4 mL	25	20 min
Wash	3	CH_2Cl_2	2 mL	-20	25 s

Fmoc deprotection with piperidine (Module d1): The resin was washed with DMF (three times with 2 mL for 25 s) and the temperature of the reaction vessel was adjusted to 25 °C. 2 mL of Fmoc deprotection solution was delivered to the reaction vessel and kept under Ar bubbling. After 5 min, the reaction solution was drained and the resin was washed with DMF (three times with 2 mL for 25 s) and CH_2Cl_2 (five times each with 2 mL for 25 s). The temperature of the reaction vessel was decreased to -20 °C for the next module.

Action	Cycles	Solution	Amount	T (°C)	Incubation time
Wash	3	DMF	2 mL	25	25 s
Deliver	1	Fmoc depr.	2 mL	25	5 min
Wash	3	DMF	2 mL	25	25 s
Wash	5	CH_2Cl_2	2 mL	25	25 s
Cooling	1	-	-	-20	-

Fmoc deprotection with NEt_3 (Module d2): The resin was washed with DMF (three times with 2 mL for 25 s) and the temperature of the reaction vessel was adjusted to 25 °C. Fmoc deprotection solution was delivered to the reaction vessel and kept under Ar bubbling (three times with 2 mL). After 5 min, the reaction solution was drained and the resin was washed with DMF (three times with 2 mL for 25 s) and CH_2Cl_2 (five times each with 2 mL for 25 s). The temperature of the reaction vessel was decreased to -20 °C for the next module.

**Action**	**Cycles**	**Solution**	**Amount**	**T (°C)**	**Incubation time**
Wash	3	DMF	2 mL	25	25 s
Deliver	3	Fmoc depr.	2 mL	25	5 min
Wash	3	DMF	2 mL	25	25 s
Wash	5	CH_2Cl_2	2 mL	25	25 s
Cooling	1	-	-	-20	-

Levulinoyl ester deprotection (Module e): The resin was washed with CH_2Cl_2 (three times with 2 mL for 25 s) and the temperature of the reaction vessel was adjusted to 35 °C. 2 mL of Levulinoyl ester deprotection solution was delivered to the reaction vessel and kept under Ar bubbling. After 30 min, the reaction solution was drained and the resin was washed with DMF, THF and CH_2Cl_2 (six times each with 2 mL for 25 s).

Action	Cycles	Solution	Amount	T (°C)	Incubation time
Heating	-	-	-	35	-
Wash	3	CH_2Cl_2	2 mL	35	25 s
Deliver	1	Lev solution	2 mL	35	30 min
Wash	6	CH_2Cl_2	2 mL	35	25 s
Deliver	1	Lev solution	2 mL	35	30 min
Wash	9	CH_2Cl_2	2 mL	35	25 s
Deliver	1	Lev solution	2 mL	35	30 min
Wash	3	CH_2Cl_2	2 mL	-20	25 s
Wash	6	DMF	2mL	<25	25 s
Wash	6	THF	2mL	<25	25 s
Wash	6	CH_2Cl_2	2mL	<25	25 s

Solid-phase synthesis

Nap cleavage (Module f): The resin was suspended in a silanized glass microwave vial in CH_2Cl_2:H_2O (5mL, 90:10, v/v). Then, DDQ (2 eq. per nap group) was added and the reaction was stirred gently for 120 mins at room temperature. The resin was then washed successively with CH_2Cl_2 /H_2O, CH_2Cl_2, DMF, THF, methanol, and CH_2Cl_2.

Methanolysis (Module g): The resin was suspended in anhydrous THF (4.8 mL). Then, 0.2 mL of a solution of NaOMe in MeOH (0.5 M) was added and the resin was shaken at room temperature for 16 h. The resin was then washed successively with THF, CH_2Cl_2, methanol, and CH_2Cl_2.

Sulfation (Module h1): The resin was suspended in a silanized glass microwave vial in 1 mL DMF. Then, a solution of Py·SO_3 (20 eq. per OH) in DMF:Py (4 mL, 80:20 v/v) was added. The microwave vial was sealed and the temperature was raised to 50 °C with gentle stirring for 16 h. Thereafter, the reaction was cooled, and the resin was washed successively with DMF, CH_2Cl_2, methanol, and CH_2Cl_2.

Sulfation (Module h2): The resin was suspended in a silanized glass microwave vial in 1 mL DMF. Then, a solution of NEt_3·SO_3 (20 eq. per OH) in DMF:NEt_3 (4 mL, 80:20 v/v) was added. The microwave vial was sealed and the temperature was raised to 50 °C with gentle stirring for 16 h. Thereafter, the reaction was cooled, and the resin was washed successively with DMF, CH_2Cl_2, methanol, and CH_2Cl_2.

Photocleavage from the solid support (Module i): Glycans were cleaved from the solid support using a batch-flow photoreactor. The resin-bound glycan (~40 mg) was suspended in DMF (4 mL) under the irradiation of an LED lamp (370nm), with stirring for 24 hours. The solution was separated from the resin using a fritted syringe and concentrated under vacuum.

Solution-phase synthesis

Hydrogenolysis (Module j1), non-sulfated fucoidans: The crude compound was dissolved in 4 mL of THF: *t*BuOH: H_2O (60:10:30). 5% Pd-C (200 mg) was added and the reaction was stirred under H_2 atmosphere for 12 h. The reaction was filtered through a pad of celite and washed with *t*BuOH and H_2O. The filtrates were concentrated *in vacuum*, and dissolved in 3.0 mL of water for RP-HPLC purification.

Hydrogenolysis (Module j2), sulfated fucoidans: The crude compound was dissolved in 4 mL of THF: *t*BuOH: H_2O (50:20:30). 5% Pd-C (200 mg) was added and the reaction was stirred under H_2 atmosphere for 12 h. The reaction was filtered through a pad of celite and washed with *t*BuOH and H_2O. The filtrates were concentrated *in vacuum*, and dissolved in 3.0 mL of water for RP-HPLC purification

HPLC analysis and purification

Analytical traces of crude and pure compounds were collected using an analytic RP-HPLC Agilent 1200 Series (**Methods 1**, **3**, **5**, **6**, **7**, **8**). Purification of the crudes was conducted using a preparative RP-HPLC Agilent 1200 Series (**Methods 2**, **4**, **9**).

Method 1, analytic RP-HPLC (non-sulfated oligosaccharide): (Hypercarb column, 150 x 4.6 mm, 3 µm) flow rate of 0.7 mL/min with ACN/H_2O (0.1% formic acid) as eluents [isocratic 100 % H_2O (0.1% formic acid) (5 min), linear gradient to 100% ACN (30 min)].

Method 2, prep RP-HPLC (non-sulfated oligosaccharide): (Hypercarb column, 150 x 10 mm, 5 µm), flow rate of 3.5 mL /min with H_2O (0.1% formic acid) as eluents [isocratic 100 % H_2O (0.1% formic acid) (5 min), linear gradient to 100% ACN (30 min)].

Method 3 analytic RP-HPLC (sulfated oligosaccharide): (Hypercarb column, 150 x 4.6 mm, 3 µm) flow rate of 0.7 mL/min with ACN/H_2O (0.1mM $(NH_4)_2CO_3$) as eluents [isocratic 100 % H_2O (0.1mM $(NH_4)_2CO_3$) (5 min), linear gradient to 100% ACN (30 min)].

Method 4 prep RP-HPLC (sulfated oligosaccharide): (Hypercarb column, 150 x 10 mm, 5 µm), flow rate of 3.5 mL /min with ACN/H_2O (0.1mM $(NH_4)_2CO_3$) as eluents [isocratic 100 % H_2O (0.1mM $(NH_4)_2CO_3$) (5 min), linear gradient to 100% ACN (30 min)].

Method 5 analytic RP-HPLC (semi-protected oligosaccharide): (Phenomenex, luna C5 column, 250 x 4.6 mm, 5 µm), flow rate of 1.0 mL /min with ACN/H_2O (0.1% formic acid) as eluents [isocratic 5% ACN (5 min), linear gradient to 100% ACN (30 min)].

Method 6 analytic RP-HPLC (semi-protected sulfated oligosaccharide): (Phenomenex, luna C5 column, 250 x 4.6 mm, 5 µm), flow rate of 1.0 mL /min with ACN/H_2O (0.1mM $(NH_4)_2CO_3$) as eluents [isocratic 5% ACN (5 min), linear gradient to 100% ACN (30 min)].

Method 7 SEC-HPLC (sulfated or non-sulfated oligosaccharide): (TSKgel G 3000 PWXL column, 7.8 mm x 30 cm) flow rate of 0.4 mL/min with water as an eluent.

Method 8 SEC-HPLC analytic (sulfated or non-sulfated oligosaccharide): (CatPhil-P-100 column, 300 mm x 8 mm) flow rate of 0.3 mL/min with water as an eluent.

Method 9 SEC-HPLC prep (sulfated or non-sulfated oligosaccharide): (CatPhil-P-100 column, 300 mm x 20 mm) flow rate of 1.0 mL/min with water as an eluent.

Method 10 HILIC analytical (sulfated oligosaccharides): (TSKgel Amide-80 column, 4.6 mm x 25 mm) flow rate of 1.0 mL/min with ACN/H_2O (0.1mM $(NH_4)_2CO_3$) as eluents [isocratic 90% ACN (5 min), linear gradient to 60% ACN (50 min), isocratic at 60% ACN (60 min), gradient to 90% ACN (70 min).

Method 11 HILIC prep (sulfated oligosaccharides): TSKgel Amide-80 column, 250 mm x 10 mm) flow rate of 4.0 mL/min with ACN/H_2O (0.1mM $(NH_4)_2CO_3$) as eluents

[isocratic 90% ACN (5 min), linear gradient to 60% ACN (50 min), isocratic at 60% ACN (60 min), gradient to 90% ACN (70 min).

Method 12 HILIC analytical (sulfated oligosaccharides): TSKgel Amide-80 column, 4.6 mm x 25 mm) flow rate of 1.0 mL/min with ACN/H_2O (0.1mM $(NH_4)_2CO_3$, [80% ACN to 60% ACN (20 min), gradient to 50% ACN (30 min), gradient to 20% ACN (35 min), isocratic at 20% ACN (40 min), and gradient to 80% ACN (50 min).

Following purification, all products were lyophilized on a Christ Alpha 2-4 LD plus freeze dryer before characterization.

LC-MS analysis of oligosaccharides

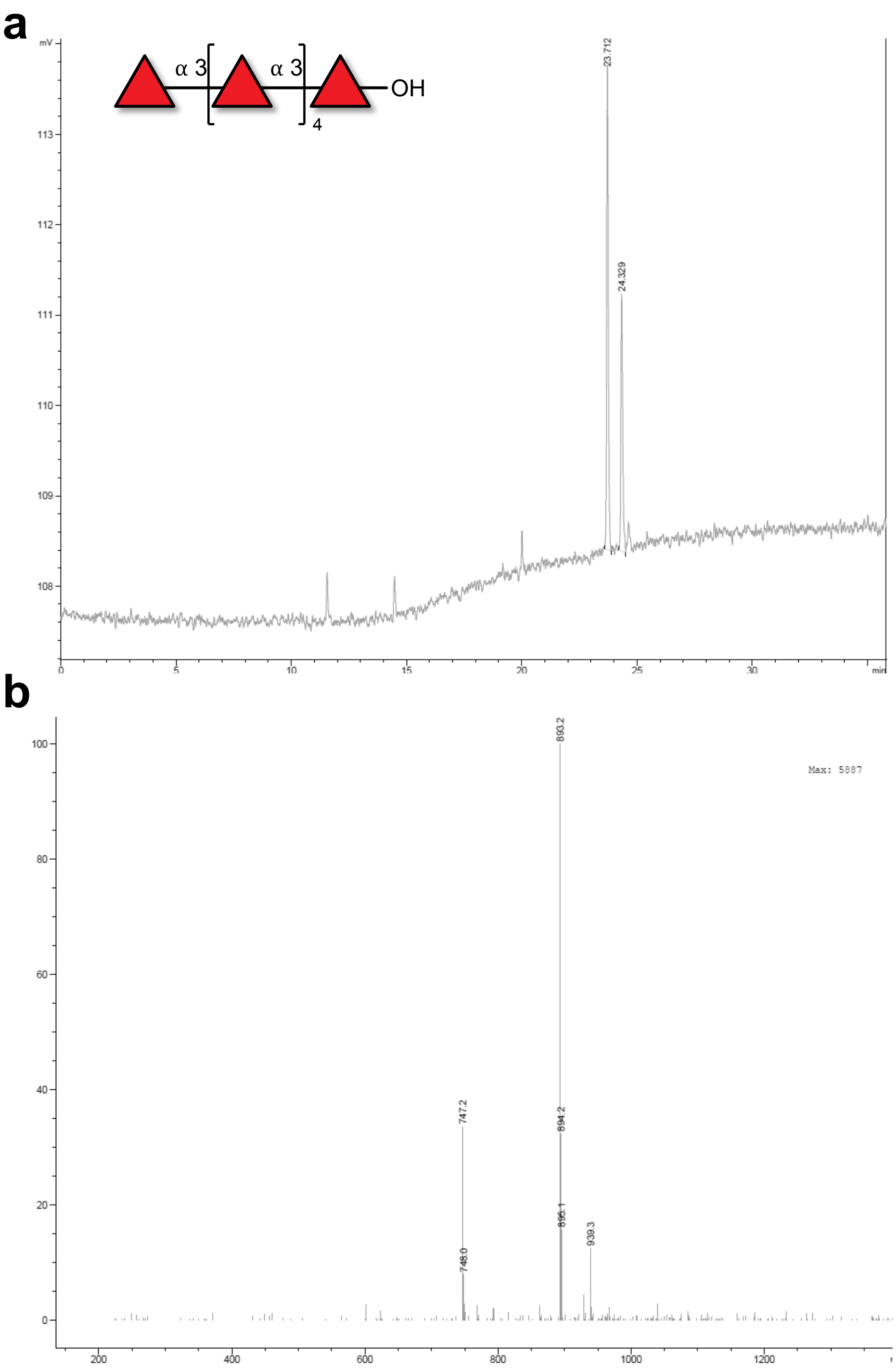

Trace of compound **6**. **a** ELSD trace. Hybercarb column, method 1. **b** MSD trace. $[C_{36}H_{62}O_{25}]^{-1}$ Expected mass, 893.3, observed 893.2

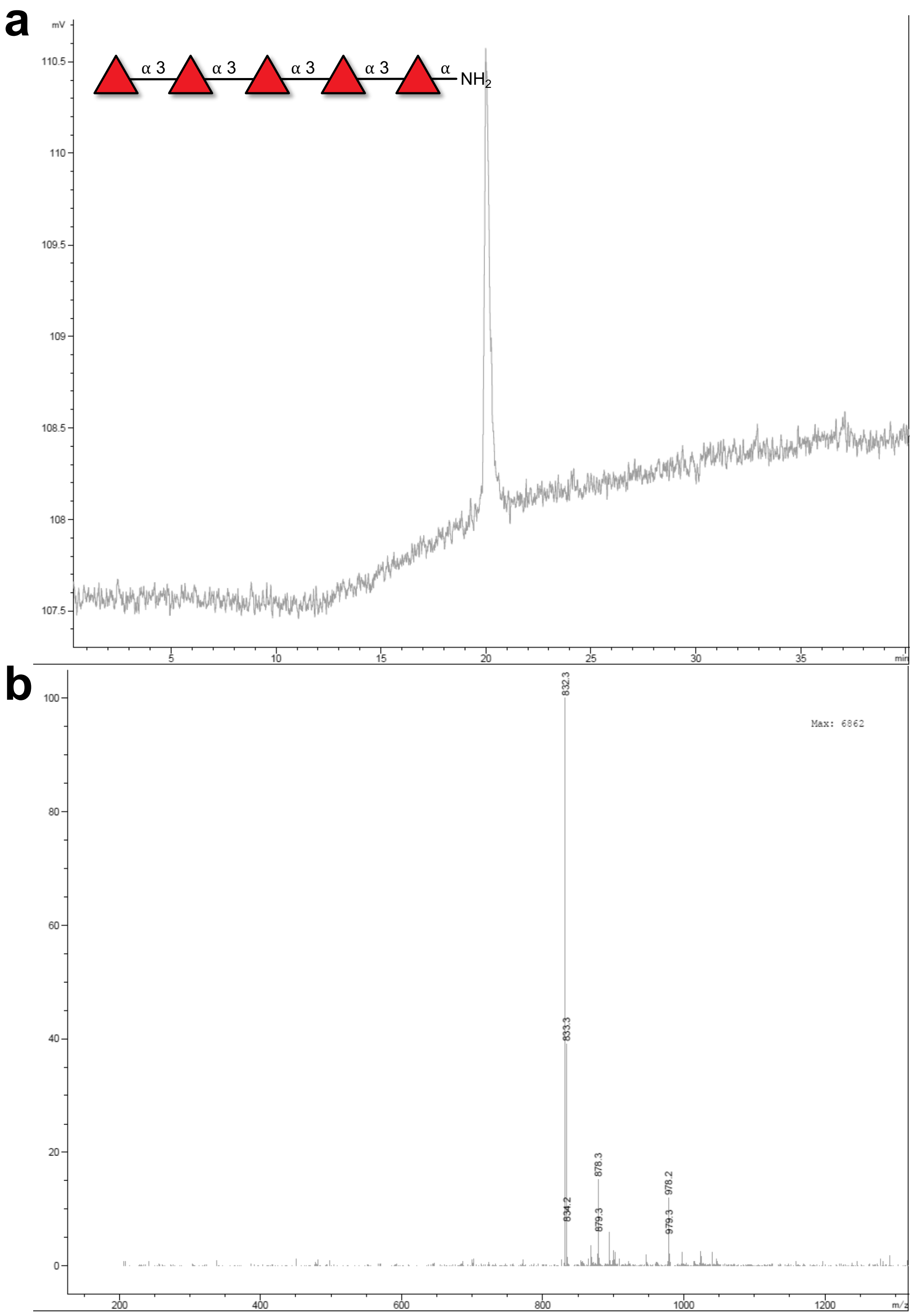

Trace of compound **7**. **a** ELSD trace. Hybercarb column, method 1. **b** MSD trace. $[C_{35}H_{62}NO_{21}]^{-1}$ Expected mass, 832.3, observed 832.3

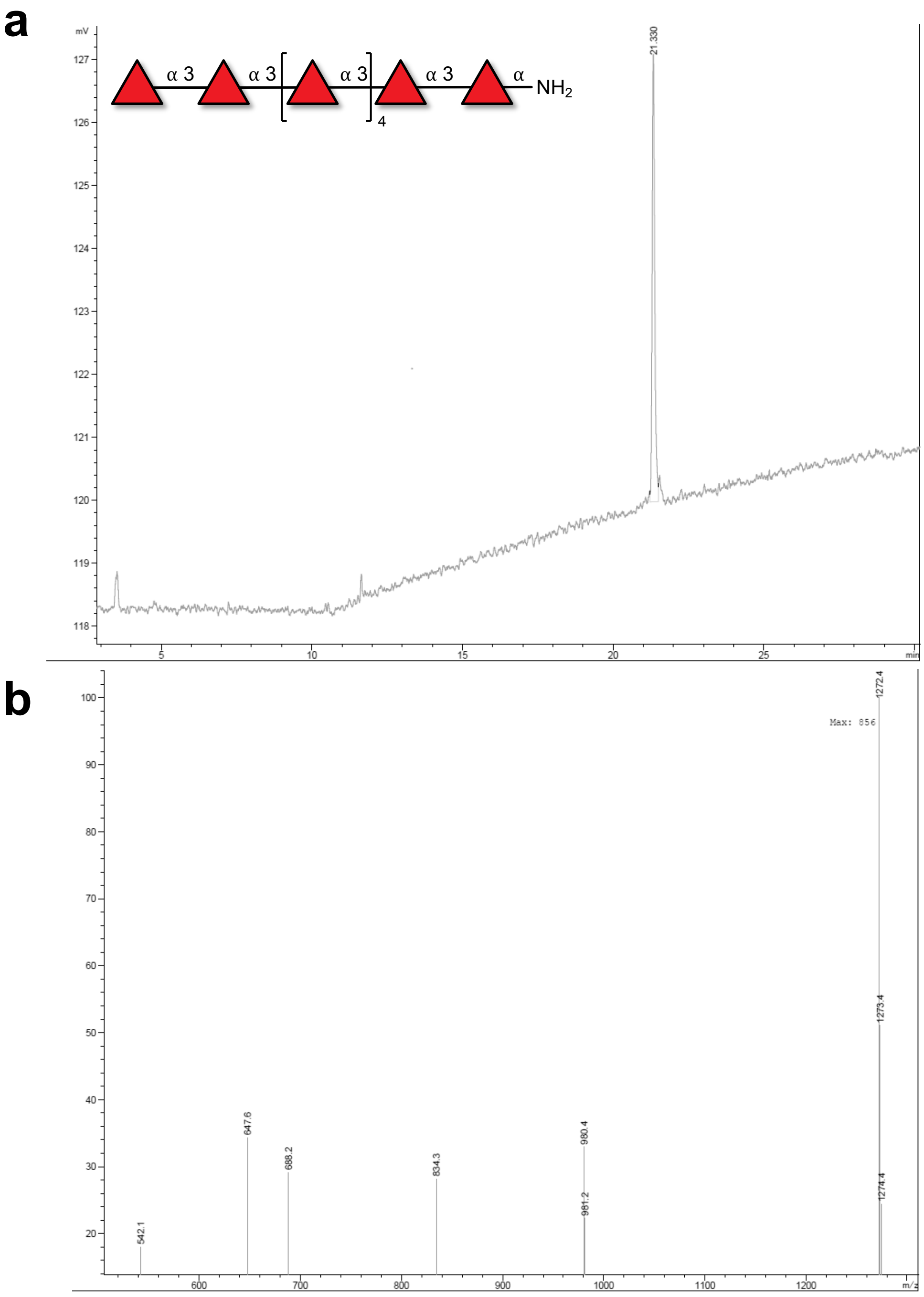

Trace of compound **8**. **a** ELSD trace. Hybercarb column, method 1. **b** MSD trace. $[C_{53}H_{94}NO_{33}]^{+1}$ Expected mass, 1272.5, observed 1272.4.

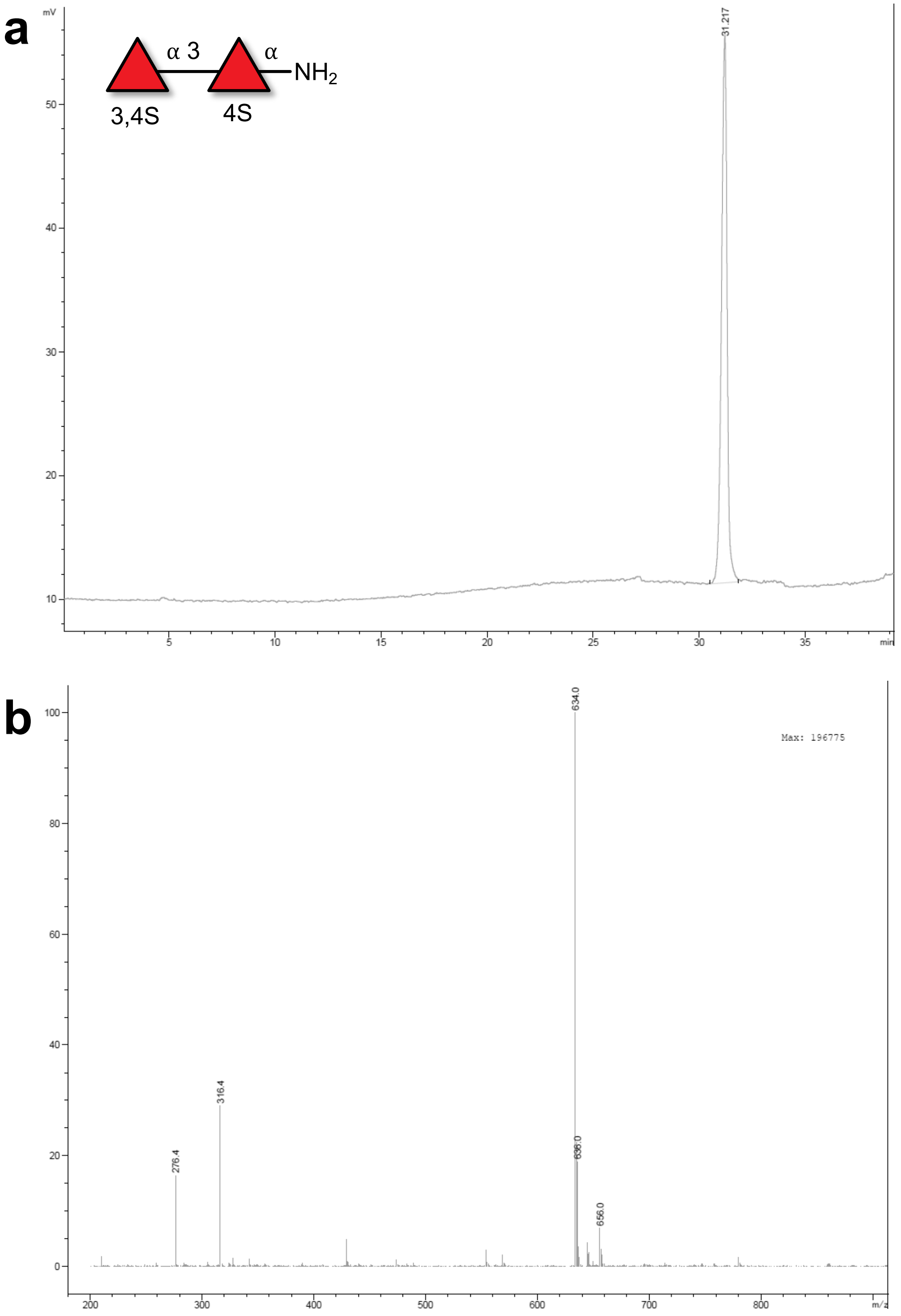

Trace of compound **9**. **a** ELSD trace. HILIC column, method 10. **b** MSD trace. $[C_{17}H_{32}NO_{18}S_3]^{-1}$ Expected mass, 634.0, observed 634.0.

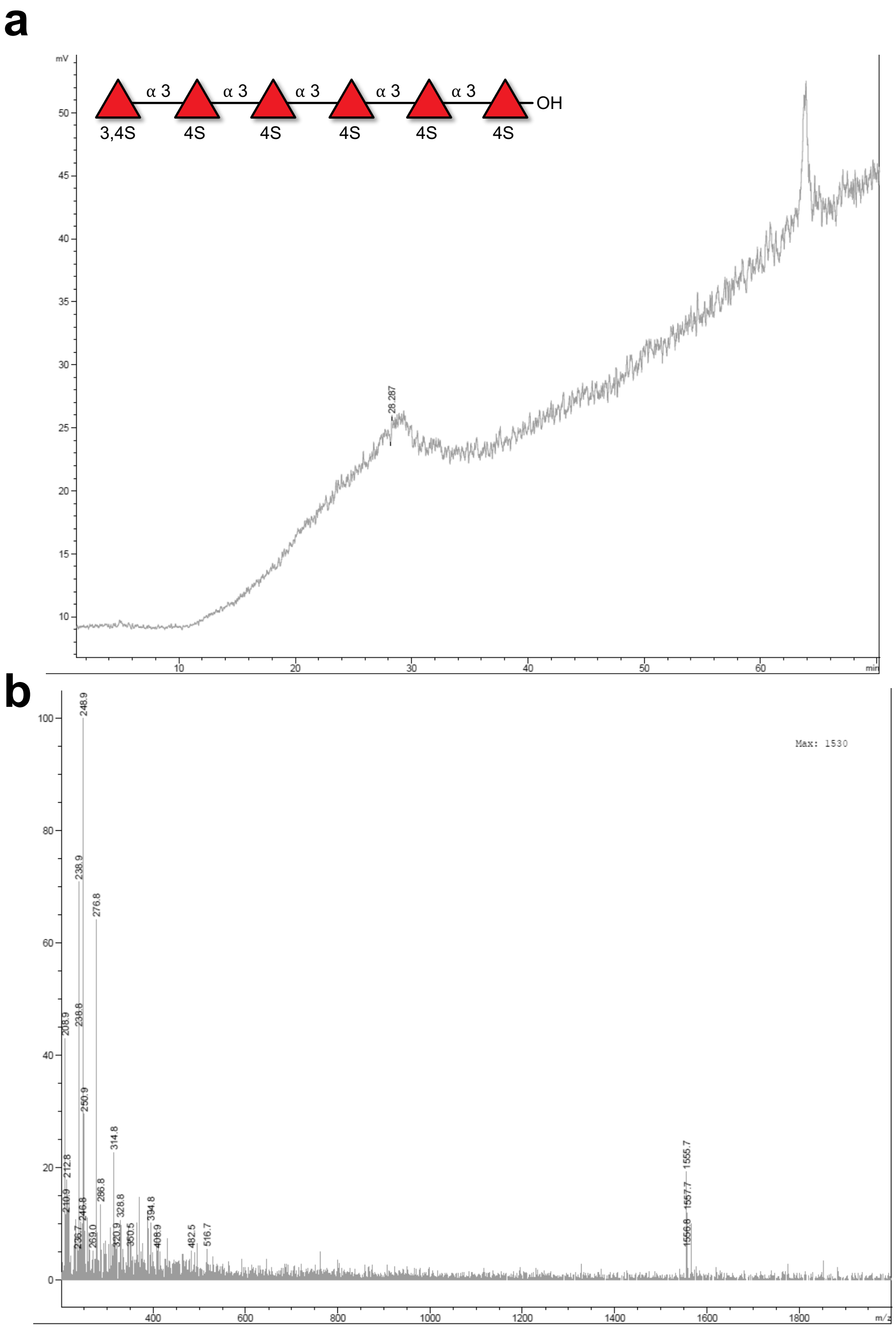

Trace of compound **10**. **a** ELSD trace. HILIC column, method 10. **b** MSD trace. $[C_{36}H_{79}N_6O_{46}S_7]^{-1}$ Expected mass, 1555.2, observed 1555.7.

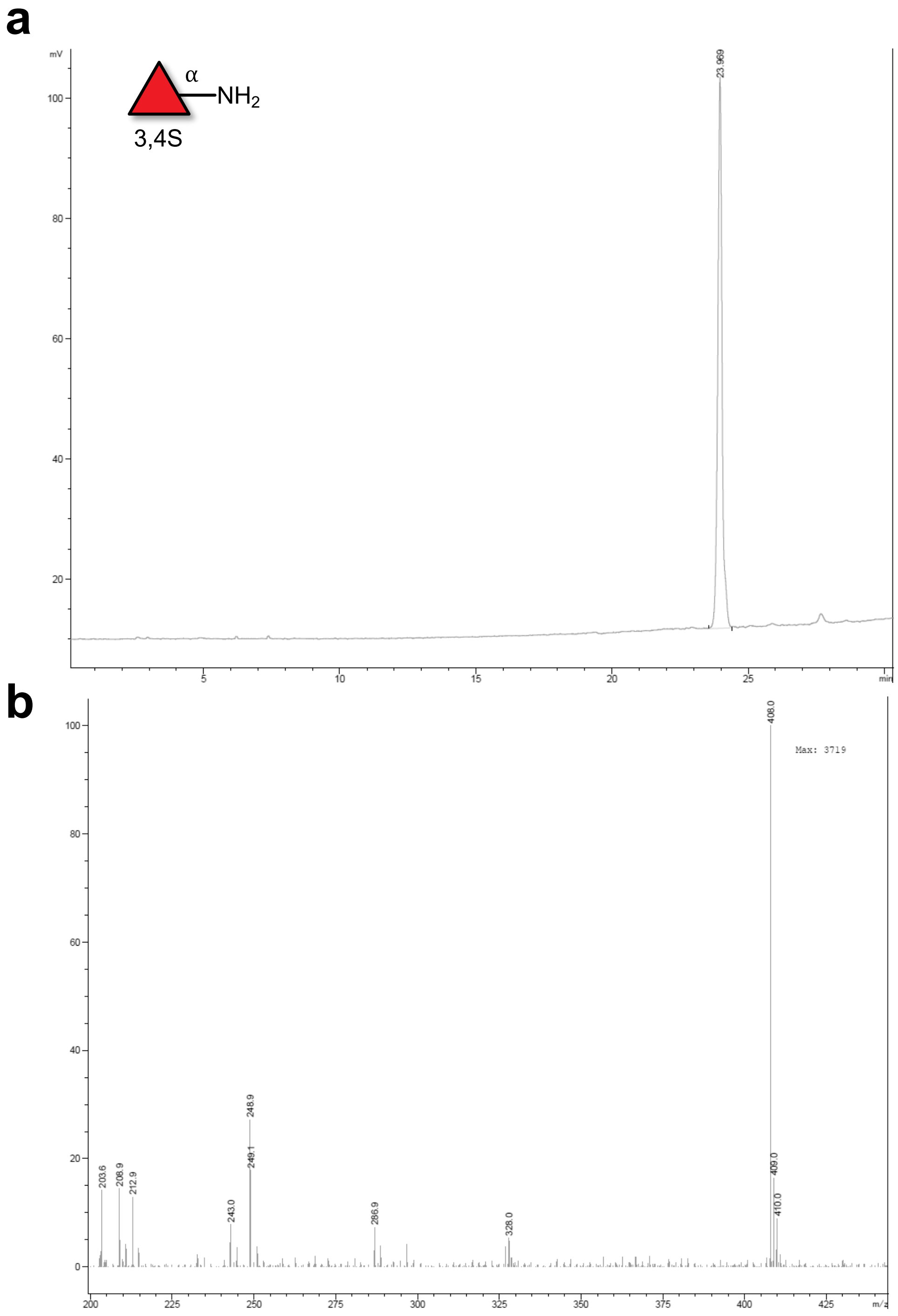

Trace of compound **11**. **a** ELSD trace. HILIC column, method 10. **b** MSD trace. $[C_{11}H_{22}NO_{11}S_2]^{-1}$Expected mass, 408.0, observed 408.0.

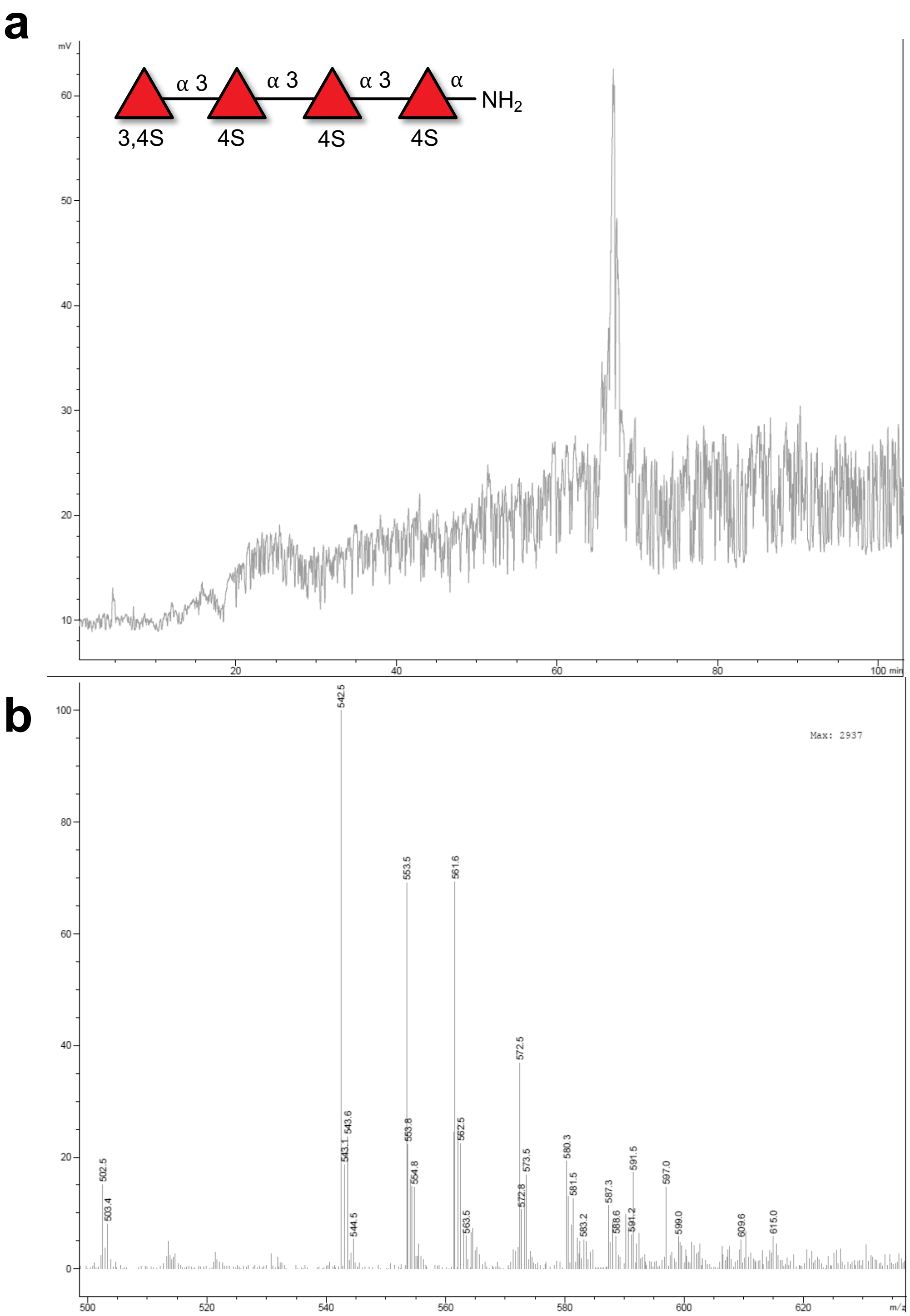

Trace of compound **13**. **a** ELSD trace. HILIC column, method 10. **b** MSD trace. MSD trace. $[C_{29}H_{51}NO_{32}S_5]^{-2}$ Expected mass, 542.5, observed 542.5. Comment: higher m/z are sodium and/or ammonium (NH4) adducts of sulfate esters. e.g. $[C_{29}H_{50}NNaO_{32}S_5]^{-2}$ Expected mass, 553.5 m/z, observed 553.5.

a

b

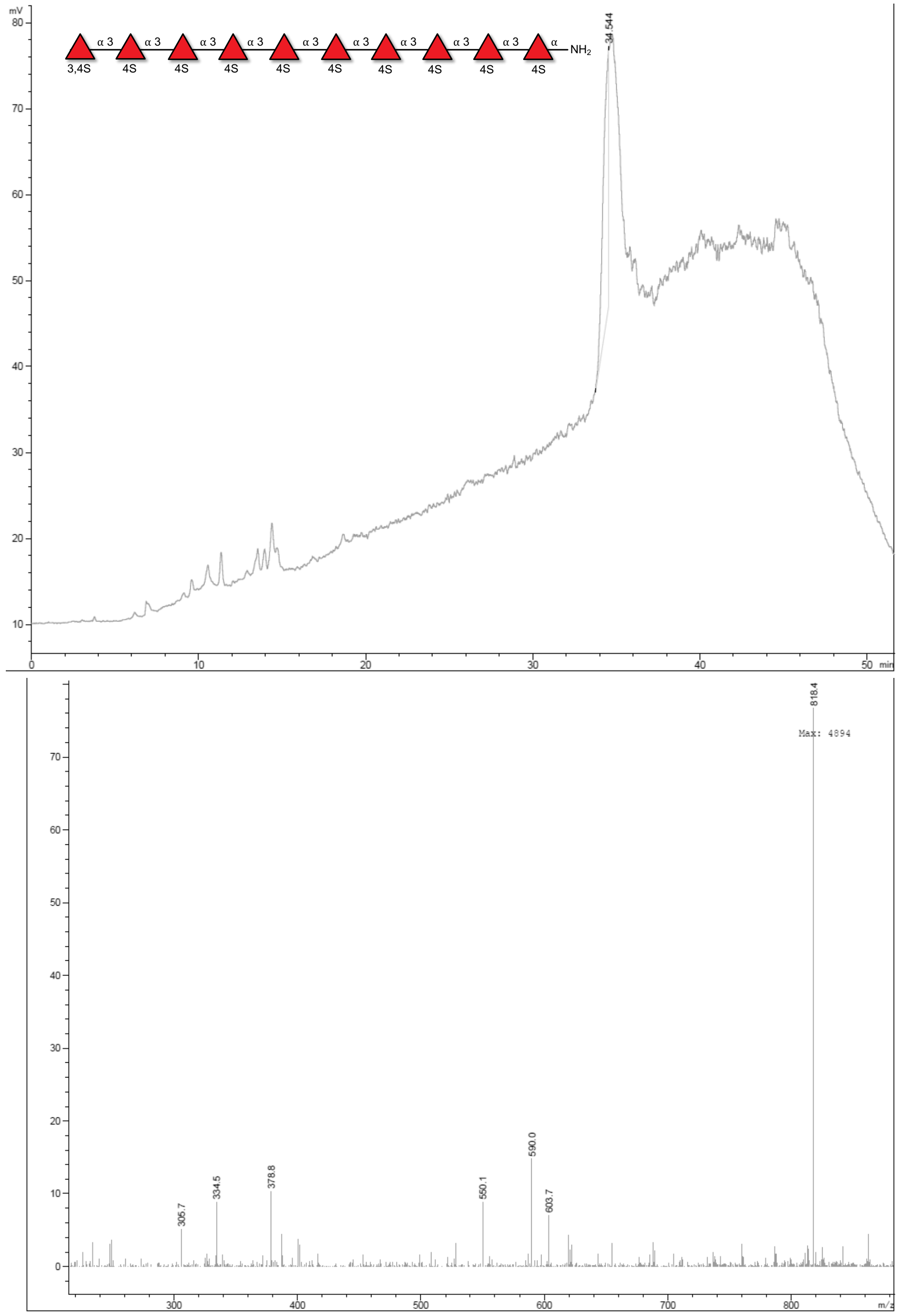

Trace of compound **14**. **a** ELSD trace. HILIC column, method 12. **b** MSD trace. $[C_{65}H_{113}N_2O_{74}S_{11}]^{-3}$ Expected mass, 819.0, observed 818.4.

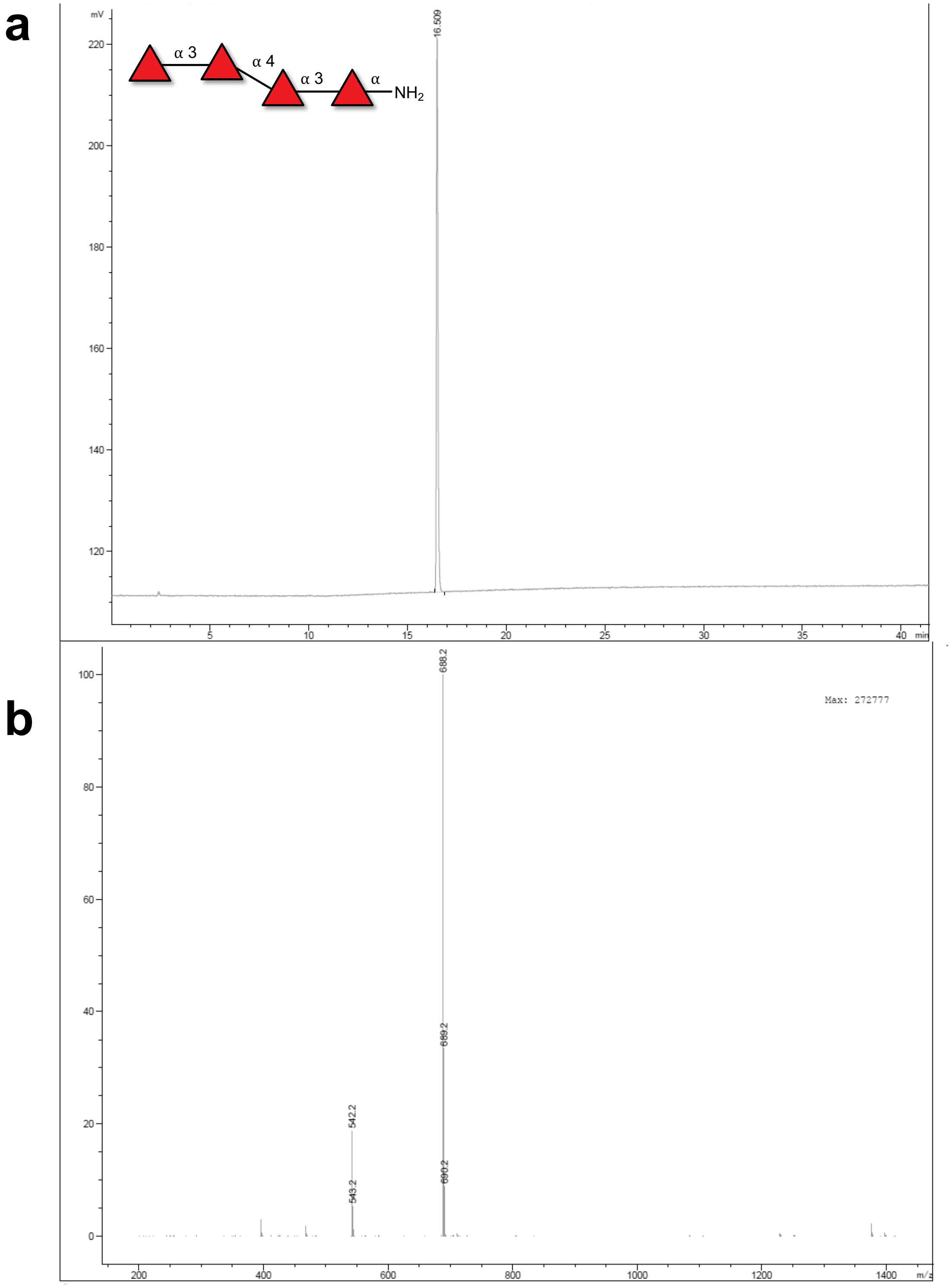

Trace of compound **15**. **a** ELSD trace. Hypercarb column, method 1. **b** MSD trace. $[C_{29}H_{54}NO_{17}]^{+1}$ Expected mass, 688.3, observed 688.2.

a

b

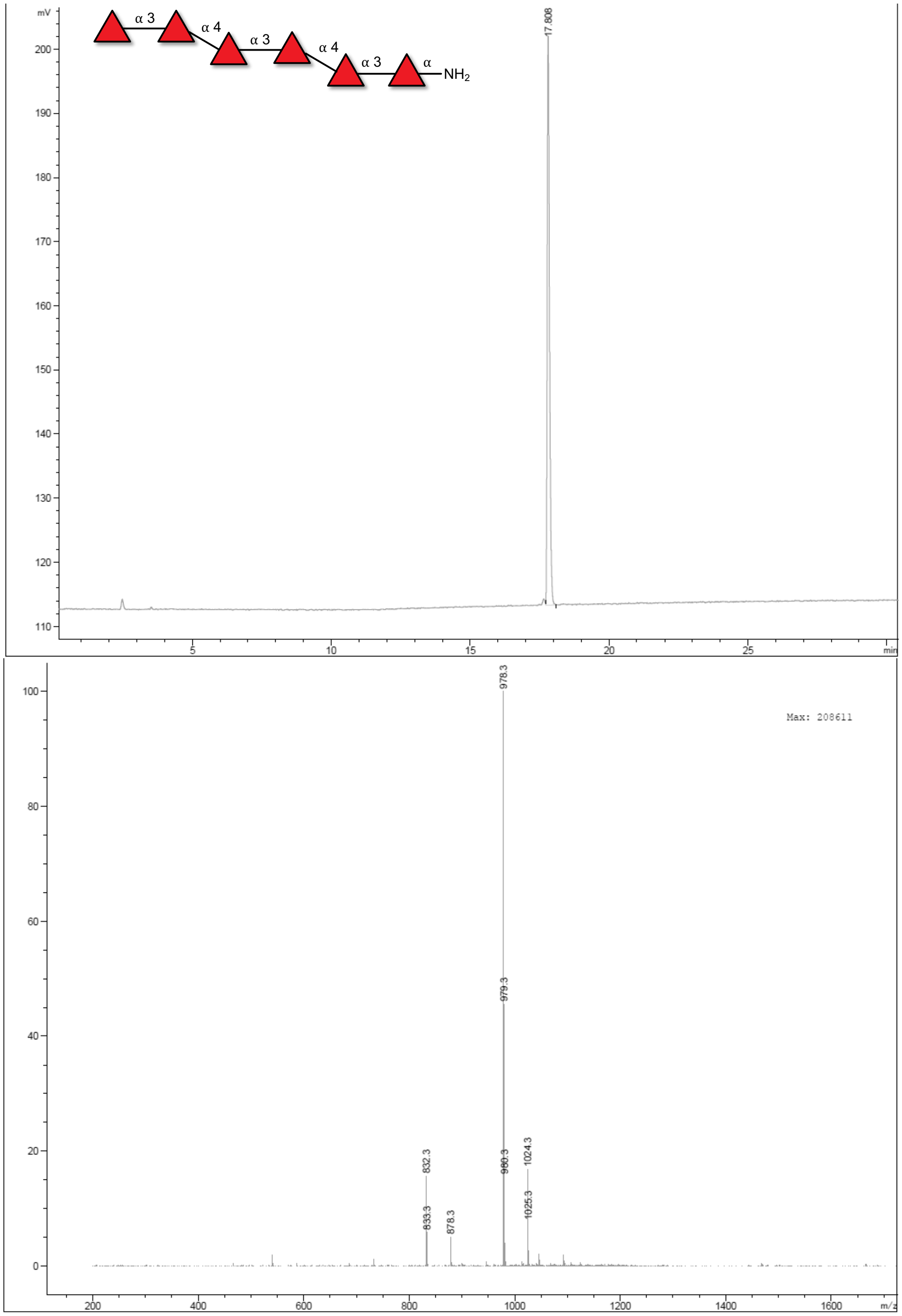

Trace of compound **16**. **a** ELSD trace. Hypercarb column, method 1. **b** MSD trace. $[C_{41}H_{72}NO_{25}]^{-1}$Expected mass, 978.4, observed 978.3.

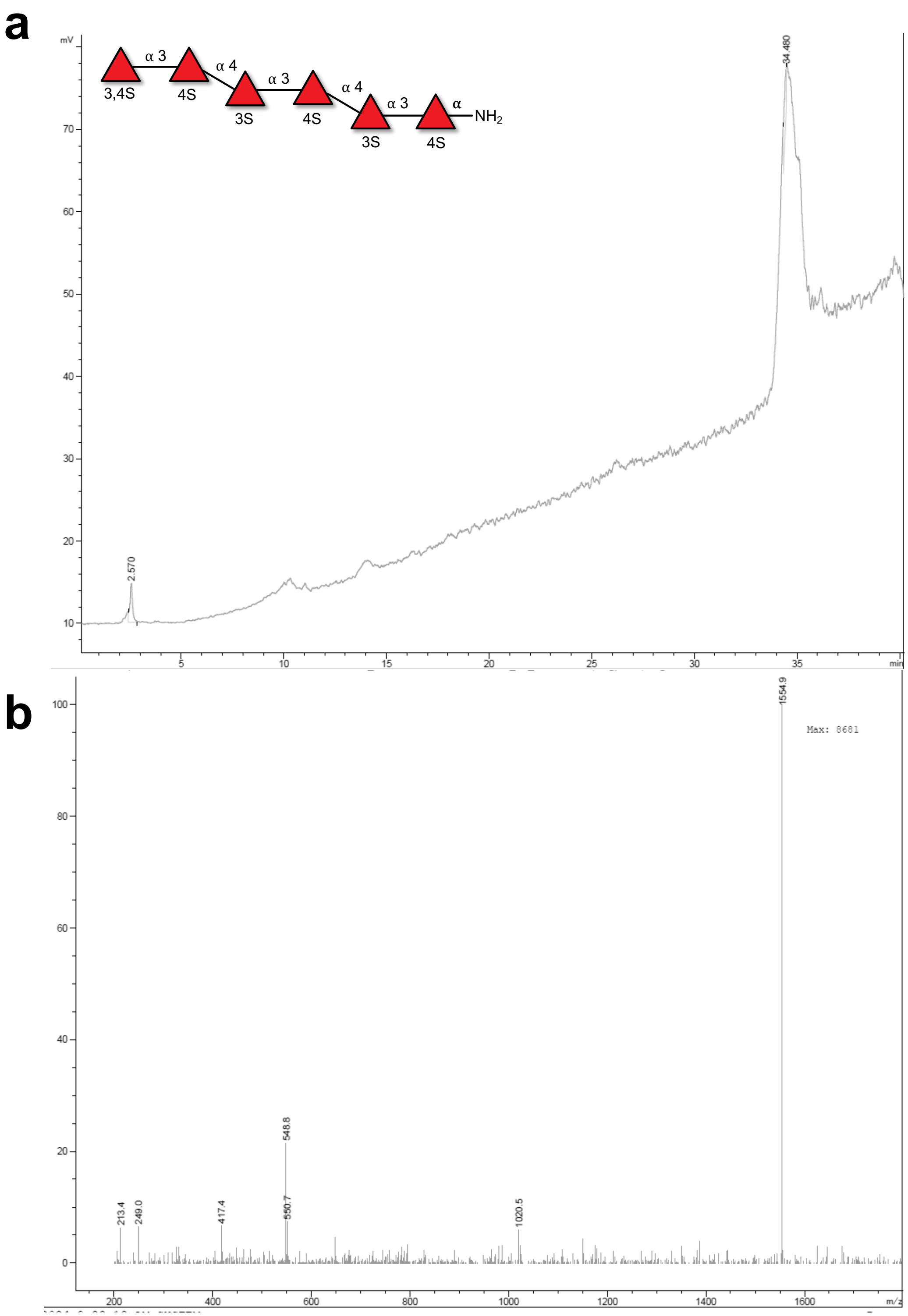

Trace of compound **17**. **a** ELSD trace. HILIC column, method 12. **b** MSD trace. $[C_{41}H_{75}N_2O_{46}S_7]^{-1}$ Expected mass, 1555.1, observed 1554.9.

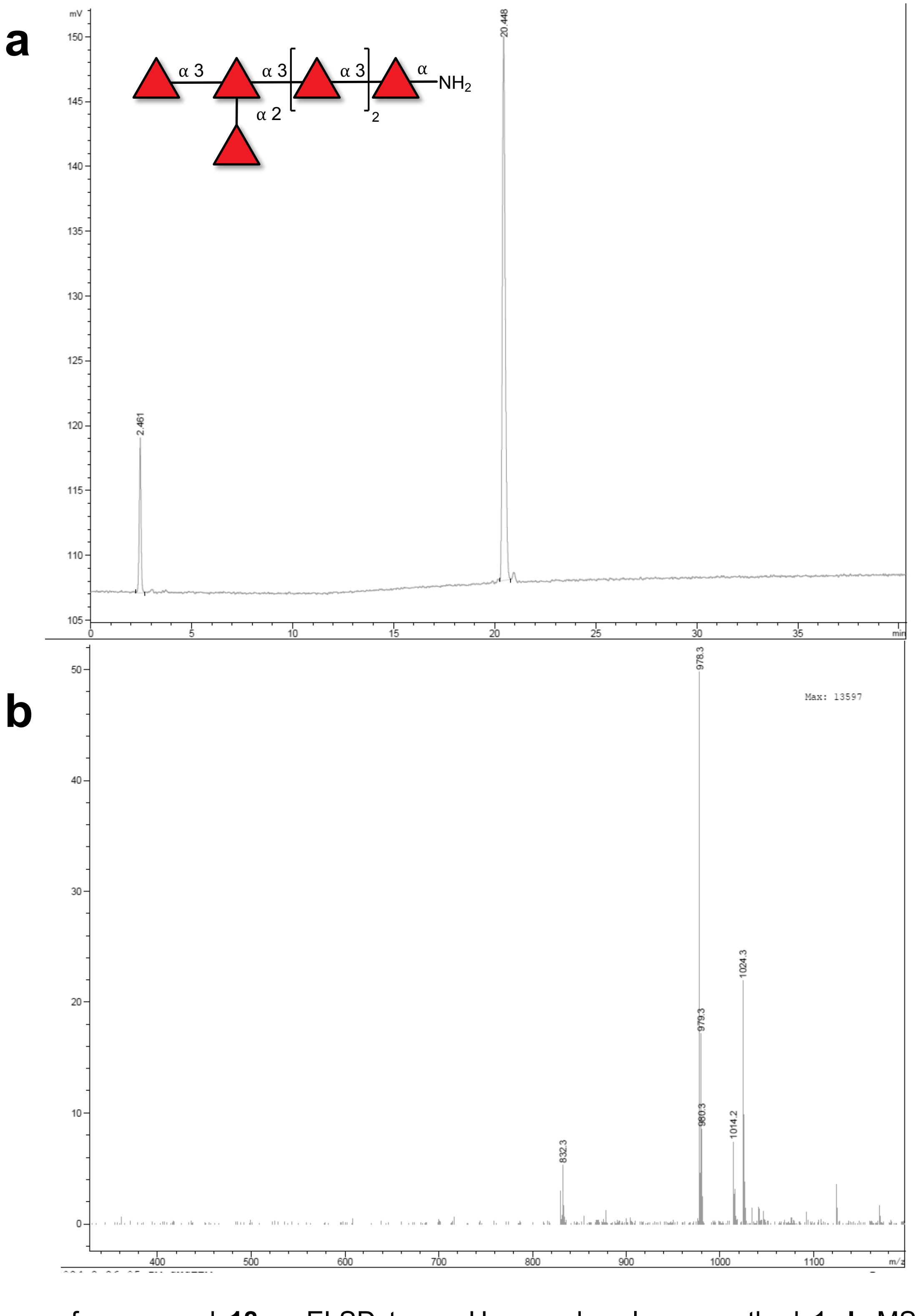

Trace of compound **18**. **a** ELSD trace. Hypercarb column, method 1. **b** MSD trace. $[C_{41}H_{72}NO_{25}]^{-1}$Expected mass, 978.4, observed 978.3.

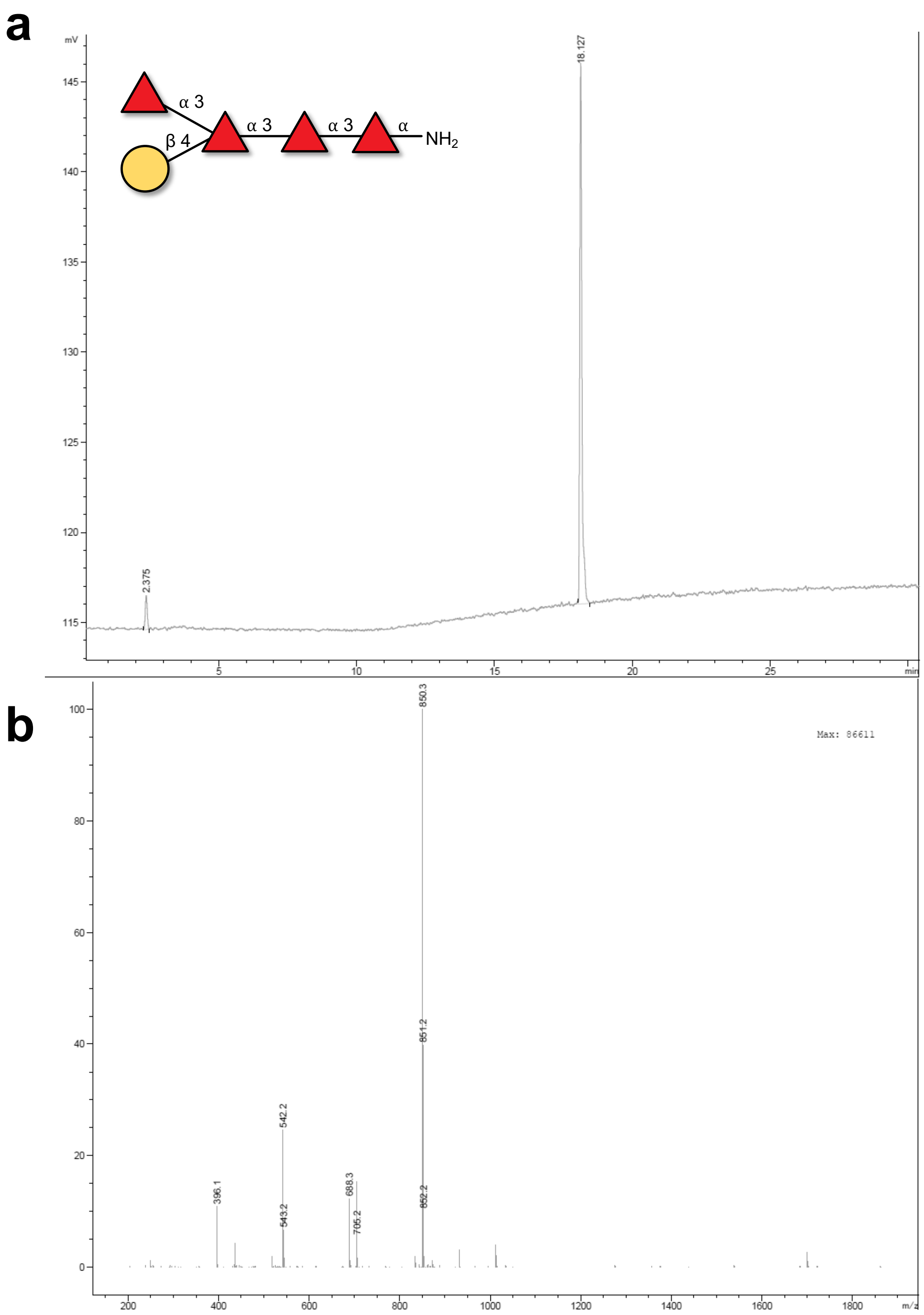

Trace of compound **19**. **a** ELSD trace. Hypercarb column, method 1. **b** MSD trace. $[C_{35}H_{64}NO_{22}]^{+1}$Expected mass, 850.3, observed 850.3.

a

b

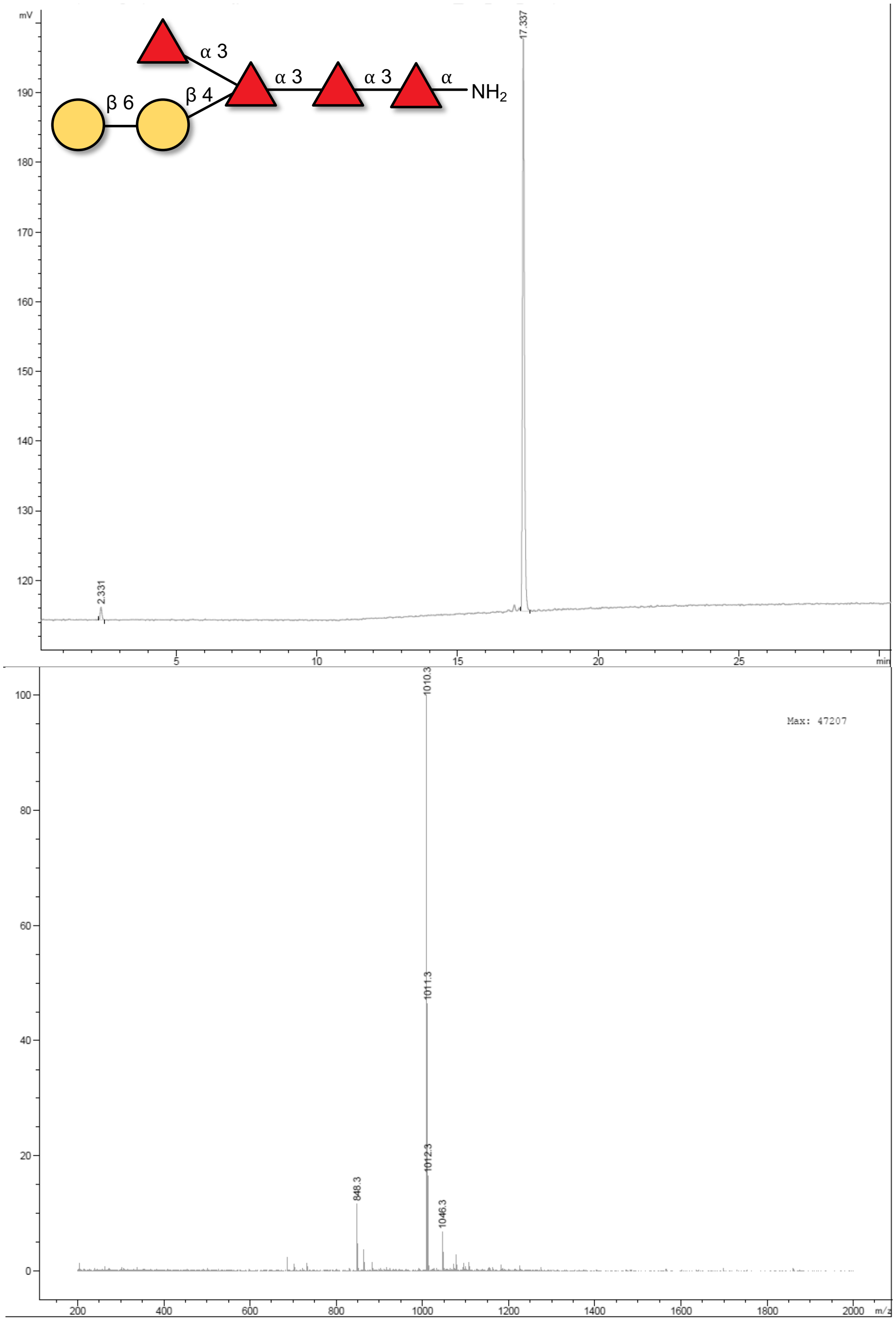

Trace of compound **20**. **a** ELSD trace. Hypercarb column, method 1. **b** MSD trace. $[C_{41}H_{72}NO_{27}]^{-1}$Expected mass, 1010.4, observed 1010.3.

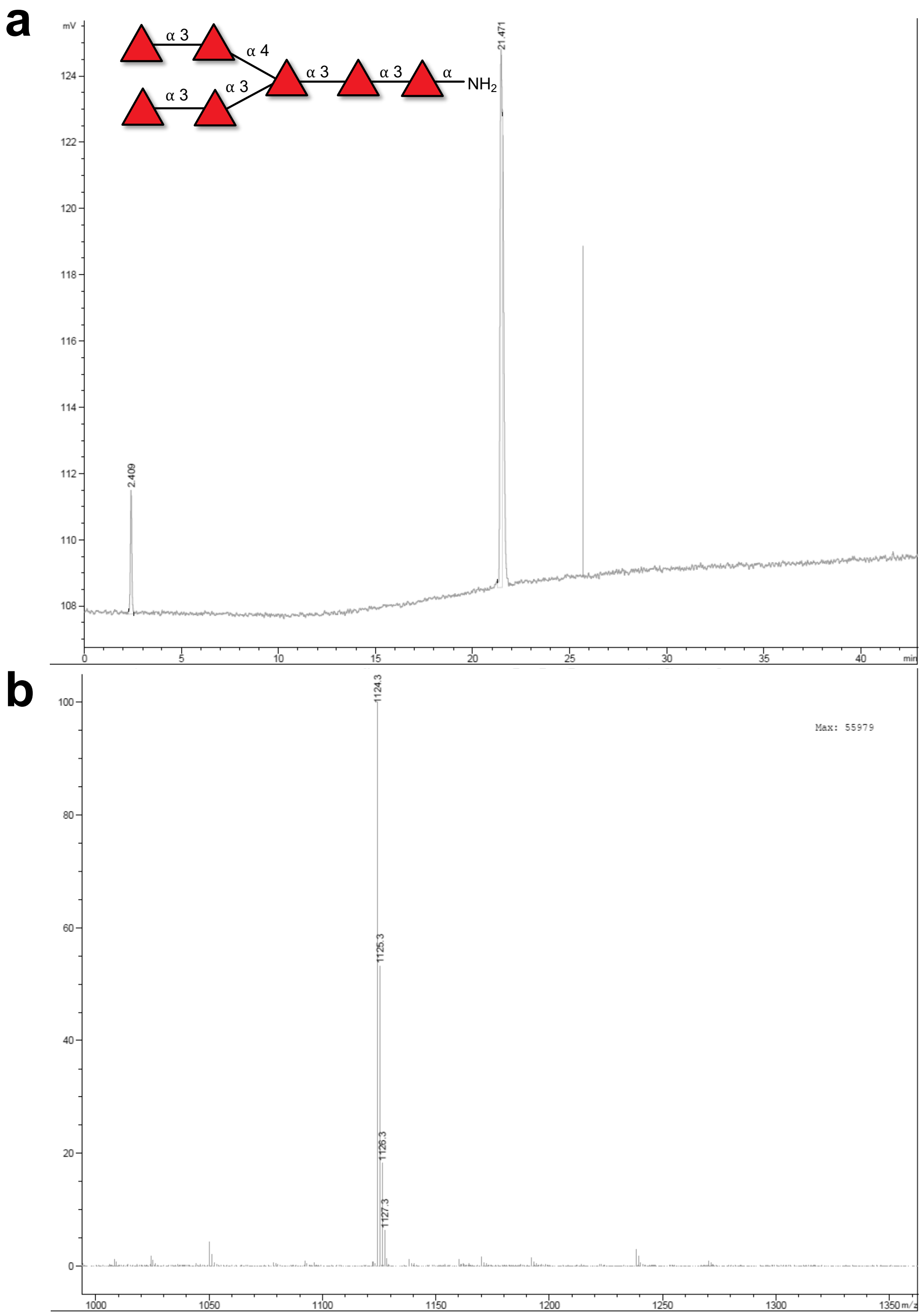

Trace of compound **21**. **a** ELSD trace. Hypercarb column, method 1. **b** MSD trace. $[C_{47}H_{82}NO_{29}]^{-1}$Expected mass, 1124.4, observed 1124.3.

α-L-fucopyranosyl-(1→3)-α-L-fucopyranosyl-(1→3)-α-L-fucopyranosyl-(1→3)-α-L-fucopyranosyl-(1→3)-α-L-fucopyranosyl-(1→3)-α-L-fucopyranose (6)

1

L2

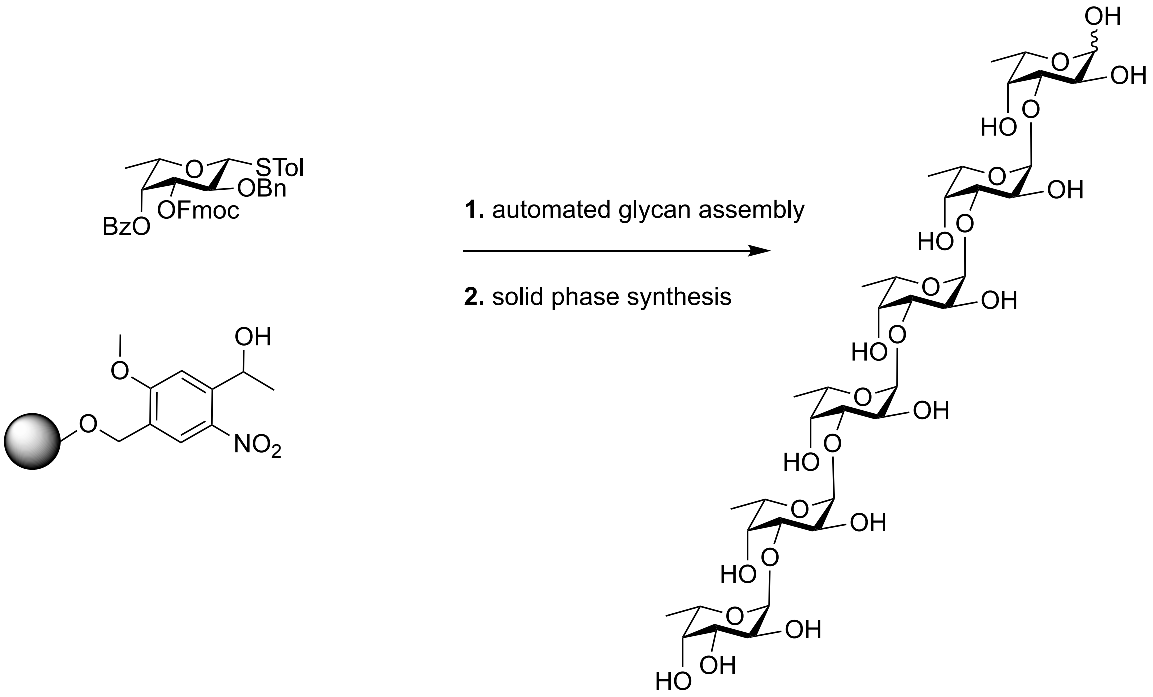

		Modules	Notes
**1. AGA**	**1**	a, b1, c, d1	L2<br>x6
	**Step**	**Module**	**Notes**
**2. Solid-phase synthesis**	Methanolysis	g	
	photocleavage	i	
	**Step**	**Module**	**Notes**
**3. Solution-phase**	Hydrogenolysis	j1	
	Purification	Method 2	

The desired fractions were then collected and lyophilized to yield 3 mg (23%). **^{1}H NMR** (400 MHz, D_2O) δ 5.19 (d, *J* = 3.5 Hz, 0H, α-H-1), 5.09 – 5.04 (m, 4H, H-1, 1→3), 5.02 (d, *J* = 4.0 Hz, 1H, H-1, 1→3), 4.57 (d, *J* = 7.9 Hz, 1H, β-H-1), 4.32 – 4.23 (m, 5H), 4.07 – 3.96 (m, 8H), 3.96 – 3.86 (m, 6H), 3.81 – 3.71 (m, 3H), 3.67 (dd, *J* = 9.9, 3.2 Hz, 1H), 3.57 – 3.48 (m, 1H), 1.25 – 1.13 (m, 18H, CH_3).**^{13}C NMR** (101 MHz, D_2O) δ 96.0 (β-C-1), 95.5 (C-1, 1→3), 95.4 (C-1, 1→3), 95.4 (C-1, 1→3), 95.2 (C-1, 1→3), 77.4, 74.7, 74.6, 71.8, 70.4, 69.8, 69.3, 68.4, 67.9, 67.6, 66.8, 66.5, 66.4, 66.37, 66.30, 15.5 (CH_3), 15.2 (CH_3), 15.1 (CH_3). **HRMS** QTOF-MS: calcd. $C_{36}H_{62}NaO_{25}$ for $[M+Na]^+$ 917.3478, found 917.3478.

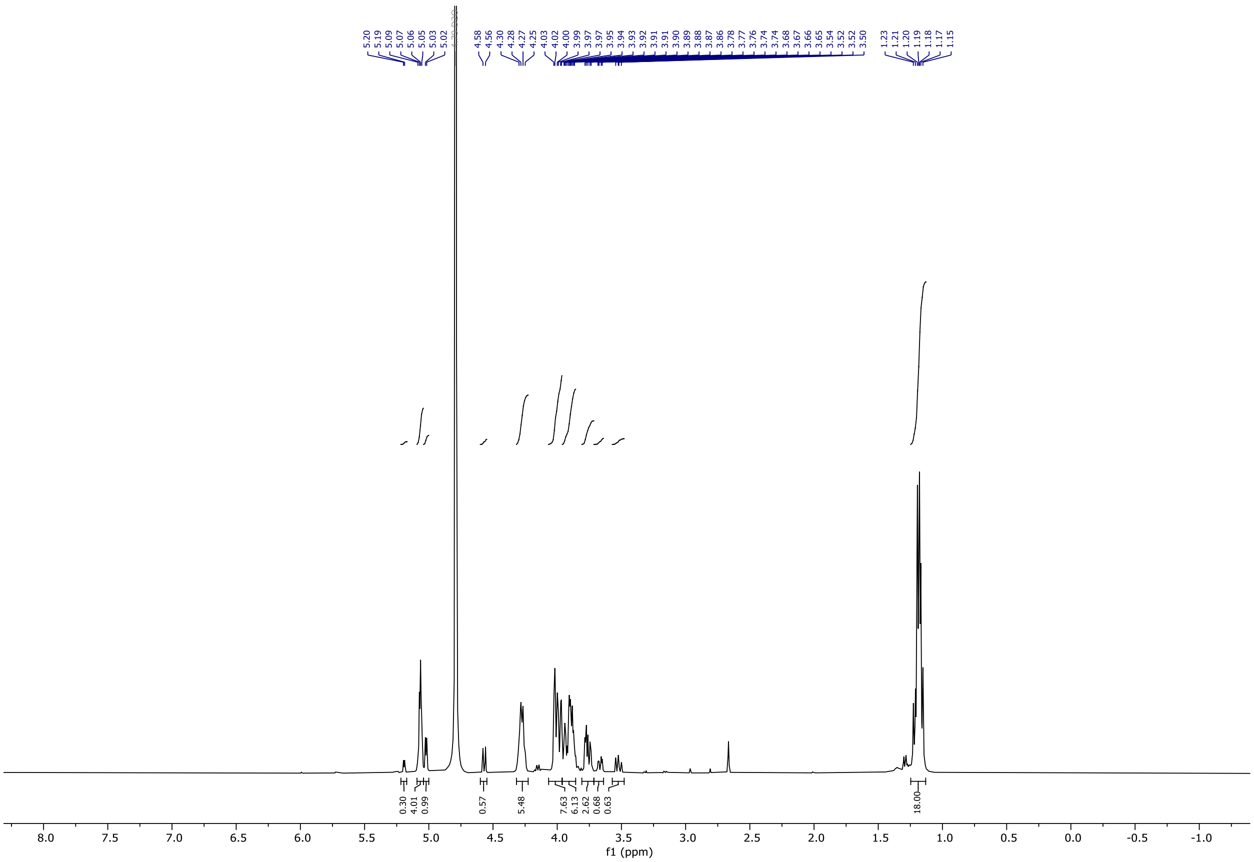

^{1}H NMR

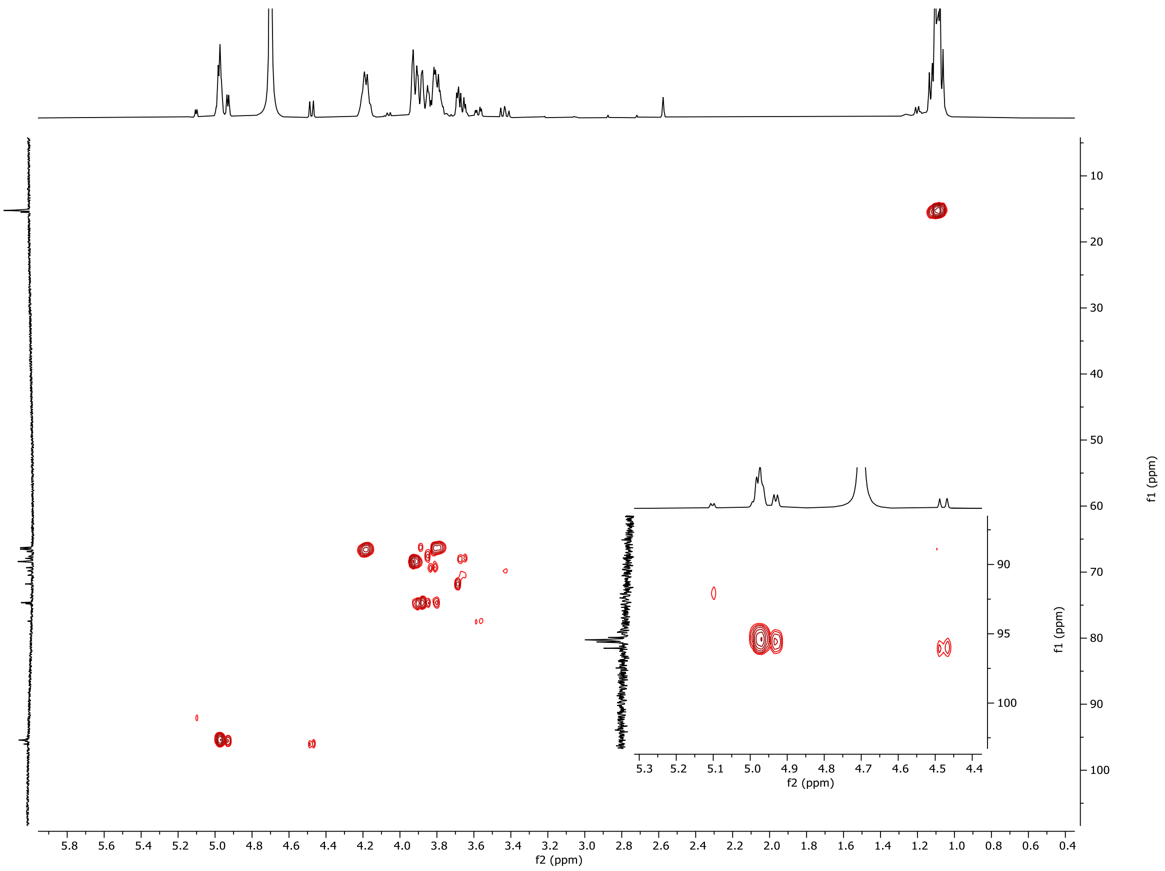

^{1}H-^{13}C HSQC NMR. Inset is of zoomed anomeric region.

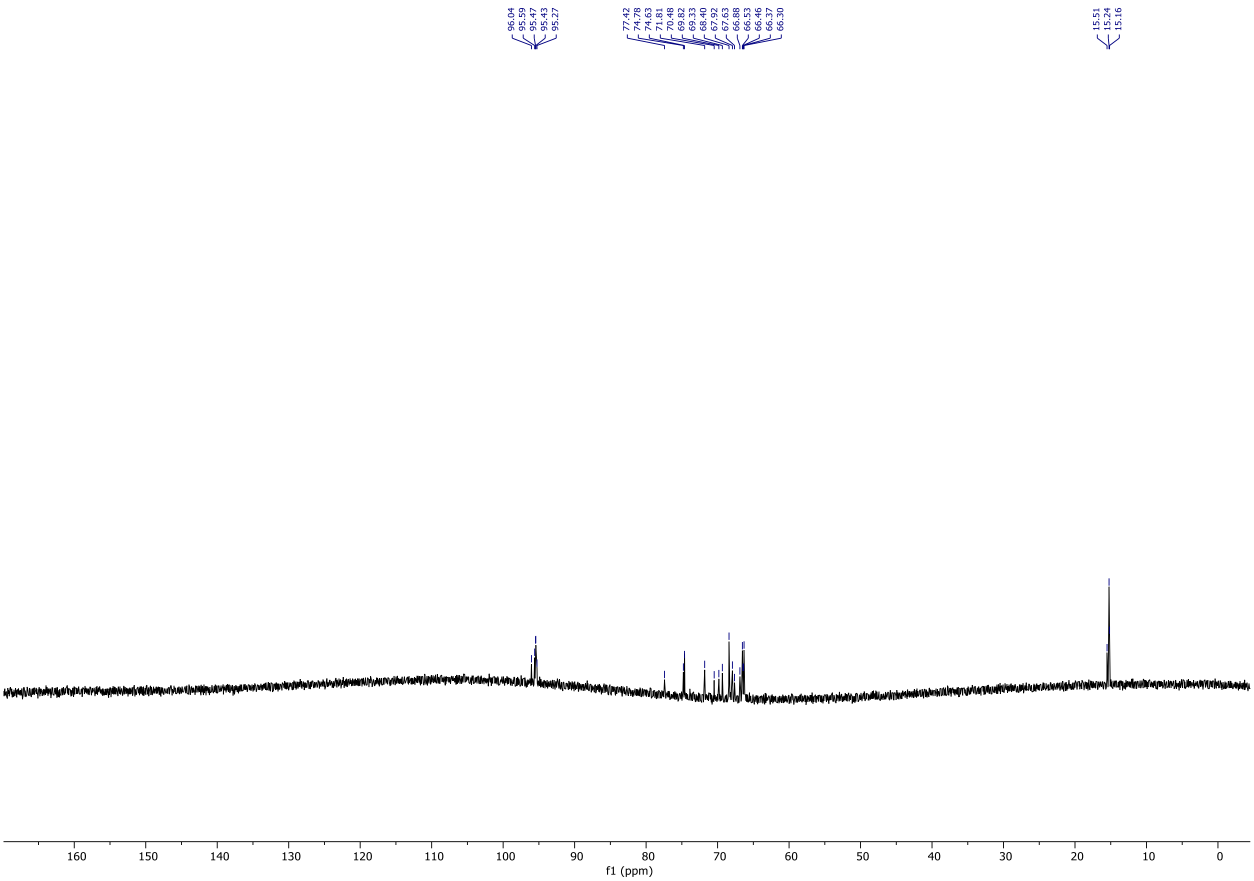

^{13}C NMR

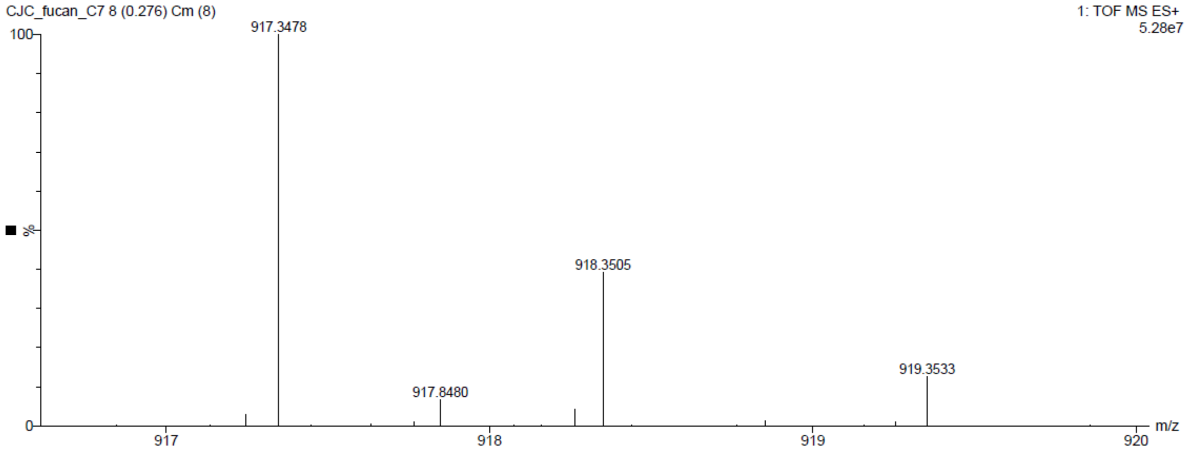

Q-TOF MS

Amino pentyl α-L-fucopyranosyl-(1→3)-α-L-fucopyranosyl-(1→3)-α-L-fucopyranosyl-(1→3)-α-L-fucopyranosyl-(1→3)-α-L-fucopyranoside (7)

1

L1

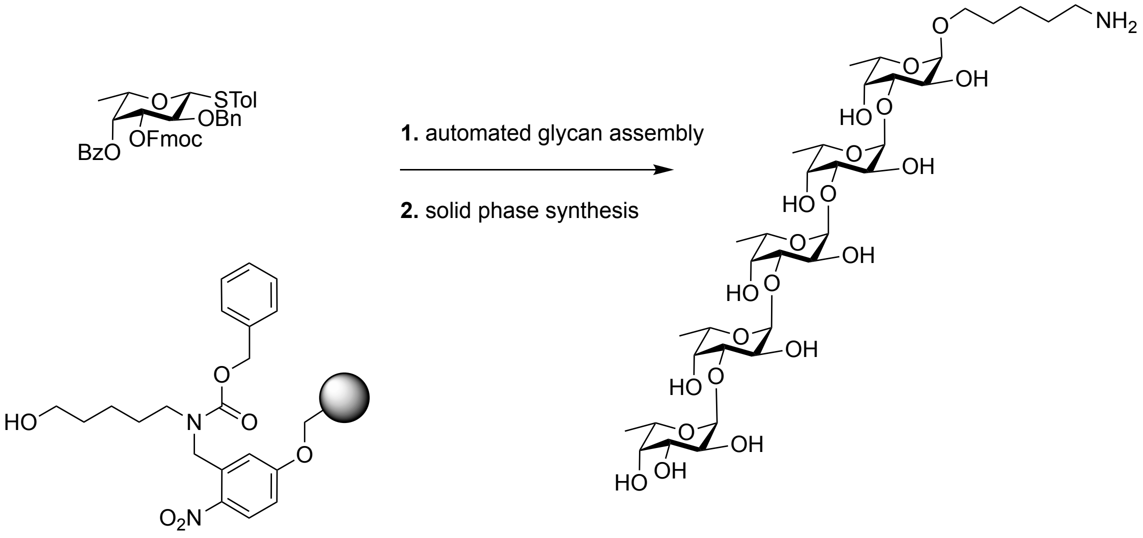

		Modules	Notes
**1. AGA**	**1**	a, b1, c, d1	L1<br>x5
	**Step**	**Module**	**Notes**
**2. Solid-phase synthesis**	Methanolysis<br>photocleavage	g<br>i	
	**Step**	**Module**	**Notes**
**3. Solution-phase**	Hydrogenolysis<br>Purification	j1<br>Method 2	

The desired fractions were then collected and lyophilized to yield 0.9 mg (8%). **^{1}H NMR** (400 MHz, D_2O) δ 5.06 (d, *J* = 3.7 Hz, 3H, H-1, 1→3), 5.02 (d, *J* = 4.0 Hz, 1H, H-1, 1→3), 4.87 (d, *J* = 2.2 Hz, 1H, H-1, amino pentyl), 4.31 – 4.22 (m, 5H), 4.04 – 3.99 (m, 6H), 3.99 – 3.96 (m, 1H), 3.95 – 3.85 (m, 7H), 3.81 – 3.70 (m, 2H), 3.70 – 3.59 (m, 1H), 3.58 – 3.43 (m, 2H), 3.00 – 2.92 (m, 3H, $OCH_2CH_2CH_2CH_2CH_2NH_2$), 1.70 – 1.57 (m, 5H, $OCH_2CH_2CH_2CH_2CH_2NH_2$), 1.42 (m, 2H, $OCH_2CH_2CH_2CH_2CH_2NH_2$), 1.18 (d, *J* = 6.6 Hz,

10H, CH_3), 1.15 (m, 2H, CH_3). **^{13}C NMR‡** (101 MHz, D_2O) δ 98.2 (C-1, amino pentyl), 95.6 (C-1, 1→3), 95.3 (C-1, 1→3), 74.6, 74.3, 71.7, 68.2, 67.8, 67.9, 66.6, 66.2, 39.1, 28.0, 26.4, 22.4, 15.1 (CH_3) **HRMS** QTOF-MS: calcd. $C_{35}H_{64}NO_{21}$ for $[M+H]^+$ 834.3971, found 834.3950.

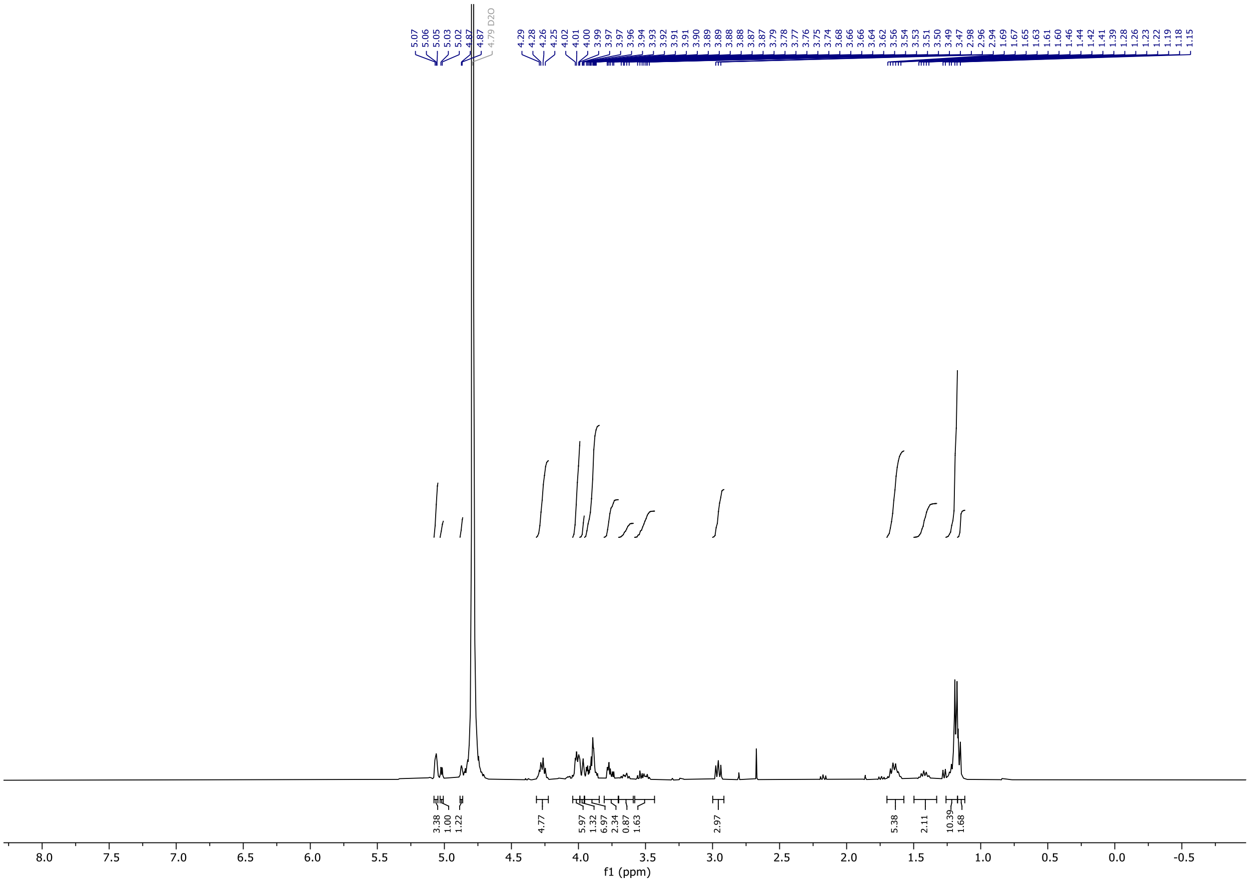

^{1}H NMR

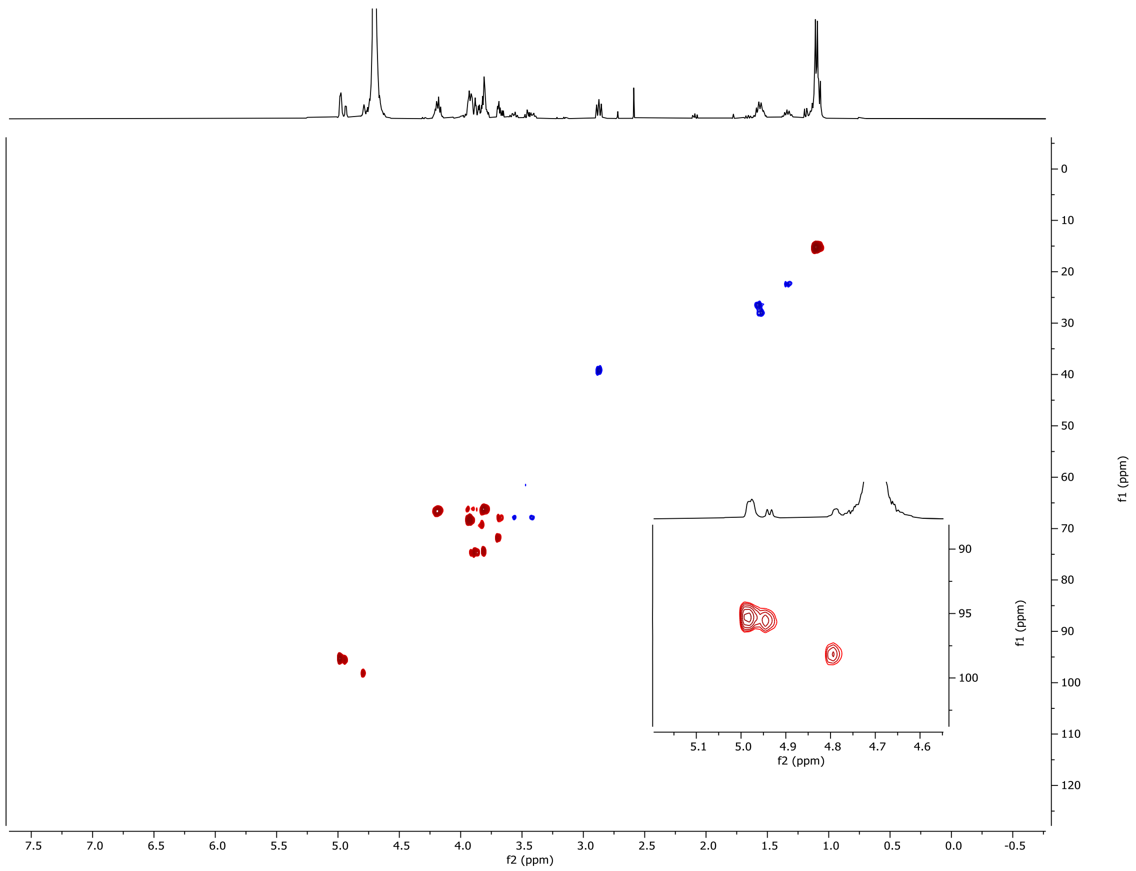

^{1}H-^{13}C HSQC NMR. Inset is of zoomed anomeric region.

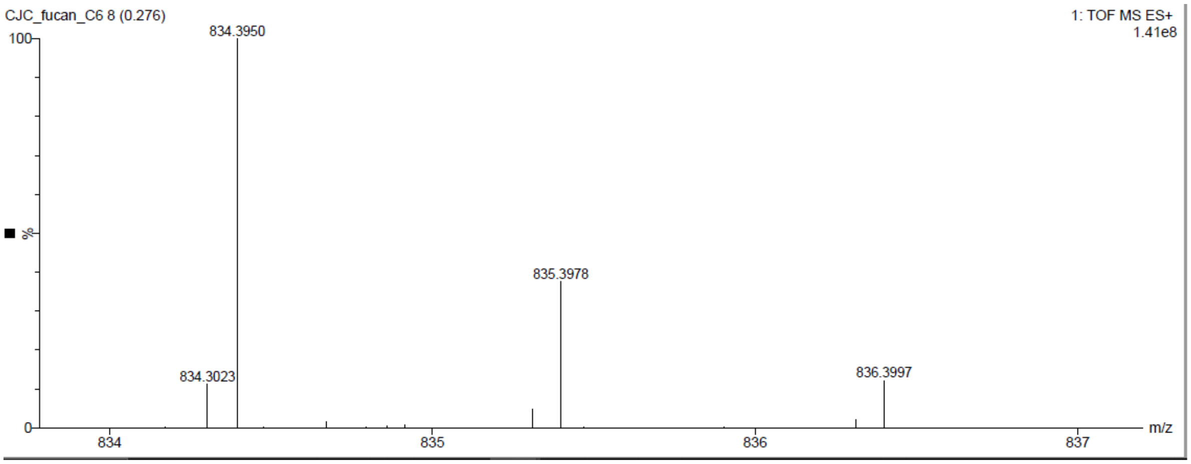

Q-TOF MS

5-amino pentyl α-L-fucopyranosyl-(1→3)-α-L-fucopyranosyl-(1→3)-α-L-fucopyranosyl-(1→3)-α-L-fucopyranosyl-(1→3)-α-L-fucopyranosyl-(1→3)-α-L-fucopyranosyl-(1→3)-α-L-fucopyranosyl-(1→3)-α-L-fucopyranoside (8)

1

L1

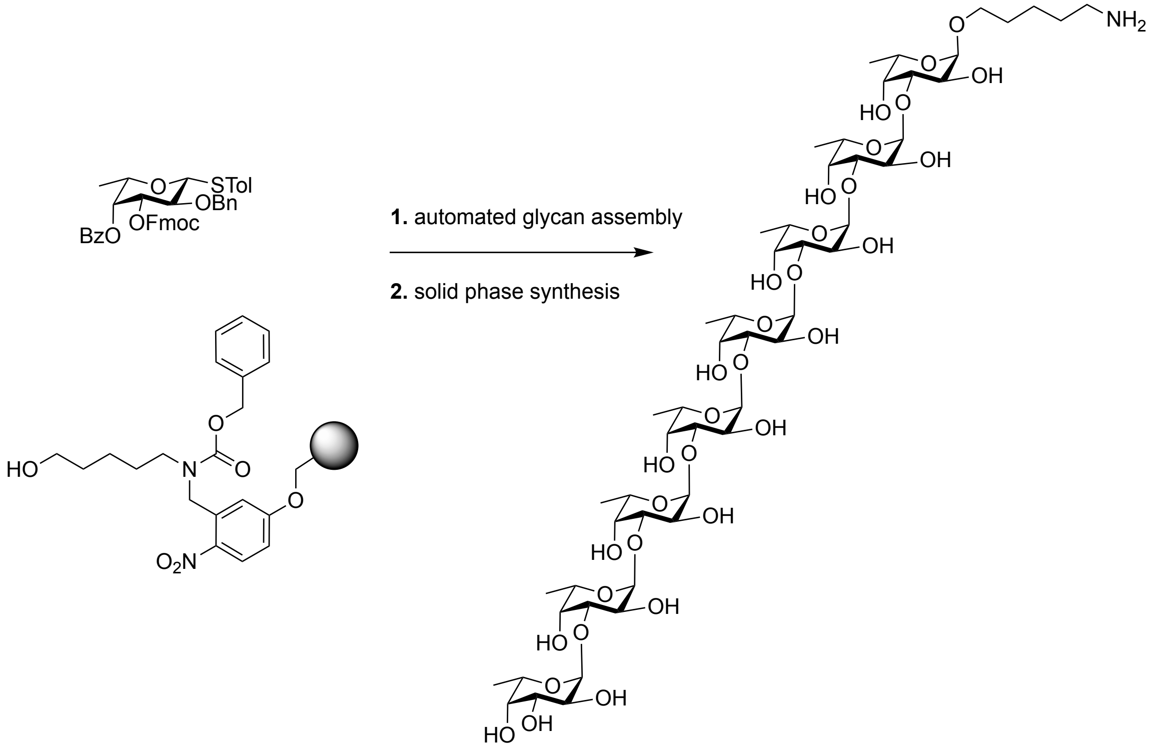

		Modules	Notes
**1. AGA**			L1
	**1**	a, b1, c, d1	x8
	**Step**	**Module**	**Notes**
**2. Solid-phase synthesis**	Methanolysis	g	
	Photocleavage	i	
	**Step**	**Module**	**Notes**
	Hydrogenolysis	j1	

3. Solution-phase	Purification	Method 2	

The desired fractions were then collected and lyophilized to yield 2.2 mg (12%). **^{1}H NMR** (700 MHz, D_2O) δ 5.07 (d, *J* = 3.9 Hz, 6H, H-1, 1→3), 5.02 (d, *J* = 4.0 Hz, 1H, H-1, 1→3), 4.88 (d, *J* = 3.0 Hz, 1H, H-1, amino pentyl), 4.30 – 4.23 (m, 6H), 4.04 – 3.96 (m, 10H), 3.95 – 3.86 (m, 10H), 3.80 – 3.72 (m, 3H), 3.68 – 3.62 (m, 1H), 3.50 (dt, *J* = 9.9, 6.2 Hz, 1H), 2.99 – 2.94 (m, 3H, $OCH_2CH_2CH_2CH_2CH_2NH_2$), 1.69 – 1.57 (m, 4H, $OCH_2CH_2CH_2CH_2CH_2NH_2$), 1.49 – 1.38 (m, 2H, $OCH_2CH_2CH_2CH_2CH_2NH_2$), 1.19 (d, *J* = 6.5 Hz, 18H, CH_3), 1.17 – 1.16 (m, 8H, CH_3). **^{13}C NMR‡** (176 MHz, D_2O) δ 98.1 (C-1, amino pentyl), 95.3 (C-1, 1→3), 95.2 (C-1, 1→3), 74.5, 74.2, 71.6, 69.2, 68.2, 67.8, 67.8, 66.5, 66.1, 66.1, 39.1, 27.9, 26.3, 22.1, 15.0 (CH_3). **HRMS** QTOF-MS: calcd. $C_{53}H_{94}O_{33}$ for $[M+H]^+$ 1272.5708, found 1272.5739.

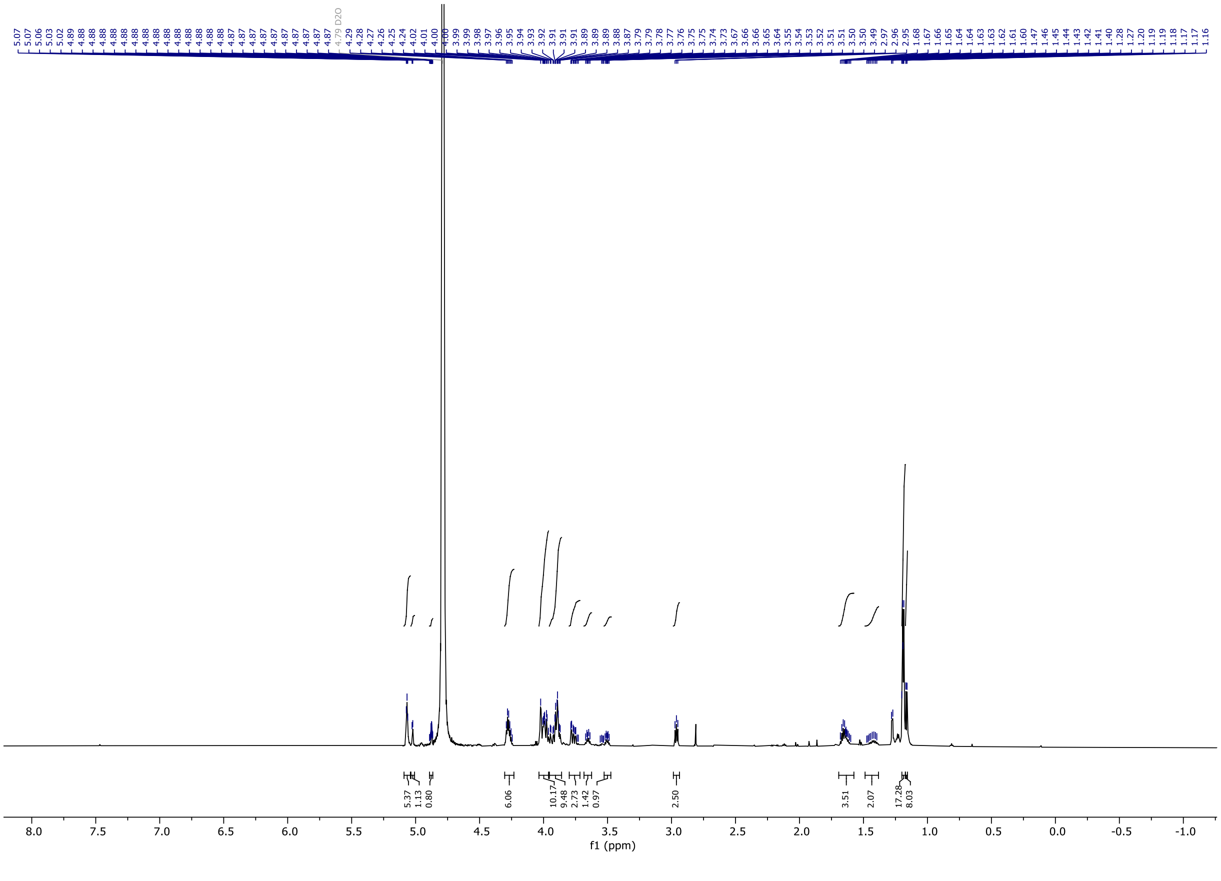

^{1}H NMR

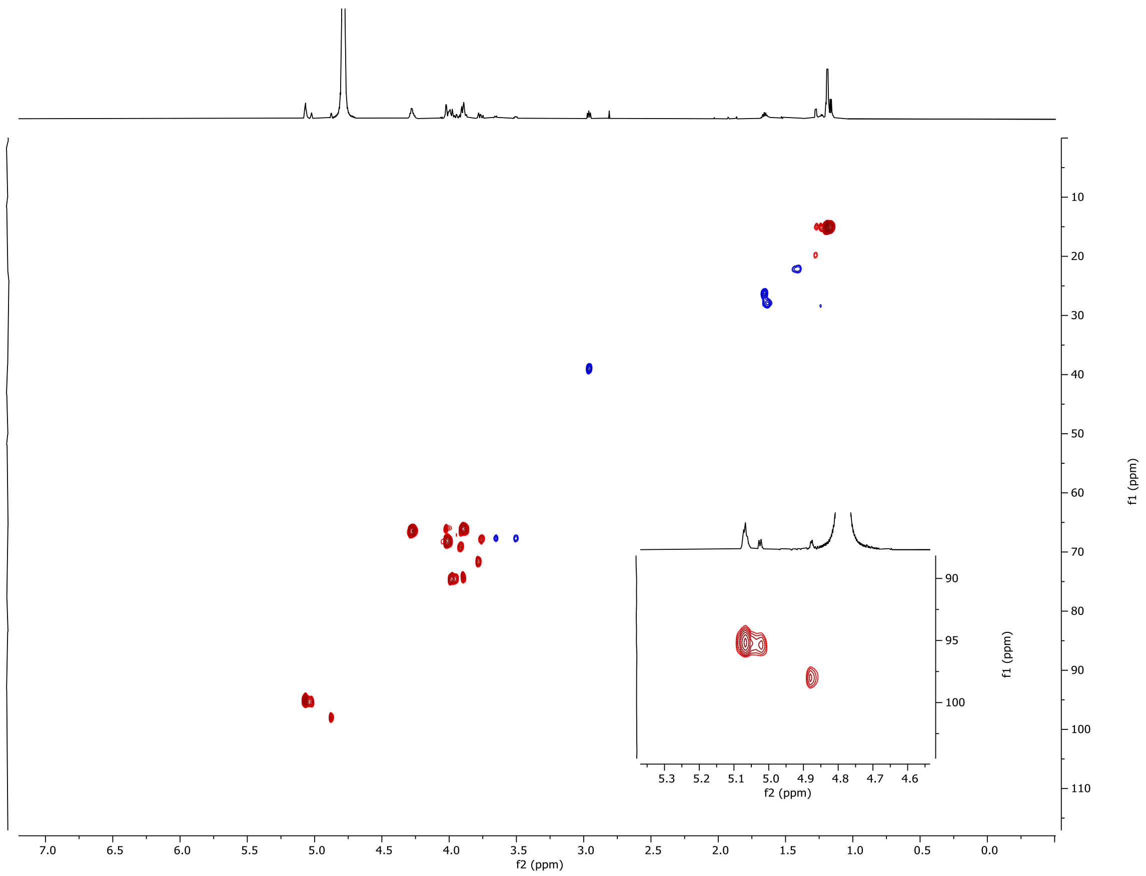

^{1}H-^{13}C HSQC NMR. Inset is of zoomed anomeric region.

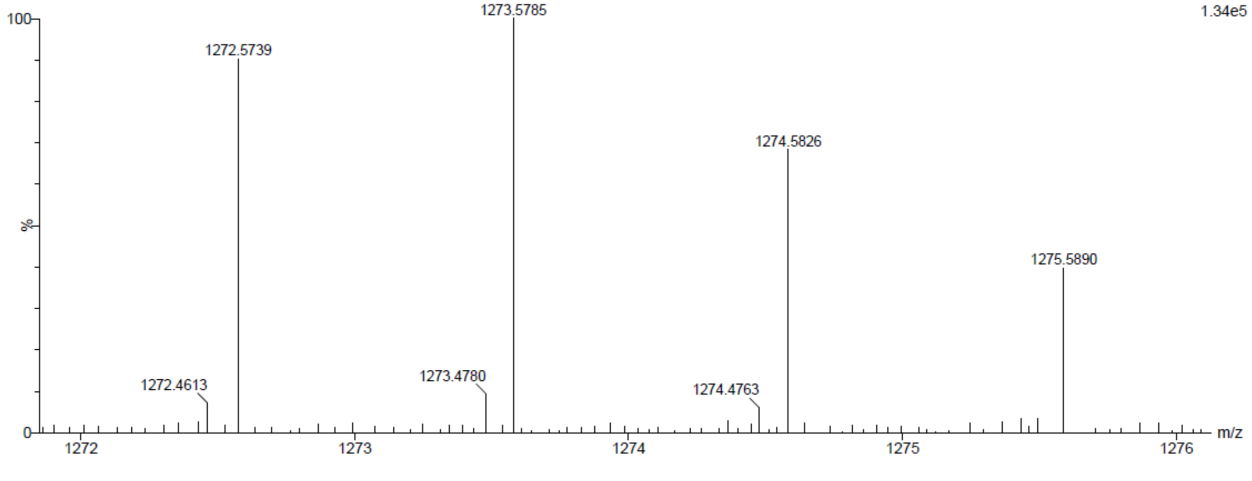

Q-TOF MS

5-Aminopentyl 3,4-di-*O*-sulfonate-α-L-fucopyranosyl-(1→3)-4-*O*-sulfonate-α-L-fucopyranoside (9)

1

L1

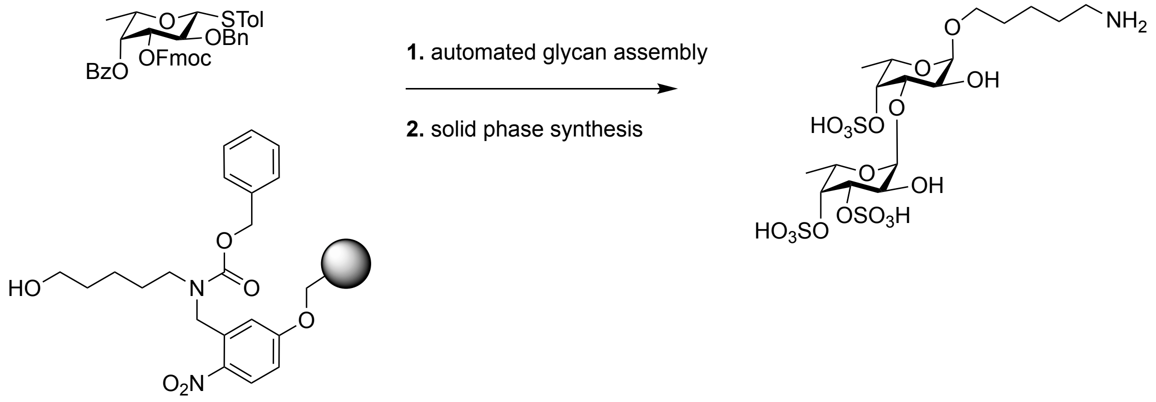

		Modules	Notes
**1. AGA**	**1**	a, b1, c, d1	L1<br>x2
	**Step**	**Module**	**Notes**
**2. Solid-phase synthesis**	Methanolysis<br>Sulfation<br>photocleavage	g<br>h<br>i	Py.SO_3, 50°C
	**Step**	**Module**	**Notes**
**3. Solution-phase**	Hydrogenolysis<br>Purification	j2<br>Method 4 and 12	

The desired fractions were then collected and lyophilized to yield 1.1 mg (12%). **^{1}H NMR** (400 MHz, D_2O) δ 5.17 (d, *J* = 3.9 Hz, 1H, H-1, 1→3), 4.88 (d, *J* = 3.7 Hz, 1H, H-1, amino pentyl), 4.84 (d, *J* = 3.1 Hz, 1H, 4-*O*-sulfation), 4.74 (s, 1H, 4-*O*-sulfation), 4.57 (dd, *J* = 10.5, 2.9 Hz, 1H, 3-*O*-sulfation), 4.45 – 4.38 (m, 1H, H-5), 4.12 (q, *J* = 6.7 Hz, 1H, H-5), 4.04 – 3.92 (m, 2H), 3.92 – 3.82 (m, 2H, H-2), 3.76 (dd, *J* = 10.1, 2.9 Hz, 0H), 3.71 – 3.58 (m, 1H), 3.58 – 3.46 (m, 1H), 3.00 – 2.92 (m, 3H, $OCH_2CH_2CH_2CH_2CH_2NH_2$), 1.72 – 1.57

(m, 5H, $OCH_2CH_2CH_2CH_2CH_2NH_2$), 1.51 – 1.39 (m, 2H, $OCH_2CH_2CH_2CH_2CH_2NH_2$), 1.28 – 1.19 (m, 5H, CH_3). **^{13}C NMR‡** (101 MHz, D_2O) δ 98.3 (C-1, amino pentyl), 96.6 (C-1, 1→3), 79.0 (4-*O*-sulfation), 78.2 (4-*O*-sulfation), 75.4 (3-*O*-sulfation), 74.1, 67.7, 66.4, 66.2, 66.1, 39.2, 28.0, 26.4, 22.4, 15.6 (CH_3). **HRMS** QTOF-MS: calcd. $C_{17}H_{32}NO_{18}S_3$ for $[M-H]^-$ 634.0787, found 634.0776.

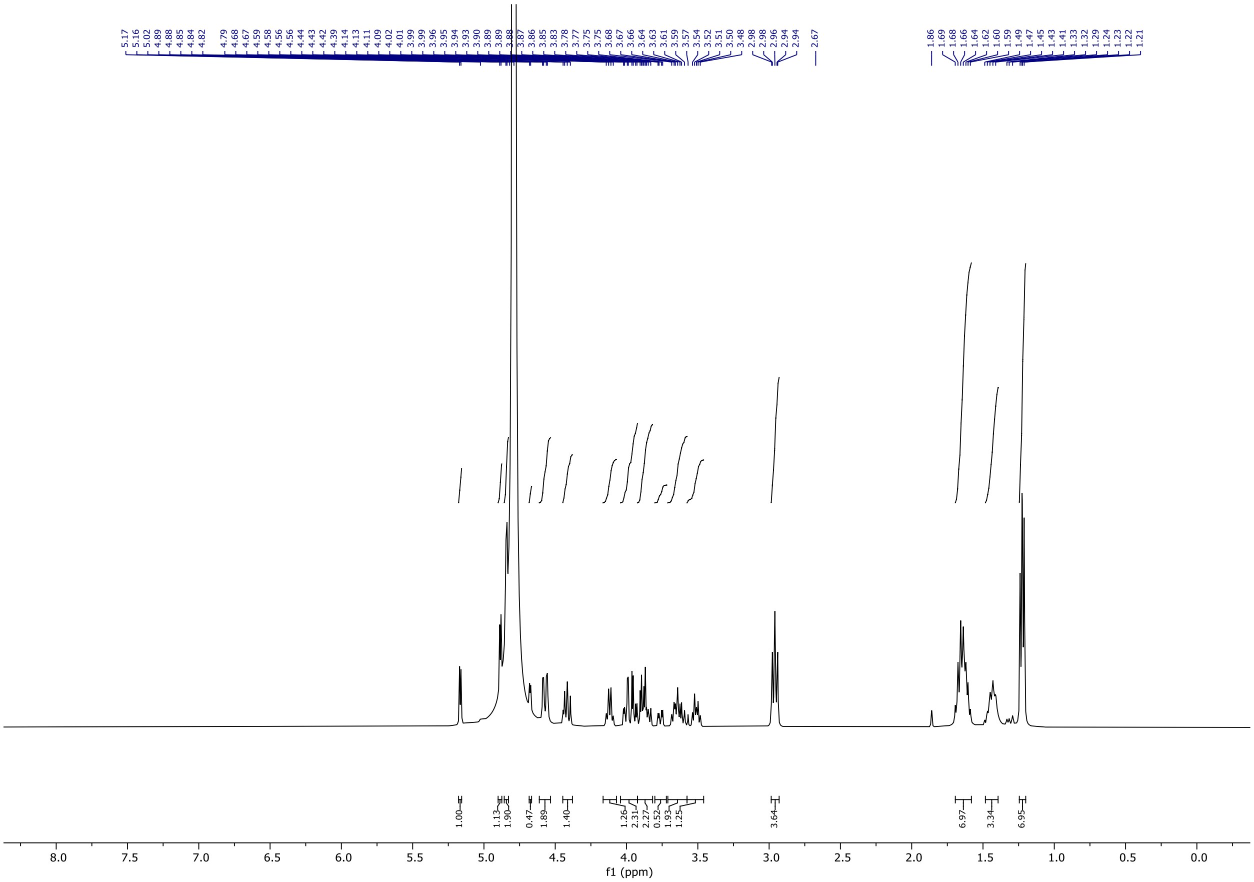

^{1}H NMR

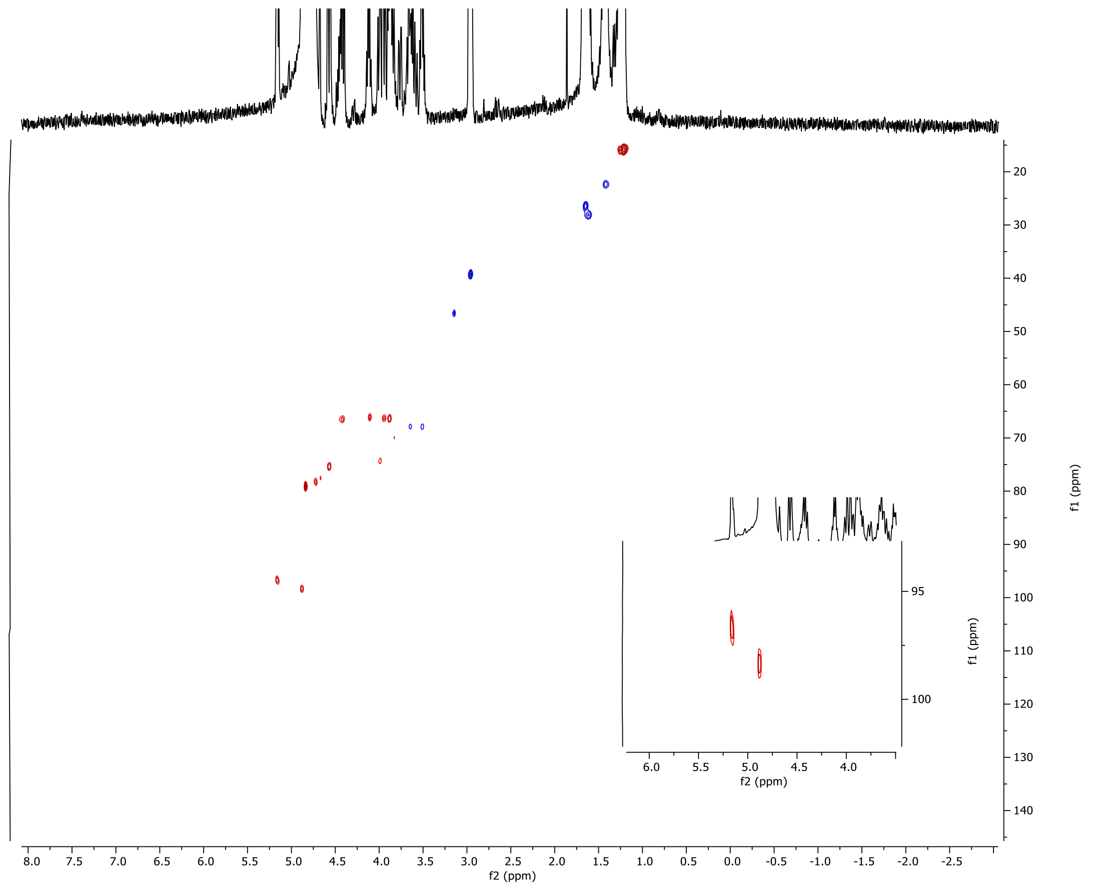

^{1}H-^{13}C HSQC NMR. Inset is of zoomed anomeric region.

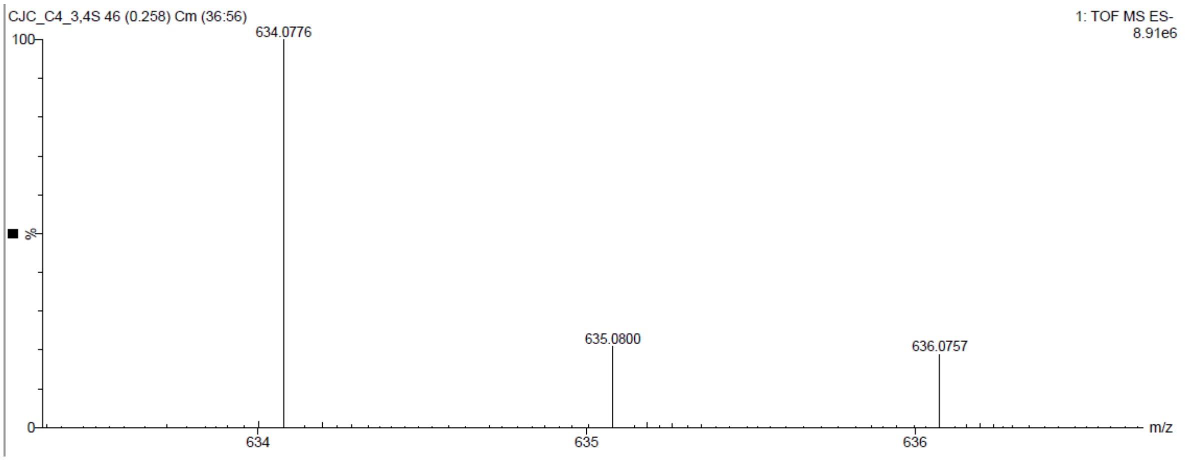

Q-TOF MS.

3,4-di-*O*-sulfonate-α-L-fucopyranosyl-(1→3)-4-*O*-sulfonate-α-L-fucopyranosyl-(1→3)-4-*O*-sulfonate-α-L-fucopyranosyl-(1→3)-4-*O*-sulfonate-α-L-fucopyranosyl-(1→3)-4-*O*-sulfonate-α-L-fucopyranosyl-(1→3)-4-*O*-sulfonate-α-L-fucopyranose (10)

1

L2

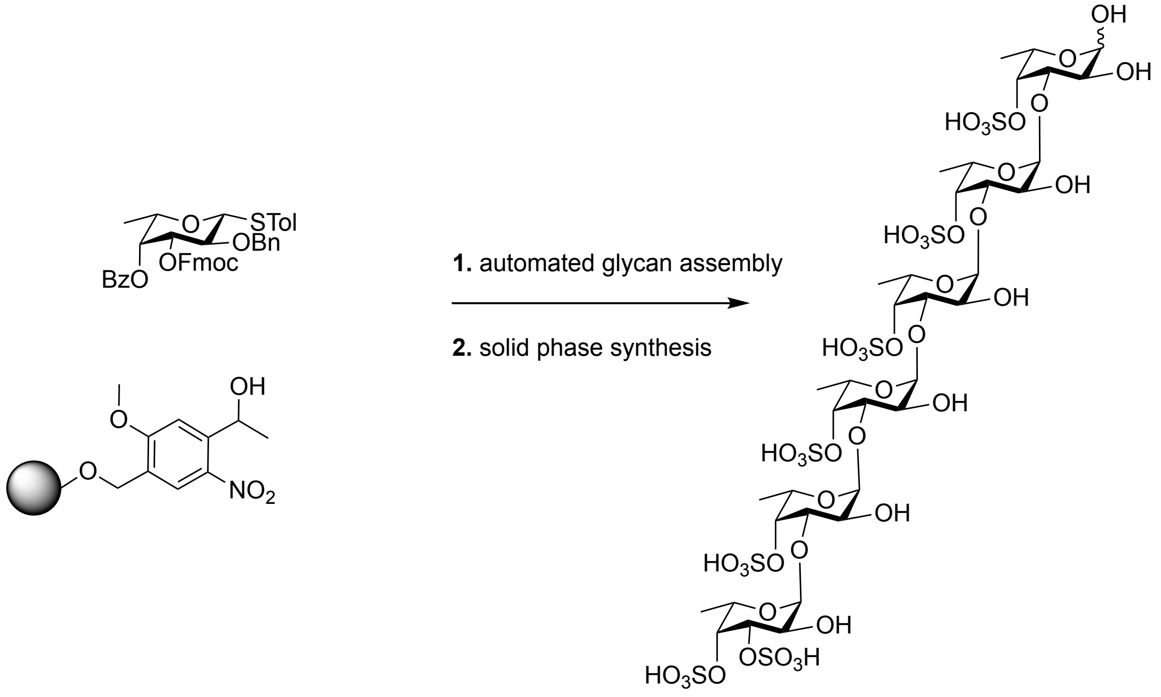

		Modules	Notes
**1. AGA**	**1**	a, b1, c, d1	L2<br>x6
	**Step**	**Module**	**Notes**
**2. Solid-phase synthesis**	Methanolysis<br>Sulfation<br>photocleavage	g<br>h1<br>i	
	**Step**	**Module**	**Notes**
**3. Solution-phase**	Hydrogenolysis<br>Purification	j2<br>Method 4 then 7	

The desired fractions were then collected and lyophilized to yield 3.3 mg (15%). **^{1}H NMR** (400 MHz, D_2O) δ 5.20 (d, *J* = 3.7 Hz, 0H, α-H-1), 5.11 (d, *J* = 4.0 Hz, 1H, H-1, 1→3), 5.07 (m, 4H, H-1, 1→3), 4.85 – 4.83 (m, 1H, 4-*O*-sulfation), 4.74 – 4.70 (m, 4H, 4-*O*-sulfation), 4.66 (d, *J* = 3.0 Hz, 1H, 4-*O*-sulfation), 4.60 – 4.53 (m, 2H, β-H-1, 3-*O*-sulfation), 4.52 – 4.36 (m, 4H), 4.04 – 3.94 (m, 4H), 3.88 – 3.81 (m, 5H), 3.74 (dd, *J* = 10.0, 3.0 Hz, 1H), 3.57 (dd, *J* = 10.0, 7.9 Hz, 1H), 1.30 – 1.15 (m, 19H, CH_3). **^{13}C NMR‡** (101 MHz, D_2O) δ 98.5 (C-1, 1→3), 97.7 (C-1, 1→3), 96.2 (β-C-1), 92.4 (α-C-1), 79.5 (4-*O*-sulfation), 79.3 (4-*O*-sulfation), 79.0, 78.3 (4-*O*-sulfation), 76.1, 76.1, 75.4 (3-*O*-sulfation), 70.1, 69.9, 69.6, 67.0, 66.9, 66.6, 15.4 (CH_3). **HRMS** QTOF-MS: calcd. $C_{36}H_{60}O_{46}S_7$ for $[M - 2H]^{2-}$ 726.0206, found 726.0211.

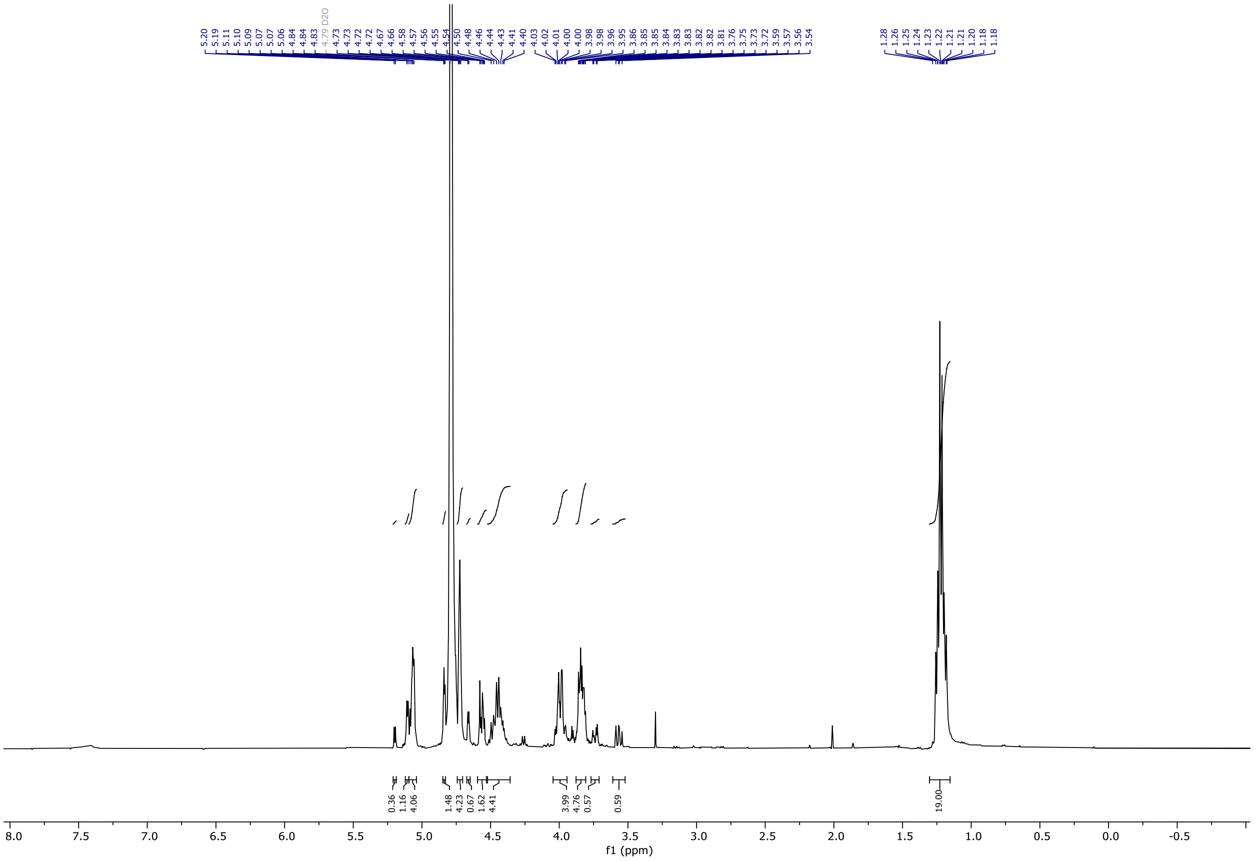

^{1}H NMR

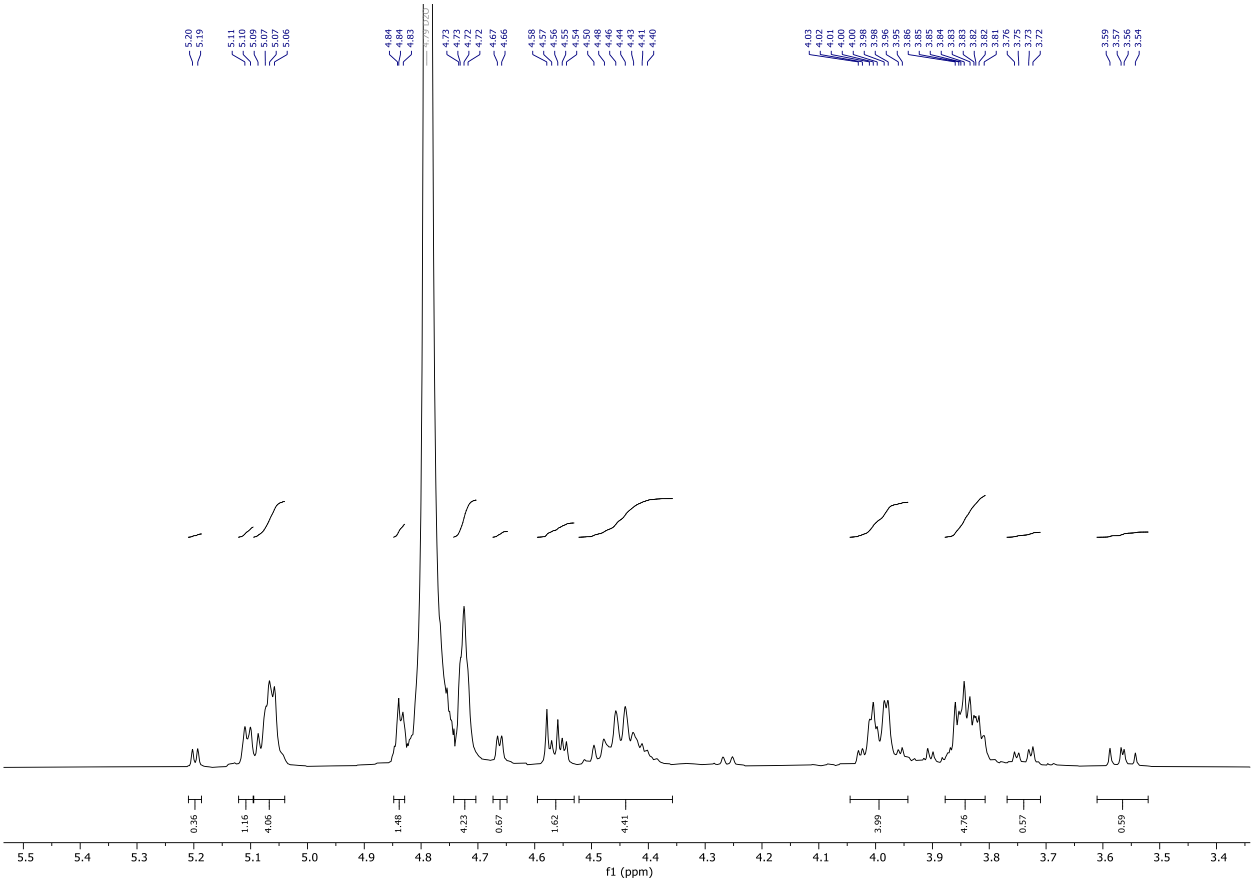

Zoomed ^{1}H NMR

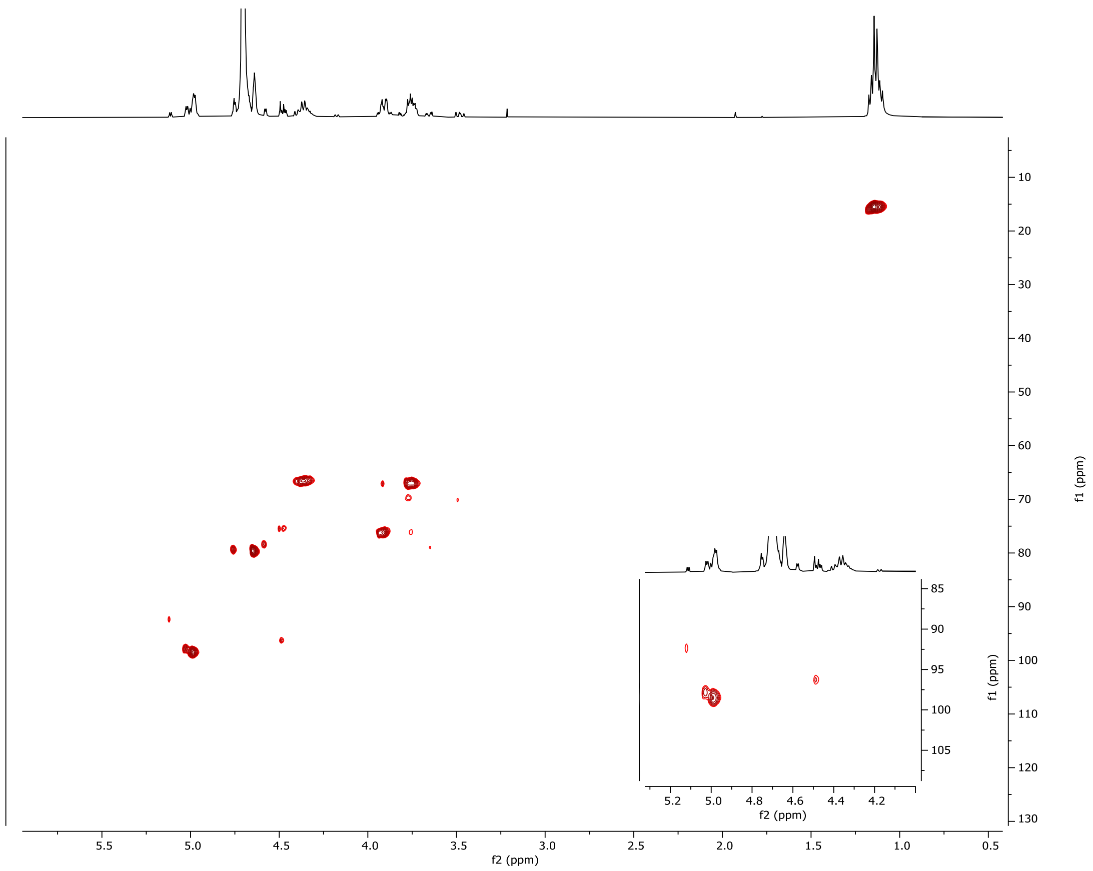

^{1}H-^{13}C HSQC NMR. Inset is of zoomed anomeric region.

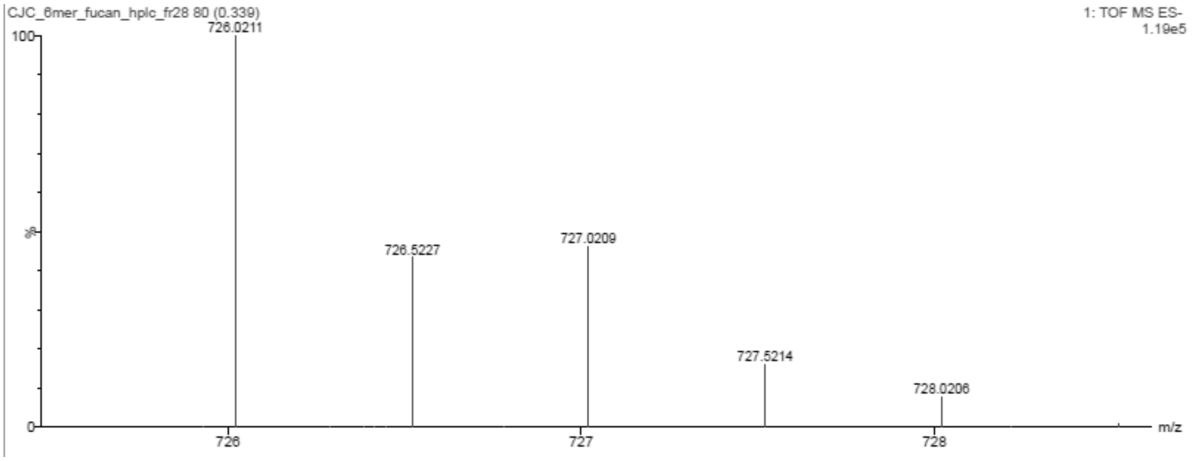

Q-TOF MS

5-Aminopentyl 3,4-di-*O*-sulfonate-α-L-fucopyranoside (11)

1

L1

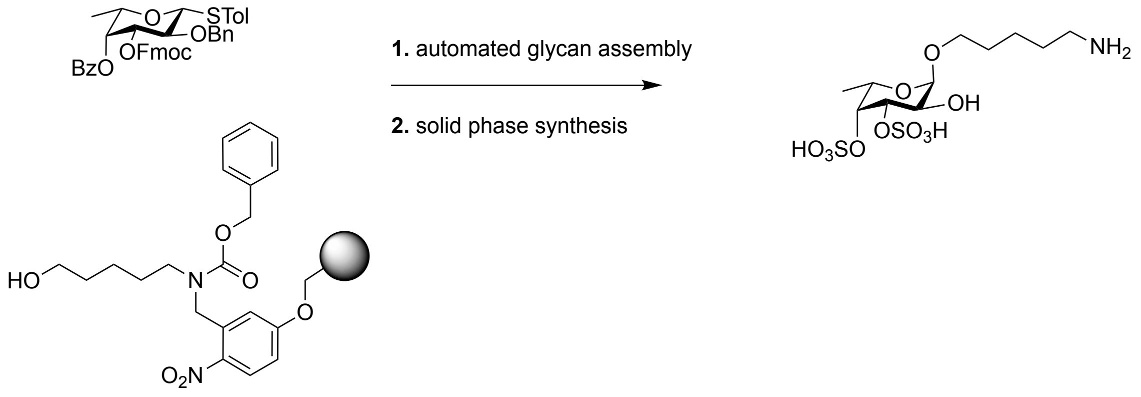

		Modules	Notes3
**1. AGA**	**1**	a, b1, c, d1	L1
	**Step**	**Module**	**Notes**
**2. Solid-phase synthesis**	Methanolysis<br>Sulfation<br>photocleavage	g<br>h1<br>i	rt
	**Step**	**Module**	**Notes**
**3. Solution-phase**	Hydrogenolysis<br>Purification	j2<br>Method 4	

The desired fractions were then collected and lyophilized to yield 2.5 mg (44%). **^{1}H NMR** (700 MHz, D_2O) δ 4.83 (d, *J* = 3.9 Hz, 1H, H-1), 4.75 (d, *J* = 3.0 Hz, 1H, H-4), 4.46 (dd, *J* = 10.3, 3.0 Hz, 1H, H-3), 4.08 (q, *J* = 6.6 Hz, 1H, H-5), 3.86 (dd, *J* = 10.4, 3.9 Hz, 1H, H-2), 3.63 – 3.56 (m, 1H, $OC\mathit{H}_2CH_2CH_2CH_2CH_2NH_2$), 3.42 (dt, *J* = 9.7, 5.9 Hz, 1H, $OC\mathit{H}_2CH_2CH_2CH_2CH_2NH_2$), 2.88 (t, *J* = 7.5 Hz, 2H, $OCH_2CH_2CH_2CH_2C\mathit{H}_2NH_2$), 1.62 – 1.50 (m, 4H, $OCH_2C\mathit{H}_2CH_2C\mathit{H}_2CH_2NH_2$), 1.43 – 1.30 (m, 2H, $OCH_2CH_2C\mathit{H}_2CH_2CH_2NH_2$), 1.16 (d, *J* = 6.6 Hz, 3H, H-6, CH_3). **^{13}C NMR**‡ (176 MHz, D_2O) δ 97.9 (C-1), 78.8 (C-4), 75.3 (C-3), 67.9, 66.1 (C-5), 65.9 (C-2), 39.2, 27.9, 26.4, 22.4,15.6 (CH_3). **HRMS** QTOF-MS: calcd. $C_{11}H_{22}NO_{11}S_2$ for $[M-H]^-$ 408.0640, found 408.0643.

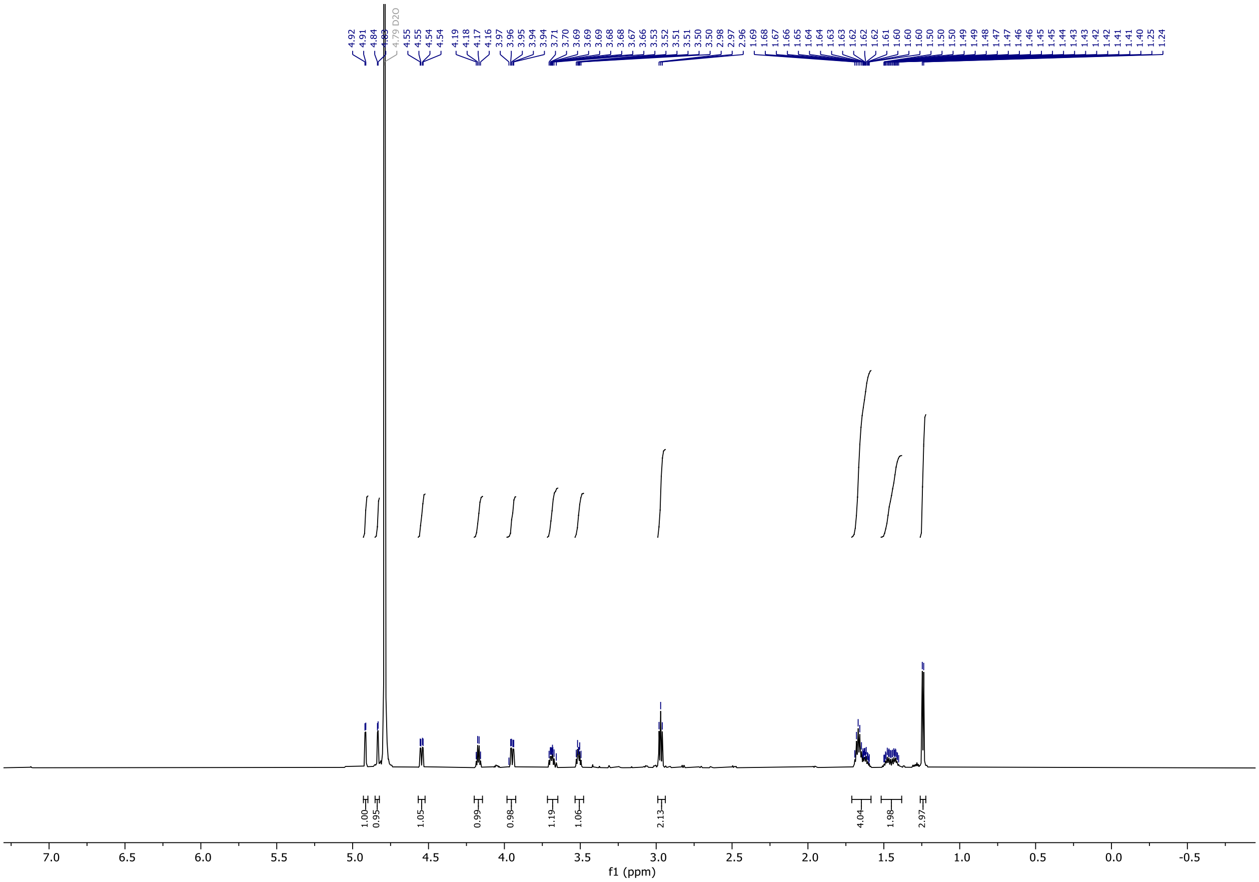

^{1}H NMR

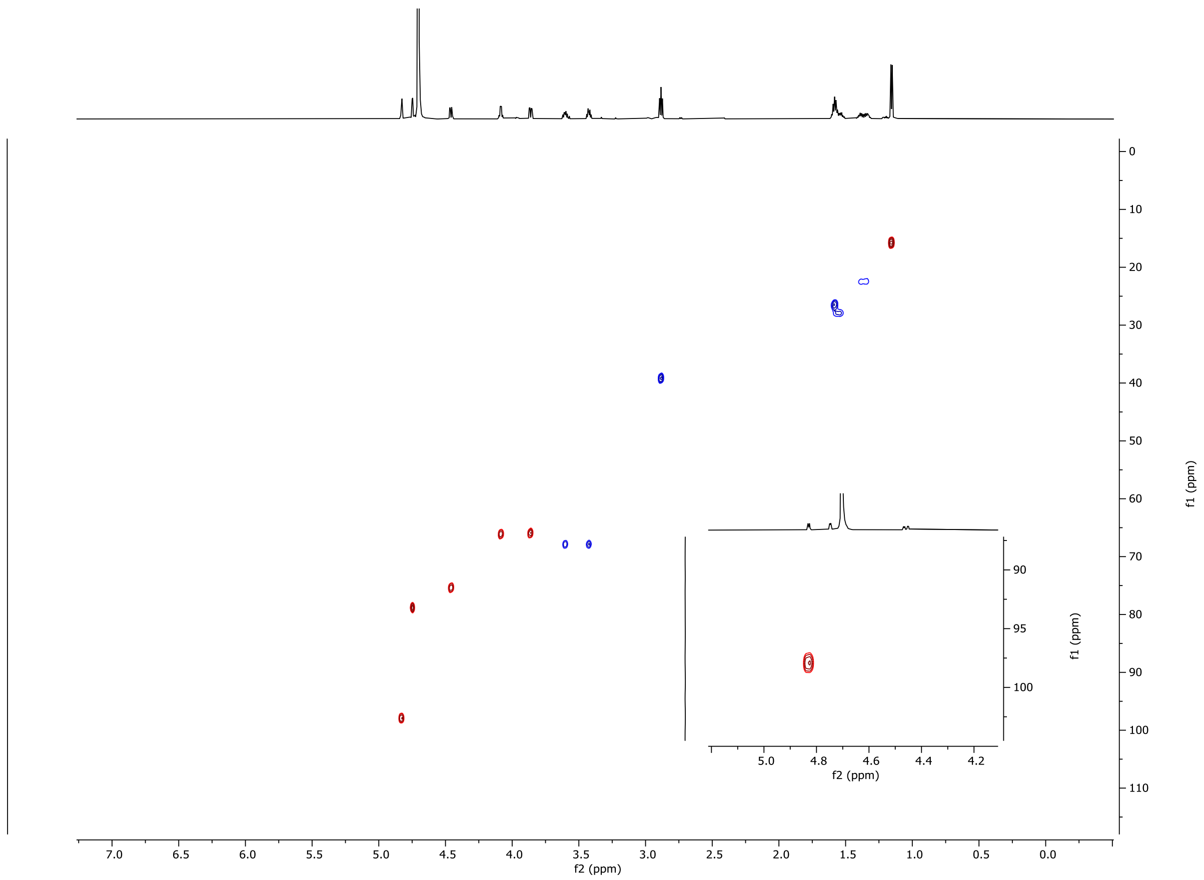

^{1}H-^{13}C **HSQC NMR.** Insert anomeric region.

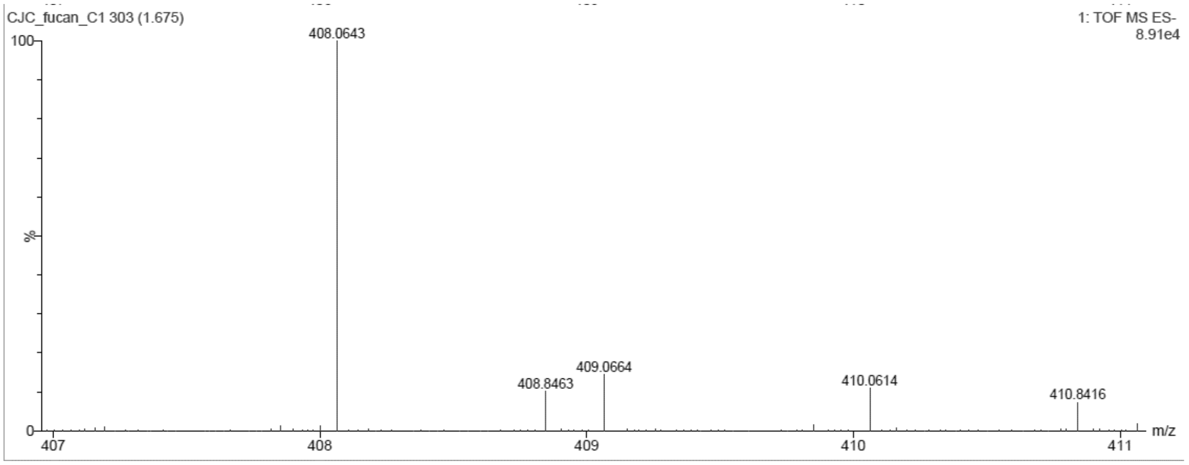

Q-TOF MS

5-aminopentyl α-L-fucopyranosyl-(1→3)-α-L-fucopyranosyl-(1→3)-α-L-fucopyranosyl-(1→3)-α-L-fucopyranosyl-(1→3)-α-L-fucopyranoside-α-L-fucopyranosyl-(1→3)-α-L-fucopyranosyl-(1→3)-α-L-fucopyranosyl-(1→3)-α-L-fucopyranosyl-(1→3)-α-L-fucopyranoside-α-L-fucopyranosyl-(1→3)-α-L-fucopyranosyl-(1→3)-α-L-fucopyranosyl-(1→3)-α-L-fucopyranosyl-(1→3)-α-L-fucopyranoside-α-L-fucopyranosyl-(1→3)-α-L-fucopyranosyl-(1→3)-α-L-fucopyranosyl-(1→3)-α-L-fucopyranosyl-(1→3)-α-L-fucopyranoside (12)

1

L1

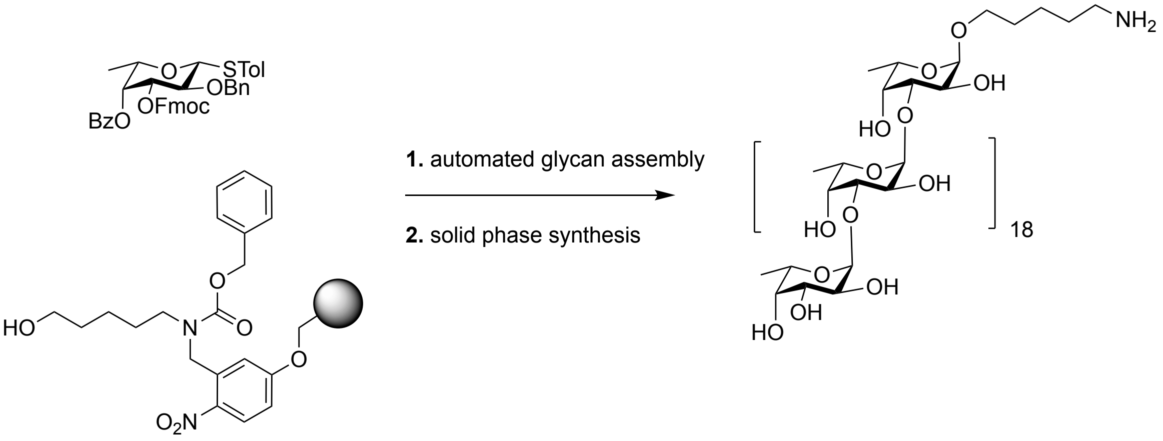

		Modules	Notes
**1. AGA**			L1
	**1**	a, b1, c, d1	x15
	**1**	a, b1, c, d1	x5 (double cycles)
	**Step**	**Module**	**Notes**
**2. Solid-phase synthesis**	Methanolysis	g	48h
	photocleavage	i	
	**Step**	**Module**	**Notes**

**3. Solution-phase**	Hydrogenolysis<br>Purification	j1<br>Method 7	

The desired fractions were then collected and lyophilized to yield 5 mg (12%). **^{1}H NMR** (600 MHz, D_2O) δ 5.07 (s, 4H, H-1, 1→3), 5.02 (d, *J* = 4.0 Hz, 1H, H-1, 1→3), 4.87 (s, 1H, H-1), 4.28 (s, 5H, H-4), 4.03 – 4.01 (m, 3H, H-5), 4.01 – 3.99 (m, 3H, H-3), 3.99 – 3.96 (m, 3H), 3.95 – 3.86 (m, 9H, H-2), 3.79 – 3.74 (m, 4H), 1.61 (s, 5H, $OCH_2C\mathit{H}_2CH_2C\mathit{H}_2CH_2NH_2$), 1.49 (s, 3H, $OCH_2CH_2C\mathit{H}_2CH_2CH_2NH_2$), 1.22 (d, *J* = 6.4 Hz, 2H, CH_3), 1.19 (d, *J* = 5.6 Hz, 10H, CH_3), 1.16 (d, *J* = 6.6 Hz, 4H, CH_3). **^{13}C NMR**‡ δ 95.7 (C-1, 1→3), 95.5 (C-1, 1→3), 74.8, 74.6, 71.9, 69.4, 68.5, 67.3, 66.7, 66.4, 15.6 (CH_3), 15.3 (CH_3). **HRMS** QTOF-MS: calcd. $C_{125}H_{214}NNaO_{81}$ for $[M+H]^{+2}$ 1524.6289, found 1524.6373.

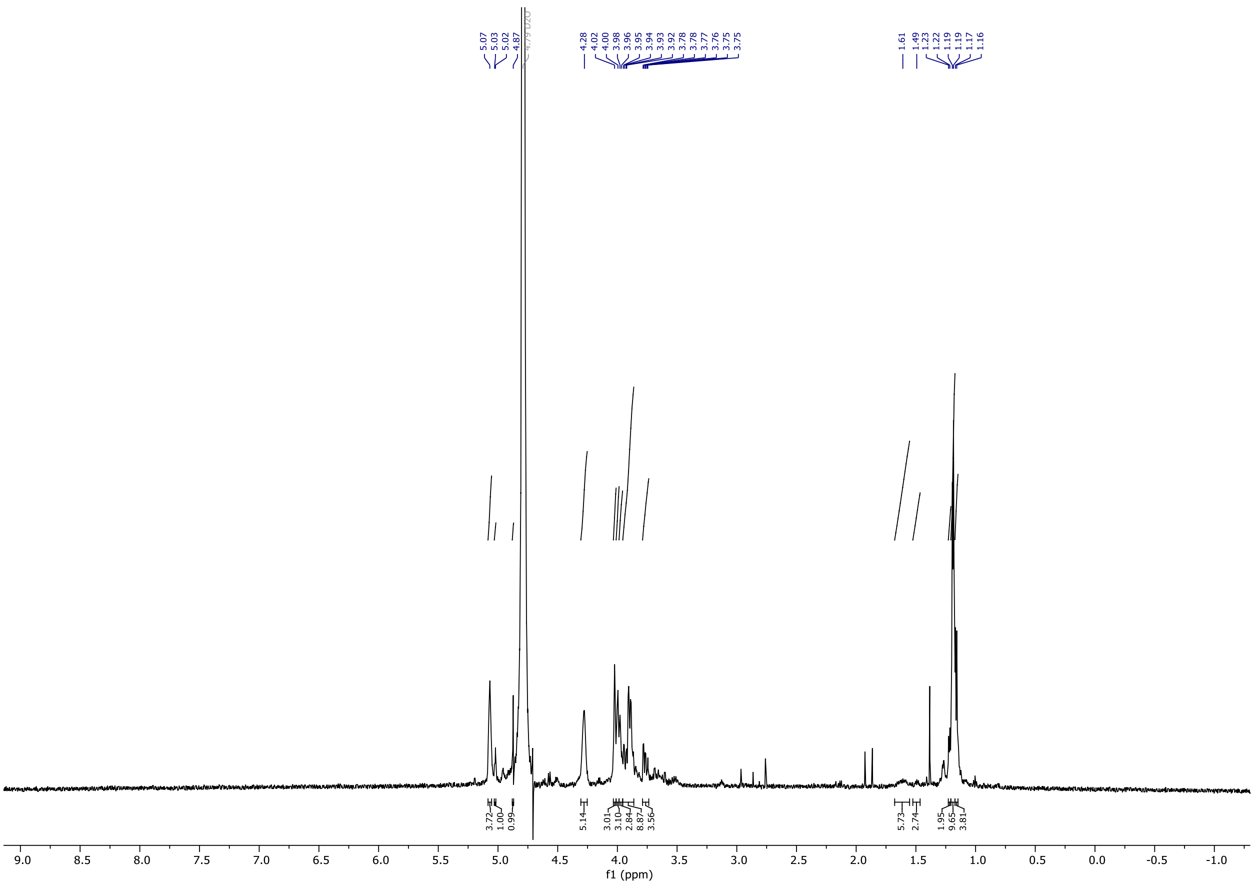

^{1}H NMR

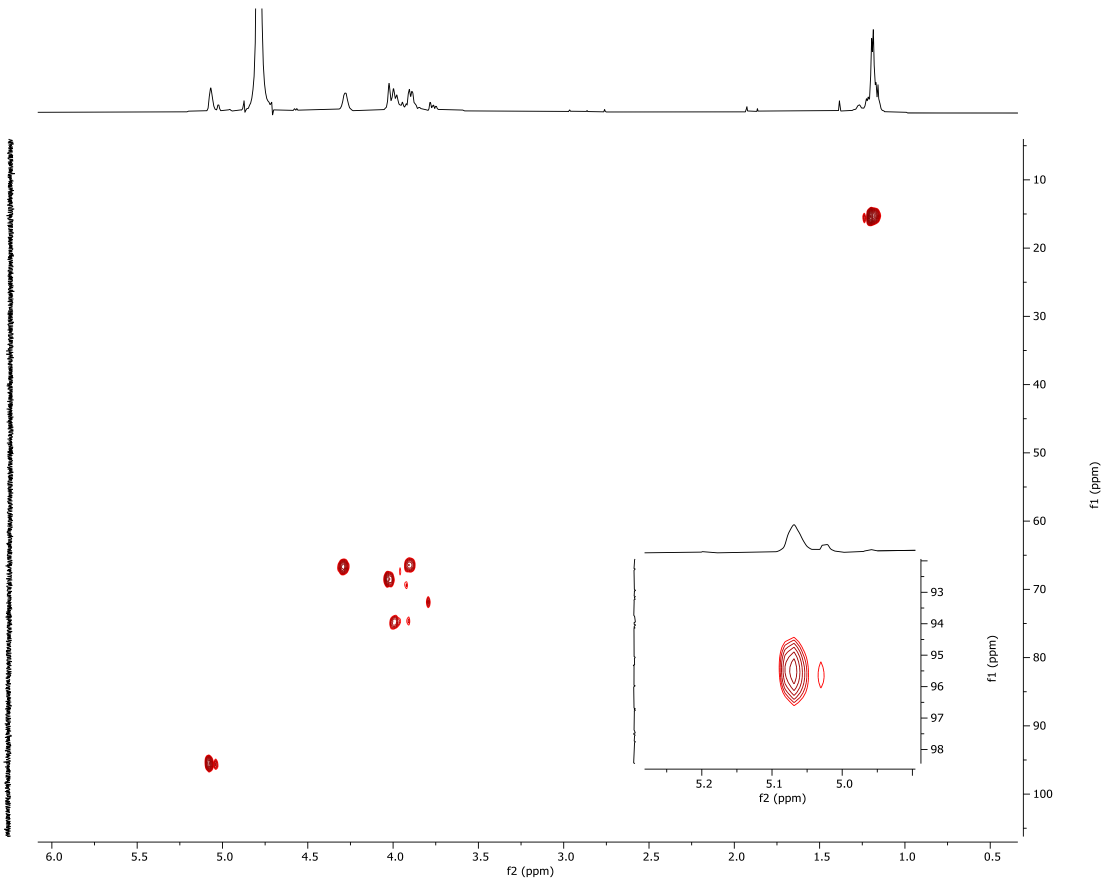

¹H-¹³C HSQC NMR. Inset is of zoomed anomeric region.

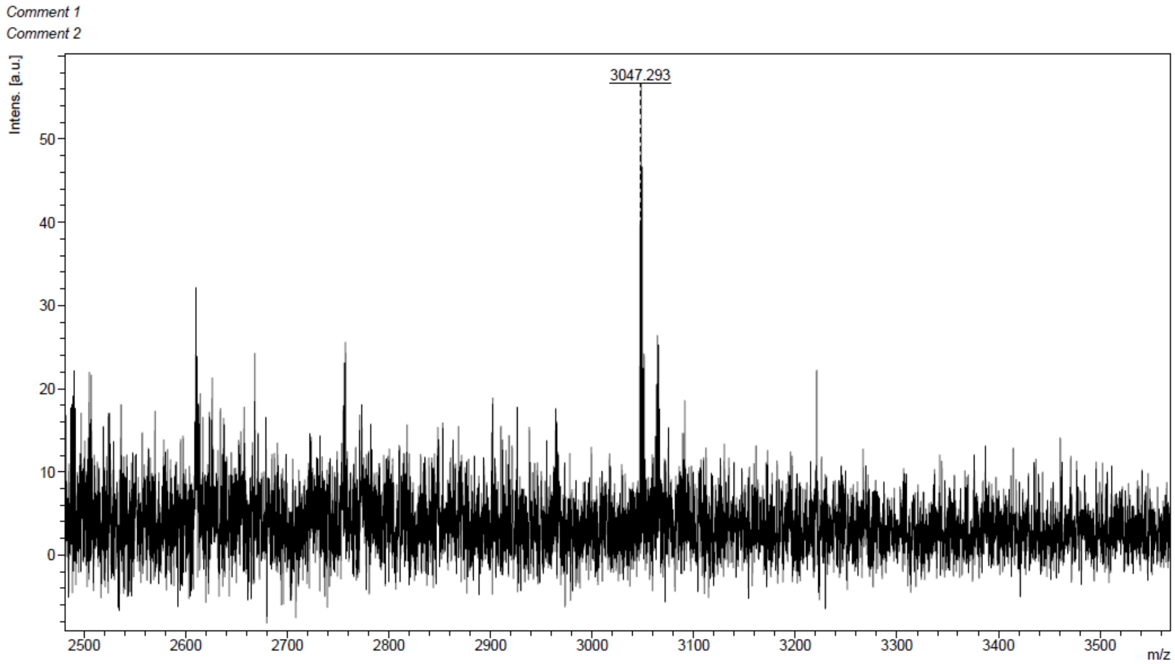

MALDI-TOF MS. Chemical formula, $[C_{125}H_{213}NNaO_{81}]^+$, exact mass: 3047.2471.

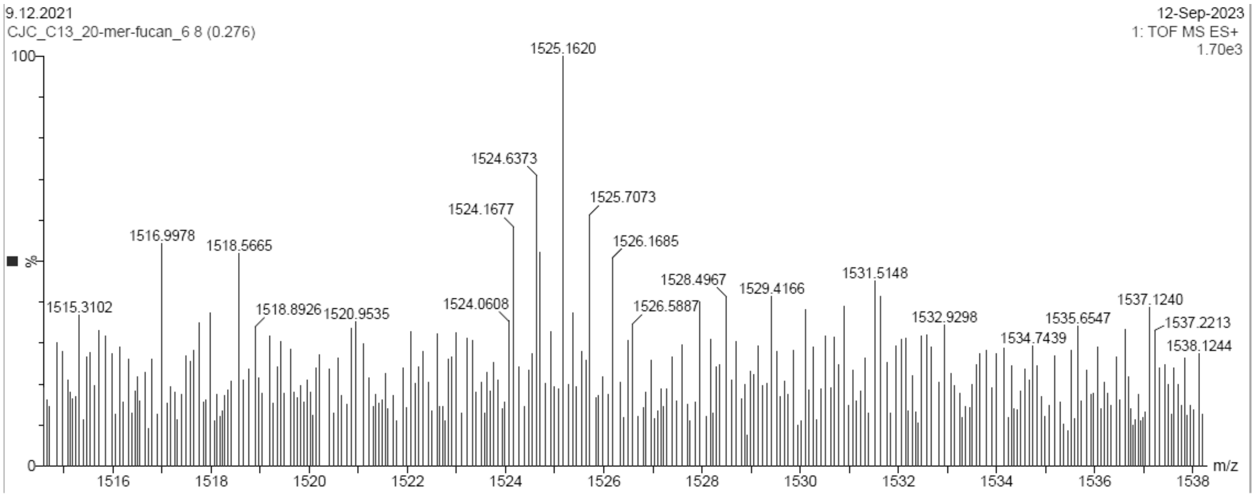

Q-TOF MS

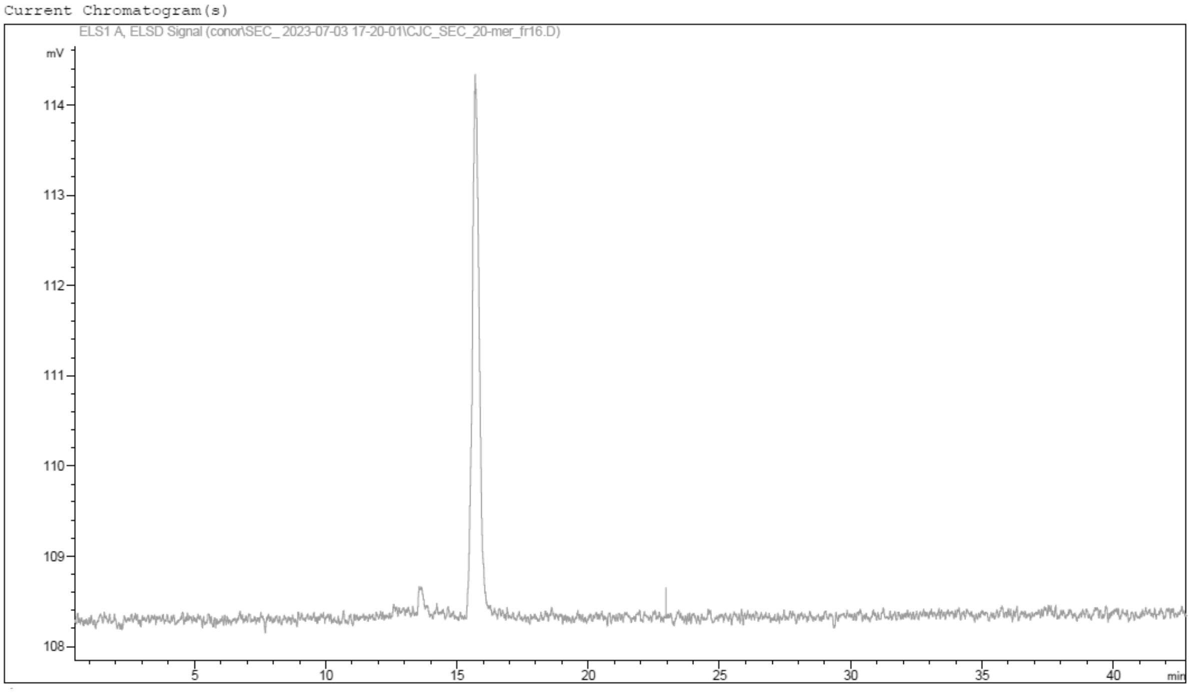

SEC (ELSD Trace)

5-Aminopentyl 3,4-di-*O*-sulfonate-α-L-fucopyranosyl-(1→3)-4-*O*-sulfonate-α-L-fucopyranosyl-(1→3)-4-*O*-sulfonate-α-L-fucopyranosyl-(1→3)-4-*O*-sulfonate-α-L-fucopyranoside (13)

1

L1

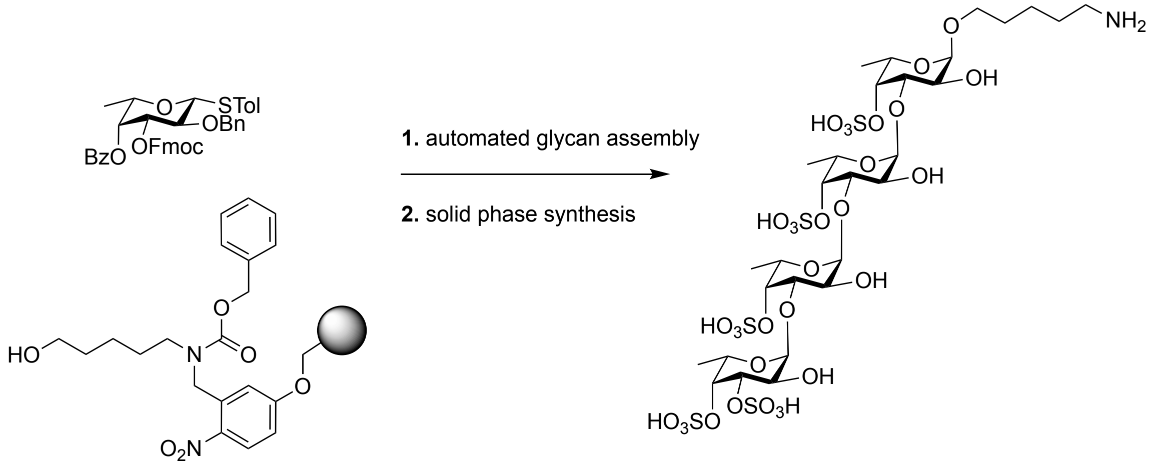

		Modules	Notes
**1. AGA**	**1**	a, b1, c, d1	L1<br>x4
	**Step**	**Module**	**Notes**
**2. Solid-phase synthesis**	Methanolysis<br>Sulfation<br>photocleavage	g<br>h1<br>i	
	**Step**	**Module**	**Notes**
**3. Solution-phase**	Hydrogenolysis<br>Purification	j2<br>Method 4	

The desired fractions were then collected and lyophilized to yield 2.1 mg (27%). **^{1}H NMR** (700 MHz, D_2O) δ 5.02 (d, *J* = 4.0 Hz, 1H, H-1, 1→3), 5.01 (d, *J* = 4.0 Hz, 1H, H-1, 1→3), 4.98 (d, *J* = 4.0 Hz, 1H), 4.79 (d, *J* = 3.9 Hz, 1H, H-1), 4.75 (d, *J* = 3.1 Hz, 1H, 4-*O*-sulfation), 4.66 – 4.62 (m, 3H, 4-*O*-sulfation), 4.50 – 4.45 (m, 1H, 3-*O*-sulfation), 4.40 (q, *J* = 6.6 Hz, 1H, H-5), 4.36 (q, *J* = 6.6 Hz, 1H, H-5), 4.32 (q, *J* = 6.7 Hz, 1H, H-5), 4.03 (q,

J = 6.7 Hz, 1H, H-5), 3.95 – 3.88 (m, 3H), 3.84 (dd, *J* = 10.4, 3.8 Hz, 1H, H-2), 3.79 – 3.74 (m, 3H, H-2), 3.60 – 3.53 (m, 1H, OC*H*$_2$CH$_2$CH$_2$CH$_2$CH$_2$NH$_2$), 3.46 – 3.40 (m, 1H, OC*H*$_2$CH$_2$CH$_2$CH$_2$CH$_2$NH$_2$), 2.88 (t, *J* = 7.6 Hz, 2H, OCH$_2$CH$_2$CH$_2$CH$_2$C*H*$_2$NH$_2$), 1.62 – 1.47 (m, 4H, OCH$_2$C*H*$_2$CH$_2$C*H*$_2$CH$_2$NH$_2$), 1.42 – 1.28 (m, 3H, OCH$_2$CH$_2$C*H*$_2$CH$_2$CH$_2$NH$_2$), 1.17 – 1.10 (m, 12H, H-6 CH$_3$). **^{13}C NMR‡** (176 MHz, D$_2$O) δ 98.5 (C-1, 1→3), 98.3 (C-1, amino pentyl), 98.0 (C-1, 1→3), 79.1 (4-*O*-sulfation), 79.1 (4-*O*-sulfation), 76.1, 75.3 (3-*O*-sulfation), 67.71 (C-2), 66.72 (C-2), 66.6 (C-5), 66.2 (C-2), 65.9 (C-2), 39.2, 28.0, 26.4, 22.4, 15.6 (CH$_3$). **HRMS** QTOF-MS: calcd. $C_{29}H_{50}NNaO_{32}S_5$ for $[M+Na - 3H]^{2-}$ 553.5414, found 553.5405.

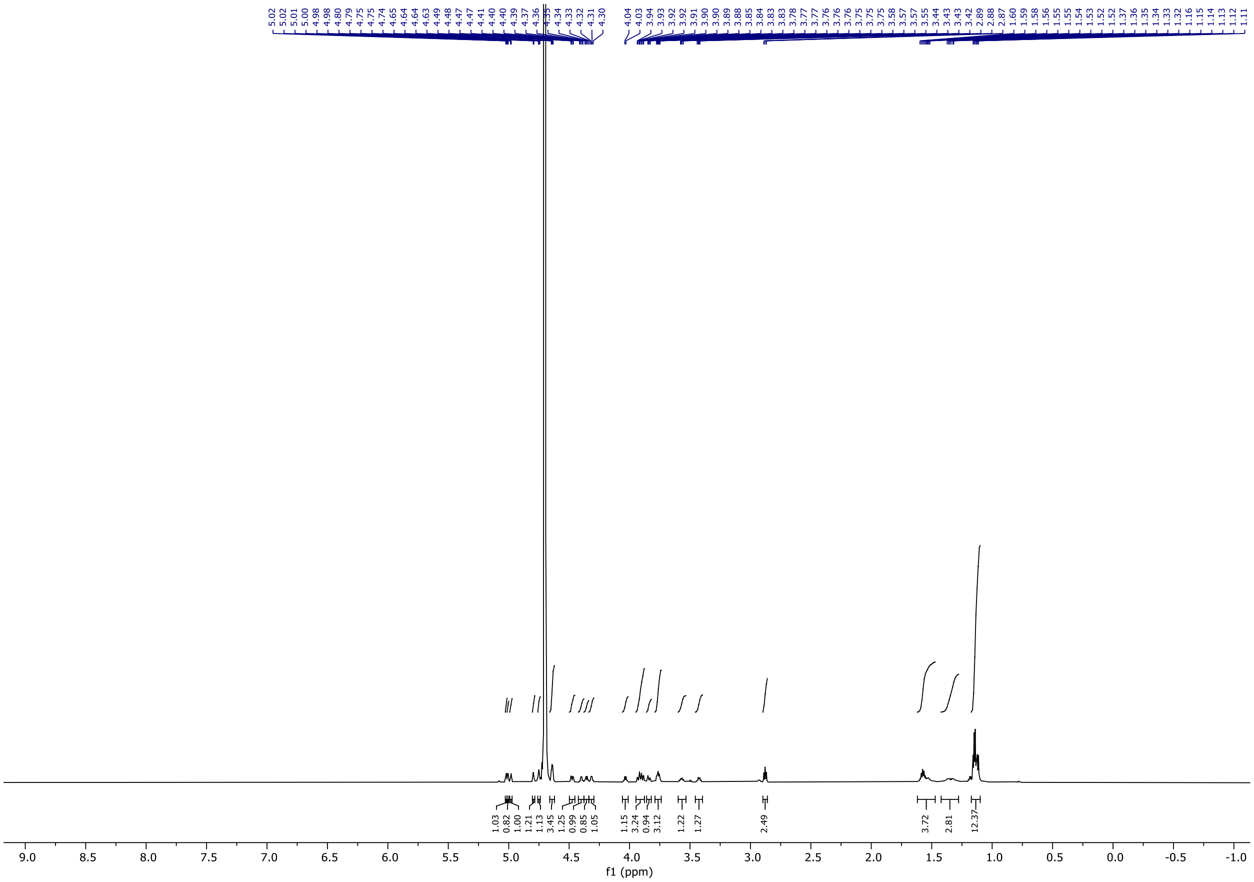

^{1}H NMR

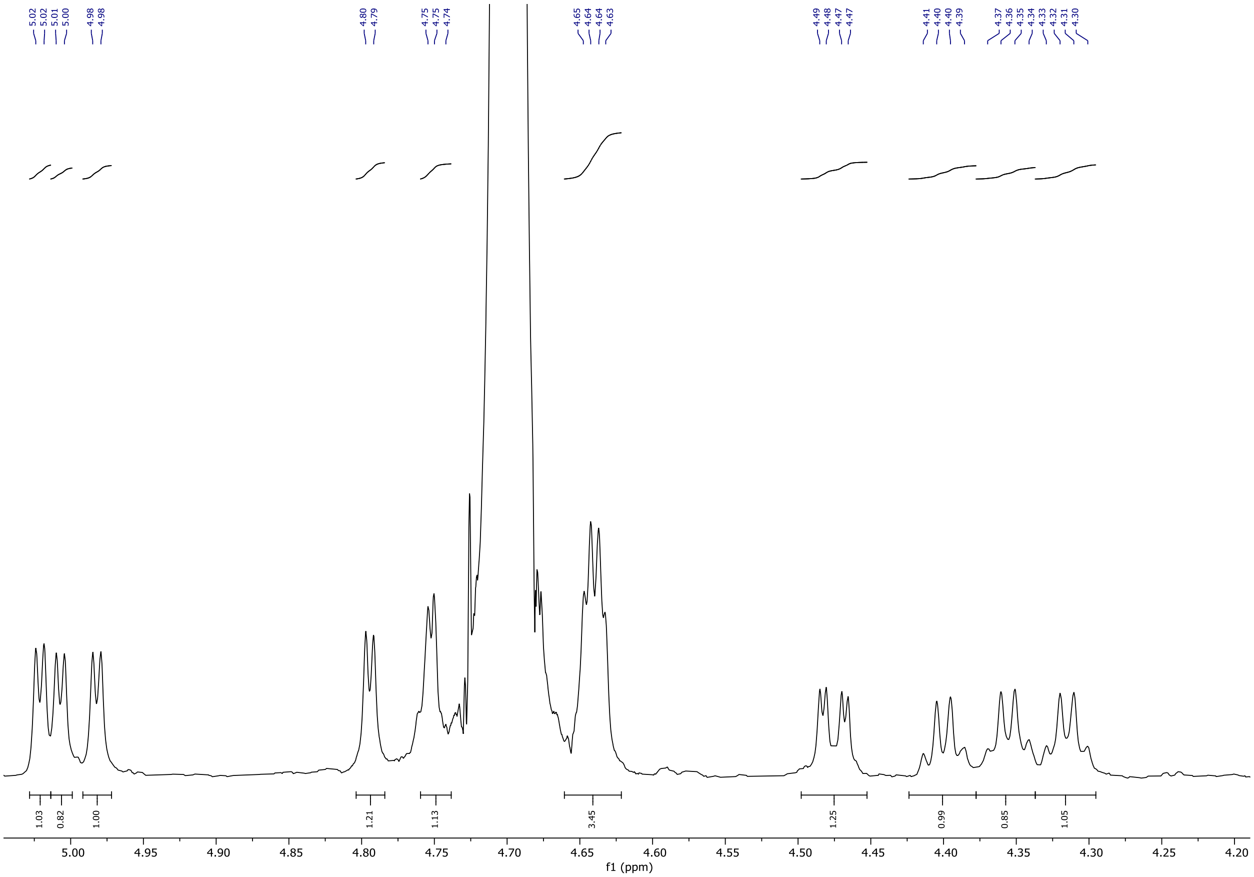

^{1}H NMR Zoomed

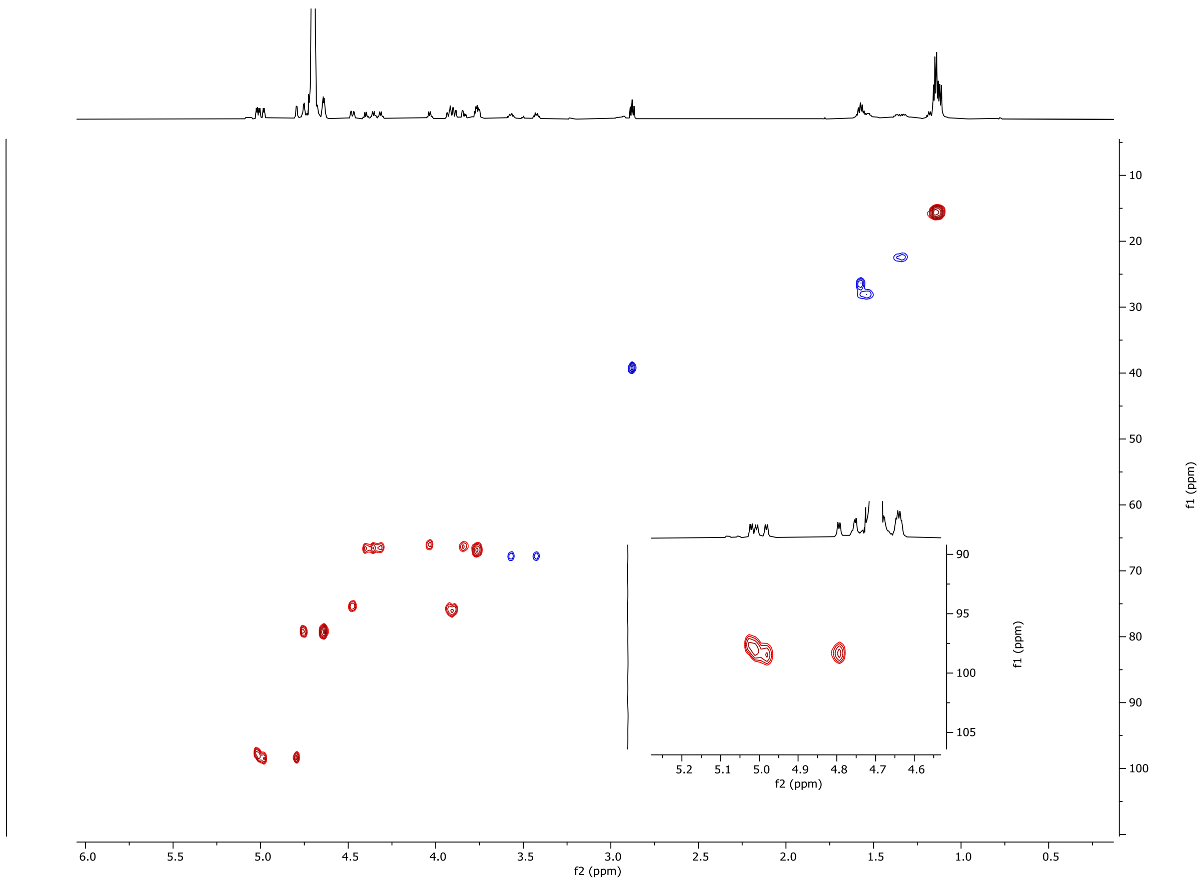

^{1}H-^{13}C HSQC NMR. Inset is of zoomed anomeric region.

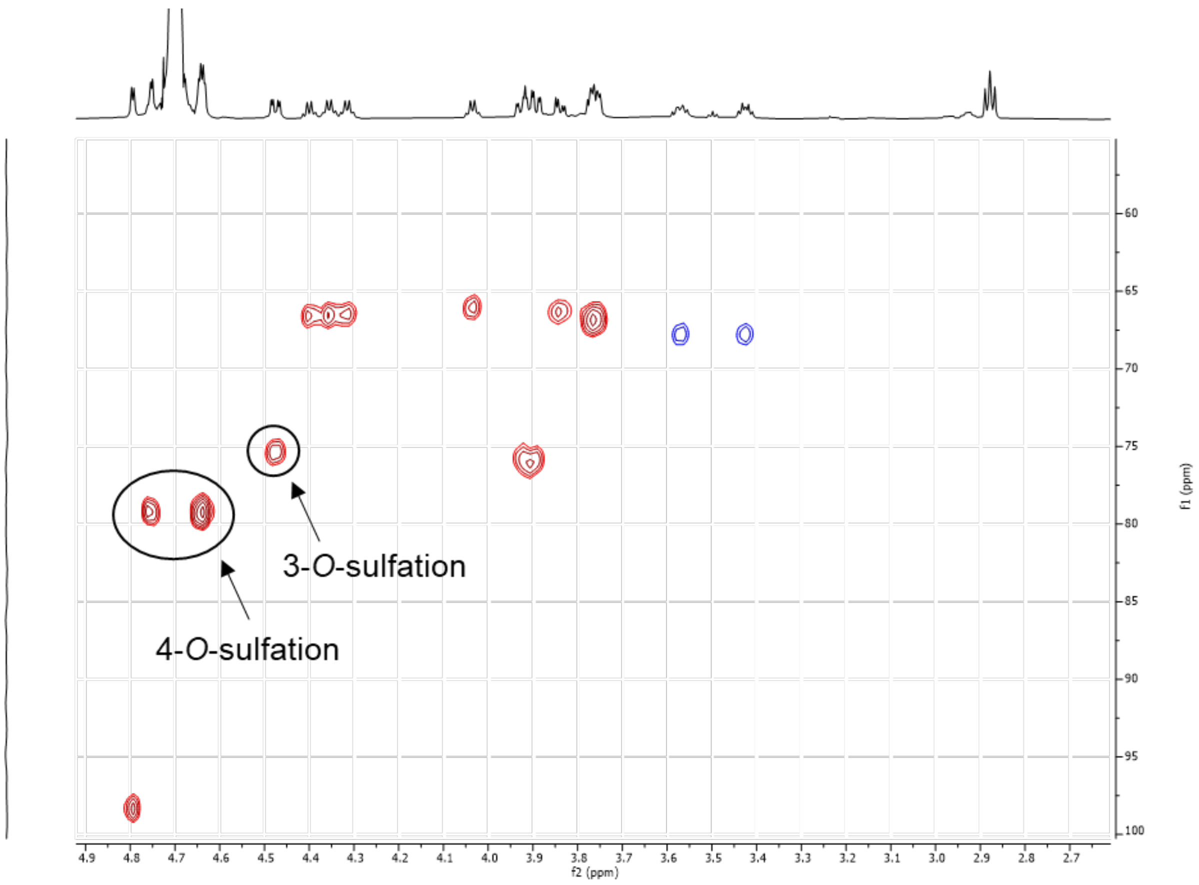

^{1}H-^{13}C **HSQC of tetrasaccharide 13 with sites of sulfation labelled.**

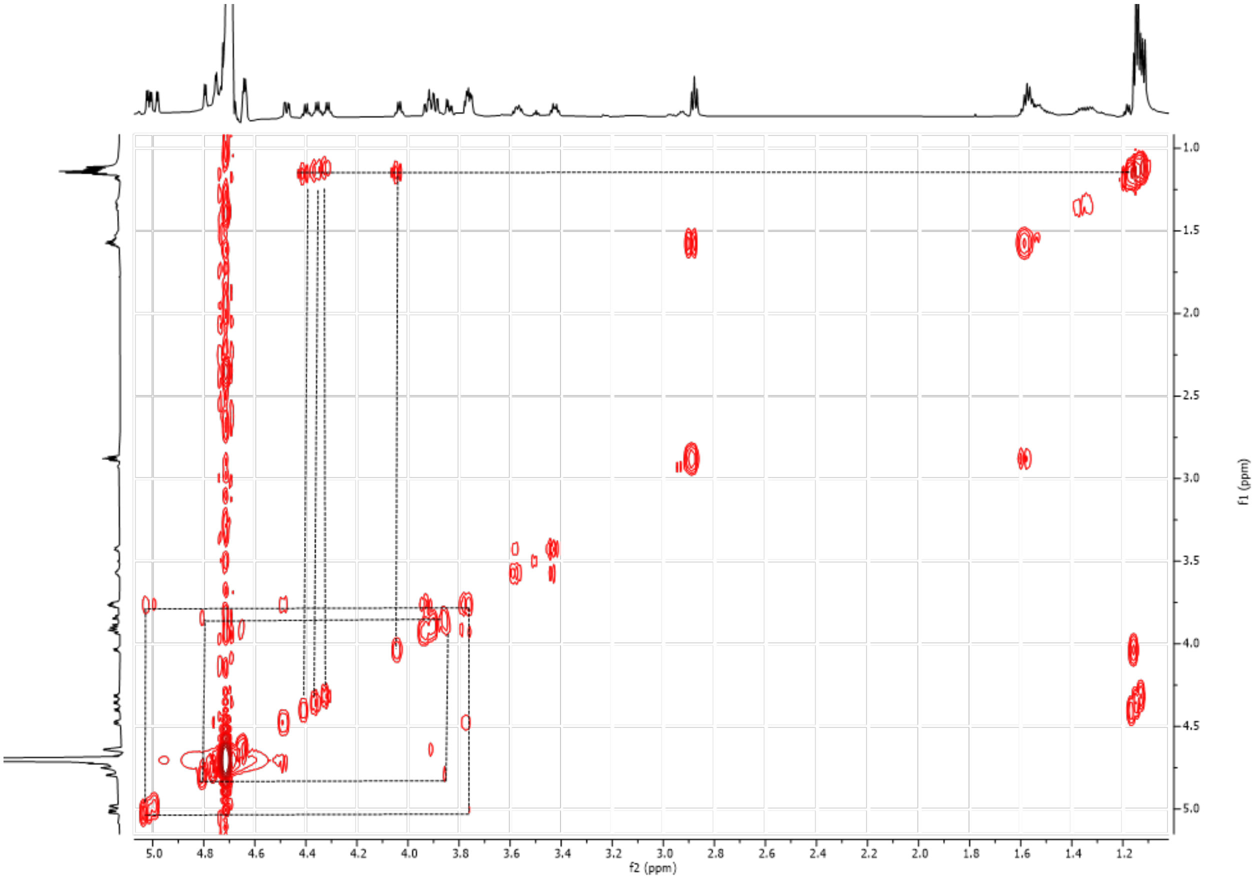

^1H-^1H COSY of tetrasaccharide 13 with labels showing connectivity between H-1 and H-2 and H-6 and H-5.

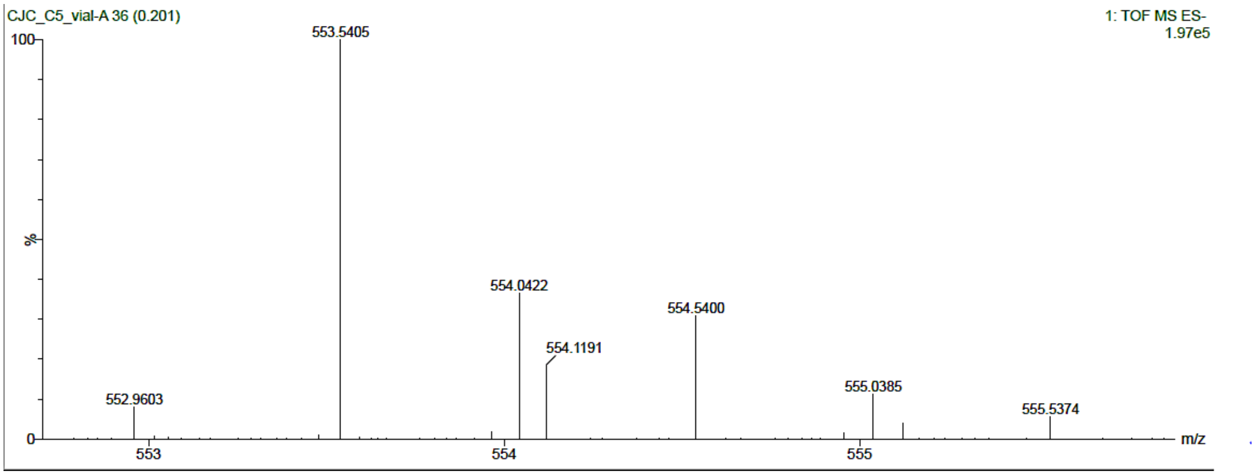

CJC_C5_vial-A 36 (0.201)
1: TOF MS ES-
1.97e5
100
%
0
553.5405
554.0422
554.5400
554.1191
552.9603
555.0385
555.5374
553
554
555
m/z

Q-TOF MS

5-Aminopentyl 3,4-di-*O*-sulfonate-α-L-fucopyranosyl-(1→3)-4-*O*-sulfonate-α-L-fucopyranosyl-(1→3)-4-*O*-sulfonate-α-L-fucopyranosyl-(1→3)-4-*O*-sulfonate-α-L-fucopyranosyl-(1→3)-4-*O*-sulfonate-α-L-fucopyranosyl-(1→3)-4-*O*-sulfonate-α-L-fucopyranosyl-(1→3)--4-*O*-sulfonate-α-L-fucopyranosyl-(1→3)-4-*O*-sulfonate-α-L-fucopyranosyl-(1→3)-4-*O*-sulfonate-α-L-fucopyranosyl-(1→3)-4-*O*-sulfonate-α-L-fucopyranoside (14)

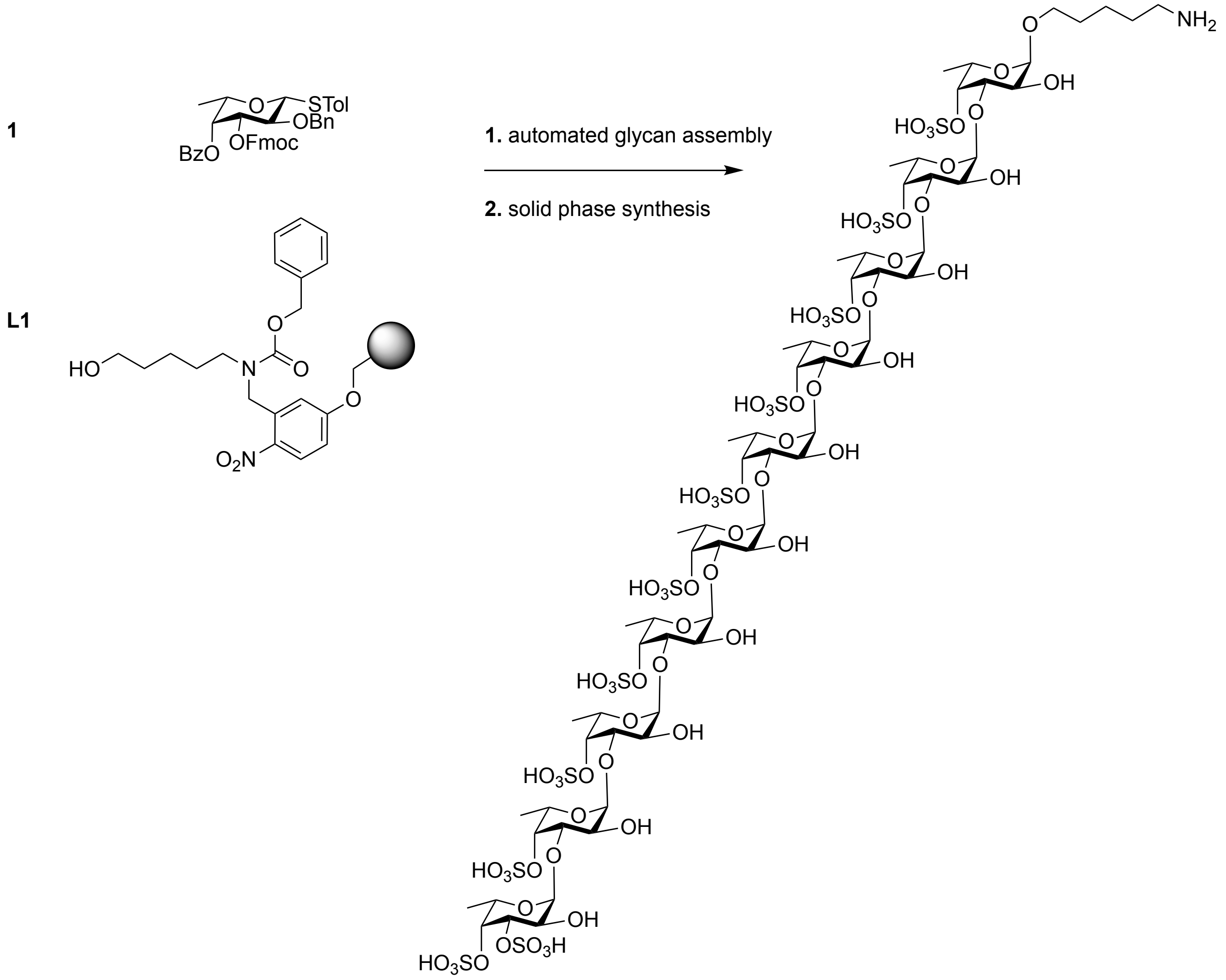

		Modules	Notes
**1. AGA**	**1**	a, b1, c, d1	L1<br>x10
	**Step**	**Module**	**Notes**
**2. Solid-phase synthesis**	Methanolysis<br>Sulfation<br>photocleavage	g<br>h2<br>i	48h<br>32h
	**Step**	**Module**	**Notes**
**3. Solution-phase**	Hydrogenolysis<br>Purification	j2<br>Method 4 then 7	

The desired fractions were then collected and lyophilized to yield 1.8 mg (5%). **^{1}H NMR** (400 MHz, D_2O) δ 5.06 (s, 9H, H-1, 1→3), 4.87 (s, 1H, H-1, amino pentyl), 4.72 (s, 17H, 4-*O*-sulfation), 4.66 (s, 1H, 4-*O*-sulfation), 4.59 – 4.53 (m, 2H, 3-*O*- and 4-*O*-sulfation), 4.48 – 4.42 (m, 12H, H-5), 4.40 – 4.39 (m, 2H, H-5), 4.12 (d, *J* = 6.7 Hz, 1H, H-5), 4.03 – 3.91 (m, 9H, H-2), 3.88 – 3.81 (m, 8H, H-2), 3.75 – 3.61 (m, 2H, $OCH_2CH_2CH_2CH_2CH_2NH_2$), 3.54 – 3.47 (m, 1H, $OCH_2CH_2CH_2CH_2CH_2NH_2$), 2.96 (t, *J* = 7.5 Hz, 3H, $OCH_2CH_2CH_2CH_2CH_2NH_2$), 1.67 – 1.61 (m, 5H, $OCH_2CH_2CH_2CH_2CH_2NH_2$), 1.48 – 1.34 (m, 1H, $OCH_2CH_2CH_2CH_2CH_2NH_2$), 1.22 (d, *J* = 6.8 Hz, 31H, CH_3). **^{13}C NMR‡** (176 MHz, D_2O) δ 98.3 (C-1, 1→3), 98.3 (C-1), 80.7, 79.5 (4-*O*-sulfation), 79.3 (4-*O*-sulfation), 76.2, 75.6, 75.3 (3-*O*-sulfation), 67.7, 67.0 (C-2), 66.6, 66.4 (C-5), 66.1, 39.1, 28.0, 26.4, 22.2, 15.4 (CH_3). **HRMS** QTOF-MS: calcd. $C_{65}H_{103}NNa_6O_{74}S_{11}$ for $[M+6Na - 10H]^{4-}$ 642.7666, found 642.7661.

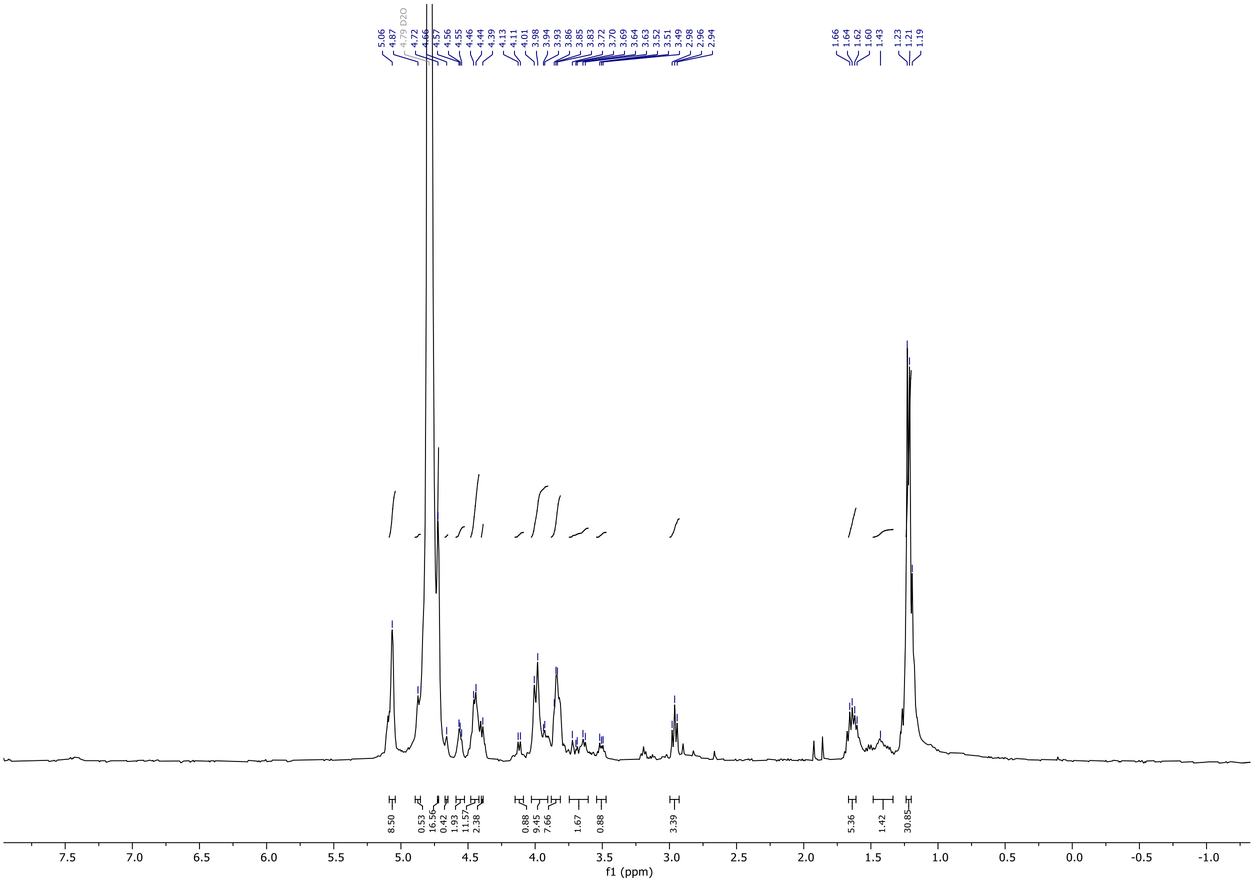

^{1}H NMR

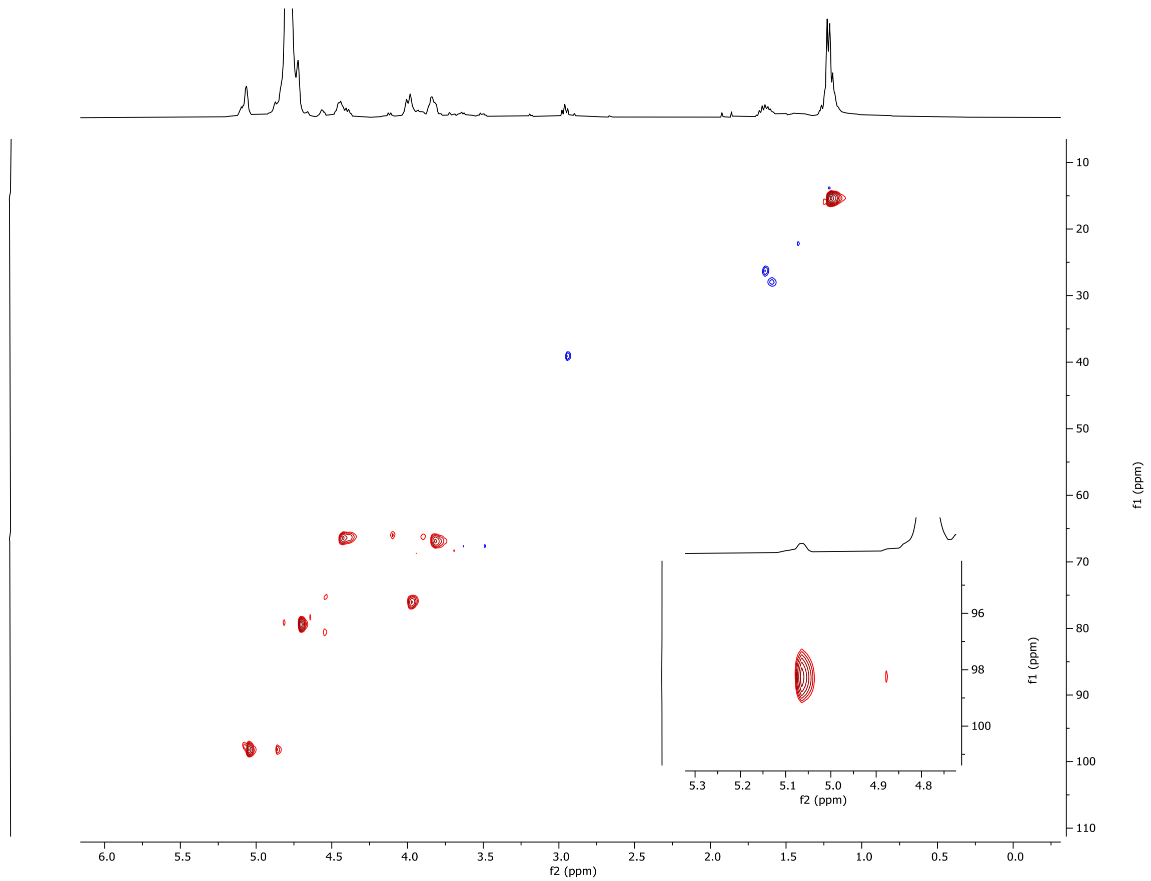

^1H-^{13}C HSQC NMR. Inset is of zoomed anomeric region.

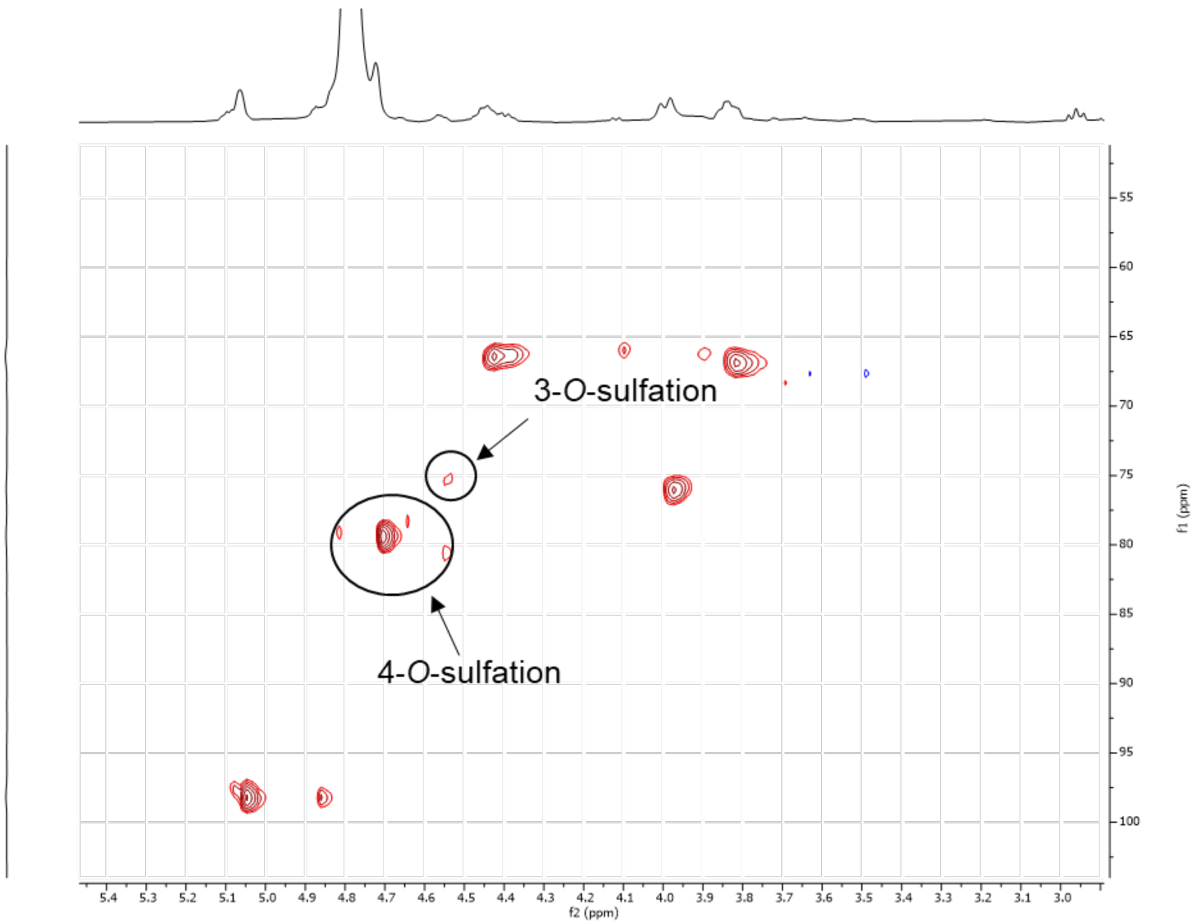

^{1}H-^{13}C HSQC of decasaccharide 14 with sites of sulfation labelled

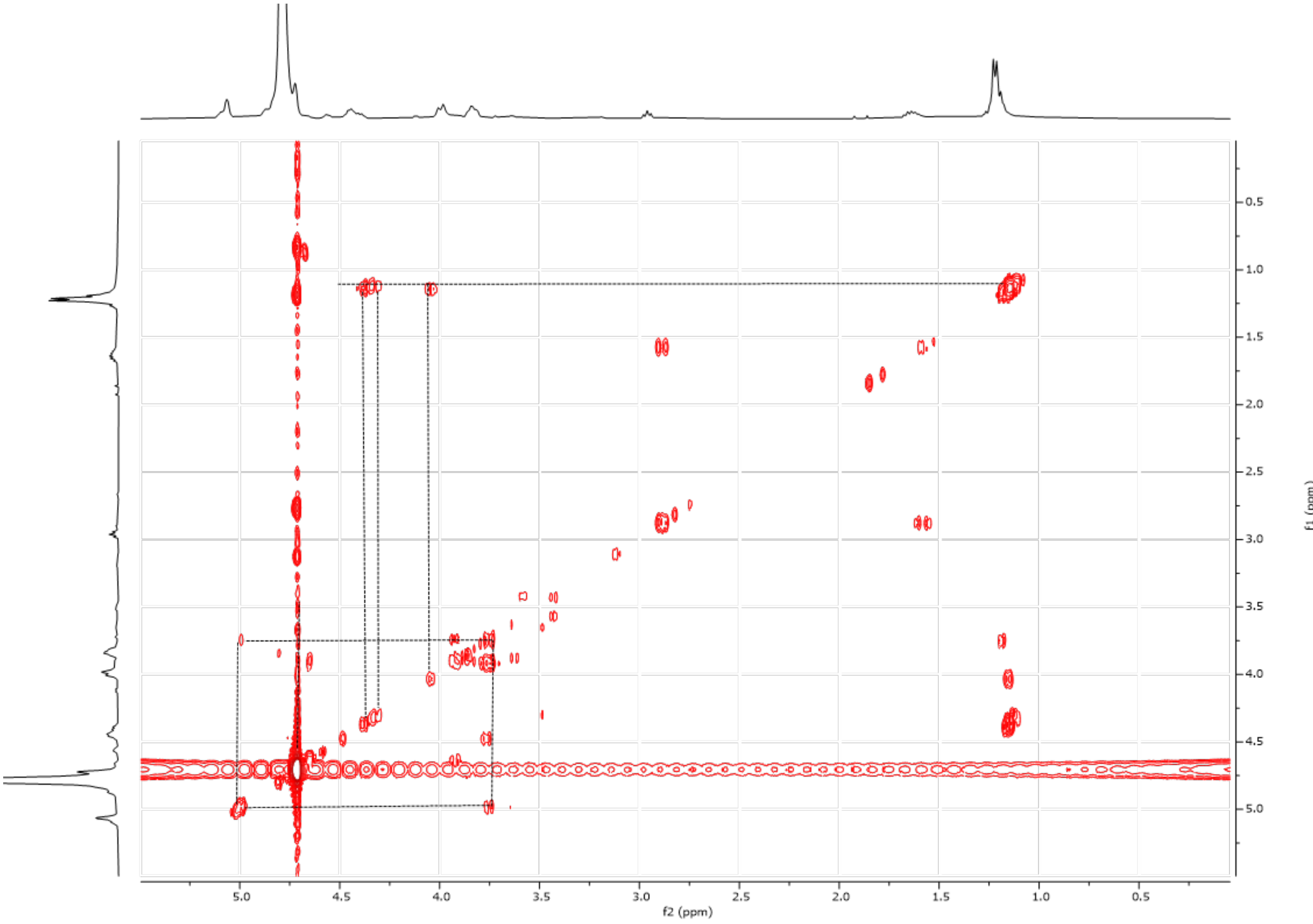

^{1}H-^{1}H COSY of decasaccharide 14 with labels showing connectivity between H-1 and H-2 and H-6 and H-5.

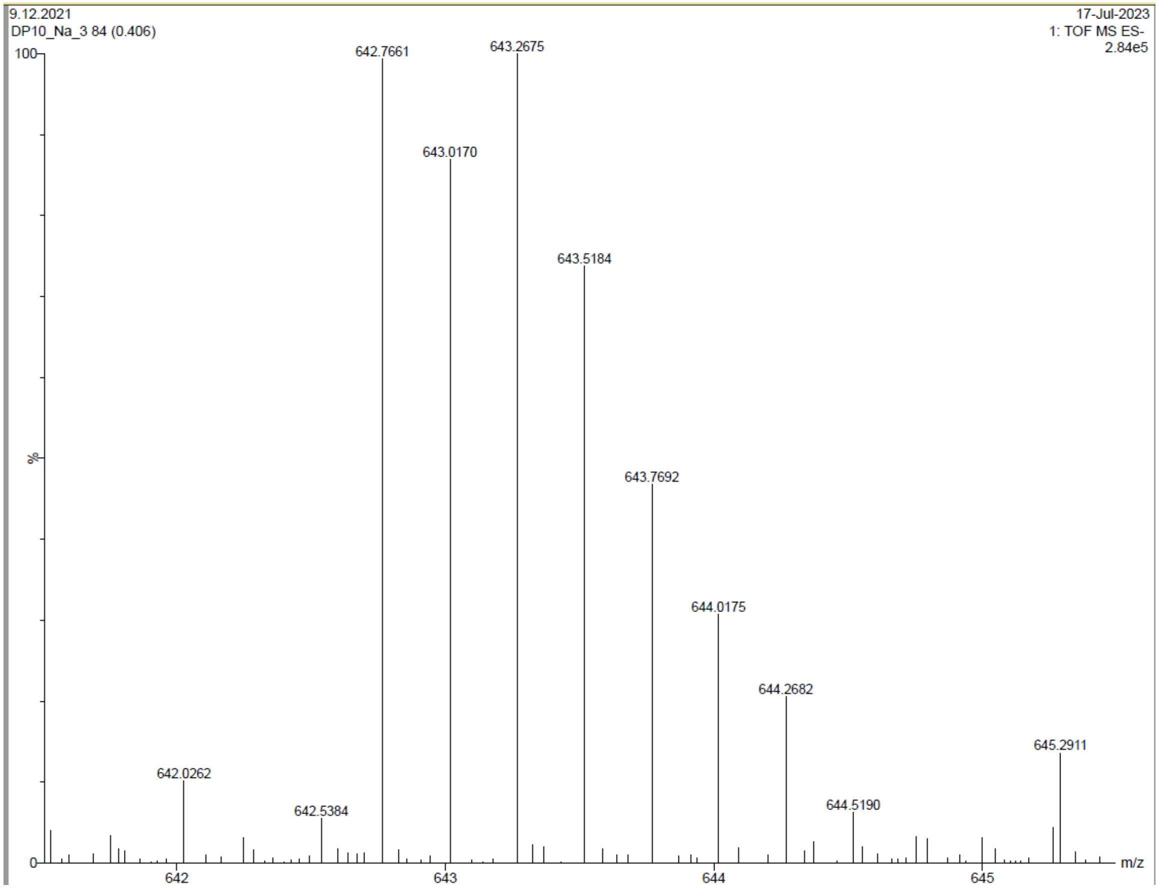

9.12.2021
DP10_Na_3 84 (0.406)
17-Jul-2023
1: TOF MS ES-
2.84e5
100
%
0
642.7661
643.2675
643.0170
643.5184
643.7692
644.0175
644.2682
645.2911
642.0262
642.5384
644.5190
642
643
644
645
m/z

Q-TOF MS

5-aminopentyl α-L-fucopyranosyl-(1→3)-α-L-fucopyranosyl-(1→4)-α-L-fucopyranosyl-(1→3)-α-L-fucopyranoside (15)

1

2

L1

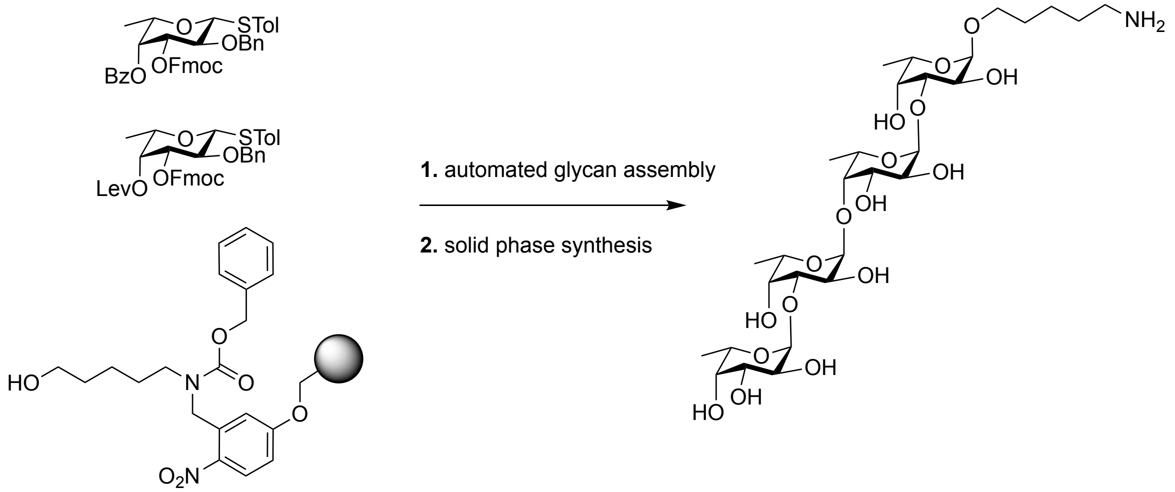

		Modules	Notes
**1. AGA**			L1
	**1**	a, b1, c, d2	x1
	**2**	a, b1, d2, c, e	x1
	**1**	a, b1, c, d2	x1
	**2**	a, b1	x1
	**Step**	**Module**	**Notes**
**2. Solid-phase synthesis**	Methanolysis	g	
	photocleavage	i	
	**Step**	**Module**	**Notes**
**3. Solution-phase**	Hydrogenolysis	j1	
	Purification	Method 2	

The desired fractions were then collected and lyophilized to yield 0.5 mg (5%). **^{1}H NMR** (400 MHz, D_2O) δ 5.06 (d, *J* = 4.0 Hz, 1H, H-1, 1→4), 5.01 (d, *J* = 4.0 Hz, 1H, H-1, 1→3), 4.95 (d, *J* = 3.4 Hz, 1H, H-1, 1→3), 4.87 (d, *J* = 3.1 Hz, 2H, H-1, amino pentyl), 4.54 – 4.46 (m, 1H), 4.33 – 4.24 (m, 1H), 4.06 – 3.95 (m, 4H), 3.94 – 3.71 (m, 10H), 3.70 – 3.59 (m, 1H $OC\mathit{H_2}CH_2CH_2CH_2CH_2NH_2$), 3.54 – 3.44 (m, 1H, $OC\mathit{H_2}CH_2CH_2CH_2CH_2NH_2$), 3.00 – 2.91 (m, 2H, $OCH_2CH_2CH_2CH_2C\mathit{H_2}NH_2$), 1.71 – 1.57 (m, 4H, $OCH_2C\mathit{H_2}CH_2C\mathit{H_2}CH_2NH_2$), 1.47 – 1.35 (m, 1H, $OCH_2CH_2C\mathit{H_2}CH_2CH_2NH_2$), 1.26 (d, *J* = 6.7 Hz, 3H, CH_3), 1.19 (d, *J* = 6.7 Hz, 4H, CH_3), 1.17 – 1.13 (m, 7H, CH_3). **^{13}C NMR‡** (101 MHz, D_2O) δ 100.4 (C-1, 1→3), 98.3 (C-1, amino pentyl), 96.4 (C-1, 1→3), 95.6 (C-1, 1→4), 79.8, 75.2, 71.9, 69.3, 68.6, 68.1, 67.8, 67.8, 67.2, 66.9, 66.5, 66.2, 39.3, 28.2, 26.5, 22.4, 15.3 (CH_3), 15.3 (CH_3), 15.1 (CH_3). **HRMS** QTOF-MS: calcd. $C_{29}H_{54}O_{17}$ for $[M+H]^+$ 688.3386, found 688.3394.

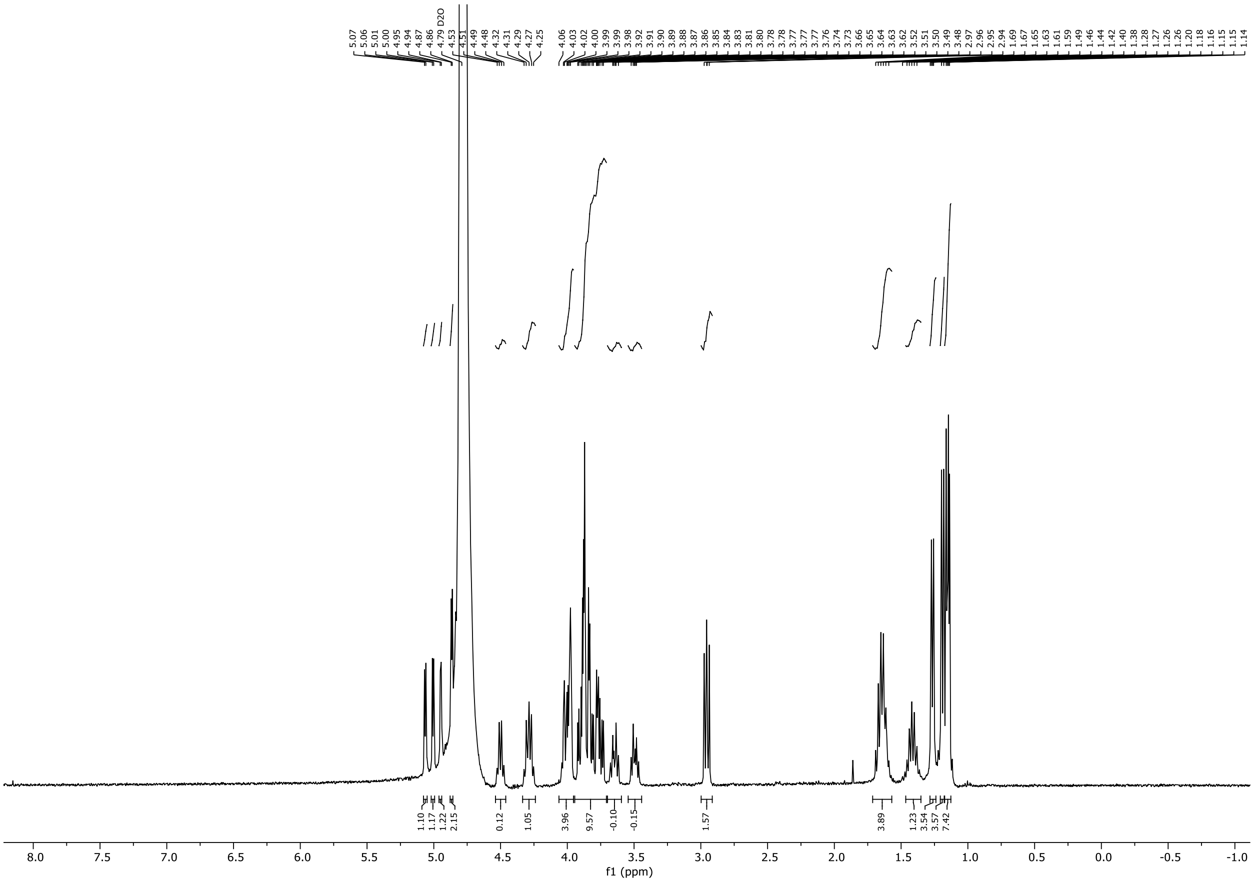

^{1}H NMR

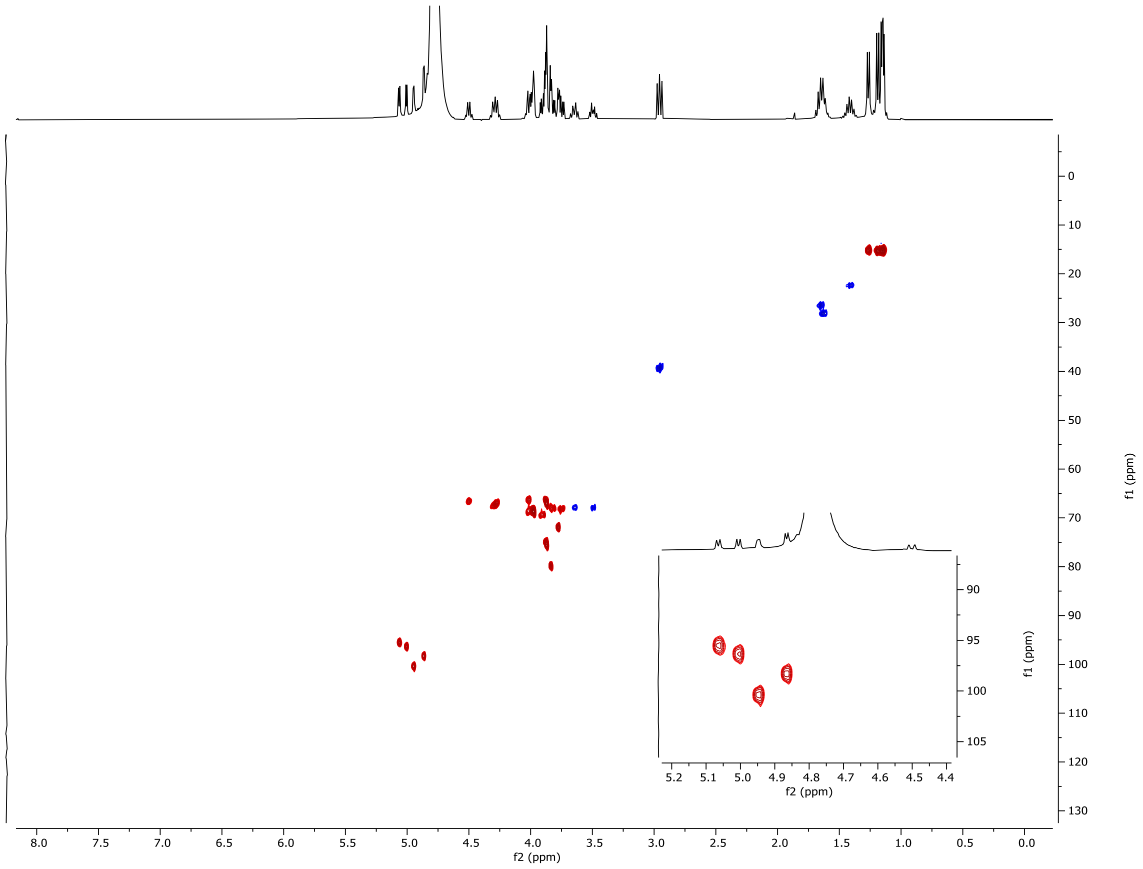

^{1}H-^{13}C HSQC. Inset is of zoomed anomeric region.

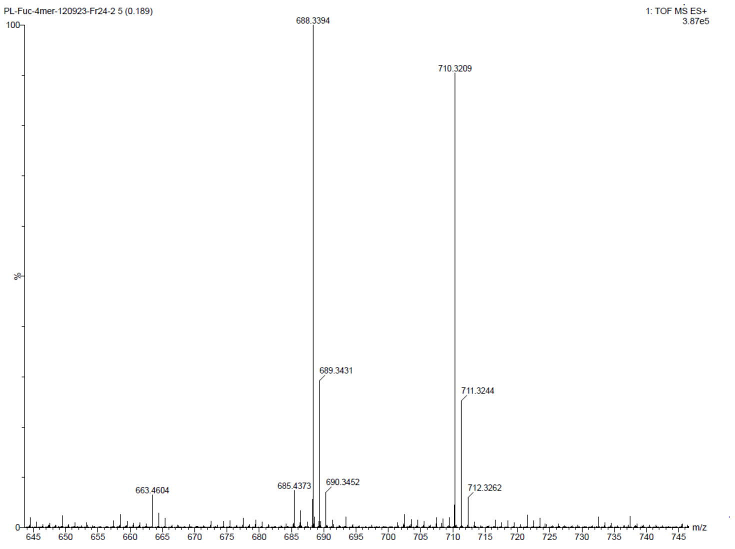

Q-TOF MS

5-aminopentyl α-L-fucopyranosyl-(1→3)-α-L-fucopyranosyl-(1→4)-α-L-fucopyranosyl-(1→3)-α-L-fucopyranosyl-(1→4)-α-L-fucopyranosyl-(1→3)-α-L-fucopyranoside (16)

1

2

L1

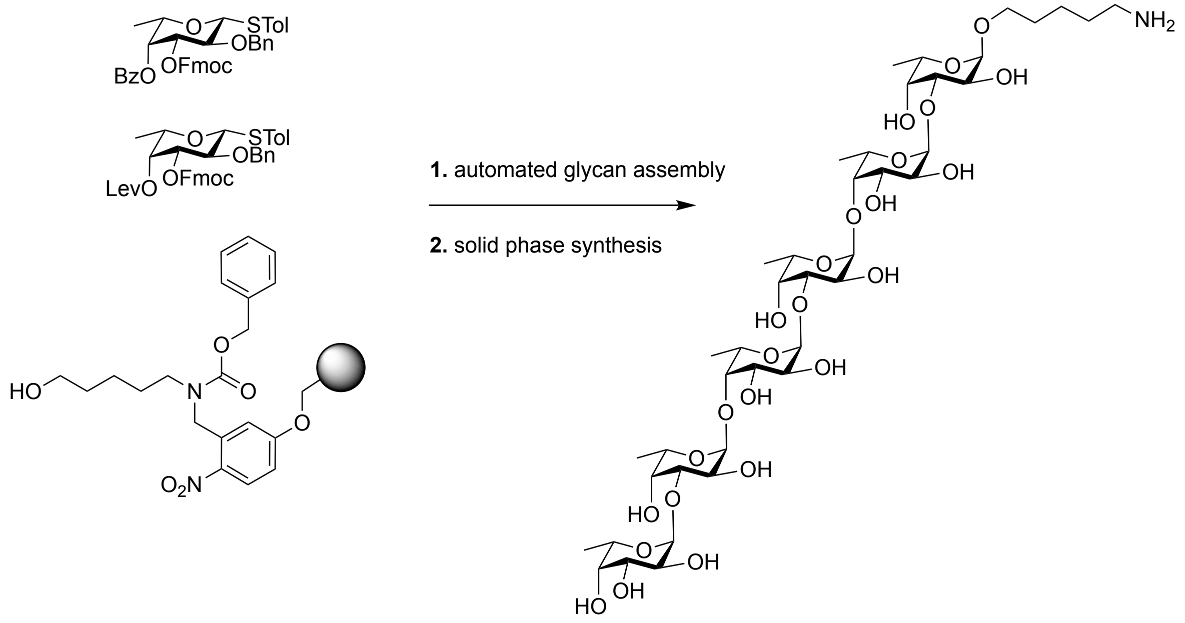

		Modules	Notes
1. AGA			L1
	**1**	a, b1, c, d2	x1
	**2**	a, b1, d2, c, e	x1
	**1**	a, b1, c, d2	x1
	**2**	a, b1, d2, c, e	x1
	**1**	a, b1, c, d2	x1
	**2**	a, b1	x1
	**Step**	**Module**	**Notes**
**2. Solid-phase synthesis**	Methanolysis	g	
	photocleavage	i	
	**Step**	**Module**	**Notes**
	Hydrogenolysis	j1	

3. Solution-phase	Purification	Method 2

The desired fractions were then collected and lyophilized to yield 0.6 mg (5%). **^{1}H NMR** (400 MHz, D_2O) δ 5.07 (ap t, *J* = 3.9 Hz, 2H, H-1, 1→4), 5.02 (d, *J* = 4.0 Hz, 1H, H-1, 1→3), 4.96 (ap t, *J* = 3.2 Hz, 2H, H-1, 1→3), 4.88 (d, *J* = 3.2 Hz, 1H, H-1, amino pentyl), 4.55 – 4.48 (m, 2H), 4.39 – 4.26 (m, 3H), 4.07 – 3.96 (m, 7H), 3.94 – 3.92 (m, 1H), 3.92 – 3.87 (m, 7H), 3.87 – 3.80 (m, 5H), 3.80 – 3.74 (m, 2H), 3.71 – 3.57 (m, 1H, $OC\mathit{H}_2CH_2CH_2CH_2CH_2NH_2$), 3.56 – 3.46 (m, 1H, $OC\mathit{H}_2CH_2CH_2CH_2CH_2NH_2$), 2.97 (t, *J* = 6.9 Hz, 2H, $OCH_2CH_2CH_2CH_2C\mathit{H}_2NH_2$), 1.73 – 1.57 (m, 4H, $OCH_2\mathit{CH}_2CH_2\mathit{CH}_2CH_2NH_2$), 1.50 – 1.37 (m, 2H, $OCH_2CH_2C\mathit{H}_2CH_2CH_2NH_2$), 1.28 (dd, *J* = 6.7, 2.6 Hz, 6H, CH_3), 1.20 (d, *J* = 6.7 Hz, 4H, CH_3), 1.17 (dd, *J* = 6.6, 3.8 Hz, 9H, CH_3). **^{13}C NMR‡** (101 MHz, D_2O) δ 100.4 (C-1, 1→3), 98.5 (C-1, amino pentyl), 96.2 (C-1, 1→3), 96.2 (C-1, 1→4), 80.1, 75.6, 72.0, 69.1, 68.8, 68.1, 67.8, 67.8, 67.5, 67.2, 66.5, 39.4, 27.2, 22.4, 15.6 (CH_3), 15.3 (CH_3), 15.3 (CH_3). **HRMS** QTOF-MS: calcd. $C_{41}H_{74}NO_{25}$ for $[M+H]^+$ 980.4550, found 980.4267.

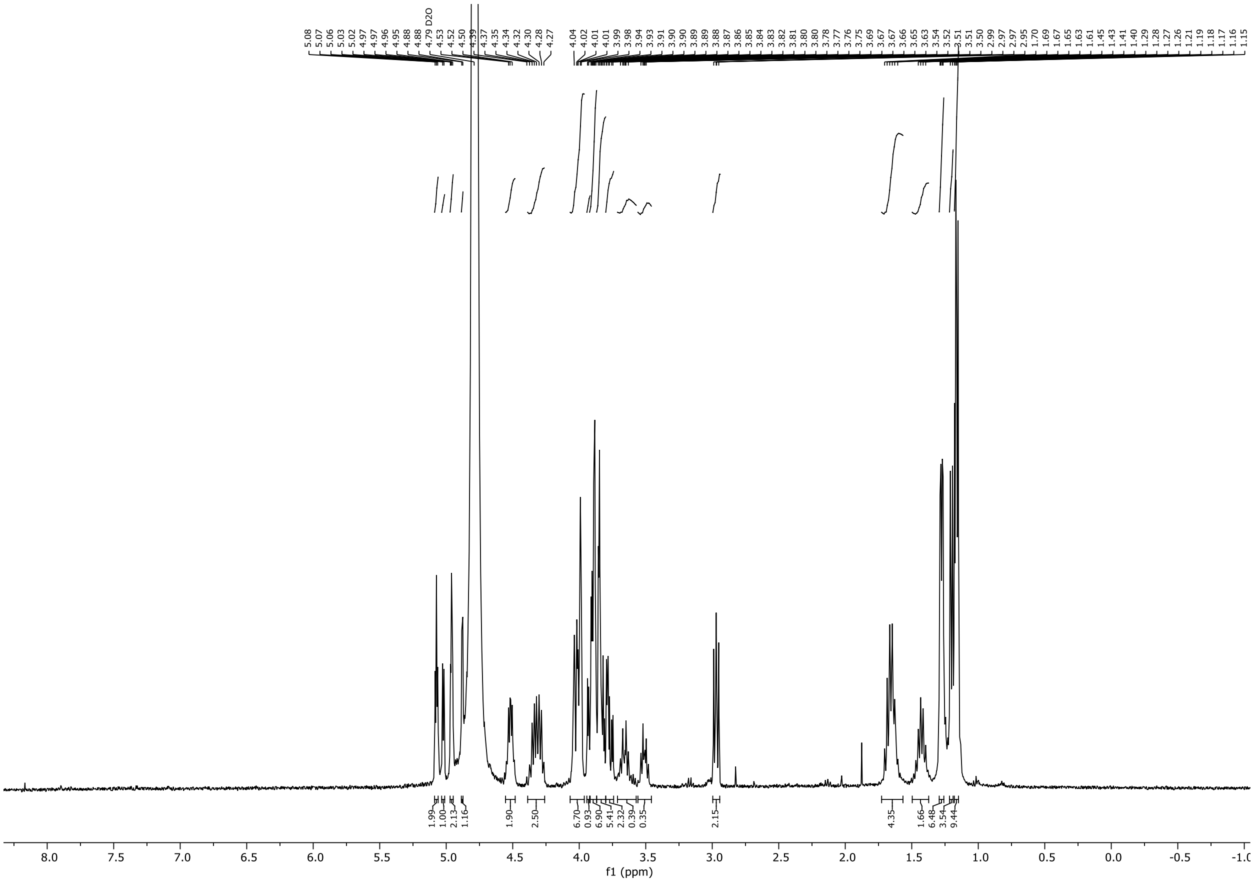

^{1}H NMR

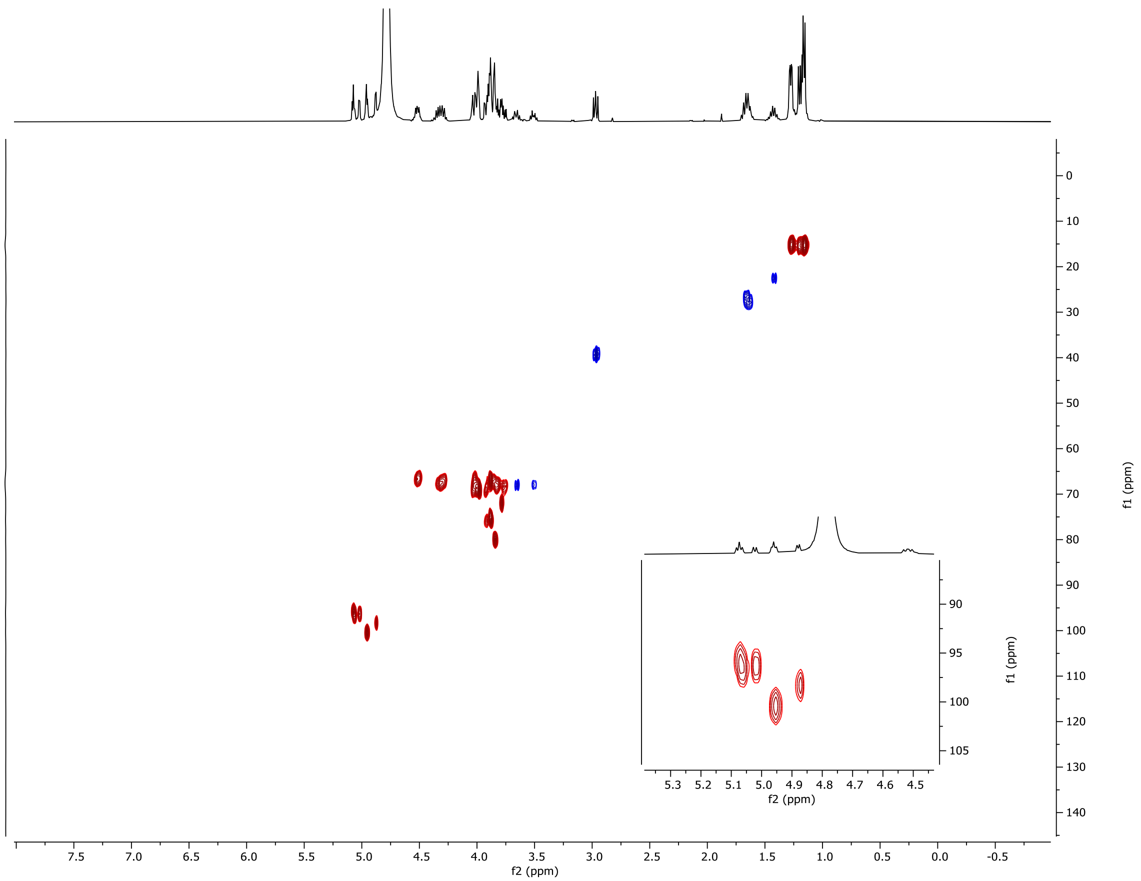

^{1}H-^{13}C HSQC NMR. Inset is of zoomed anomeric region.

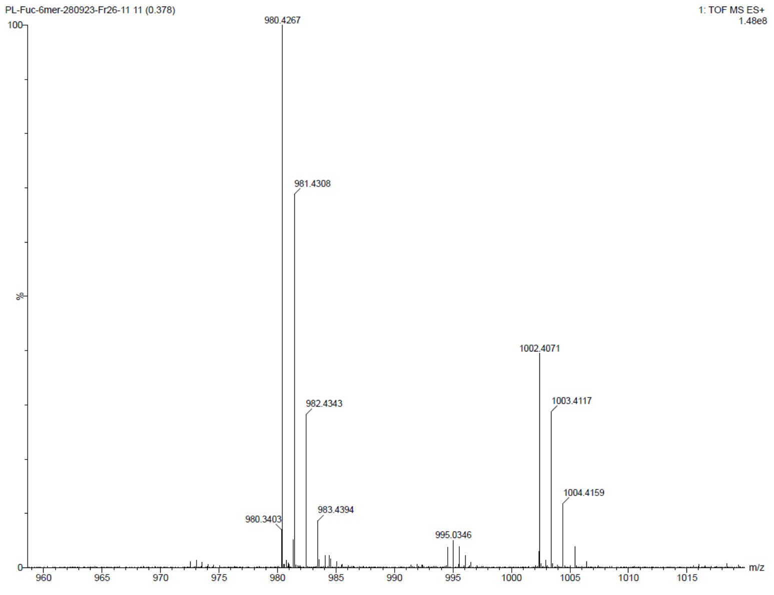

Q-TOF MS

5-aminopentyl 3,4-di-*O*-sulfonate-α-L-fucopyranosyl-(1→3)-4-*O*-sulfonate-α-L-fucopyranosyl-(1→4)-3-*O*-sulfonate-α-L-fucopyranosyl-(1→3)-4-*O*-sulfonate-α-L-fucopyranosyl-(1→4)-3-*O*-sulfonate-α-L-fucopyranosyl-(1→3)-4-*O*-sulfonate-α-L-fucopyranoside (17)

1

2

L1

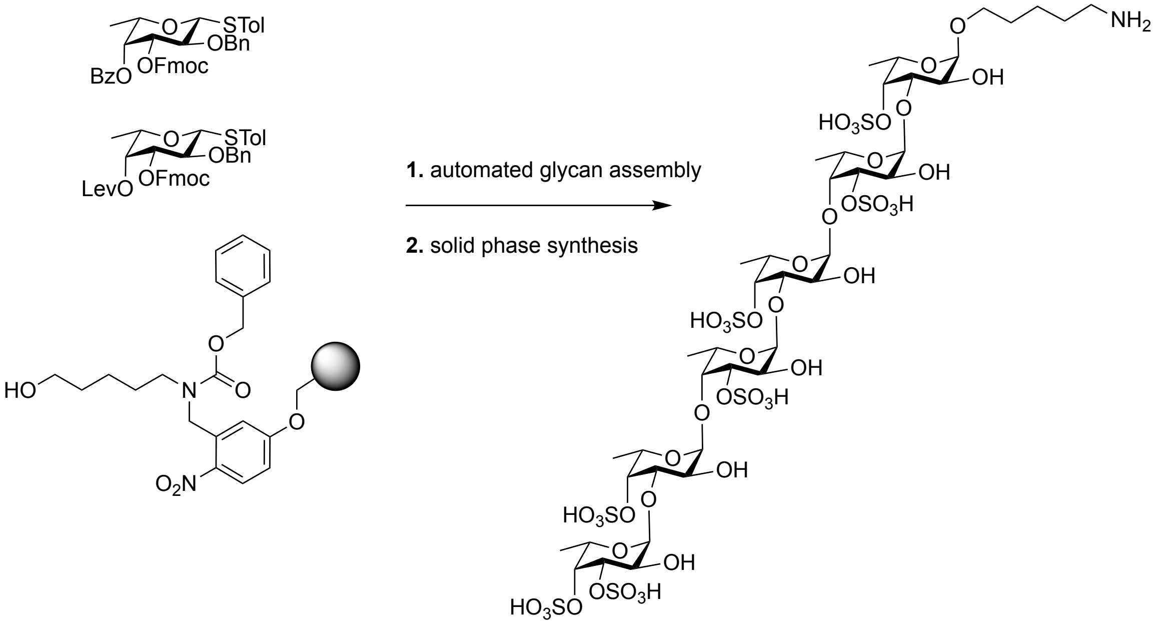

		Modules	Notes
			L1
**1. AGA**	**1**	a, b1, c, d2	x1
	**2**	a, b1, d2, c, e	x1
	**1**	a, b1, c, d2	x1
	**2**	a, b1, d2, c, e	x1
	**1**	a, b1, c, d2	x1
	**2**	a, b1	x1
	**Step**	**Module**	**Notes**
**2. Solid-phase synthesis**	Methanolysis	g	
	Sulfation	h2	
	photocleavage	i	

	Step	Module	Notes
**3. Solution-phase**	Hydrogenolysis	J2	
	Purification	Method 9 and 11	

The desired fractions were then collected and lyophilized to yield 1 mg (5%). **^{1}H NMR** ^{1}H NMR (400 MHz, D_2O) δ 5.24 (s, 1H, 1→3), 5.19 (d, *J* = 4.4 Hz, 1H, 1→4), 5.16 (d, *J* = 3.5 Hz, 1H, 1→4), 5.07 (s, 2H, 1→3), 4.94 – 4.88 (m, 3H, H-1, amino pentyl, 4-*O*-sulfation), 4.72 – 4.66 (m, 2H, 3-*O*-sulfation), 4.66 – 4.59 (m, 2H, 3-*O*-sulfation), 4.51 – 4.40 (m, 4H), 4.23 (s, 2H), 4.20 – 4.14 (m, 2H), 4.12 – 3.89 (m, 12H), 3.73 – 3.67 (m, 2H), 3.57 (d, *J* = 7.3 Hz, 2H), 3.02 (t, *J* = 7.6 Hz, 2H, $OC\mathit{H_2}CH_2CH_2CH_2CH_2NH_2$), 1.76 – 1.64 (m, 6.9 Hz, 5H, $OCH_2\mathit{CH_2}CH_2\mathit{CH_2}CH_2NH_2$), 1.51 – 1.45 (m, 2H, $OCH_2CH_2C\mathit{H_2}CH_2CH_2NH_2$), 1.34 (d, *J* = 7.2 Hz, 7H, CH_3), 1.31 – 1.20 (m, 11H, CH_3). **^{13}C NMR‡** (151 MHz, D_2O) δ 100.4 (C-1, 1→3, *J* = 172 Hz), 98.8 (C-1, amino pentyl, *J* = 170 Hz), 97.5 (C-1, 1→3, *J* = 173 Hz), 96.2 (C-1, 1→4, *J* = 169 Hz,), 79.6 (4-*O*-sulfation), 78.8 (4-*O*-sulfation), 78.0, 76.8 (3-*O*-sulfation), 76.4 (3-*O*-sulfation), 76.0, 69.1, 68.3, 68.3, 67.2, 67.0, 67.0, 66.5, 39.8, 28.5, 26.9, 22.9, 15.9 (CH_3), 15.8 (CH_3). **HRMS** QTOF-MS: calcd. $C_{41}H_{67}NNa_4O_{46}S_7$ for $[M+4Na - 6H]^{2-}$ 812.5290, found 812.5275.

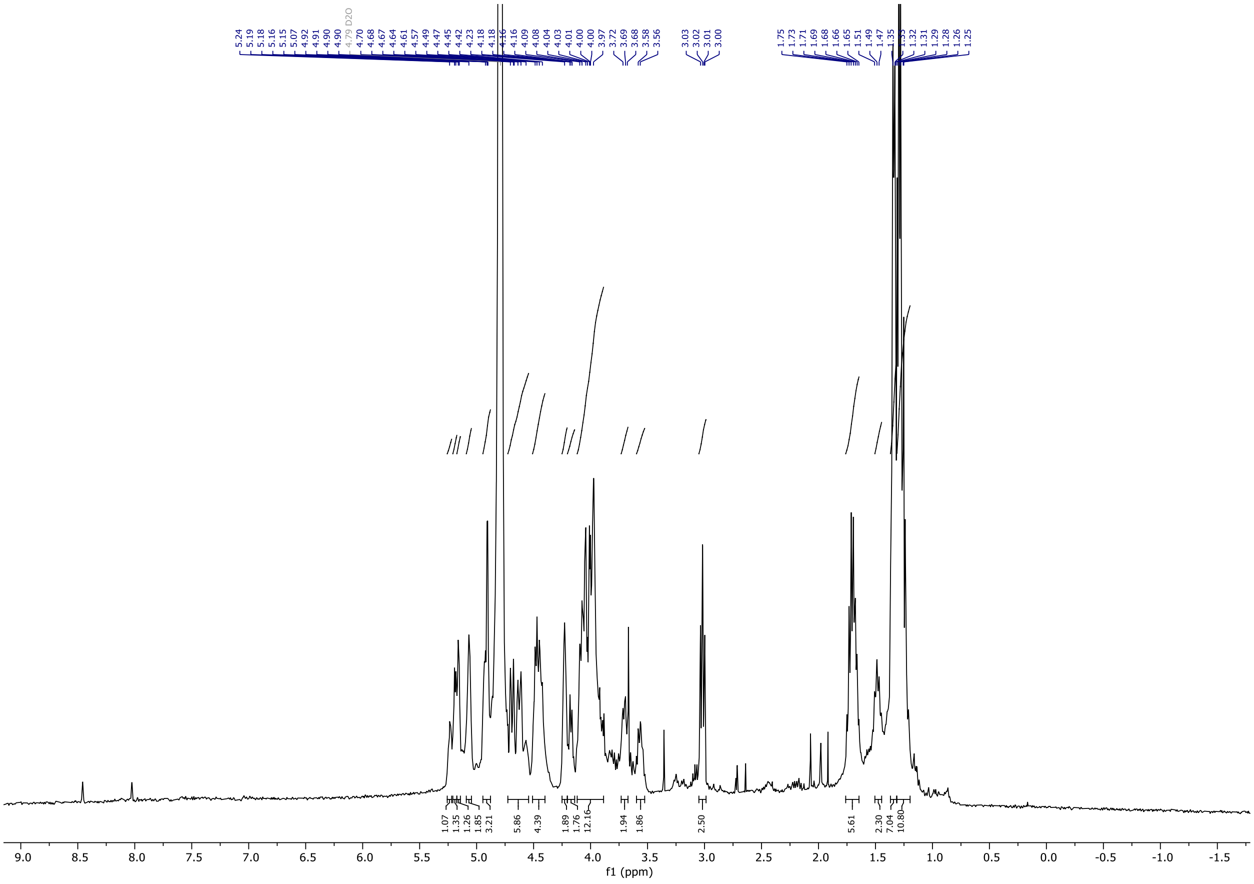

^{1}H NMR

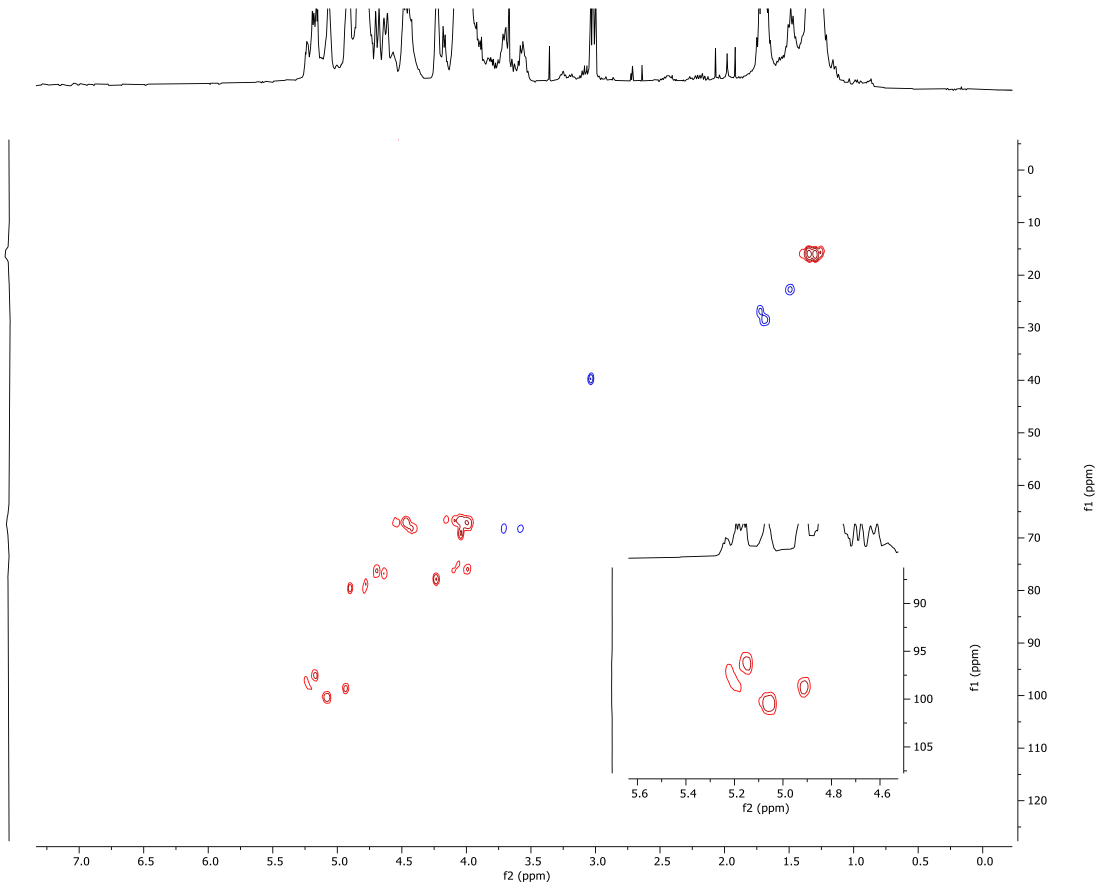

^{1}H-^{13}C HSQC NMR. Inset is of zoomed anomeric region.

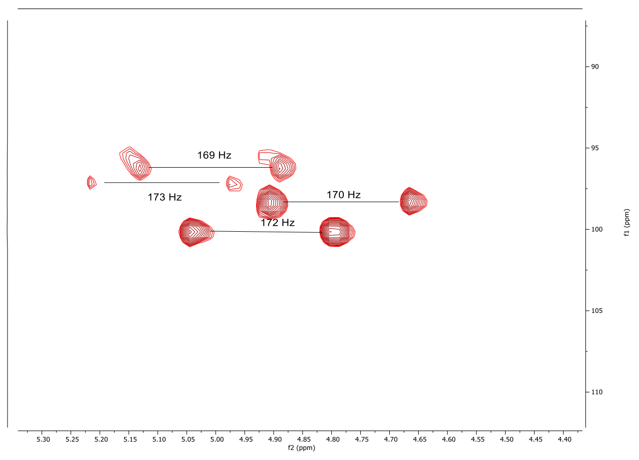

Coupled ^{1}H-^{13}C HSQC NMR.

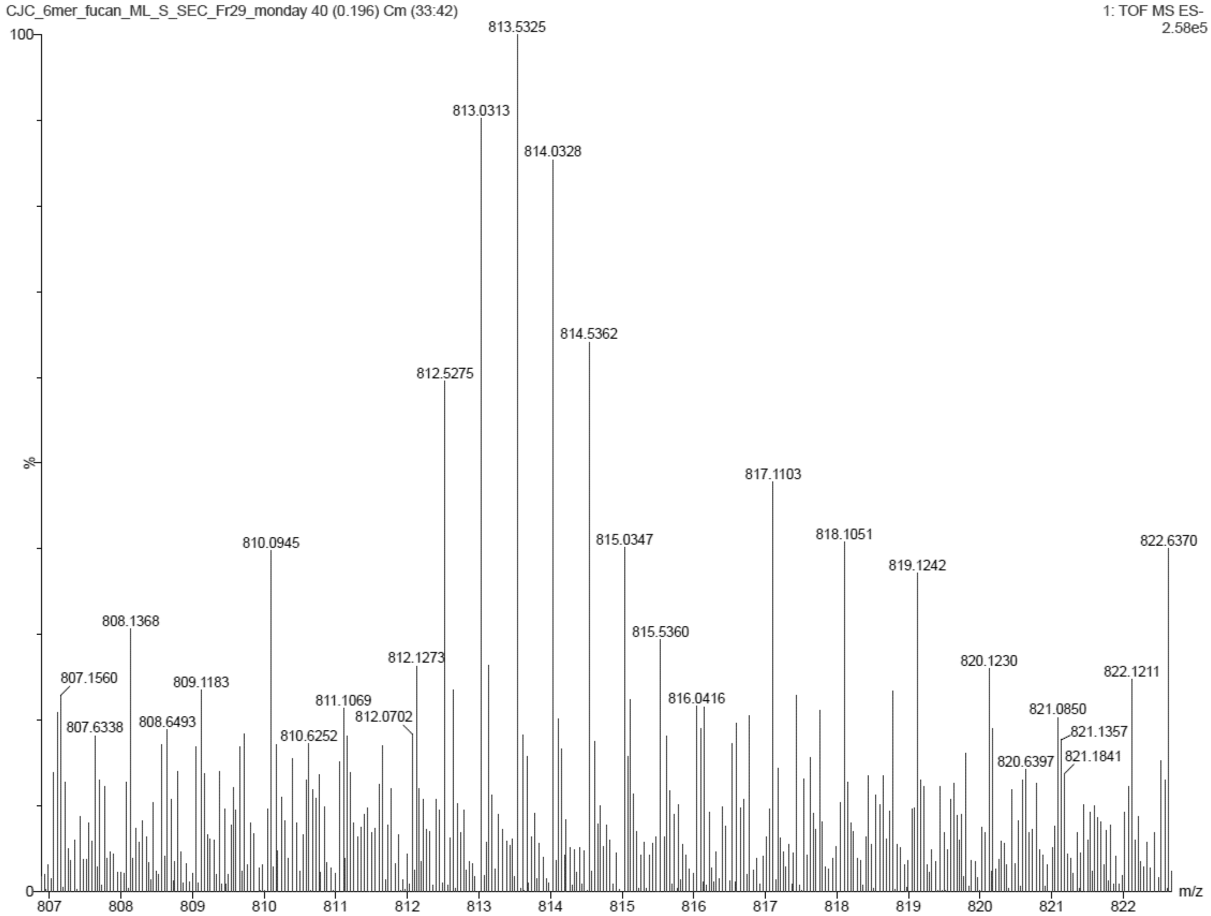
CJC_6mer_fucan_ML_S_SEC_Fr29_monday 40 (0.196) Cm (33:42)
1: TOF MS ES-
2.58e5
100
%
0
813.5325
813.0313
814.0328
814.5362
812.5275
817.1103
818.1051
815.0347
822.6370
810.0945
819.1242
808.1368
815.5360
812.1273
820.1230
807.1560
809.1183
822.1211
816.0416
811.1069
821.0850
807.6338
808.6493
812.0702
810.6252
821.1357
821.1841
820.6397
807
808
809
810
811
812
813
814
815
816
817
818
819
820
821
822
m/z

Q-TOF MS

5-aminopentyl α-L-fucopyranosyl-(1→3)-[α-L-fucopyranosyl-(1→2)]-α-L-fucopyranosyl-(1→3)-α-L-fucopyranosyl-(1→3)-α-L-fucopyranosyl-(1→3)-α-L-fucopyranoside (18)

1

3

L1

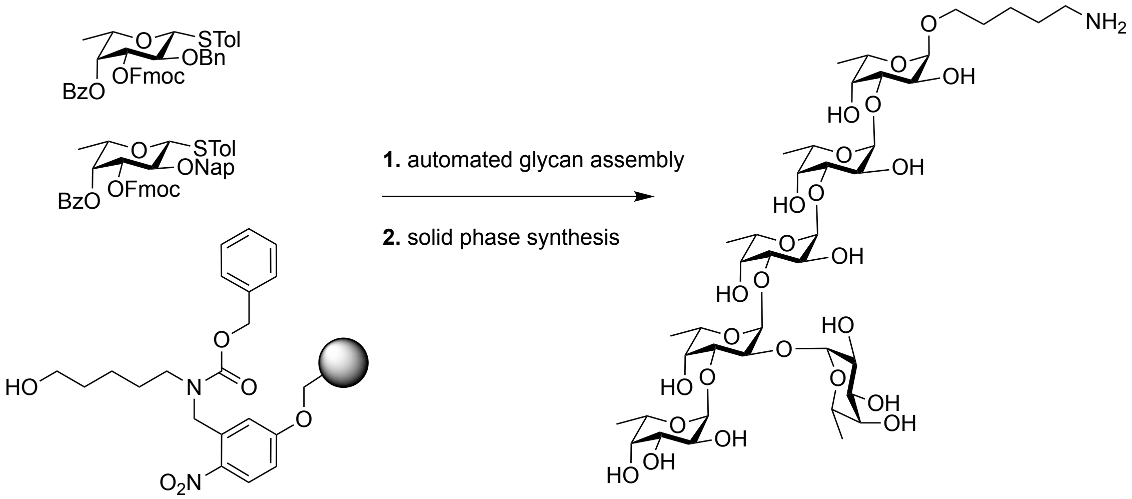

		Modules	Notes
1. AGA			L1
	1	a, b1, c, d	x3
	3	a, b1, c, f, d	x2 (double cycle)
	1	a, b1, c, d	x2
	**Step**	**Module**	**Notes**
**2. Solid-phase synthesis**	Methanolysis	g	
	photocleavage	i	
	**Step**	**Module**	**Notes**
**3. Solution-phase**	Hydrogenolysis	j1	
	Purification	Method 2	

The desired fractions were then collected and lyophilized to yield 1.5 mg (10%). **^{1}H NMR** (400 MHz, D_2O) δ 5.33 (d, *J* = 3.8 Hz, 1H, H-1, 1→2,3), 5.11 (ap t, *J* = 4.7 Hz, 2H, H-1, 1→3), 5.06 (s, 2H, H-1, 1→3), 4.87 (s, 2H, H-1, amino pentyl), 4.31 – 4.23 (m, 4H), 4.20 – 4.11 (m, 1H), 4.11 – 4.05 (m, 2H), 4.05 – 3.98 (m, 5H), 3.98 – 3.85 (m, 7H), 3.84 – 3.60 (m, 8H), 3.54 – 3.47 (m, 1H, $OC\mathit{H_2}CH_2CH_2CH_2CH_2NH_2$), 2.98 – 2.93 (m, 3H, $OCH_2CH_2CH_2CH_2C\mathit{H_2}NH_2$), 1.68 – 1.63 (m, 5H, $OCH_2\mathit{CH_2}CH_2\mathit{CH_2}CH_2NH_2$), 1.46 – 1.38 (m, 2H, $OCH_2CH_2C\mathit{H_2}CH_2CH_2NH_2$), 1.22 – 1.17 (m, 18H, CH_3). **^{13}C NMR**‡ (101 MHz, D_2O) δ 98.2 (C-1, amino pentyl), 95.15 (C-1, 1→3), 95.14 (C-1, 1→3), 94.3 (C-1, 1→3), 91.7 (C-1, 1→2,3), 74.5, 74.3, 71.6, 69.8, 68.0, 67.8, 67.5, 67.4, 66.4, 66.2, 66.13, 66.12, 39.1, 26.4, 22.4, 15.3 (CH_3). **HRMS** QTOF-MS: calcd. $C_{41}H_{74}NO_{25}$ for $[M+H]^+$ 980.4550, found 980.4559.

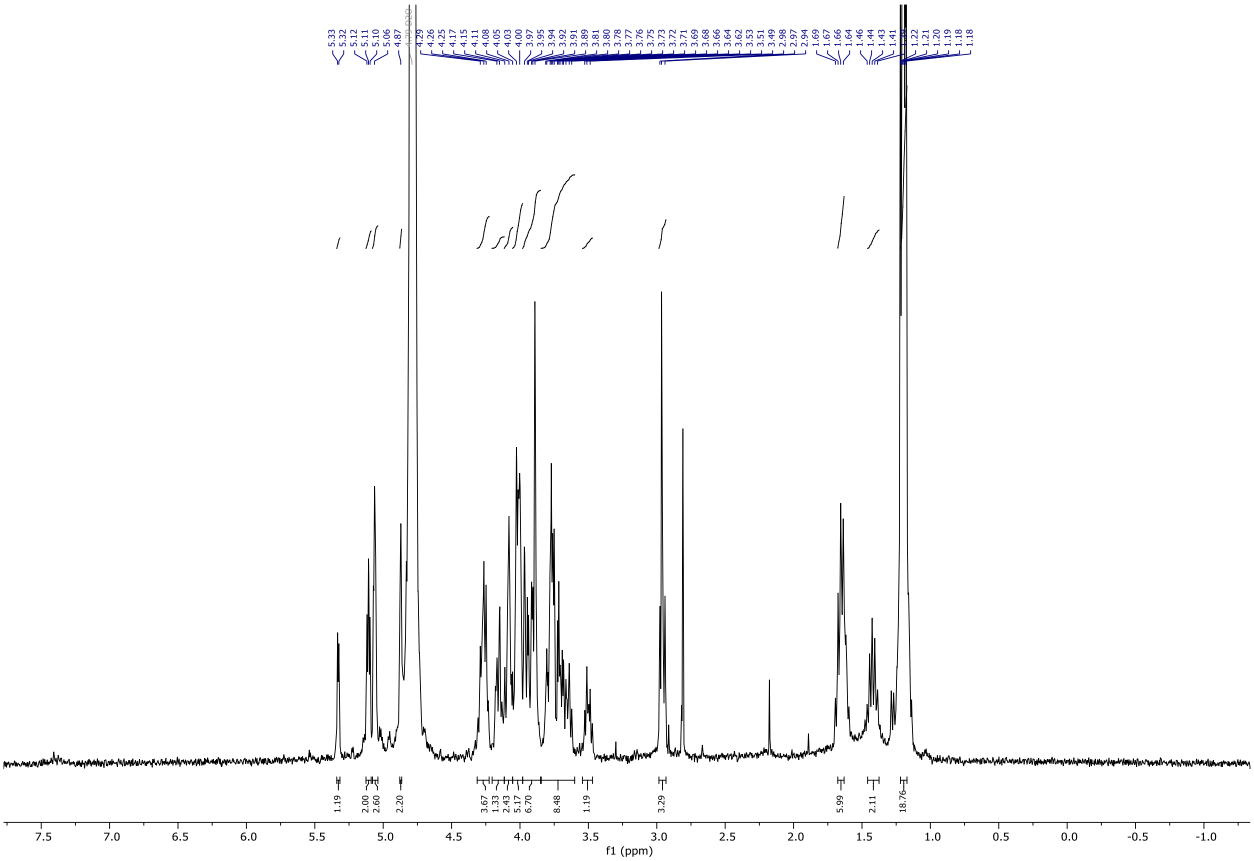

^{1}H NMR

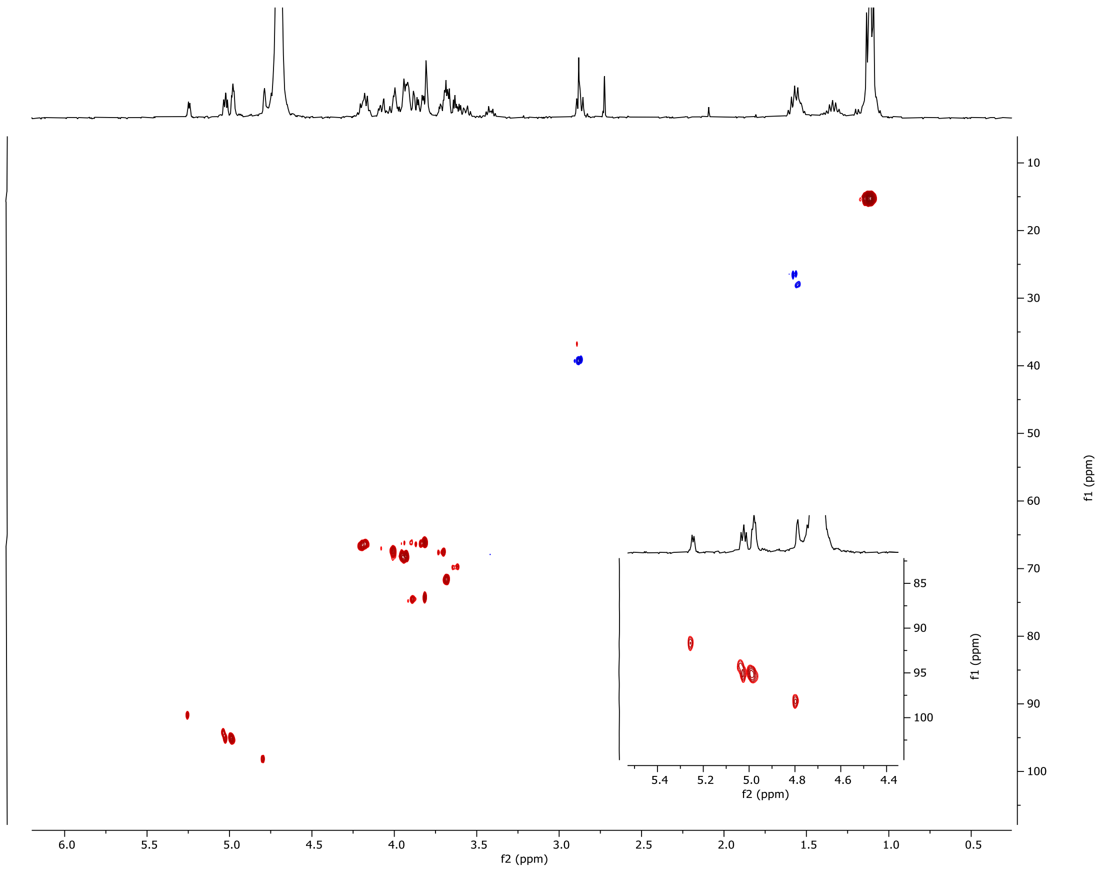

^{1}H-^{13}C HSQC NMR. Inset is of zoomed anomeric region.

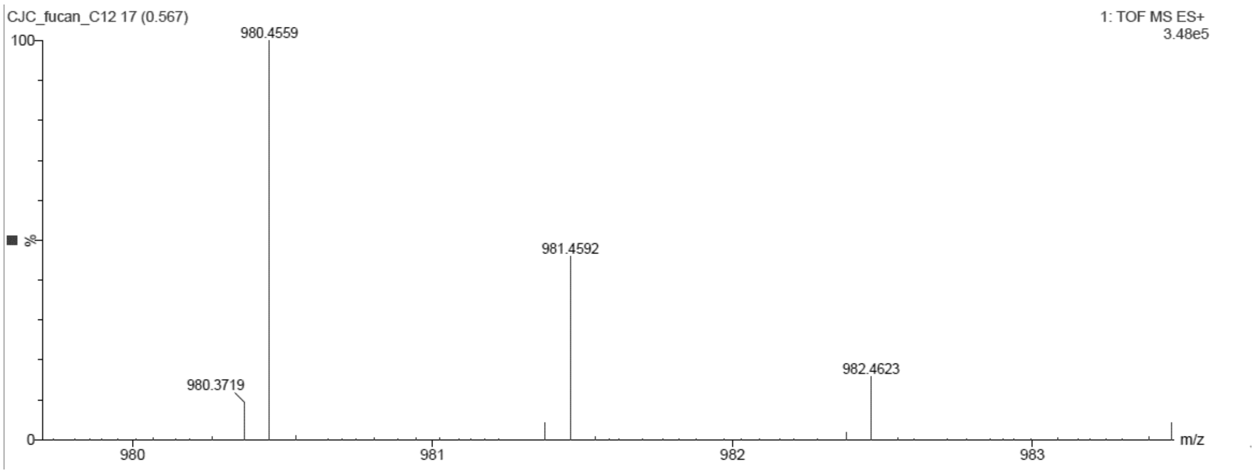

Q-TOF MS

5-aminopentyl α-L-fucopyranosyl-(1→3)-[β-D-galactosyl-(1→4)]-α-L-fucopyranosyl-(1→3)-α-L-fucopyranosyl-(1→3)-α-L-fucopyranoside (19)

1

2

5

L1

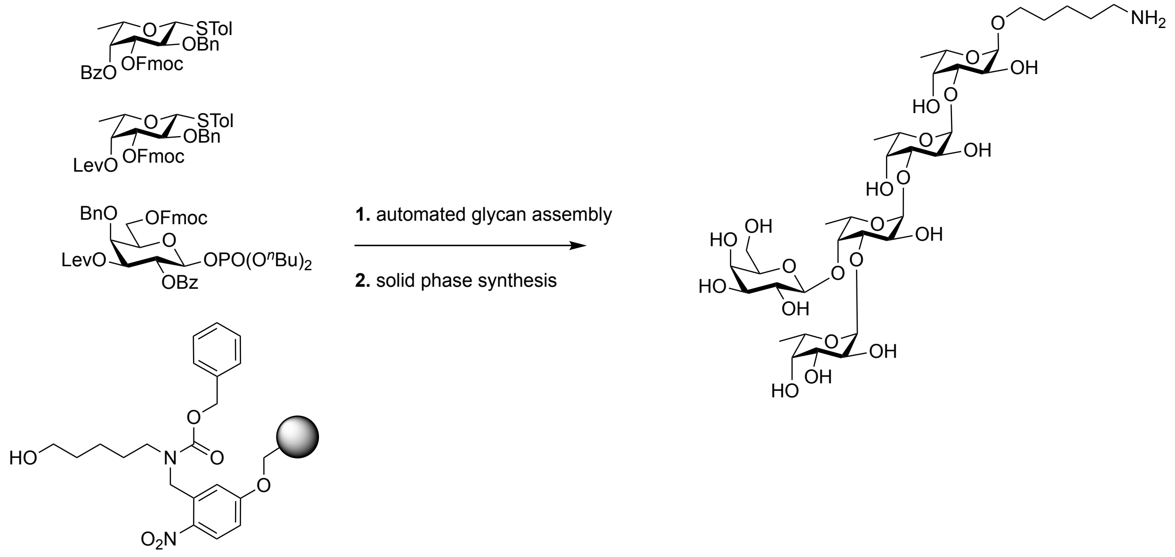

		Modules	Notes
**1. AGA**			L1
	**1**	a, b1, c, d2	x2
	**2**	a, b1, c, d2	x1
	**1**	a, b1, c, e	x1
	**5**	a, b2	x1
	**Step**	**Module**	**Notes**
**2. Solid-phase synthesis**	Methanolysis	g	
	photocleavage	i	
	**Step**	**Module**	**Notes**
**3. Solution-phase**	Hydrogenolysis	j1	
	Purification	Method 2	

The desired fractions were then collected and lyophilized to yield 0.9 mg (8%).**^{1}H NMR** (600 MHz, D_2O) δ 5.24 (d, *J* = 4.2 Hz, 1H, H-1, 1→3,4), 5.09 (d, *J* = 4.1 Hz, 1H, H-1, 1→3), 5.05 (d, *J* = 4.2 Hz, 1H, H-1, 1→3), 4.86 (s, 1H, H-1, amino pentyl), 4.54 (d, *J* = 7.4 Hz, 1H, β-H-1_{gal}), 4.36 – 4.28 (m, 2H), 4.28 – 4.23 (m, 1H), 4.22 – 4.15 (m, 2H), 4.02 – 3.96 (m, 6H), 3.93 (dd, *J* = 10.5, 4.0 Hz, 2H), 3.91 – 3.85 (m, 6H), 3.83 – 3.68 (m, 7H), 3.67 – 3.45 (m, 9H), 2.96 – 2.93 (m, 3H, $OCH_2CH_2CH_2CH_2C\mathit{H_2}NH_2$), 1.66 – 1.61 (m, 5H, $OCH_2C\mathit{H_2}CH_2C\mathit{H_2}CH_2NH_2$), 1.43 – 1.38 (m, 3H, $OCH_2CH_2C\mathit{H_2}CH_2CH_2NH_2$), 1.29 (d, *J* = 6.7 Hz, 3H, CH_3), 1.18 – 1.16 (m, 7H, CH_3), 1.15 (d, *J* = 6.6 Hz, 4H, CH_3).**^{13}C NMR‡** (176 MHz, D_2O) δ 103.4 (C-1_{gal}, 1→4), 98.0 (C-1, amino pentyl), 95.6 (C-1, 1→3), 95.0 (C-1, 1→3), 94.7 (C-1, 1→3,4), 75.2, 74.8, 74.2, 74.2, 72.9, 72.4, 71.8, 71.3, 69.7, 68.5, 68.2, 67.9, 67.9, 67.1, 66.9, 66.8, 66.4, 66.4, 66.1, 66.0, 60.8, 46.6, 39.2, 28.1, 26.3, 22.4, 19.8, 15.5 (CH_3), 15.2 (CH_3). **HRMS** QTOF-MS: calcd. $C_{35}H_{64}NO_{22}$ for $[M+H]^+$ 850.3920, found 850.3936.

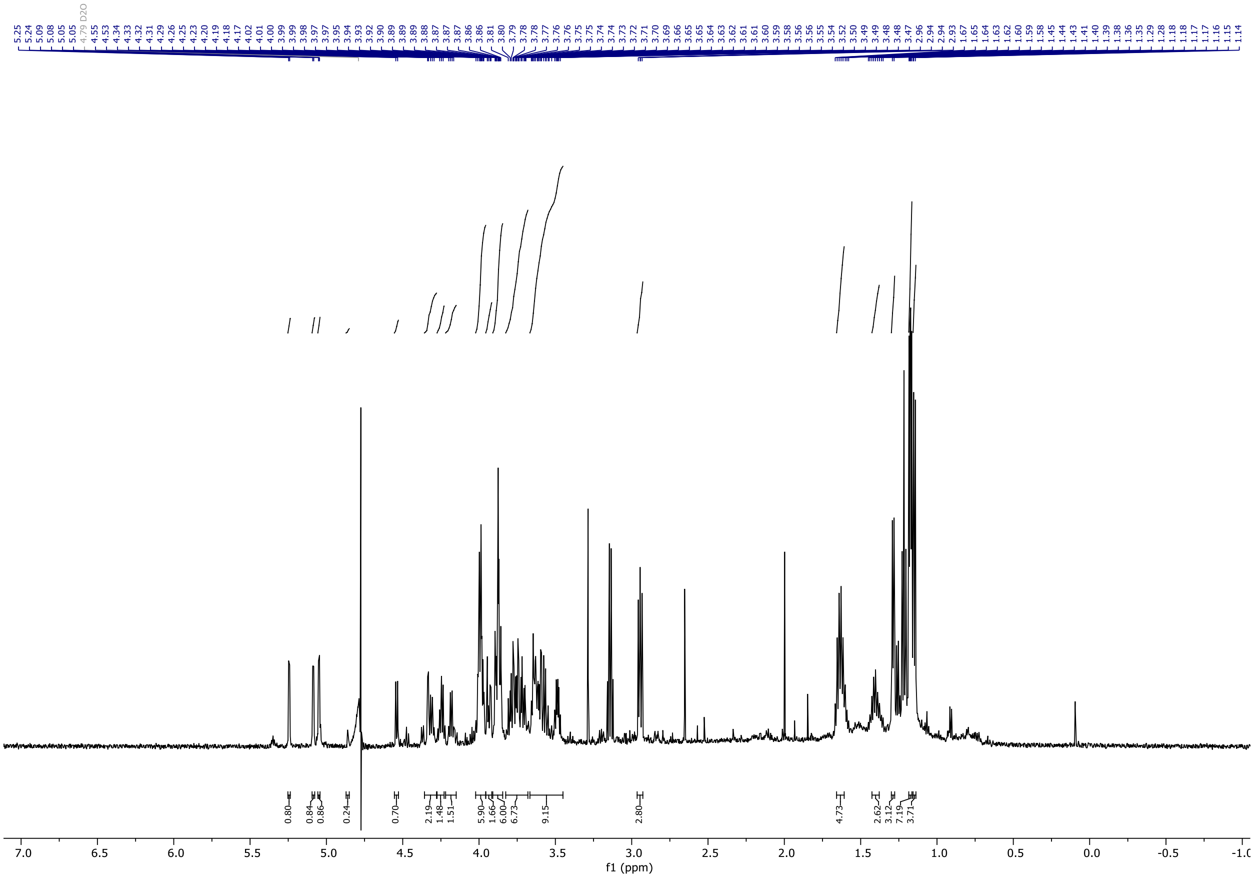

^{1}H NMR

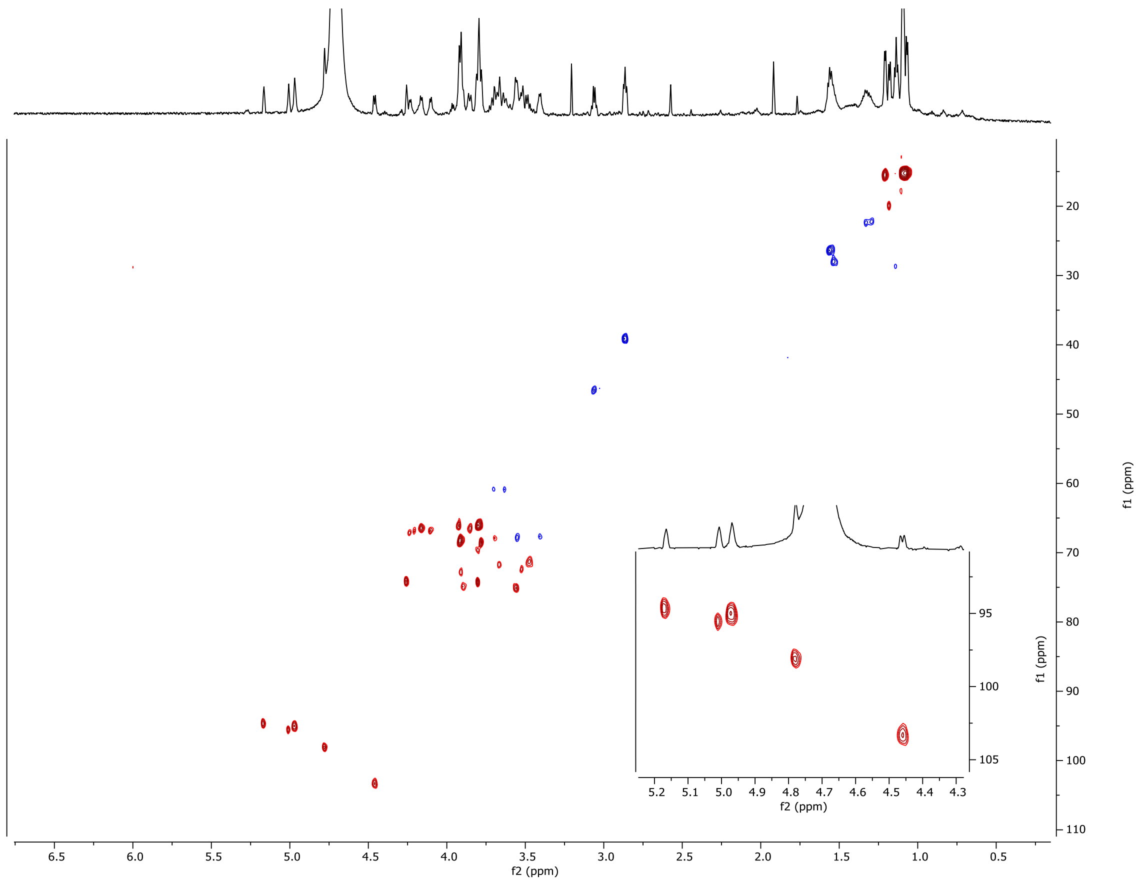

^{1}H-^{13}C HSQC NMR. Inset is of zoomed anomeric region.

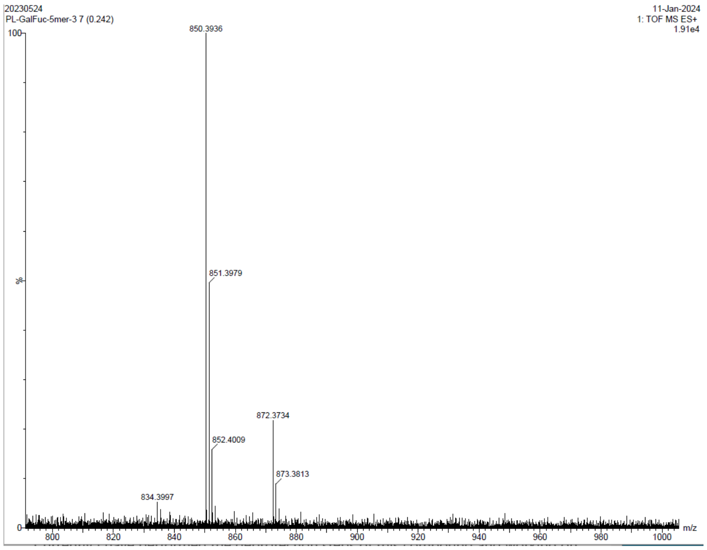

Q-TOF MS

5-aminopentyl β-D-galactosyl-(1→6)-β-D-galactosyl-(1→4)-[α-L-fucopyranosyl-(1→3)]-α-L-fucopyranosyl-(1→3)-α-L-fucopyranosyl-(1→3)-α-L-fucopyranoside (20)

1

2

5

L1

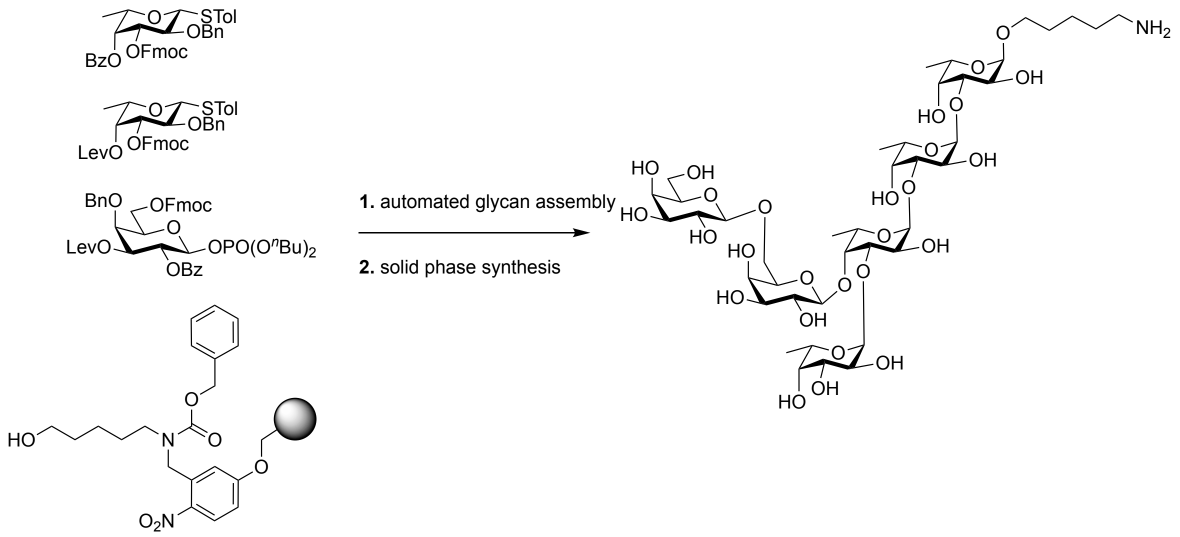

		Modules	Notes
**1. AGA**			L1
	**1**	a, b1, c, d2	x2
	**2**	a, b1, c, d2	x1
	**1**	a, b1, c, e	x1
	**5**	a, b2, c, d2	x1
	**5**	a, b2	x1
	**Step**	**Module**	**Notes**
**2. Solid-phase synthesis**	Methanolysis	g	
	photocleavage	i	
	**Step**	**Module**	**Notes**
**3. Solution-phase**	Hydrogenolysis	j1	
	Purification	Method 2	

The desired fractions were then collected and lyophilized to yield 0.5 mg (4%). **^{1}H NMR** (600 MHz, D_2O) δ 5.28 (d, *J* = 4.4 Hz, 1H, H-1, 1→3, 4), 5.10 (d, *J* = 4.0 Hz, 1H, 1H, H-1, 1→3), 5.06 (d, *J* = 4.0 Hz, 1H, 1H, H-1, 1→3), 4.87 (d, *J* = 3.3 Hz, 1H, H-1, amino pentyl), 4.58 (d, *J* = 7.6 Hz, 1H, 1H, β-H-1_{gal}, β-1→4_{fuc}), 4.41 – 4.37 (m, 3H, β-H-1_{gal}, β1→6_{gal}), 4.36 – 4.31 (m, 3H), 4.26 (q, *J* = 6.8 Hz, 3H), 4.20 (q, *J* = 6.7 Hz, 3H), 4.06 – 3.97 (m, 7H), 3.96 – 3.86 (m, 10H), 3.86 – 3.77 (m, 5H), 3.77 – 3.69 (m, 6H), 3.68 – 3.54 (m, 10H), 3.53 – 3.45 (m, 4H), 2.97 – 2.94 (m, 3H, $OCH_2CH_2CH_2CH_2C\mathit{H_2}NH_2$), 1.64 (d, *J* = 7.3 Hz, 4H, $OCH_2\mathit{CH_2}CH_2\mathit{CH_2}CH_2NH_2$), 1.43 – 1.40 (m, 3H, $OCH_2CH_2C\mathit{H_2}CH_2CH_2NH_2$), 2.97 –1.30 (d, *J* = 6.7 Hz, 3H, CH_3), 1.21 – 1.18 (m, 5H, CH_3), 1.16 (d, *J* = 6.5 Hz, 3H, CH_3). **^{13}C NMR‡** (151 MHz, D_2O) δ 103.3 (C-1_{gal}, β-1→4_{fuc}), 103.3(C-1_{gal}, β-1→6_{gal}), 98.3 (C-1, amino pentyl), 95.7 (C-1, 1→3), 95.1, 94.6 (C-1, 1→3,4), 75.2, 75.1, 74.3, 74.0, 73.8, 73.8, 72.8, 72.5, 71.9, 71.9, 71.5, 70.7, 69.0, 68.6, 68.5, 68.3, 68.0, 68.0, 67.0, 66.9, 66.7, 66.7, 66.2, 66.2, 60.9, 39.3, 28.2, 26.6, 22.4, 15.6 (CH_3), 15.5 (CH_3). **HRMS** QTOF-MS: calcd. $C_{41}H_{74}NO_{27}$ for $[M+H]^+$ 1012.4448, found 1012.4467.

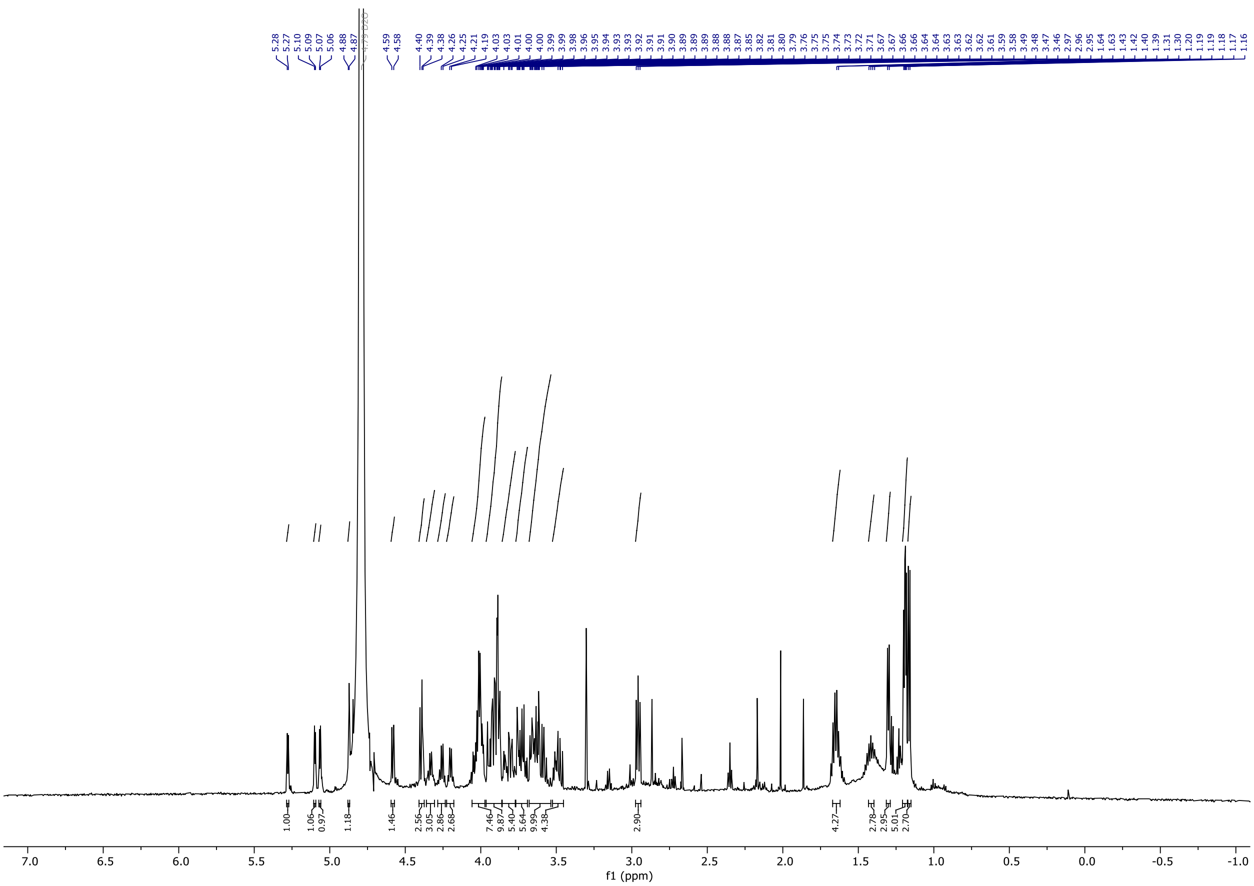

^{1}H NMR

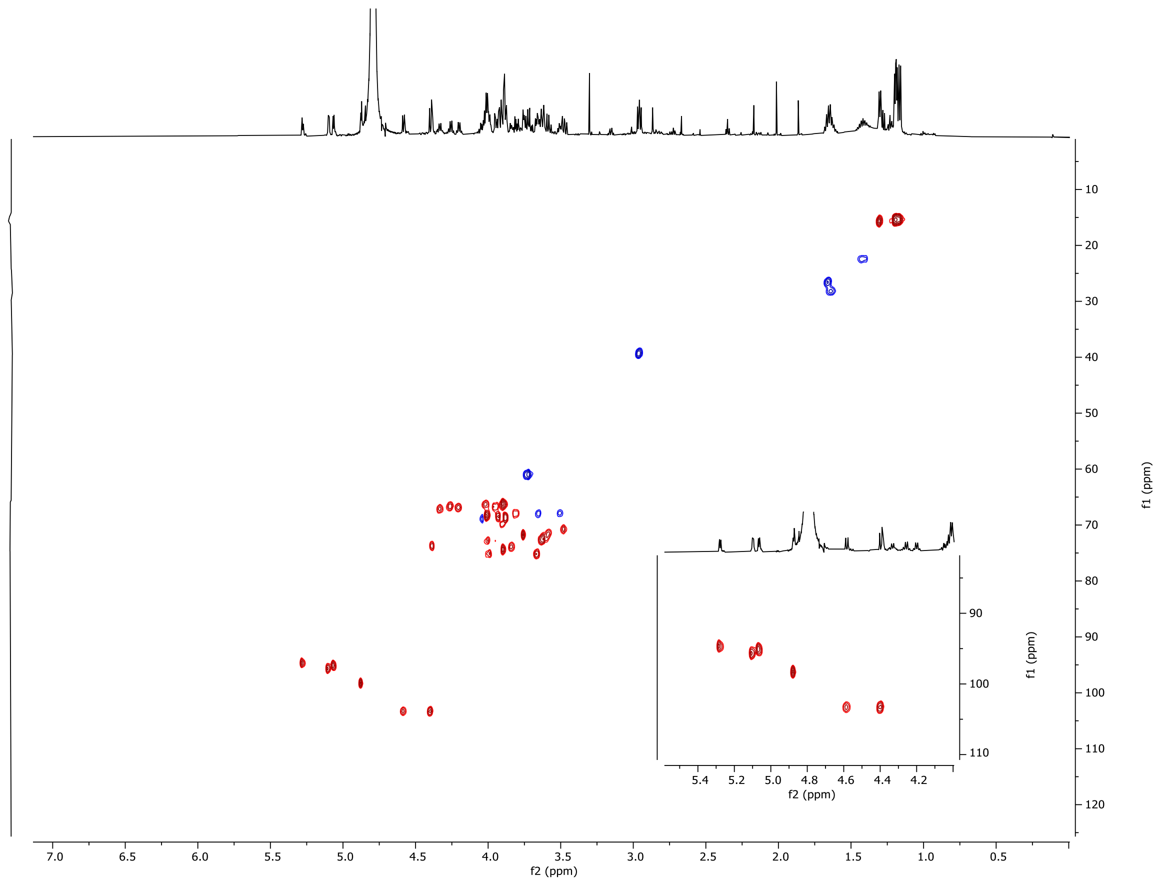

^{1}H-^{13}C HSQC NMR. Inset is of zoomed anomeric region.

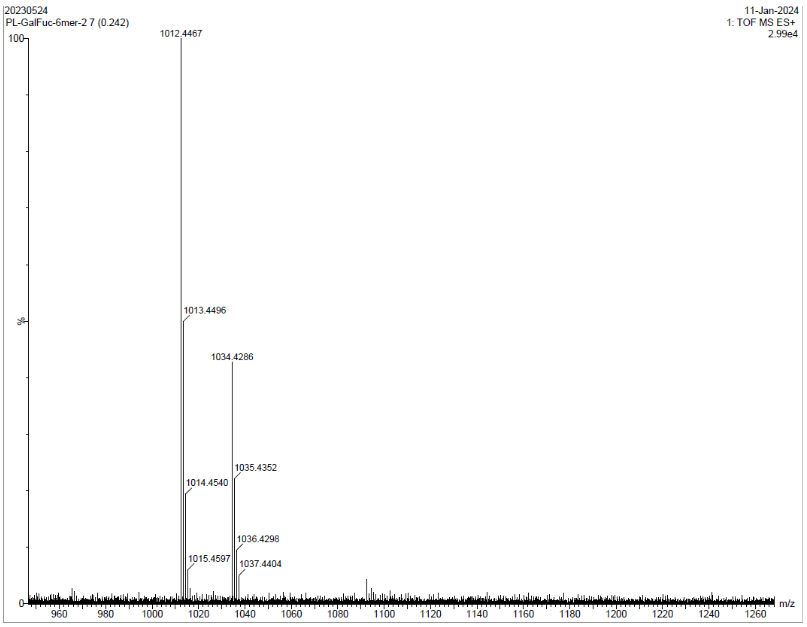

Q-TOF MS

5-aminopentyl α-L-fucopyranosyl-(1→3)-α-L-fucopyranosyl-(1→3)-[α-L-fucopyranosyl-(1→3)-α-L-fucopyranosyl-(1→4)]-α-L-fucopyranosyl-(1→3)-α-L-fucopyranoside (21)

1

2

L1

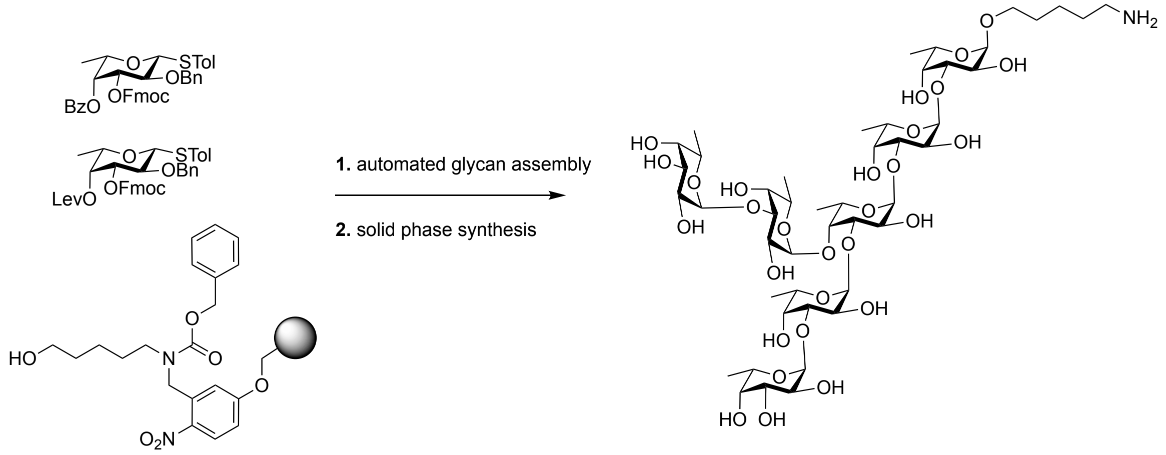

		**Modules**	**Notes**
**1. AGA**			L1
	**1**	a, b1, c, d1	x2
	**2**	a, b1, c, e, d1	
	**1**	a, b1, c, d1	x4
	**Step**	**Module**	**Notes**
**2. Solid-phase synthesis**	Methanolysis	g	32h
	photocleavage	i	
	**Step**	**Module**	**Notes**
**3. Solution-phase**	Hydrogenolysis	j1	
	Purification	Method 2	

The desired fractions were then collected and lyophilized to yield 1.6 mg (10%). **^{1}H NMR** (700 MHz, D_2O) δ 5.15 (d, *J* = 4.0 Hz, 1H, H-1, 1→4), 5.10 (s, 1H, 1H, H-1, 1→3), 5.08 – 5.06 (m, 2H, 1H, H-1, 1→3), 5.03 (d, *J* = 4.0 Hz, 2H, 1H, H-1, 1→3), 4.87 (d, *J* = 2.9 Hz, 1H, H-1, amino pentyl), 4.34 (m, 1H), 4.31 – 4.21 (m, 6H), 4.11 (s, 1H), 4.04 – 3.97 (m, 9H), 3.97 – 3.85 (m, 9H), 3.79 – 3.73 (m, 5H), 3.68 – 3.62 (m, 2H), 3.53 – 3.47 (m, 1H), 2.96 (t, *J* = 7.6 Hz, 3H, $OCH_2CH_2CH_2CH_2C\mathit{H_2}NH_2$), 1.68 – 1.63 (m, 5H, $OCH_2C\mathit{H_2}CH_2C\mathit{H_2}CH_2NH_2$), 1.47 – 1.37 (m, 2H, $OCH_2CH_2C\mathit{H_2}CH_2CH_2NH_2$), 1.33 (d, *J* = 6.6 Hz, 3H, CH_3), 1.24 (d, *J* = 6.5 Hz, 4H, CH_3), 1.20 – 1.18 (m, 11H, CH_3), 1.17 (d, *J* = 7.1 Hz, 3H,CH_3)**^{13}C NMR‡** (151 MHz, D_2O) δ 100.3 H-1, branched at 1,3,4), 98.2 (C-1, amino pentyl), 95.5 (C-1, 1→4), 95.3 (C-1, 1→3), 95.3 (C-1, 1→3), 95.0 (C-1, 1→3), 77.9, 74.5, 74.4, 71.8, 69.4, 68.2, 67.9, 67.9, 66.8, 66.6, 66.1, 39.2, 28.1, 26.5, 22.3, 16.0 (CH_3), 16.0 (CH_3), 15.4 (CH_3), 15.4 (CH_3). **HRMS** QTOF-MS: calcd. $C_{47}H_{84}NO_{29}$ for $[M + H]^+$ 1126.5124, found 1126.5215.

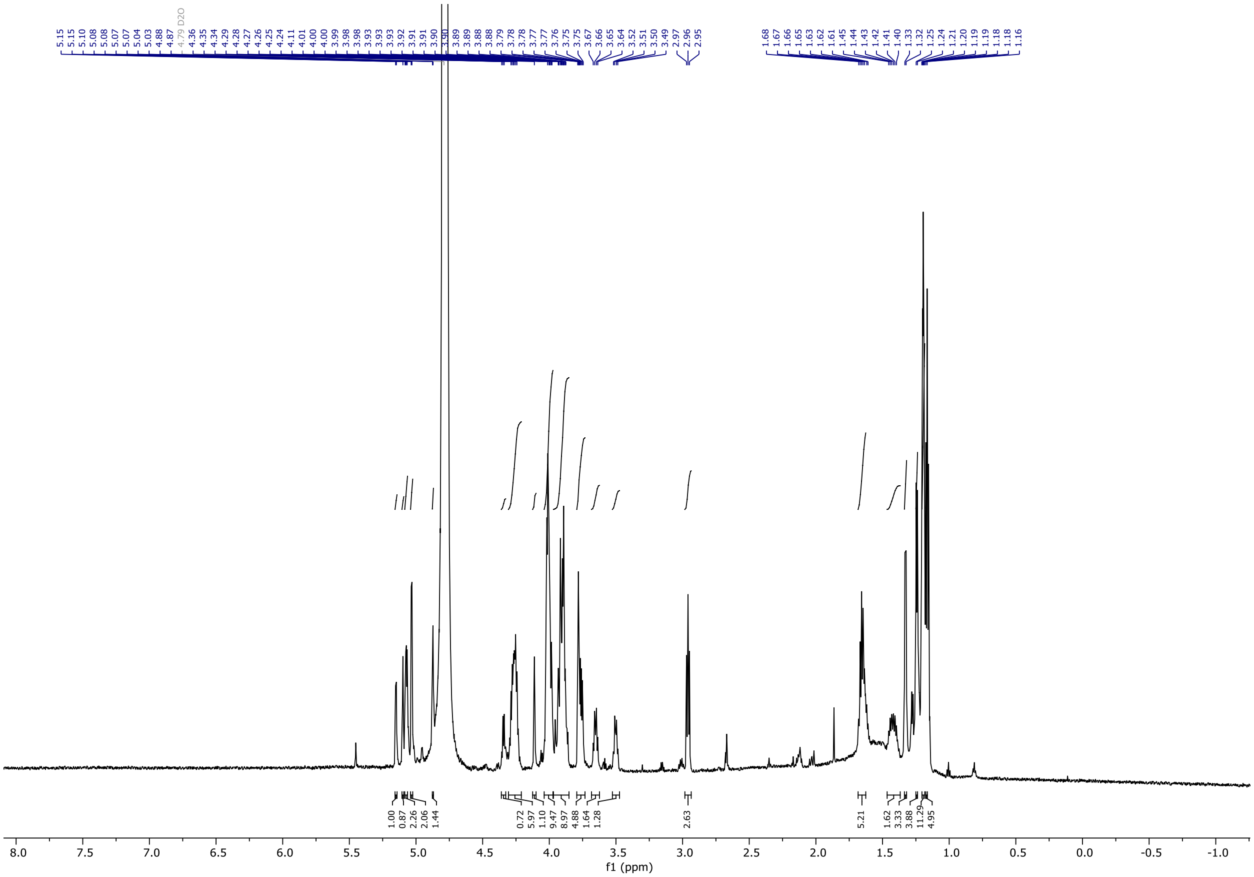

^{1}H NMR

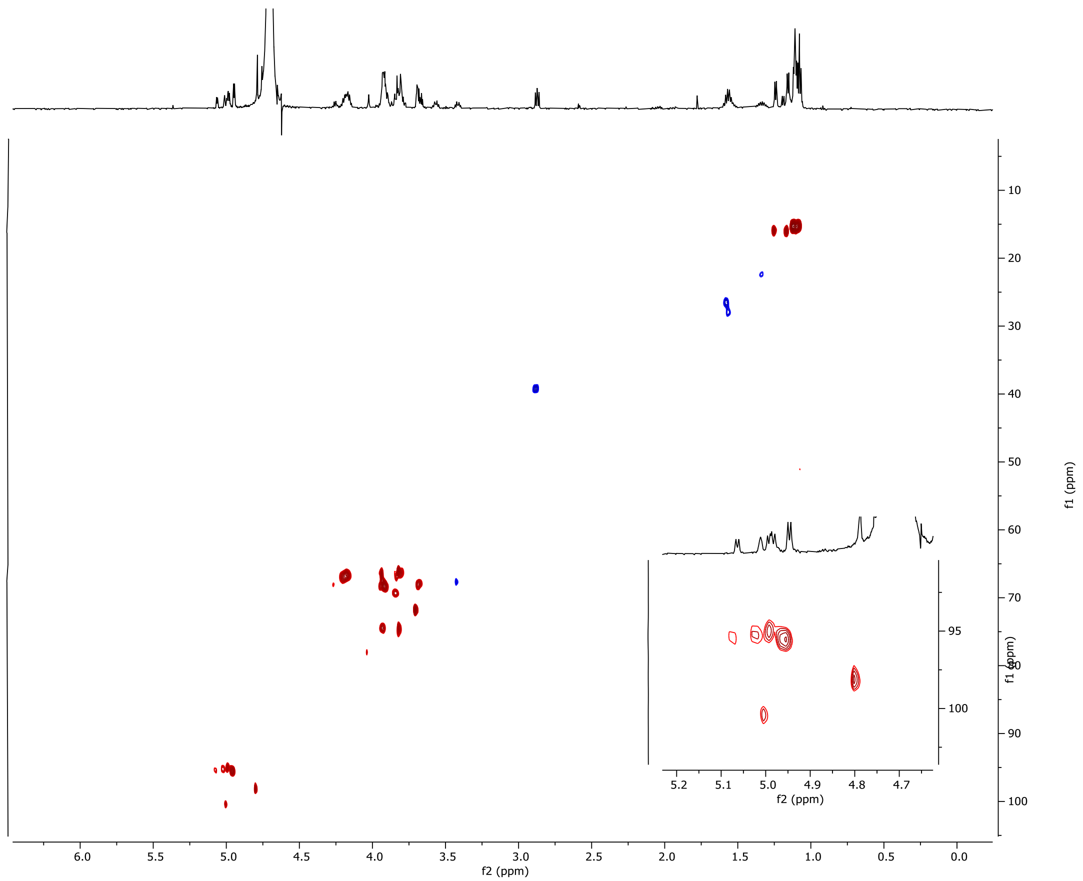

^{1}H-^{13}C HSQC NMR. Inset is of zoomed anomeric region.

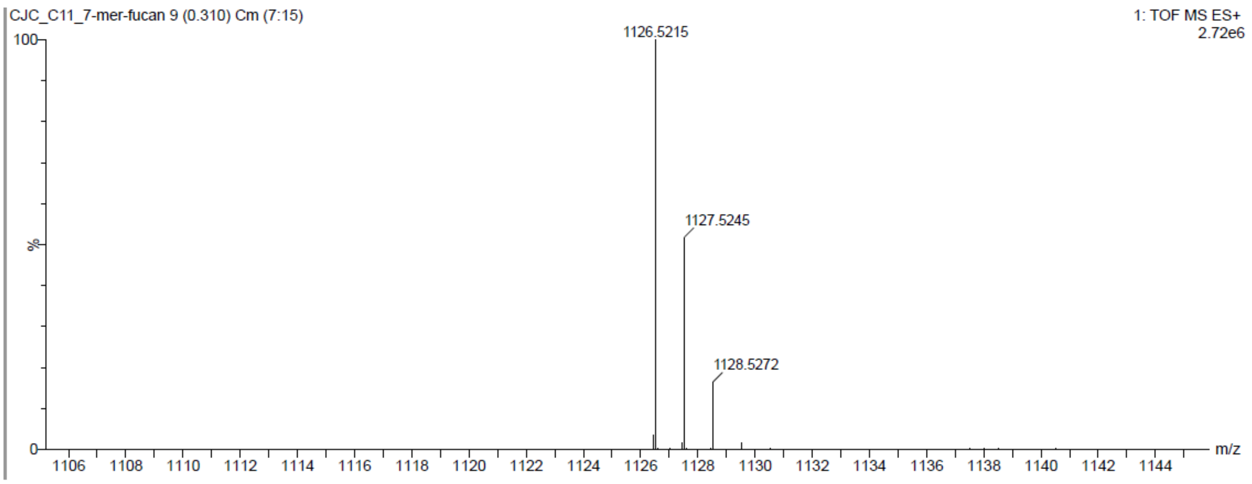

Q-TOF MS

References

(1) Tyrikos-Ergas, T.; Sletten, E. T.; Huang, J. Y.; Seeberger, P. H.; Delbianco, M. On Resin Synthesis of Sulfated Oligosaccharides. *Chem Sci* **2022**, *13* (7), 2115–2120. https://doi.org/10.1039/D1SC06063E.

(2) Ruhaak, L. R.; Deelder, A. M.; Wuhrer, M. Oligosaccharide Analysis by Graphitized Carbon Liquid Chromatography-Mass Spectrometry. *Anal Bioanal Chem* **2009**, *394* (1), 163–174. https://doi.org/10.1007/S00216-009-2664-5/FIGURES/4.

(3) Vickers, C.; Liu, F.; Abe, K.; Salama-Alber, O.; Jenkins, M.; Springate, C. M. K.; Burke, J. E.; Withers, S. G.; Boraston, A. B. Endo-Fucoidan Hydrolases from Glycoside Hydrolase Family 107 (GH107) Display Structural and Mechanistic Similarities to α-l-Fucosidases from GH29. *J Biol Chem* **2018**, *293* (47), 18296. https://doi.org/10.1074/JBC.RA118.005134.

(4) Sichert, A.; Corzett, C. H.; Schechter, M. S.; Unfried, F.; Markert, S.; Becher, D.; Fernandez-Guerra, A.; Liebeke, M.; Schweder, T.; Polz, M. F.; Hehemann, J. H. Verrucomicrobia Use Hundreds of Enzymes to Digest the Algal Polysaccharide Fucoidan. *Nature Microbiology 2020 5:8* **2020**, *5* (8), 1026–1039. https://doi.org/10.1038/s41564-020-0720-2.

(5) Reisky, L.; Stanetty, C.; Mihovilovic, M. D.; Schweder, T.; Hehemann, J. H.; Bornscheuer, U. T. Biochemical Characterization of an Ulvan Lyase from the Marine Flavobacterium Formosa Agariphila KMM 3901T. *Appl Microbiol Biotechnol* **2018**, *102* (16), 6987–6996. https://doi.org/10.1007/S00253-018-9142-Y.

(6) Vidal-Melgosa, S.; Sichert, A.; Francis, T. Ben; Bartosik, D.; Niggemann, J.; Wichels, A.; Willats, W. G. T.; Fuchs, B. M.; Teeling, H.; Becher, D.; Schweder, T.; Amann, R.; Hehemann, J. H. Diatom Fucan Polysaccharide Precipitates Carbon during Algal Blooms. *Nat Commun* **2021**, *12* (1). https://doi.org/10.1038/s41467-021-21009-6.
